# Supplementary material for: Transferable enantioselectivity models from sparse data
Source: Nature. 2026 Feb 11;651(8106):637–46. doi: 10.1038/s41586-026-10239-7 (PMC12999503; doi:10.1038/s41586-026-10239-7)
Supplement: Supplementary file 1 — This file contains Supplementary Information, including the following eight sections: 1. General information; 2. Computational workflow and benchmarking; 3. Case study 1; 4. Case study 2; 5. Case study 3; 6. Additional synthesis and characterization of ligands; 7. NMR spectra; and 8. References. [file 41586_2026_10239_MOESM1_ESM.pdf]

nature portfolio

https://doi.org/10.1038/s41586-026-10239-7

Supplementary information

Transferable enantioselectivity models from sparse data

In the format provided by the
authors and unedited

Supporting Information for

Transferable enantioselectivity models from sparse data

Simone Gallarati,[a,b,‡] Erin Bucci,[b,‡] Abigail G. Doyle,[b]* and Matthew S. Sigman[a]*

[a]Department of Chemistry, University of Utah, Salt Lake City, Utah 84112, United States

[b]Department of Chemistry and Biochemistry, University of California, Los Angeles, California 90095, United States

*Email: matt.sigman@utah.edu
*Email: abigaildoyle@g.ucla.edu

[‡]These authors contributed equally to this work

Table of Contents

1. General Information S4

1.1 Reagents, solvents, and other materials S4

1.2 Analytical instrumentation S4

2. Computational Workflow and Benchmarking S5

2.1 Data availability S5

2.2 Computational timings S5

2.3 Ligand structures and representations S6

2.4 Repeated, stratified, nested *k*-fold and 5×2 cross-validation schemes S7

2.6 Evaluation of alternative mechanistic pathways S13

3. Case Study 1 .... S14

3.1 Preparation and characterization of racemic products S14

3.2 Evaluation of chiral ligands S19

3.3 Additional ligand screening data S21

3.3 SFC determination of enantiomeric excess S23

3.4 MLR with TSRE-only features S37

3.5 MLR with TSRC–Int–TSRE features S38

3.6 Nonlinear parameters generation and modeling S41

3.7 Predictions on L1–L29 ligands set S44

3.8 Predictions on Virtual Ligands set S46

3.9 Non-linear regression S48

4. Case Study 2 .... S49

4.1 Modeling of C(sp^3)–H activation dataset (Model 1) S49

4.2 Synthesis and characterization of *N*-tosyl-2-phenyl aziridine S53

4.3 Preparation and characterization of racemic products S54

4.4 Evaluation of chiral ligands S58

4.5 SFC determination of enantiomeric excess S60

4.6 Modeling of Ni-catalyzed XECs (Model 2) S76

4.7 Feature analysis S81

5. Case Study 3 .... S82

5.1 Out-of-sample predictions using Model 2 .... S82

5.2 GPR model training S83

5.3 Preparation and characterization of racemic products S84

5.4 Evaluation of chiral ligands S86

6. Additional Synthesis and Characterization of Ligands S89
7. NMR Spectra.. S91
8. References... S106

1. General Information

1.1 Reagents, solvents, and other materials

Commercial reagents were purchased from Sigma-Aldrich, Strem, AmBeed, Combi-Blocks, or ChemScene and used as received. Chiral ligands were purchased from either Ambeed or ChemScene. **L16**, **L28**, **L29**, and **L144** were synthesized for a prior study reported by our lab[1] and used without further purification. Appropriate characterization data is included for ligands not obtained from commercial sources (Section 5). All reagents used in catalytic reactions (nickel precatalysts, ligands, 4-CzIPN, substrates, and reaction additives) were stored in a nitrogen-filled glovebox. Prior to use, tetrahydrofuran (THF) and N,N-Dimethylacetamide (DMA) were dried by passing through activated alumina columns under an argon atmosphere, sparged with nitrogen for 45 min, and stored over 4Å molecular sieves (1/16" pellets, activated by drying in a vacuum oven overnight) in the glovebox. Preparatory thin-layer chromatography was performed on Silicycle Glass Backed TLC extra hard layer (60Å, 1000 μM) plates and visualized with UV light (254 nm).

1.2 Analytical instrumentation

^{1}H NMR and ^{13}C NMR spectra were acquired in deuterated chloroform ($CDCl_3$) or (C_6D_6) purchased from Cambridge Isotope Laboratories, Inc. NMR experiments were performed on Bruker spectrometers operating at 300 and 500 MHz for ^{1}H experiments; 126 MHz for ^{13}C experiments; and 282 MHz for ^{19}F experiments. Chemical shifts (δ) are reported in ppm relative to the residual solvent peak (^{1}H NMR: δ 7.26 ppm for $CDCl_3$ and δ 7.16 ppm for C_6D_6; ^{13}C NMR: δ 77.16 ppm for $CDCl_3$ and δ 128.06 ppm for C_6D_6. ^{1}H NMR spectral data are reported as follows: chemical shift (multiplicity, coupling constants (where applicable), integration). Multiplicities are defined as follows: s = singlet, bs = broad singlet, d = doublet, t = triplet, q = quartet, p = pentet, h = hextet, hept = heptet, m = multiplet, dd = doublet of doublets, ddd = doublet of doublets of doublets, dddd = doublet of doublets of doublets of doublets, tt = triplet of triplets, dt = doublet of triplets, td = triplet of doublets, qd = quartet of doublets, heptd = heptet of doublets. Coupling constants (*J*) are given in hertz (Hz). ^{13}C NMR spectral data are reported in terms of chemical shift. High resolution mass spectra were obtained using a Thermo Scientific Thermo Exactive Plus MSD (DART-MS) equipped with an ID-CUBE ion source and a Vapur Interface (ION Sense Inc.) (atmospheric-pressure chemical ionization, APCI). FTIR spectra were obtained with an Agilent Cary 630 FTIR Spectrometer. Chiral SFC data was collected using an Agilent 1260 Infinity II SFC system with a binary pump and diode array detector with ChiralPak IB (3μm, 4.6 x 250 mm) or IG (3μm, 4.6 x 250 mm) columns. Photochemical reactions were run in Penn PhD (both M1 and M2 series) photoreactors equipped with 450 nm lamps using the following settings: 50% intensity, stirring at 800 rpm., fan speed at 5200 rpm. Optical rotations were taken with a Rudolph Research Analytical Autopol III polarimeter with Tungsten-Halogen (WI) lamp using a 0.5 dm/1 mL cell in spectral grade $CHCl_3$ that was neutralized by stirring with K_2CO_3 and then filtered.

2. Computational Workflow and Benchmarking

2.1 Data availability

A step by step guide on how structures were generated, optimized, and featurized, including the Bash and Python scripts as well as the *Get Properties* Jupyter notebook[2] used, are provided on GitHub at: https://github.com/SigmanGroup/HT_TSs_Opt. The folder "Step_1_Templates_Generation" contains the XYZ geometries used as AaronTools[3] templates for the different reactions (epoxide arylation for case study 1,[1] and reactions **A**–**H** for case study 2[4–10]). The "NN_Ligands" folder contains a ChemDraw file with the 2D representations of all the ligands investigated in this work, their code (**L1**–**L207**), and corresponding XYZ geometry. For case study 1, ligands structures are also shown in Figure S1 and Figure S10. The folder "Step_8_Modeling" contains the Python scripts for MLR analysis and the Excel spreadsheets with the input features. The folder "Step_9_Active_Learning" contains the Jupyter notebook used in the EDBO+ active learning[11] campaign and the input Excel spreadsheet with the training data and the features of the ligands for reaction I in the search space.

2.2 Computational timings

Despite the lower level of theory (*i.e.*, spGNF2-xTB[12]) needed to ensure cost-effectiveness, the computational workflow developed herein allows ensembles of transition states and catalytic cycle intermediates to be fully optimized (*i.e.*, to either a minimum or a first-order saddle point on a potential energy surface) and featurized at an affordable computational cost, compatible with high-throughput experimentation and data mining. Doyle *et al.*[1] reported that the time required to calculate free ligand, L*NiF_2, and L*Ni(*p*-tolyl)Cl features at the M06-2X/Def2-TZVP level of theory[13–15] was 3-12 hours, 16-60 hours, and 30-60 hours, respectively. Note that no conformational sampling was reported to have been performed on these structures. Instead, using the *xTB-Gaussian* wrapper[16] described at https://github.com/SigmanGroup/HT_TSs_Opt (see Step 4: Full Optimization), 9,933 reductive elimination transition states (substrates **1** + **2** with ligands **L1**–**L29**) were optimized in 1 day, 7 hours, 23 minutes, and 8 seconds. Similarly, features from 1,426 **TSRE** structures selected by *marc*[17] were calculated in 3 h, 7 minutes, and 17 seconds. These computations were submitted as a GNU parallel job on 1 computer node of the Notchpeak cluster of the Center for High Performance Computing at the University of Utah with 52 CPUs, 1 CPU per task. Structure generation and conformational sampling, performed with AaronTools[3] and Molassembler,[18] is also exceptionally fast: the aforementioned ensemble of **TSRE** geometries (**L1**–**L29** set) was generated in 3 min and 30 seconds by submitting the Python scripts available at https://github.com/SigmanGroup/HT_TSs_Opt (see Step 1 and 2) to 1 computer node of the Notchpeak cluster with 16 CPUs.

2.3 Ligand structures and representations

$\Delta\Delta G^{\ddagger}$ values for the enantioselective Ni/photoredox-catalyzed cross-coupling of aryl iodides and styrene oxides were taken from Doyle *et al.*[1] Ligands (see Figure S1) were classified as non-benzylic BiOx (**L2–3**, **L9–L15**), benzylic BiOx (**L1**, **L16–L25**), and BiIm (**L4–8**, **L26–29**).

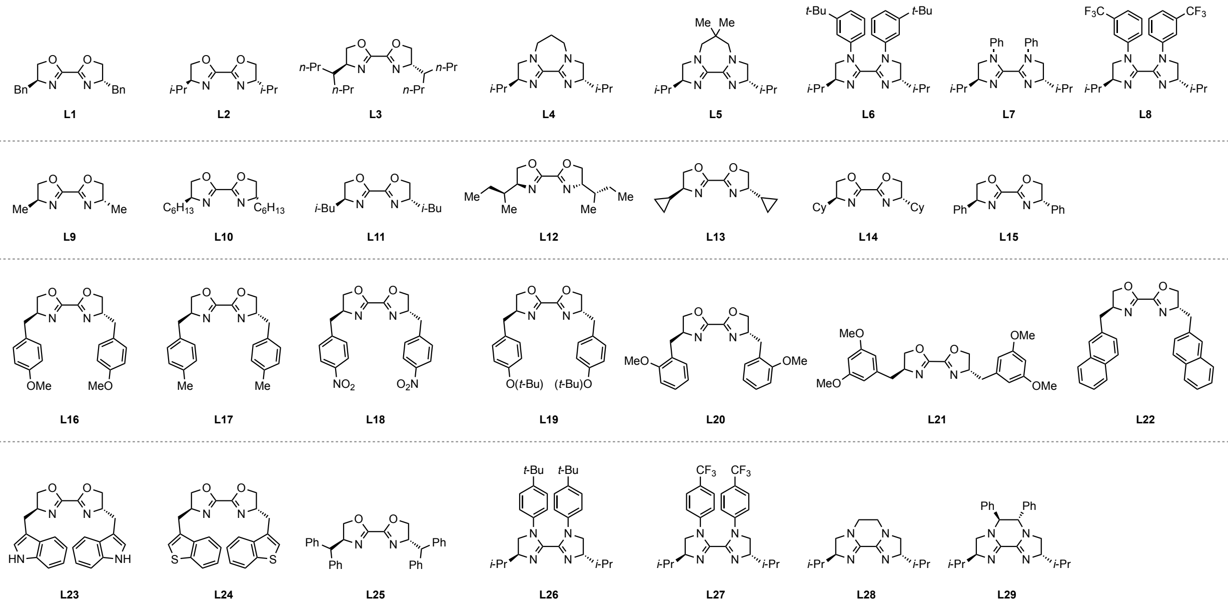

Figure S1. BiOx and BiIm ligands from Doyle *et al.*[1]

M06-2X/Def2-TZVP–level[13–15] features were taken from Doyle *et al.*[1] Features of **L1**–**L29** in the three coordination states (free ligand, L*NiF_2, and L*NiArCl, using ensembles of conformers) as well as ensembles of **TSRC**, **Int**, and **TSRE** were also computed at the spGFN2-xTB–level[12] with our newly developed workflow. The atom labels for local descriptors are shown in Figure S2.

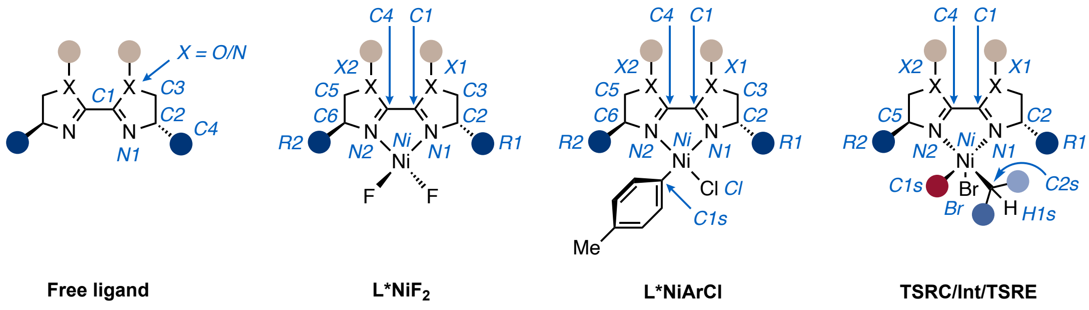

Figure S2. Atom labels for **L1**–**L29** in three coordination states and for **TSRC**, **Int**, and **TSRE**.

2.4 Repeated, stratified, nested *k*-fold and 5×2 cross-validation schemes

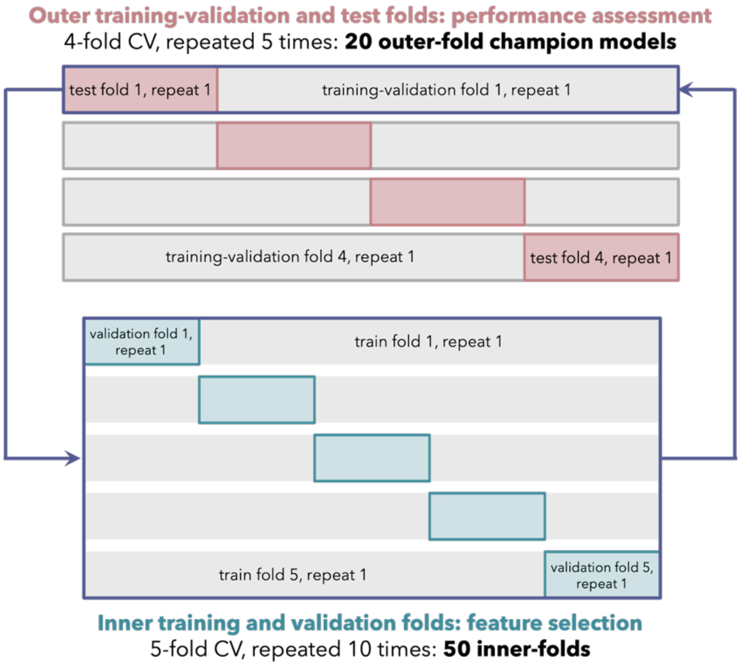

Figure S3. Repeated, stratified, nested *k*-fold CV scheme.

Multivariate linear regression was performed with our previously reported modeling workflow[1] (adapted into a Python script available on GitHub). The reaction output for modeling was the experimental $\Delta\Delta G^{\ddagger}$ values calculated using the formula $\Delta\Delta G^{\ddagger} = -\mathrm{RTln}|er|$ where R is the gas constant, T is temperature, and *er* is the enantiomeric ratio. Absolute values of enantiomeric ratios were used for modeling. In summary, a nested cross-validation (CV) scheme (Figure S3) was employed, which consists of an outer loop (4-fold CV, repeated 5 times) where the data is split into test and training/validation sets, and an inner loop (5-fold CV, repeated 10 times) which is used to select combinations of 3 features (out of 189 parameters for **TSRC**/**Int**/**TSRE**, 59 for Lig, 145 for L*NiF_2, or 152 for L*NiArCl at the spGFN2-xTB level) *via* an exhaustive search. In this case study, to reduce the computational cost of the exhaustive search on the training set, only the Boltzmann-weighted value of each descriptor was used. Descriptors were normalized and filtered according to collinearity *i.e.*, if two parameters had $R^2 \geq 0.7$, only the feature with higher R^2 with $\Delta\Delta G^{\ddagger}$ was retained. Each cross-validation fold (inner and outer) was stratified with respect to the ligand class (BiIm, BnBiOx, and non-BnBiOx). In each inner fold, the 5 models (*i.e.*, combinations of 3 features) that best minimized the residual sum of squares (RSS) of the training set were used to predict the $\Delta\Delta G^{\ddagger}$ of the validation set. The model with the lowest validation root mean square error (RMSE) was identified as the inner-fold-specific champion model. The process was repeated over the 50 inner-folds, and the model that appeared most frequently was considered the champion of that outer-fold. The outer-fold-specific champion was then retrained over the entire training/validation set, and its performance evaluated on the test set. The predictive accuracy of the outer-fold champion models was then evaluated *via* a 5×2 CV test[1] *i.e.*, a 2-fold cross-validation repeated 5 times, stratified by ligand class. Within a given repeat, pairs of RMSEs were averaged, and for each stationary point five test RMSEs were averaged and their standard deviation reported.[1] The model with the lowest mean test RMSE was selected as the dataset-specific champion model (Table S1). The parity plot for the model using features extracted from **TSRE** is shown in Figure S4.

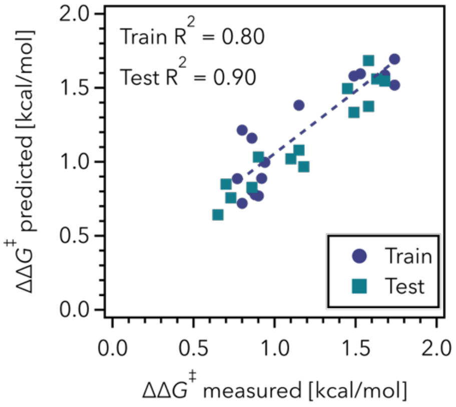

Figure S4. MLR model using **TSRE** features: $\Delta\Delta G^{‡} = 1.16 - 0.11 \times \eta^{Boltz} + 0.25 \times \%V_{bur\ 2.0\ Å\ (C1s)}{}^{Boltz} - 0.26 \times \%V_{bur\ 4.0\ Å\ (C2s)}{}^{Boltz}$. The mean value of the coefficients and intercepts across the 10 folds of the 5×2 CV scheme are reported; predicted $\Delta\Delta G^{‡}$ values are obtained using the averaged model. "Train" and "Test" labels correspond to the last *i.e.*, 10th fold of the 5×2 CV scheme.

Table S1. Repeated, stratified, nested *k*-fold CV results and 5×2 CV for comparing model performance. [a]Boltzmann-averaged values. [b]M06-2X/Def2-TZVP level features taken from Doyle *et al*.[1] (20 features for Ligand, 22 for L*NiF$_2$ and L*NiArCl); spGFN2-xTB level features computed using the newly developed workflow. [c]Results of the repeated, stratified nested *k*-fold CV scheme. [d]Results of the 5×2 CV scheme.

**Structure**	**Features[a]**	**Level[b]**	**Avg. Train RMSE[c]**	**Avg. Test RMSE[c]**	**Avg. Test Adj. R^{2}[c]**	**Test RMSE (mean, SD)[d]**
**Ligand**	Pol, $Vol_{avg\ X}$, NBO_{C4}	M06-2X	0.19	0.26	0.66	(0.27, 0.07)
**L*NiF$_2$**	HOMO, BL, NBO_{C4}	M06-2X	0.21	0.25	0.60	(0.28, 0.06)
**L*NiArCl**	Pol, NBO_{N1}, NBO_{C4}	M06-2X	0.17	0.19	0.74	(0.23, 0.04)
**Ligand**	P_{int}, $P_{int\ (N1)}$, $P_{int\ (C3)}$	spGFN2-xTB	0.20	0.30	0.64	(0.30, 0.04)
**L*NiF$_2$**	Hirsh charge$_{(X1)}$, Spin Density$_{(C4)}$, $P_{int(C4)}$	spGFN2-xTB	0.13	0.30	0.87	(0.23, 0.05)
**L*NiArCl**	$\Phi_{(C1–N1–C2–R1)}$, Buried Sterimol B5$_{(N2\rightarrow Ni,\ 4.5\ Å)}$, $P_{int\ (C1s)}$	spGFN2-xTB	0.12	0.32	0.88	(0.23, 0.06)
**TSRC**	HOMO, Hirsh CM5 charge$_{(X1)}$, Hirsh atom dipole$_{(R1)}$	spGFN2-xTB	0.15	0.21	0.79	(0.20, 0.05)
**Int**	HOMO, Hirsh CM5 charge$_{(X1)}$, Hirsh atom dipole$_{(R1)}$	spGFN2-xTB	0.15	0.26	0.80	(0.21, 0.05)
**TSRE**	η, $\%V_{bur\ 2.0\ Å\ (C1s)}$, $\%V_{bur\ 4.0\ Å\ (C2s)}$	spGFN2-xTB	0.14	0.18	0.83	(0.18, 0.02)

ANOVA with repeated measures was conducted at α = 0.05, with automatic Greenhouse-Geisser corrections applied to potential lack of sphericity. Results strongly suggested a rejection of the null hypothesis that mean test RMSE across the different structures and levels of theory are equal (F = 3.514, p = 0.00176, Table S2). Post-hoc pairwise comparisons with Holm-Bonferroni-adjusted paired-t tests revealed a significant difference between the performance of the **TSRE** model and the Ligand or the L*NiF_2 model with M06-2X/Def2-TZVP level features, and the **TSRC** models (Table S3).

Table S2. ANOVA (Repeated Measures) and Friedman Test.

**Test**	**Statistic**	***p***
One-Way Repeated Measures ANOVA	F = 3.514	0.00176
Friedman	χ^2 = 41.413	1.74×10^{-6}

Table S3. ANOVA repeated measures post-hoc pairwise *t* tests.

**post-hoc pairwise comparisons (repeated measures)**	***t***	***p* (Holm-Bonferroni-adjusted)**
Lig^{DFT} *vs*. $L*NiF_2^{DFT}$	0.13	1
Lig^{DFT} *vs*. $L*NiArCl^{DFT}$	3.98	0.10
Lig^{DFT} *vs*. Lig^{xTB}	-0.35	1
Lig^{DFT} *vs*. $L*NiF_2^{xTB}$	1.88	1
Lig^{DFT} *vs*. $L*NiArCl^{xTB}$	1.61	1
Lig^{DFT} *vs*. TSRC	3.83	0.12
Lig^{DFT} *vs*. Int	0.65	1
Lig^{DFT} *vs*. TSRE	5.54	0.01
$L*NiF_2^{DFT}$ *vs*. $L*NiArCl^{DFT}$	3.55	0.17
$L*NiF_2^{DFT}$ *vs*. Lig^{xTB}	-0.54	1
$L*NiF_2^{DFT}$ *vs*. $L*NiF_2^{xTB}$	2.43	0.91
$L*NiF_2^{DFT}$ *vs*. $L*NiArCl^{xTB}$	1.87	1
$L*NiF_2^{DFT}$ *vs*. TSRC	4.81	0.03
$L*NiF_2^{DFT}$ *vs*. Int	0.61	1
$L*NiF_2^{DFT}$ *vs*. TSRE	7.26	0.002
$L*NiArCl^{DFT}$ *vs*. Lig	-1.78	1
$L*NiArCl^{DFT}$ *vs*. $L*NiF_2^{xTB}$	0.07	1
$L*NiArCl^{DFT}$ *vs*. $L*NiArCl^{xTB}$	-0.13	1
$L*NiArCl^{DFT}$ *vs*. TSRC	1.67	1
$L*NiArCl^{DFT}$ *vs*. Int	-0.43	1
$L*NiArCl^{DFT}$ *vs*. TSRE	3.41	0.21
Lig^{xTB} *vs*. $L*NiF_2^{xTB}$	2.40	0.92
Lig^{xTB} *vs*. $L*NiArCl^{xTB}$	1.86	1
Lig^{xTB} *vs*. TSRC	3.10	0.33
Lig^{xTB} *vs*. Int	0.81	1
Lig^{xTB} *vs*. TSRE	4.24	0.07
$L*NiF_2^{xTB}$ *vs*. $L*NiArCl^{xTB}$	-0.16	1
$L*NiF_2^{xTB}$ *vs*. TSRC	1.43	1
$L*NiF_2^{xTB}$ *vs*. Int	-0.68	1
$L*NiF_2^{xTB}$ *vs*. TSRE	2.99	0.38
$L*NiArCl^{xTB}$ *vs*. TSRC	1.48	1
$L*NiArCl^{xTB}$ *vs*. Int	-0.31	1
$L*NiArCl^{xTB}$ *vs*. TSRE	3.65	0.15
TSRC *vs*. Int	-1.25	1
TSRC *vs*. TSRE	5.68	0.01
Int *vs*. TSRE	1.99	1

To assess the capability of the repeated, stratified nested *k*-fold CV strategy (Figure S3) to identify MLR models able to predict ligands outperforming those in the training data, we used the COBRA web application[19] (https://www.aocdweb.com/OMtools/cobra) to conduct a series of statistical tests on the MLR model using the **TSRE** features (*i.e.*, the top-performing model from the benchmarking study). These tests include leave-one-out CV, y-randomization, bootstrapping, predicting the top-performing ligands in the dataset, and simulating three cycles of catalyst optimization. While full details of these tests are given elsewhere (https://www.aocdweb.com/OMtools/cobra/help), the results are summarized in the synoptic radar plot shown in Figure S5 by visualizing deviations from the ideal behavior (*e.g.*, $R^2 = 1$). The radar plot indicates that the model passes all the tests, with small deviations from ideal behavior (compare Figure S5 with the prototype example reported by Cavallo *et al.*[19]). In alignment with the finding by Cavallo *et al.*,[19] y-randomization is the most problematic test for such a small dataset, but the high value of the 1–MAE_{T20}, Pre_{80}, Acc_{80}, and Rec_{80} tests (0.72, 0.85, 0.91, 1.00, respectively) supports using this model to predict better-performing ligands.

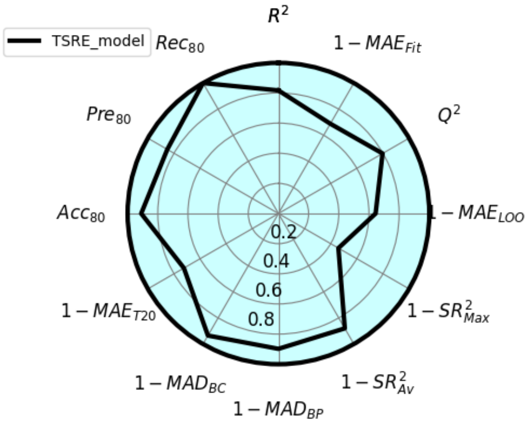

Figure S5. Synoptic radar plot summarizing the performance of the **TSRE** MLR model in all the statistical tests.

2.6 Evaluation of alternative mechanistic pathways

In this study, the widely proposed stepwise mechanism[20,21] for carbon–carbon bond formation was considered, leading to the generation and featurization of **TSRC**, **Int**, and **TSRE** structures. Recent work by Diao *et al.*[22] suggested that an alternative concerted inner-sphere radical capture and carbon–carbon bond formation process may be viable. To assess the energetic feasibility of this mechanism, we reexamined the potential energy surface computations reported by Doyle *et al.*[1] for the coupling of styrene oxides and aryl iodides. IRC computations were performed at the B3LYP/6-31G(d) level of theory[23,24] starting from **TSRC**, leading to the optimization of the Ni(II)–benzylic radical complex. A relaxed PES scan (Figure S6) was conducted on this structure, shortening the C(sp^2)–C(sp^3) distance in order to look for a guess structure from which the concerted TS for radical capture and carbon–carbon bond formation could be optimized. However, during the scan the complex collapsed to the Ni(III) intermediate (d ~ 2.7 Å), and subsequent scan steps afforded the reductive elimination TS. Optimizing structures with d ~ 3.3 Å afforded **TSRC**. These results suggest that, for this reaction and at the level of theory reported by Doyle *et al.*,[1] a concerted transition state cannot be located. We note that in this work, the structures reported by Doyle *et al.*[1] and by Reisman *et al.*[25] were used as templates (Figure 2, Step 1a) because the PES for the enantiodetermining steps had been computed, with the assumption that these steps are relevant to the reactions investigated in case study 2. Applying the Figure 2 workflow to a different reaction class would require a preliminary computational investigation of the stereodetermining step(s).

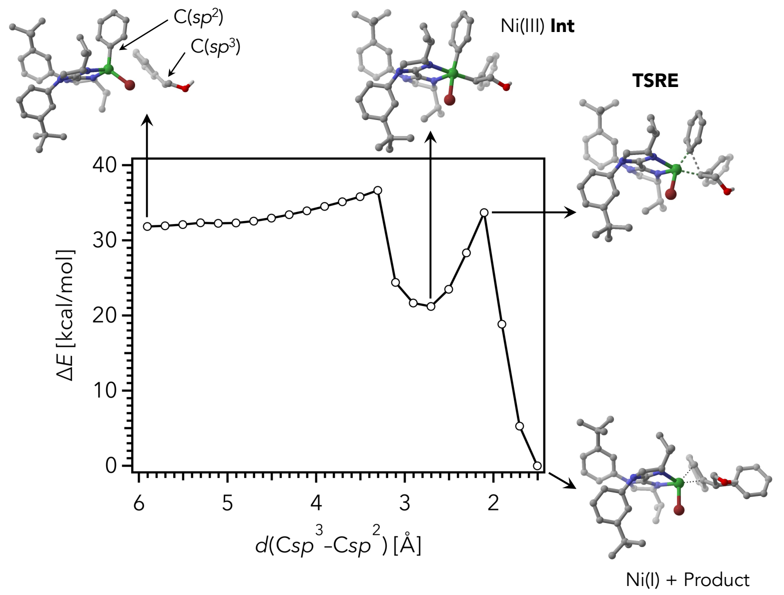

Figure S6. Relaxed PES scan at the B3LYP/6-31G(d) level shortening the distance between the C(sp^3) of the benzylic radical and the C(sp^2) of the Ni(II)–aryl species. The electronic energy (in kcal/mol) relative to the Ni(I)–product complex is reported on the y-axis.

3. Case Study 1

3.1 Preparation and characterization of racemic products

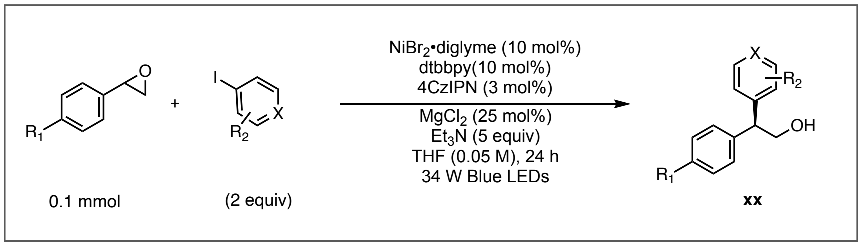

General Procedure A. This procedure was adapted from Doyle *et. al.*[1] In an N_2-filled glovebox, to an oven-dried one dram-vial equipped with a Teflon stir bar were added 4CzIPN (2.4 mg, 0.30 mmol, 0.030 equiv), styrene oxide (0.10 mmol, 1.0 equiv), aryl iodide (0.20 mmol, 2.0 equiv), $MgCl_2$ (2.38 mg, 0.25 mmol, 0.25 equiv), 1.50 mL THF, and triethylamine (69.7 µL, 0.500 mmol, 5.00 equiv). In a separate oven-dried one dram-vial equipped with a Teflon stir bar, $NiBr_2$·diglyme (2.7 mg, 0.010 mmol, 0.10 equiv) and 4,4'-*t*-Bu-bipyridine (24.3 mg, 0.010 mmol, 0.10 equiv) were prestirred in 0.50 mL THF for 20 minutes. The catalyst mixture was added to the reaction vial. The reaction vial was capped with a Qorpak PTFE lined cap, wrapped with electrical tape, and removed from the glovebox. The reaction vial was placed in a Penn PhD Photoreactor (M1 or M2) and run for 24 hours at 50% light intensity, 5200 rpm fan speed, and 800 rpm stirring rate. The crude reaction mixture was concentrated under reduced pressure and purified by preparative TLC.

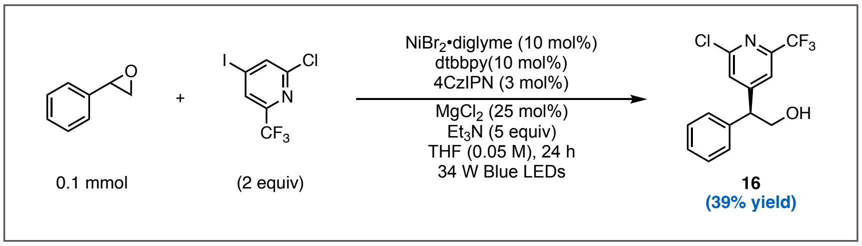

2-(2-chloro-6-(trifluoromethyl)pyridin-4-yl)-2-phenylethan-1-ol (**16**) was prepared according to General Procedure **A** from 2-phenyloxirane and 2-chloro-4-iodo-6-(trifluoromethyl)pyridine. The product was isolated by preparative TLC (20% acetone/hexanes) to yield the title compound as a yellow oil (11.6 mg, 39% yield). Characterization data are in agreement with reported literature values.[1]

^{1}H NMR (500 MHz, $CDCl_3$): δ 7.53 (d, J = 1.3 Hz, 1H), 7.46 (s, 1H), 7.42 – 7.37 (m, 2H), 7.35 – 7.31 (m, 1H), 7.21 – 7.17 (m, 2H), 4.28 – 4.18 (m, 3H), 1.58 (s, 1H).

^{13}C NMR (126 MHz, $CDCl_3$): δ 156.4, 152.4, 148.4 (q, J = 35.3 Hz), 138.4, 129.5, 128.5, 128.2, 127.3, 120.9 (q, J = 273.9 Hz), 119.5 (q, J = 2.9 Hz), 65.1, 52.7.

^{19}F NMR (282 MHz, $CDCl_3$): δ –67.90.

SFC method: Daicel ChiralPak IB column, 10% *i*-PrOH, 21 min run, 1.5 mL/min.

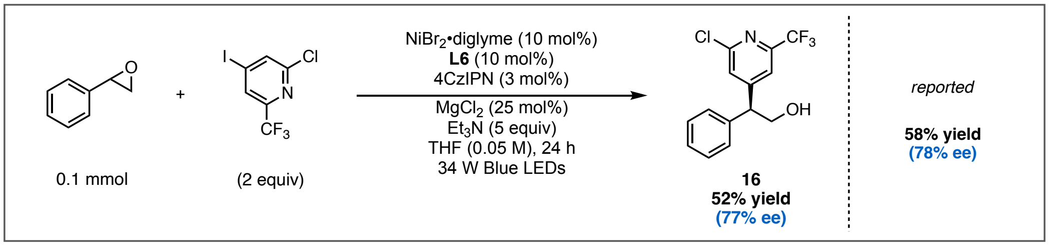

To ensure reproducibility between published data and our reaction set up, the standard reaction conditions using the reported optimal ligand **L6** were replicated prior to evaluating additional ligands. Measured *ee* agreed with reported *ee*.[1]

Racemic standard:

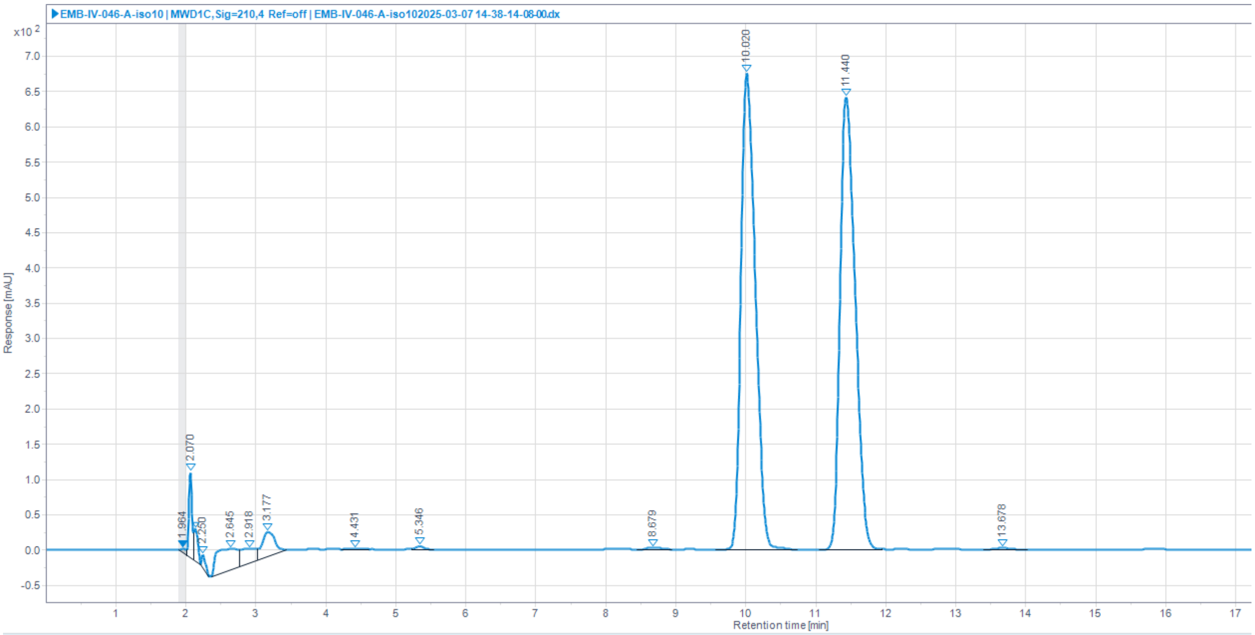

Injection Results

Peaks | Summary

#	Name	Signal description	RT (min)	Area (mAU·s)	Area%	Height (mAU)	Height%	Amount	Concentration	Start time (min)	End time (min)
11		MWD1C,Sig=210,4 Ref=off	10.020	9636.498	35.997	675.612	35.29			9.580	10.741
12		MWD1C,Sig=210,4 Ref=off	11.440	9635.556	35.994	641.679	33.52			11.058	11.946

L6: (77% *ee*)

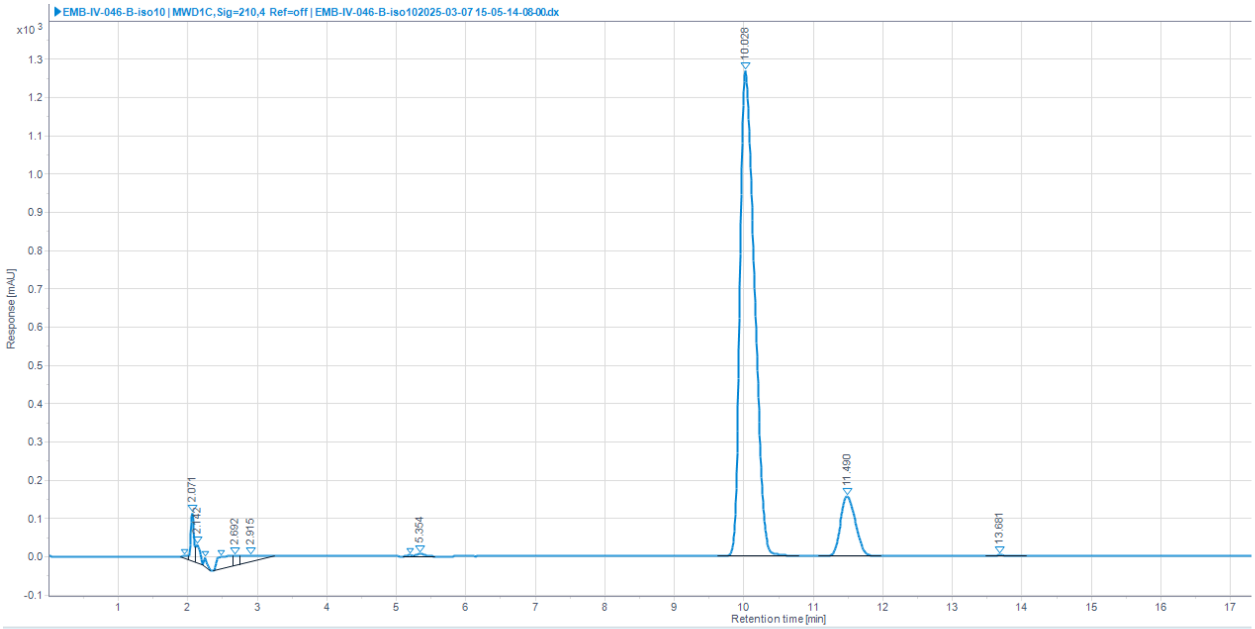

Injection Results

Peaks | Summary

#	Name	Signal description	RT (min)	Area (mAU·s)	Area%	Height (mAU)	Height%	Amount	Concentration	Start time (min)	End time (min)
10		MWD1C,Sig=210,4 Ref=off	10.028	18271.936	67.390	1268.055	63.91			9.630	10.794
11		MWD1C,Sig=210,4 Ref=off	11.490	2336.450	8.617	157.171	7.92			11.090	11.984

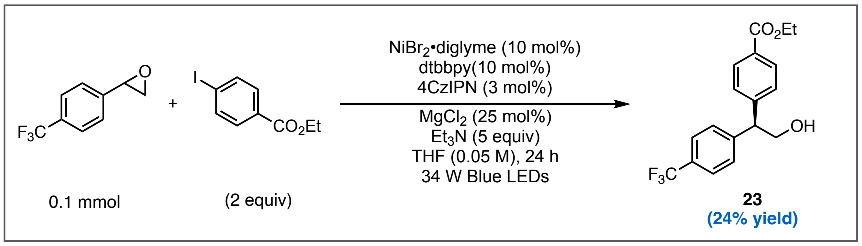

Ethyl 4-(2-hydroxy-1-(4-(trifluoromethyl)phenyl)ethyl)benzoate (**23**) was prepared according to General Procedure **A** from 2-(4-(trifluoromethyl)phenyl)oxirane and ethyl 4-iodobenzoate. The product was isolated by preparative TLC (17% acetone/hexanes) to yield the title compound as a yellow oil (8.1 mg, 24% yield). Characterization data are in agreement with reported literature values.[1]

^{1}H NMR (500 MHz, $CDCl_3$): δ 8.01 (dt, J = 8.5, 2.0 Hz, 2H), 7.61 – 7.56 (m, 2H), 7.40 – 7.35 (m, 2H), 7.34 – 7.31 (m, 2H), 4.37 (q, J = 7.1 Hz, 2H), 4.34 – 4.31 (m, 1H), 4.26 – 4.21 (m, 2H), 1.38 (t, J = 7.1 Hz, 3H).

^{13}C NMR (126 MHz, $CDCl_3$): δ 166.7, 146.1, 145.3, 130.5, 129.9, 129.8 (q, J = 32.4 Hz), 129.1, 128.7, 126.1 (q, J = 3.8 Hz), 124.5 (q, J = 272.1 Hz), 66.0, 61.4, 53.7, 14.7.

^{19}F NMR (282 MHz, $CDCl_3$): δ –62.52.

SFC method: Daicel ChiralPak IB column, 7% *i*-PrOH, 31 min run, 1.5 mL/min.

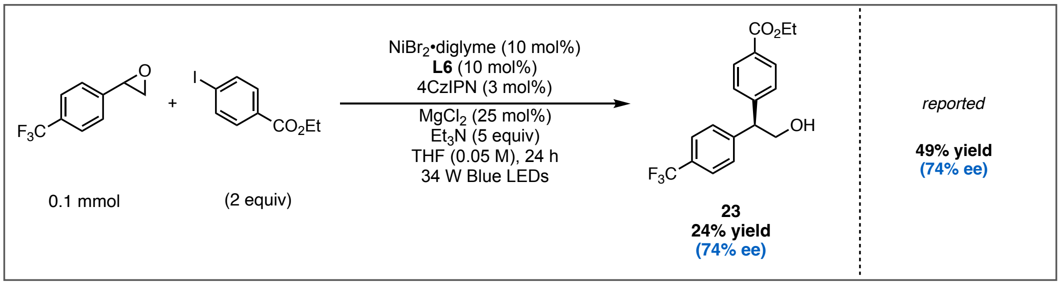

To ensure reproducibility between published data and our reaction set up, the standard reaction conditions using the reported optimal ligand **L6** were replicated prior to evaluating additional ligands. Measured *ee* agreed with reported *ee*.[1]

Racemic standard:

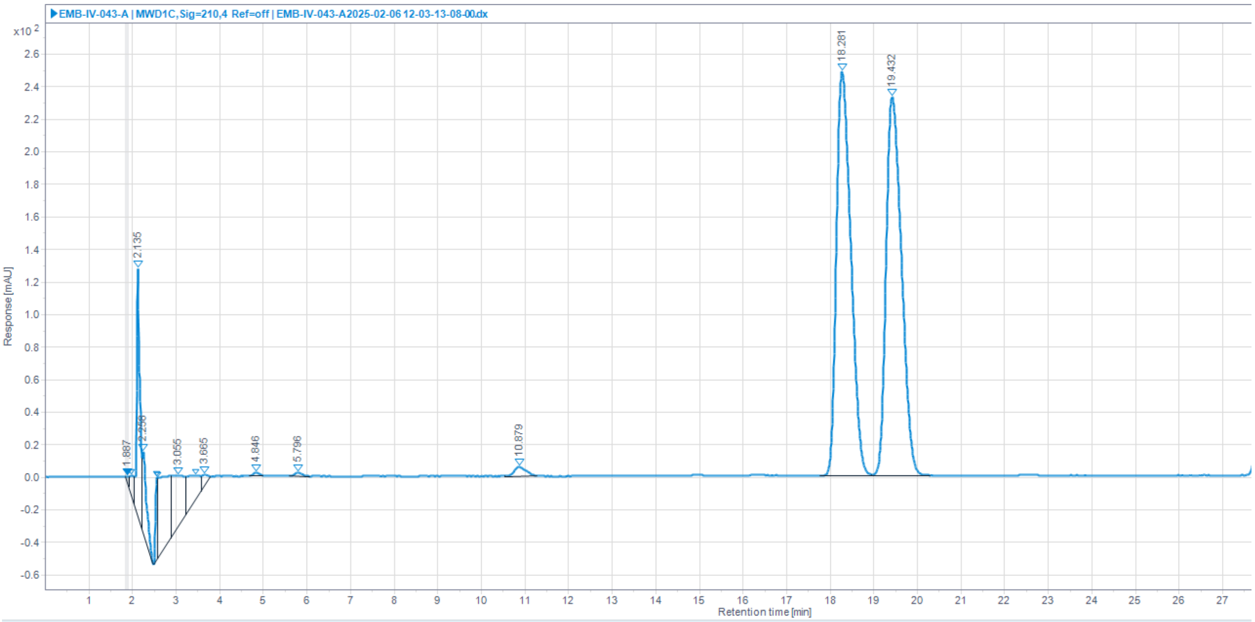

Injection Results

Peaks Summary

#	Name	Signal description	RT (min)	Area (mAU·s)	Area%	Height (mAU)	Height%	Amount	Concentration	Start time (min)	End time (min)
13		MWD1C,Sig=210,4 Ref=off	18.281	5710.977	30.659	248.120	19.82			17.770	18.966
14		MWD1C,Sig=210,4 Ref=off	19.432	5710.519	30.657	232.663	18.59			18.966	20.301

L6: (74% *ee*)

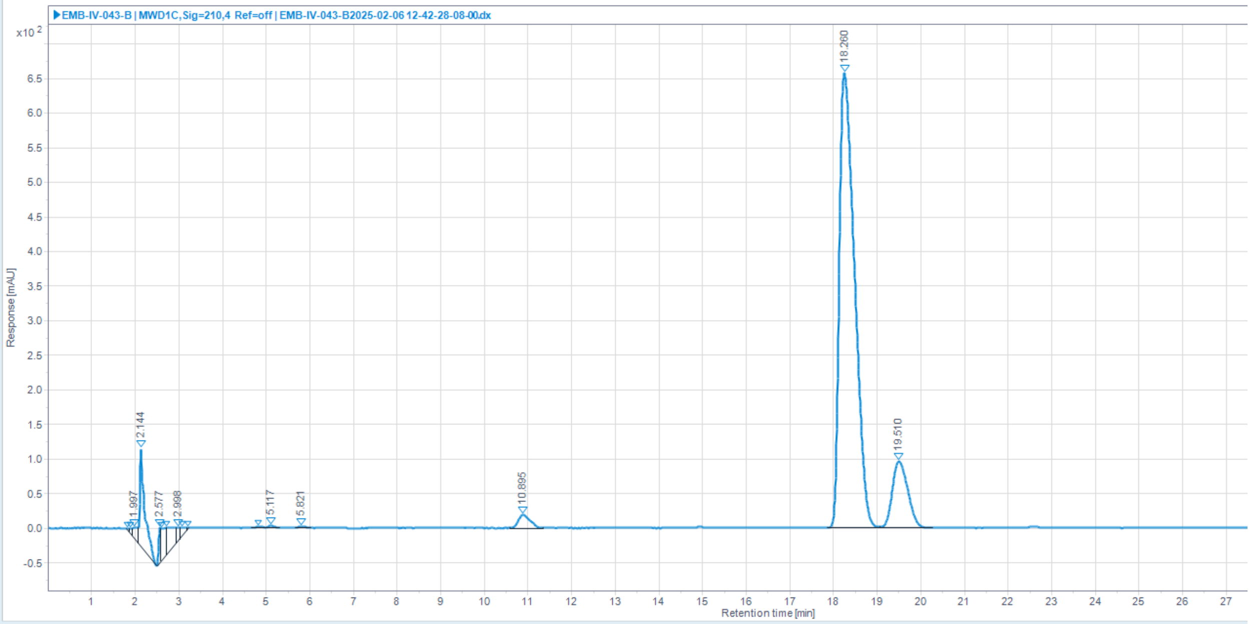

Injection Results

Peaks Summary

#	Name	Signal description	RT (min)	Area (mAU·s)	Area%	Height (mAU)	Height%	Amount	Concentration	Start time (min)	End time (min)
15		MWD1C,Sig=210,4 Ref=off	18.260	15884.355	51.828	657.007	42.15			17.871	19.075
16		MWD1C,Sig=210,4 Ref=off	19.510	2380.709	7.768	96.112	6.17			19.075	20.284

3.2 Evaluation of chiral ligands

Reactions were set up according to General Procedure **A**, modifying the ligand as noted in Table S4 and Table S5. Products were isolated *via* preparative TLC for SFC analysis. Note: only enantioselectivity data was used for modeling. The reaction yields reported were calculated using the mass of product isolated for SFC analysis and were not checked rigorously for purity. We have included these approximate yields to help rationalize lower-than-expected and/or irreproducible experimental *ee* values. Additionally, impurities that did not elute at retention times that obscured integration of product peaks are present in some of the SFC traces reported.

Table S4: Ligand screening results for product **16**.

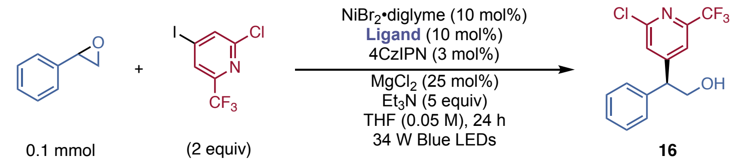

0.1 mmol (2 equiv) **16**

Ligand		Yield 16 (%)	ee (%)	Ligand		Yield 16 (%)	ee (%)
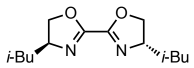 **L11**	run 1	57	45	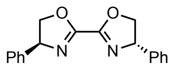 **L15**	run 1	37	39
	run 2	39	46		run 2	26	39
	**average**	**48**	**45**		**average**	**31**	**39**
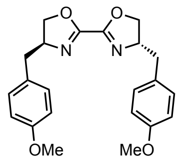 **L16**	run 1	52	47				
	run 2	39	47				
	**average**	**45**	**47**				

Table S5: Ligand screening results for product **23**.

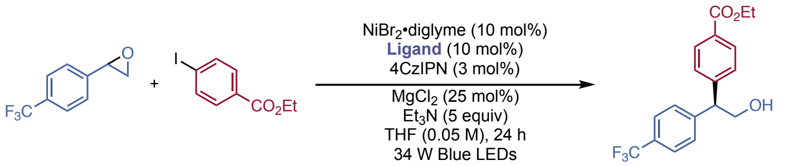

0.1 mmol (2 equiv) **23**

Ligand		Yield 23 (%)	ee (%)	Ligand		Yield 23 (%)	ee (%)
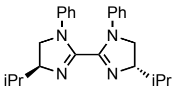	run 1	27	64	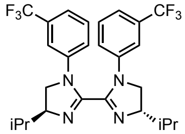	run 1	48	56
	run 2	30	63		run 2	26	57
**L7**	**average**	**28**	**63**	**L8**	**average**	**37**	**56**
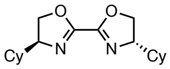	run 1	22	69	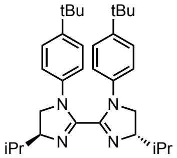	run 1	25	65
	run 2	14	72		run 2	35	63
**L14**	**average**	**18**	**70**	**L26**	**average**	**30**	**64**
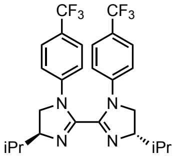	run 1	14	62	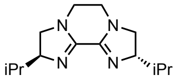	run 1	25	75
	run 2	20	63		run 2	26	37
**L27**	**average**	**17**	**62**	**L28**	**average**	**25**	**56***
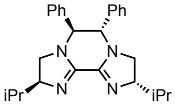	run 1	53	64	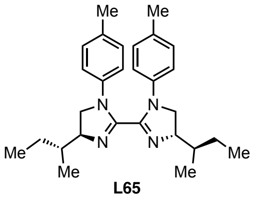	run 1	40	71
	run 2	17	58		run 2	35	72
**L29**	**average**	**35**	**61**	**L65**	**average**	**37**	**71**
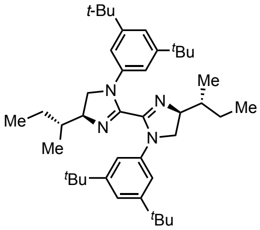	run 1	40	84	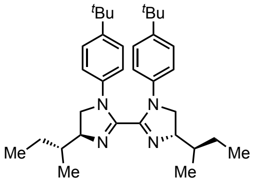	run 1	40	69
	run 2	36	85		run 2	44	70
**L66**	**average**	**38**	**84**	**L144**	**average**	**42**	**69**

*Due to solubility issues, measured *ee* values for the reaction using ligand **L28** was considered unreliable and therefore excluded from the validation data set of the models.

3.3 Additional ligand screening data

Among the ligands included in the "virtual" set, **L65**, **L66**, and **L144** were predicted by the ensemble models with linear or nonlinear parameters to afford the highest $\Delta\Delta G^{\ddagger}$ in the reaction to form product **23** (Table S12) and were tested first due to their availability in our lab. Following the identification of **L66** as a better-performing ligand, we purchased and evaluated three additional ligands (**L73**, **L124**, and **L143**) in the reaction to form product **23** that were also predicted to afford high $\Delta\Delta G^{\ddagger}$ values with an acceptable level of uncertainty. The results shown in Figure S7 indicate that **L66** remains optimal. The large deviation between predicted and measured $\Delta\Delta G^{\ddagger}$ value for **L73**, which afforded product **23** in only 2% *ee*, may be attributed to its inefficient ligation to the Ni-center due to the bulky *t*-butyl substituents. Additionally, we evaluated the performance of **L66** in the reactions yielding products **3** and **16** to verify whether it would afford a similar improvement in enantioselectivity compared to **L6** as it did for product **23** (Figure S8). **L6** and **L66** were found to afford similar *ee* values, suggesting that **L66** is tailored for forming product **23** in higher selectivity.

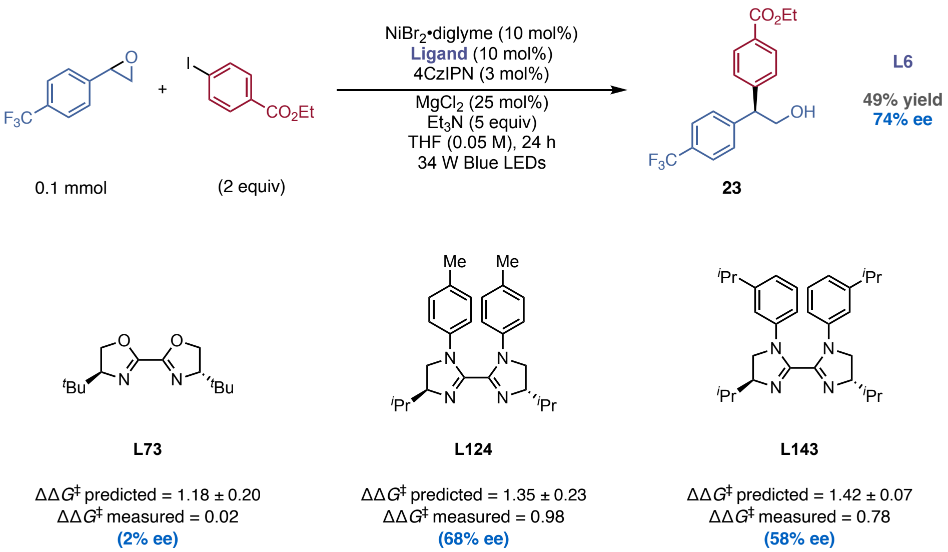

Figure S7. Additional ligands evaluated in the cross-coupling to form product **23**.

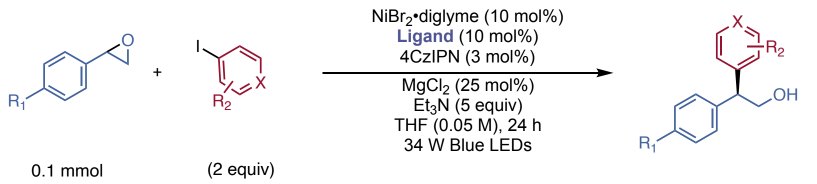

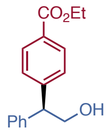

3

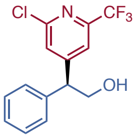

16

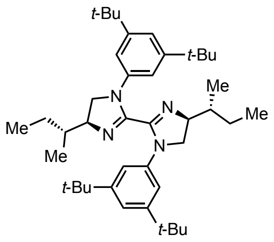

L66

	3	16
**L6**	**70% yield** **91% ee**	**58% yield** **78% ee**
**L66**	**67% yield** **92% ee**	**48% yield** **79% ee**

Figure S8. Evaluation of **L66** in cross-couplings to form products **3** and **16**.

3.3 SFC determination of enantiomeric excess

Product **16**: Daicel ChiralPak IB column, 10% *i*-PrOH, 21 min run, 1.5 mL/min.
L11: (45% *ee*, 46% *ee*)

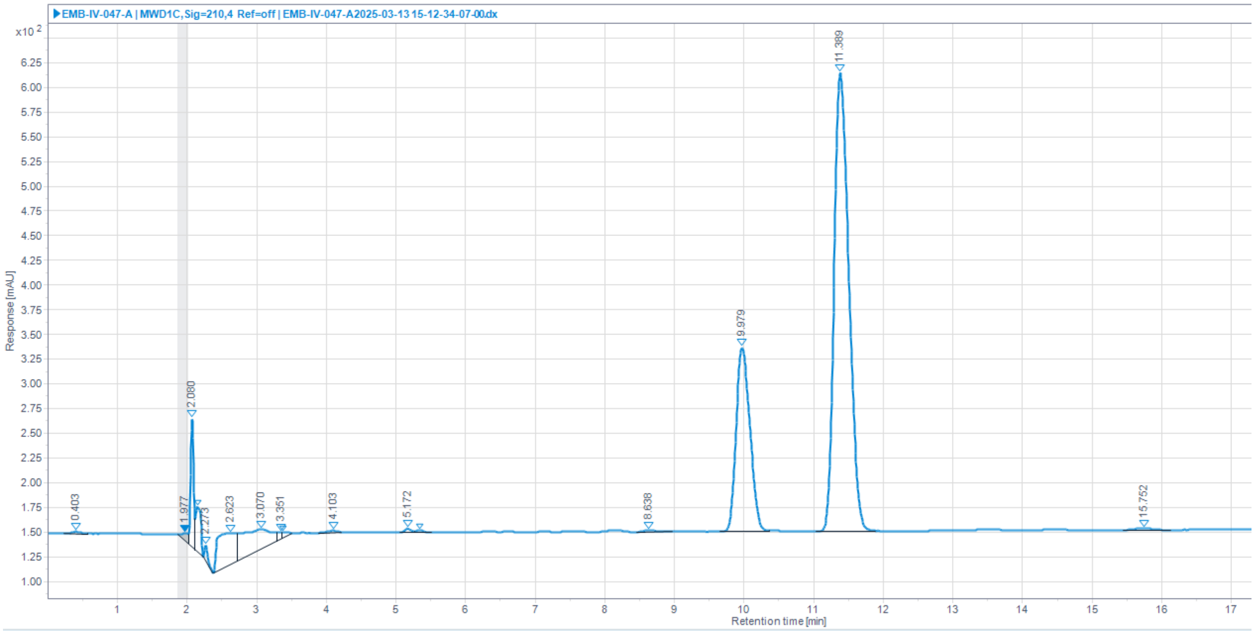

Injection Results

Peaks Summary

#	Name	Signal description	RT (min)	Area (mAU·s)	Area%	Height (mAU)	Height%	Amount	Concentration	Start time (min)	End time (min)
14		MWD1C,Sig=210,4 Ref=off	9.979	2579.644	14.519	186.319	14.60			9.657	10.367
15		MWD1C,Sig=210,4 Ref=off	11.389	6827.330	38.426	464.168	36.36			11.046	11.901

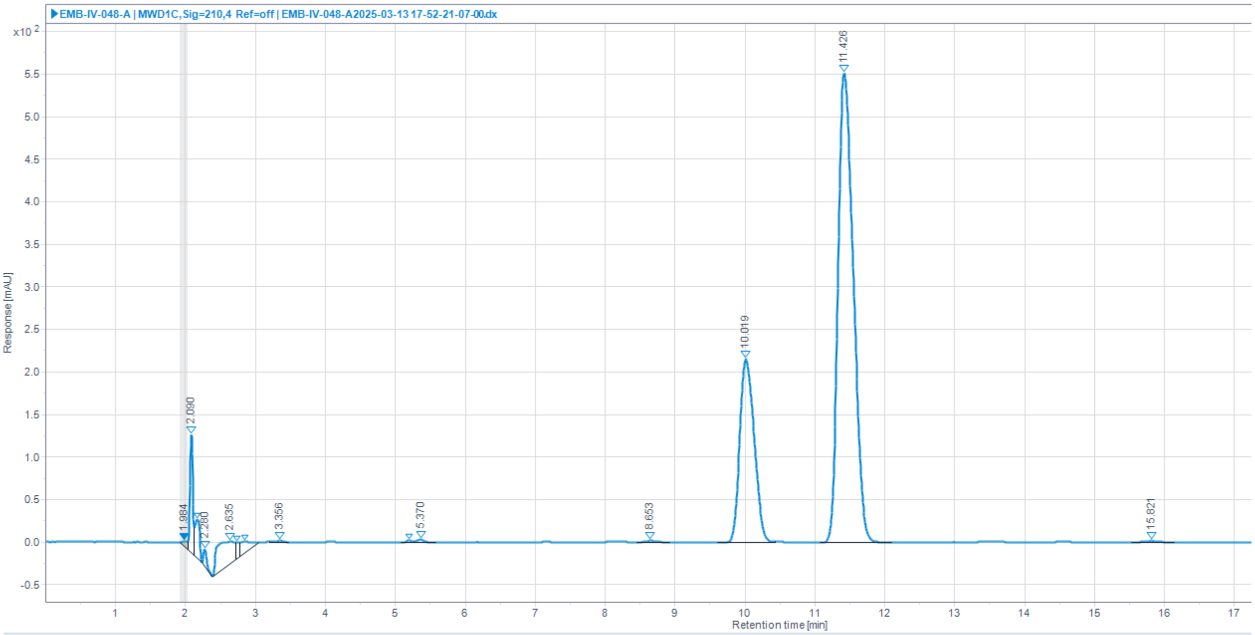

Injection Results

Peaks Summary

#	Name	Signal description	RT (min)	Area (mAU·s)	Area%	Height (mAU)	Height%	Amount	Concentration	Start time (min)	End time (min)
12		MWD1C,Sig=210,4 Ref=off	10.019	3107.716	16.800	215.461	16.76			9.614	10.445
13		MWD1C,Sig=210,4 Ref=off	11.426	8404.559	45.433	551.366	42.90			11.082	12.109

L15: (39% *ee*, 39% *ee*)

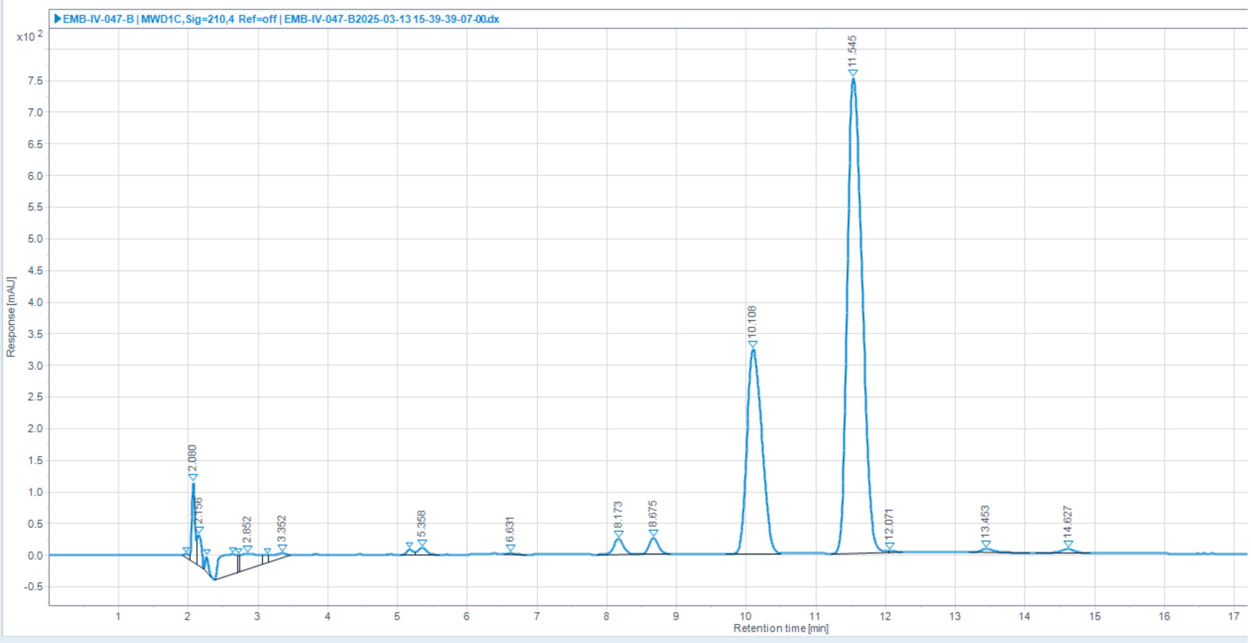

Injection Results

Peaks | Summary

#	Name	Signal description	RT (min)	Area (mAU·s)	Area%	Height (mAU)	Height%	Amount	Concentration	Start time (min)	End time (min)
15		MWD1C,Sig=210,4 Ref=off	10.108	4817.478	19.699	324.060	17.24			9.717	10.506
16		MWD1C,Sig=210,4 Ref=off	11.545	11097.550	45.379	750.977	39.96			11.224	12.049

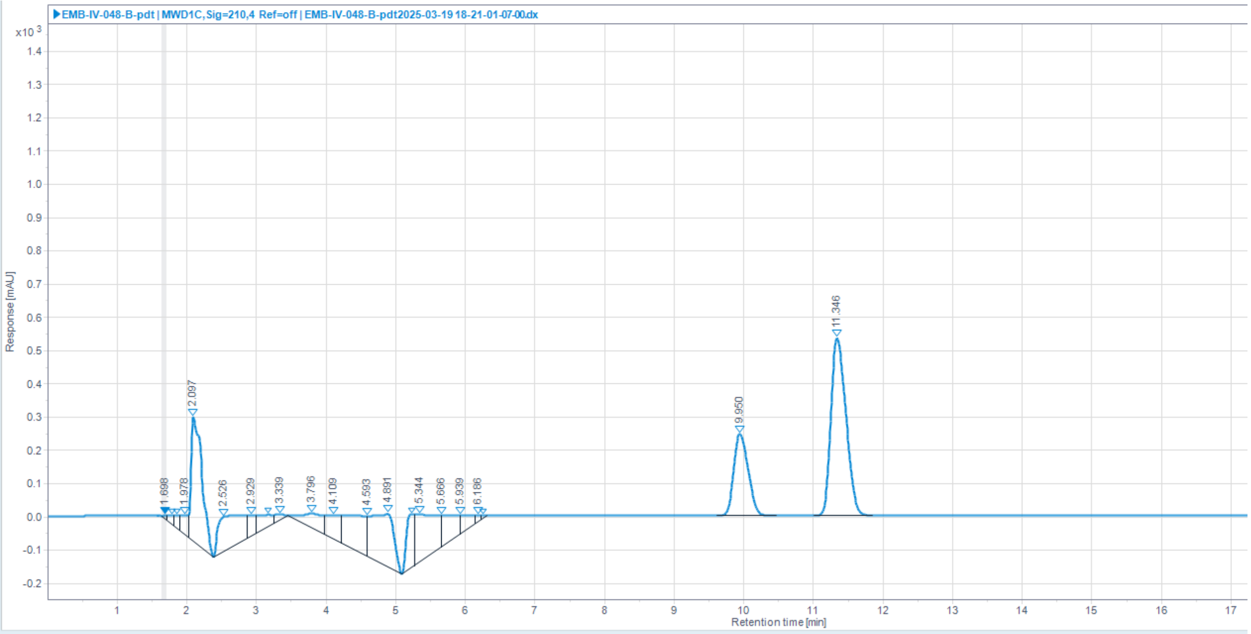

Injection Results

Peaks | Summary

#	Name	Signal description	RT (min)	Area (mAU·s)	Area%	Height (mAU)	Height%	Amount	Concentration	Start time (min)	End time (min)
20		MWD1C,Sig=210,4 Ref=off	9.950	3512.502	3.507	245.969	5.34			9.627	10.468
21		MWD1C,Sig=210,4 Ref=off	11.346	8045.282	8.032	532.487	11.57			11.013	11.849

L16: (47% *ee*, 47% *ee*)

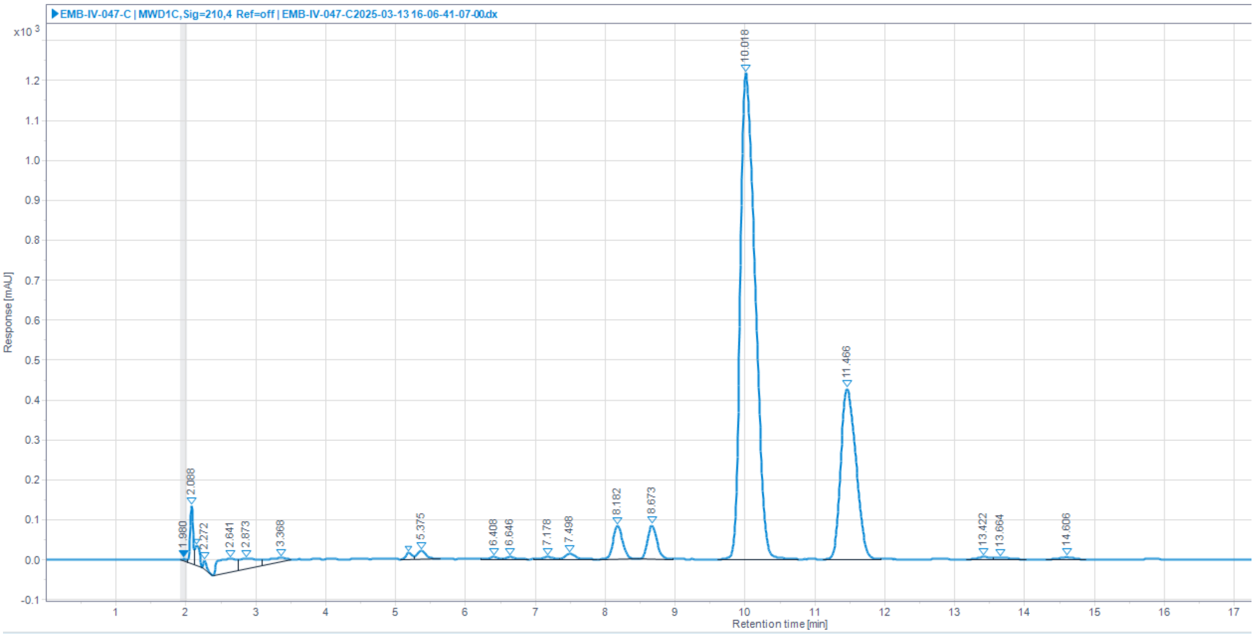

Injection Results

Peaks | Summary

#	Name	Signal description	RT (min)	Area (mAU·s)	Area%	Height (mAU)	Height%	Amount	Concentration	Start time (min)	End time (min)
16		MWD1C,Sig=210,4 Ref=off	10.018	17934.425	52.479	1217.220	48.14			9.617	10.775
17		MWD1C,Sig=210,4 Ref=off	11.466	6486.608	18.981	426.114	16.85			11.129	11.956

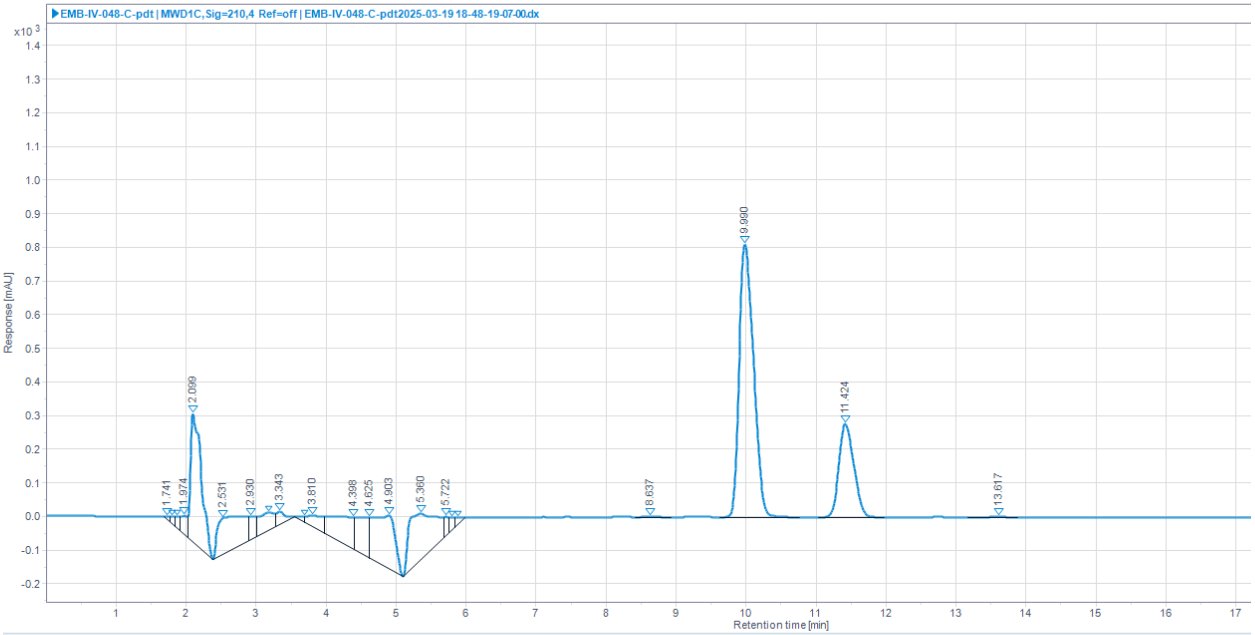

Injection Results

Peaks | Summary

#	Name	Signal description	RT (min)	Area (mAU·s)	Area%	Height (mAU)	Height%	Amount	Concentration	Start time (min)	End time (min)
20		MWD1C,Sig=210,4 Ref=off	9.990	11599.115	12.661	812.083	20.13			9.631	10.765
21		MWD1C,Sig=210,4 Ref=off	11.424	4145.306	4.525	276.987	6.86			11.040	11.984

Product **23**: Daicel ChiralPak IB column, 7% *i*-PrOH, 31 min run, 1.5 mL/min.

L7: (64% *ee*, 63% *ee*)

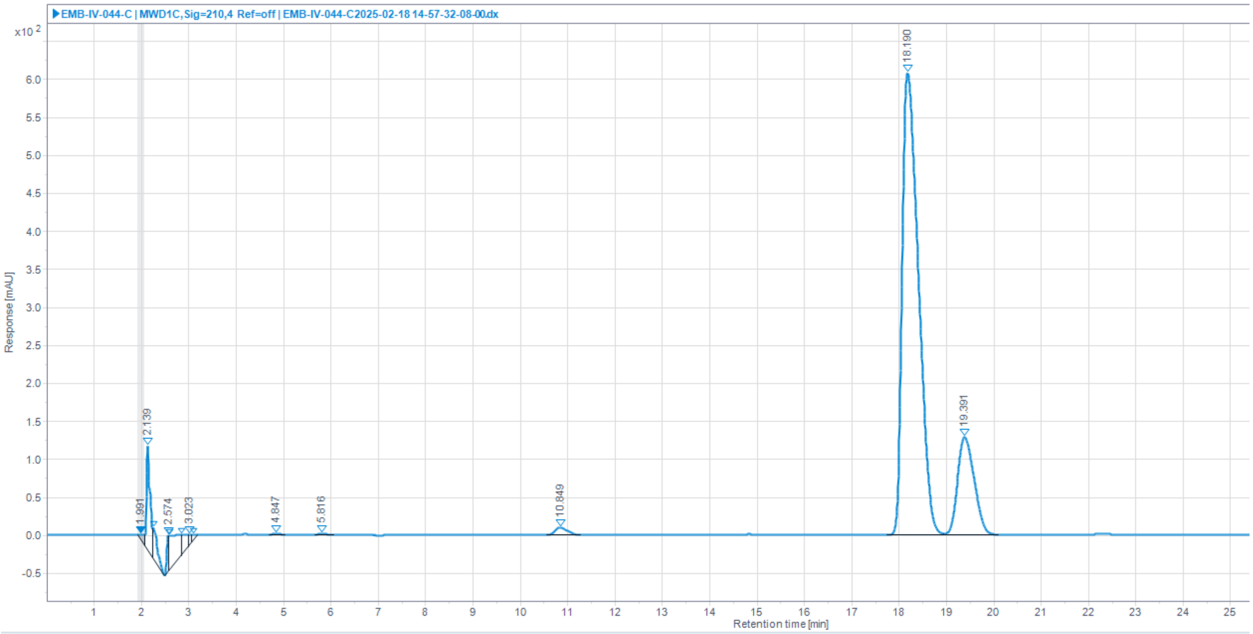

Injection Results

Peaks | Summary

#	Name	Signal description	RT (min)	Area (mAU·s)	Area%	Height (mAU)	Height%	Amount	Concentration	Start time (min)	End time (min)
12		MWD1C,Sig=210,4 Ref=off	18.190	14471.575	50.140	607.497	32.99			17.746	18.955
13		MWD1C,Sig=210,4 Ref=off	19.391	3139.563	10.878	128.251	6.96			18.955	20.110

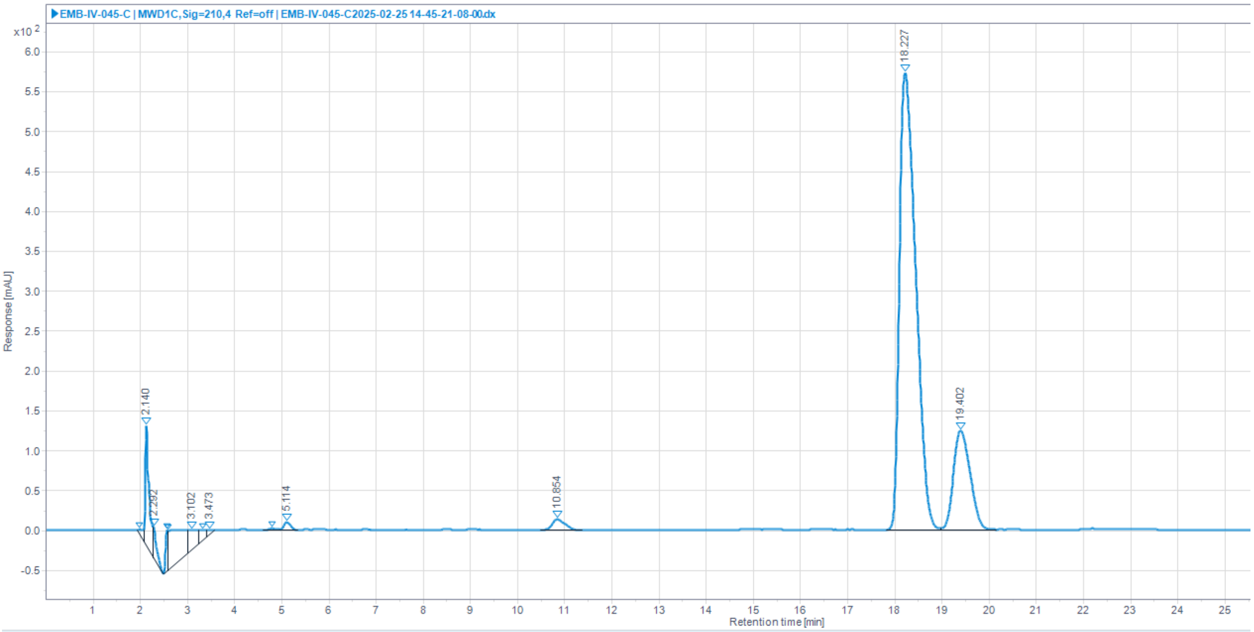

Injection Results

Peaks | Summary

#	Name	Signal description	RT (min)	Area (mAU·s)	Area%	Height (mAU)	Height%	Amount	Concentration	Start time (min)	End time (min)
12		MWD1C,Sig=210,4 Ref=off	18.227	13692.284	48.555	572.621	36.70			17.840	18.980
13		MWD1C,Sig=210,4 Ref=off	19.402	3058.781	10.847	124.258	7.96			18.980	20.172

L8: (56% *ee*, 57% *ee*)

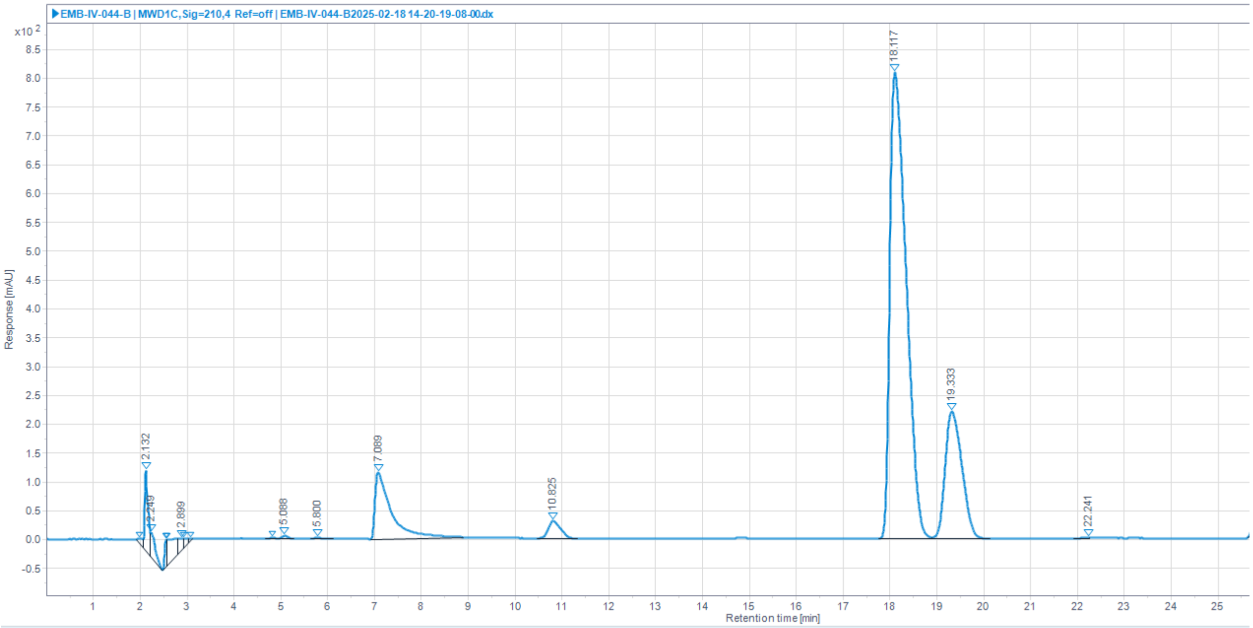

Injection Results

Peaks Summary

#	Name	Signal description	RT (min)	Area (mAU·s)	Area%	Height (mAU)	Height%	Amount	Concentration	Start time (min)	End time (min)
14		MWD1C,Sig=210,4 Ref=off	18.117	19573.707	51.382	808.662	41.65			17.762	18.908
15		MWD1C,Sig=210,4 Ref=off	19.333	5490.672	14.413	220.240	11.34			18.908	20.138

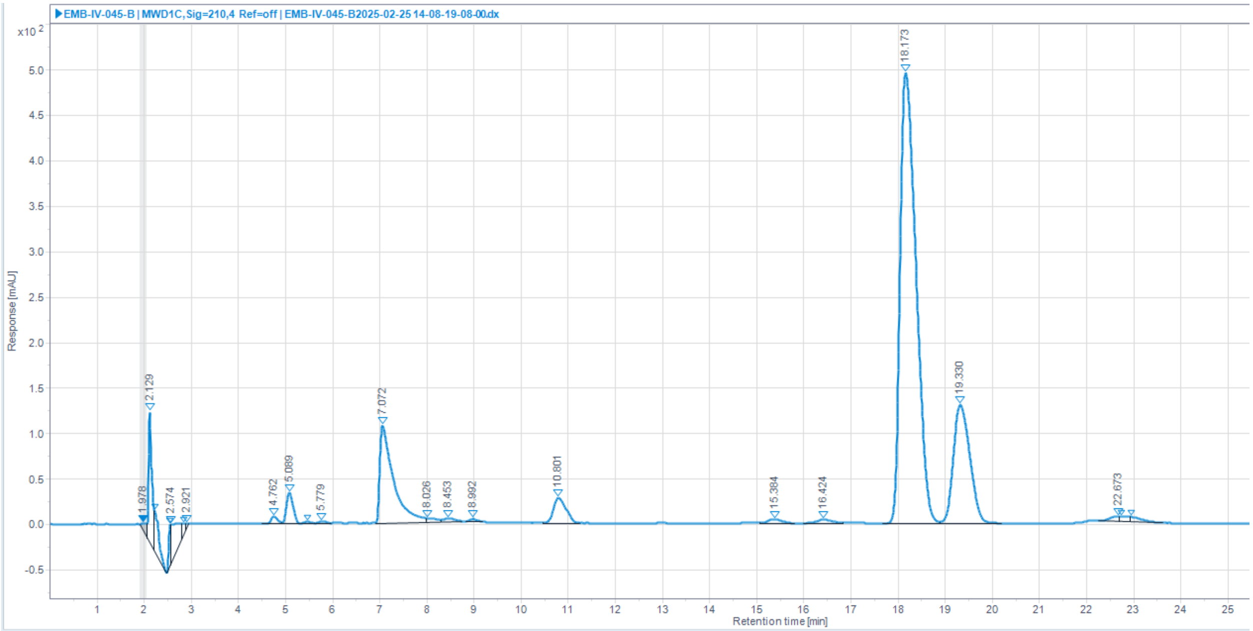

Injection Results

Peaks Summary

#	Name	Signal description	RT (min)	Area (mAU·s)	Area%	Height (mAU)	Height%	Amount	Concentration	Start time (min)	End time (min)
19		MWD1C,Sig=210,4 Ref=off	18.173	11643.519	40.063	495.406	27.23			17.689	18.896
20		MWD1C,Sig=210,4 Ref=off	19.330	3174.323	10.922	130.029	7.15			18.896	20.198

L14: (69% *ee*, 72% *ee*)

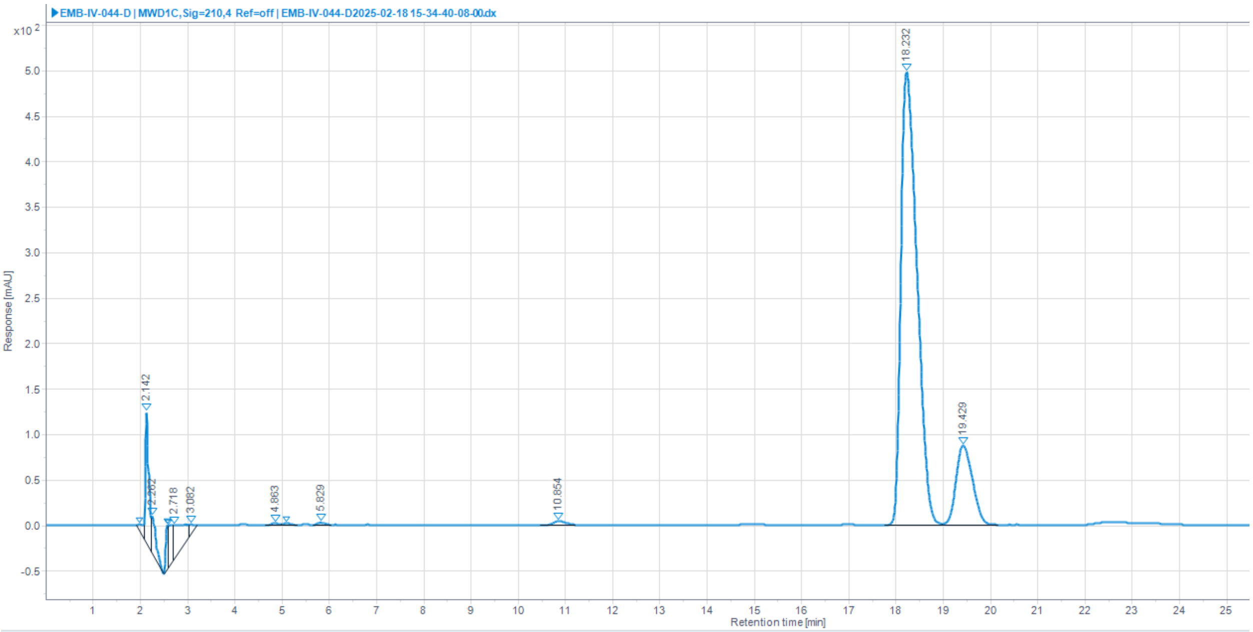

Injection Results

Peaks | Summary

#	Name	Signal description	RT (min)	Area (mAU·s)	Area%	Height (mAU)	Height%	Amount	Concentration	Start time (min)	End time (min)
12		MWD1C,Sig=210,4 Ref=off	18.232	11748.716	53.873	497.788	34.62			17.771	18.997
13		MWD1C,Sig=210,4 Ref=off	19.429	2113.092	9.689	87.058	6.06			18.997	20.162

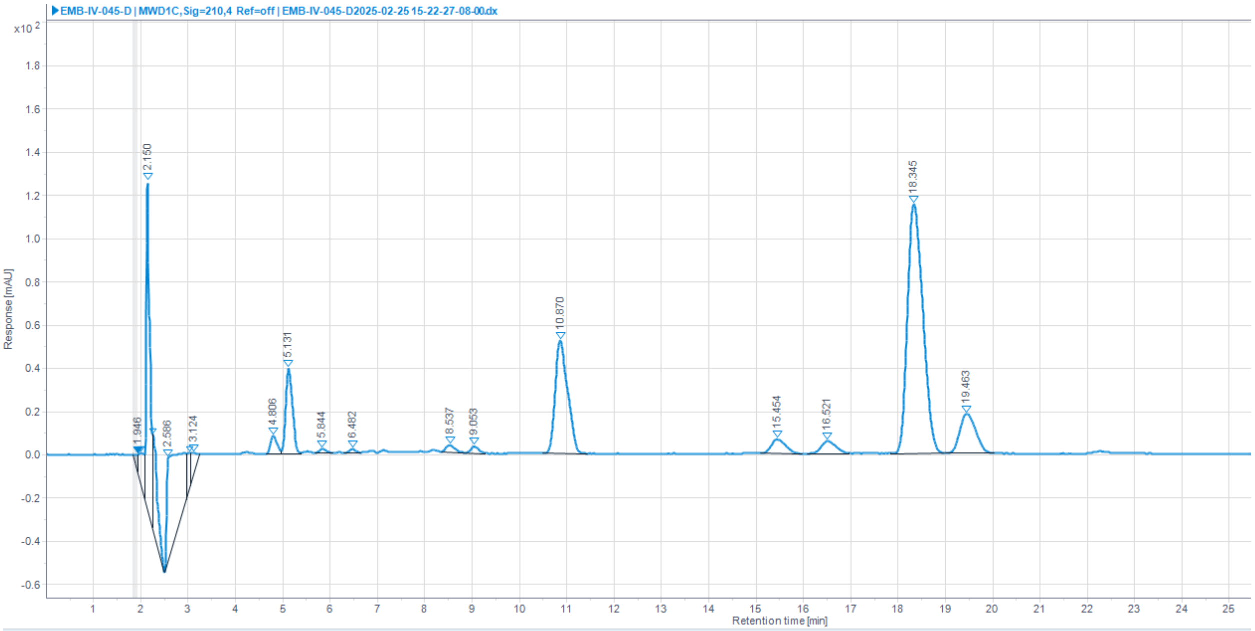

Injection Results

Peaks | Summary

#	Name	Signal description	RT (min)	Area (mAU·s)	Area%	Height (mAU)	Height%	Amount	Concentration	Start time (min)	End time (min)
17		MWD1C,Sig=210,4 Ref=off	18.345	2621.751	15.943	115.246	9.80			17.865	18.999
18		MWD1C,Sig=210,4 Ref=off	19.463	426.929	2.596	18.312	1.56			19.006	20.040

L26: (65% *ee*, 63% *ee*)

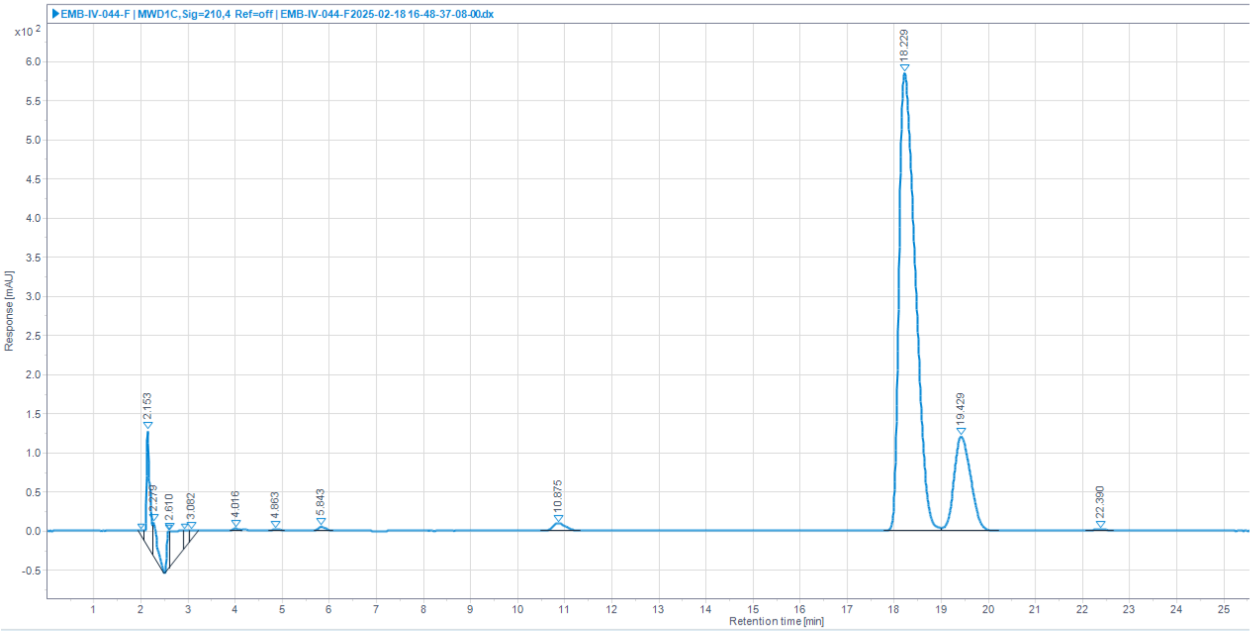

Injection Results

Peaks | Summary

#	Name	Signal description	RT (min)	Area (mAU·s)	Area%	Height (mAU)	Height%	Amount	Concentration	Start time (min)	End time (min)
12		MWD1C,Sig=210,4 Ref=off	18.229	13998.907	49.052	584.990	39.77			17.783	19.011
13		MWD1C,Sig=210,4 Ref=off	19.429	2958.930	10.368	120.328	8.18			19.011	20.227

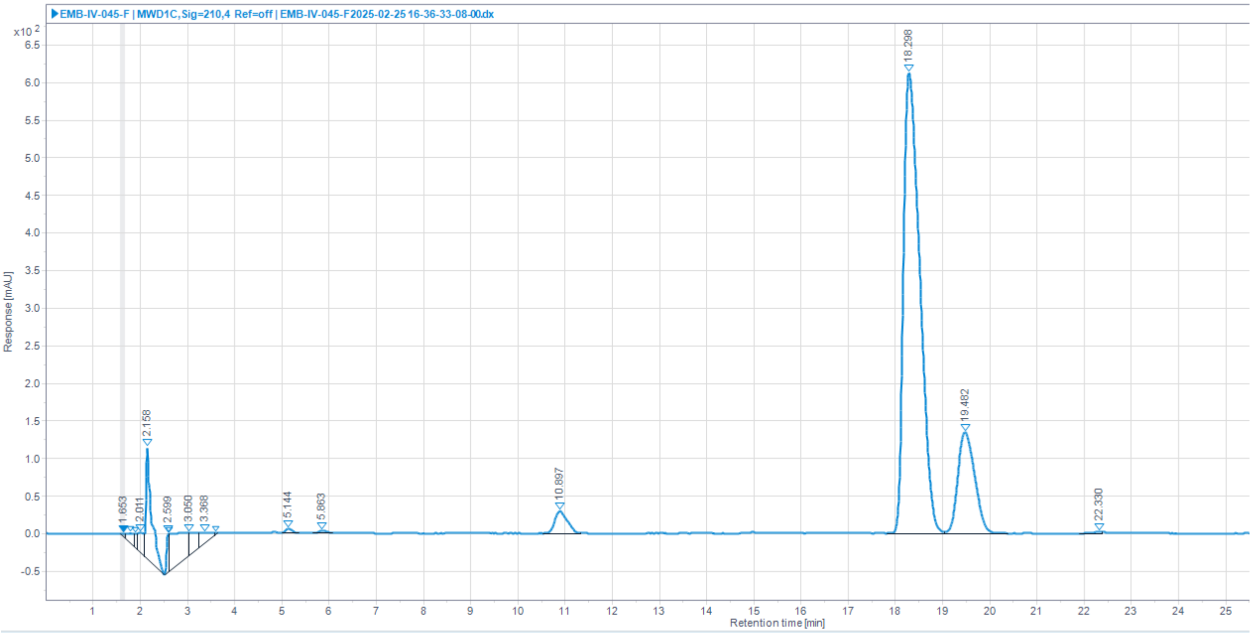

Injection Results

Peaks | Summary

#	Name	Signal description	RT (min)	Area (mAU·s)	Area%	Height (mAU)	Height%	Amount	Concentration	Start time (min)	End time (min)
14		MWD1C,Sig=210,4 Ref=off	18.298	14728.081	47.580	612.119	34.39			17.823	19.054
15		MWD1C,Sig=210,4 Ref=off	19.482	3339.753	10.789	134.554	7.56			19.054	20.396

L27: (62% *ee*, 63% *ee*)

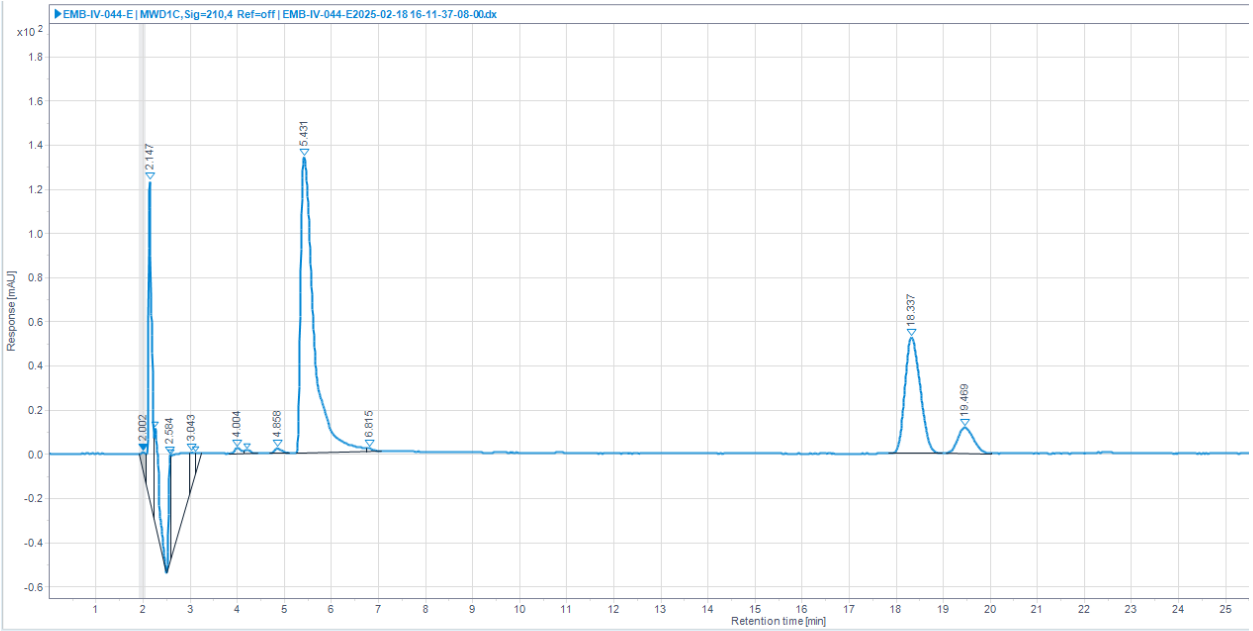

Injection Results

Peaks | Summary

#	Name	Signal description	RT (min)	Area (mAU·s)	Area%	Height (mAU)	Height%	Amount	Concentration	Start time (min)	End time (min)
13		MWD1C,Sig=210,4 Ref=off	18.337	1187.038	5.233	52.525	4.69			17.856	18.980
14		MWD1C,Sig=210,4 Ref=off	19.469	276.295	1.218	11.698	1.04			19.077	20.029

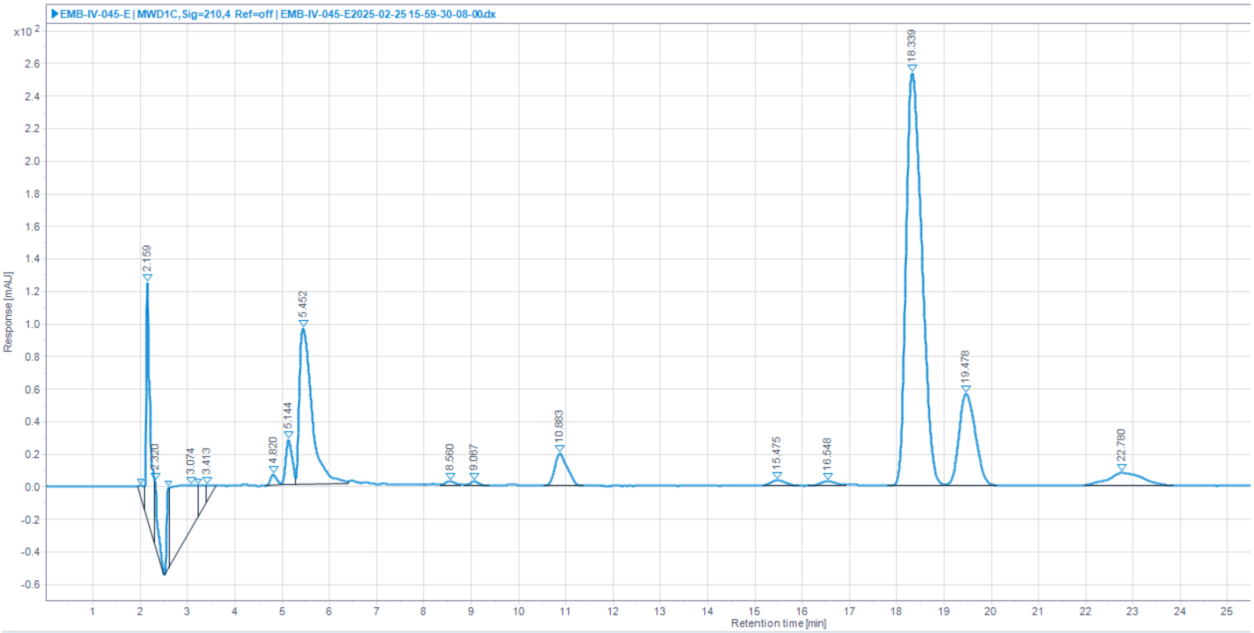

Injection Results

Peaks | Summary

#	Name	Signal description	RT (min)	Area (mAU·s)	Area%	Height (mAU)	Height%	Amount	Concentration	Start time (min)	End time (min)
16		MWD1C,Sig=210,4 Ref=off	18.339	5889.627	26.277	253.297	16.04			17.809	19.052
17		MWD1C,Sig=210,4 Ref=off	19.478	1350.679	6.026	56.217	3.56			19.052	20.120

L28: (75% *ee*, 37% *ee*)

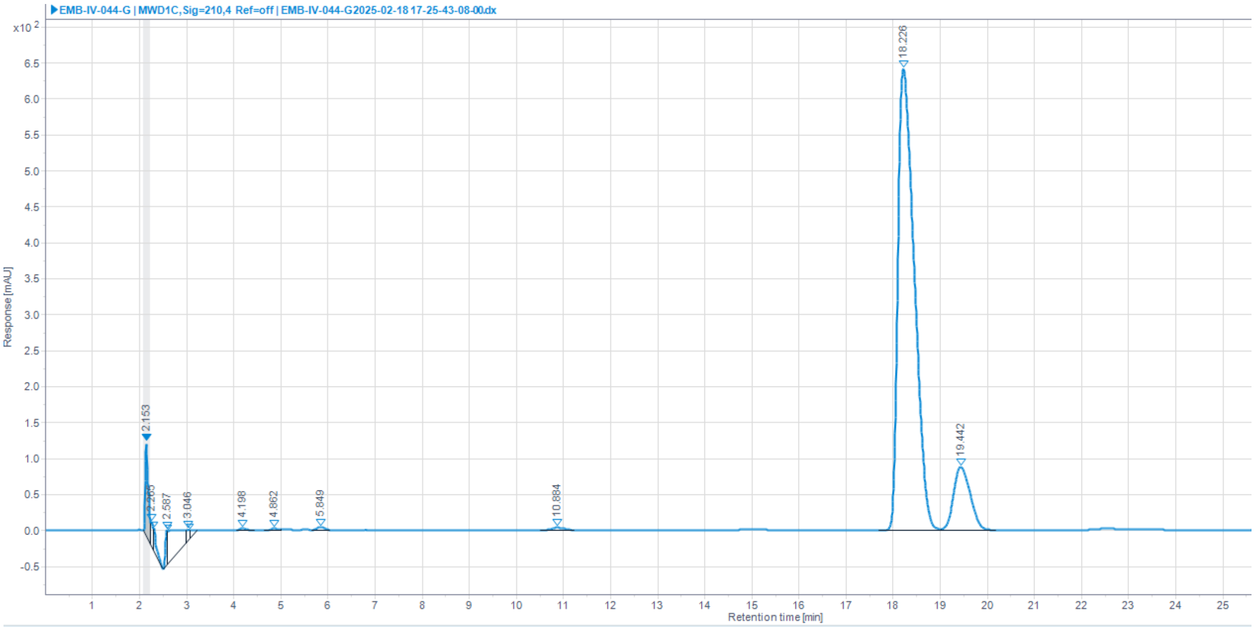

Injection Results

Peaks | Summary

#	Name	Signal description	RT (min)	Area (mAU·s)	Area%	Height (mAU)	Height%	Amount	Concentration	Start time (min)	End time (min)
12		MWD1C,Sig=210,4 Ref=off	18.226	15467.583	57.835	641.429	39.57			17.713	19.024
13		MWD1C,Sig=210,4 Ref=off	19.442	2163.672	8.090	87.765	5.41			19.024	20.194

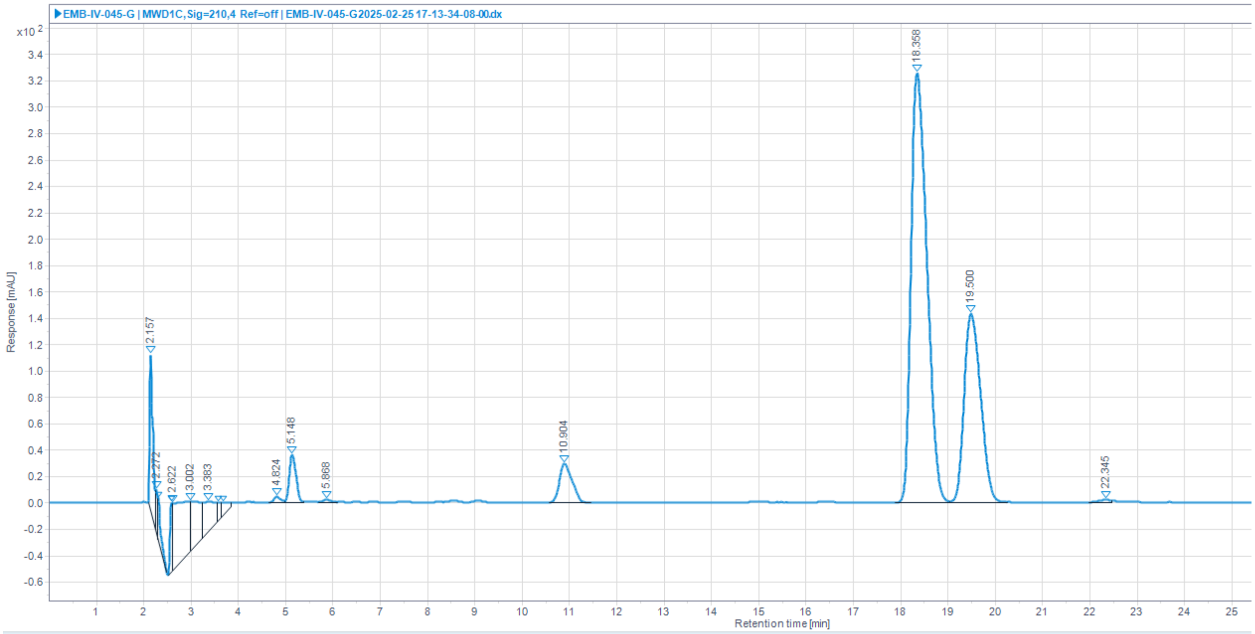

Injection Results

Peaks | Summary

#	Name	Signal description	RT (min)	Area (mAU·s)	Area%	Height (mAU)	Height%	Amount	Concentration	Start time (min)	End time (min)
14		MWD1C,Sig=210,4 Ref=off	18.358	7602.181	31.913	325.772	20.57			17.903	19.059
15		MWD1C,Sig=210,4 Ref=off	19.500	3491.223	14.656	143.173	9.04			19.059	20.266

L29: (64% *ee*, 58% *ee*)

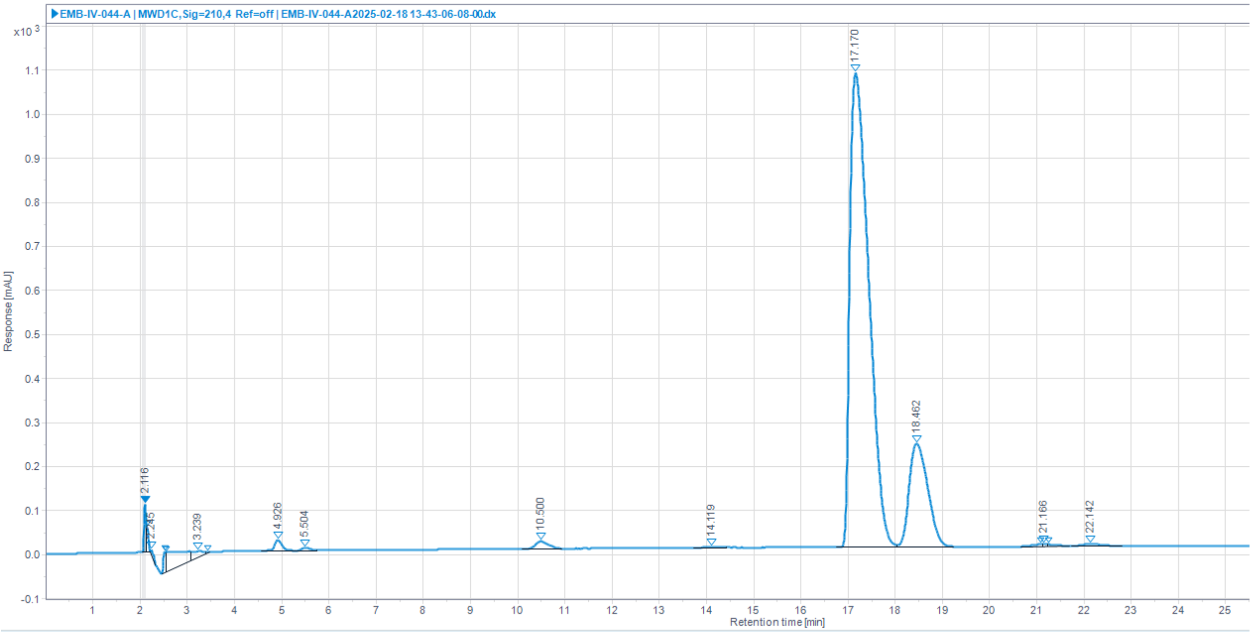

Injection Results

Peaks Summary

#	Name	Signal description	RT (min)	Area (mAU·s)	Area%	Height (mAU)	Height%	Amount	Concentration	Start time (min)	End time (min)
12		MWD1C,Sig=210,4 Ref=off	17.170	29767.674	63.480	1077.431	48.46			16.767	18.047
13		MWD1C,Sig=210,4 Ref=off	18.462	6447.796	13.750	234.481	10.55			18.047	19.245

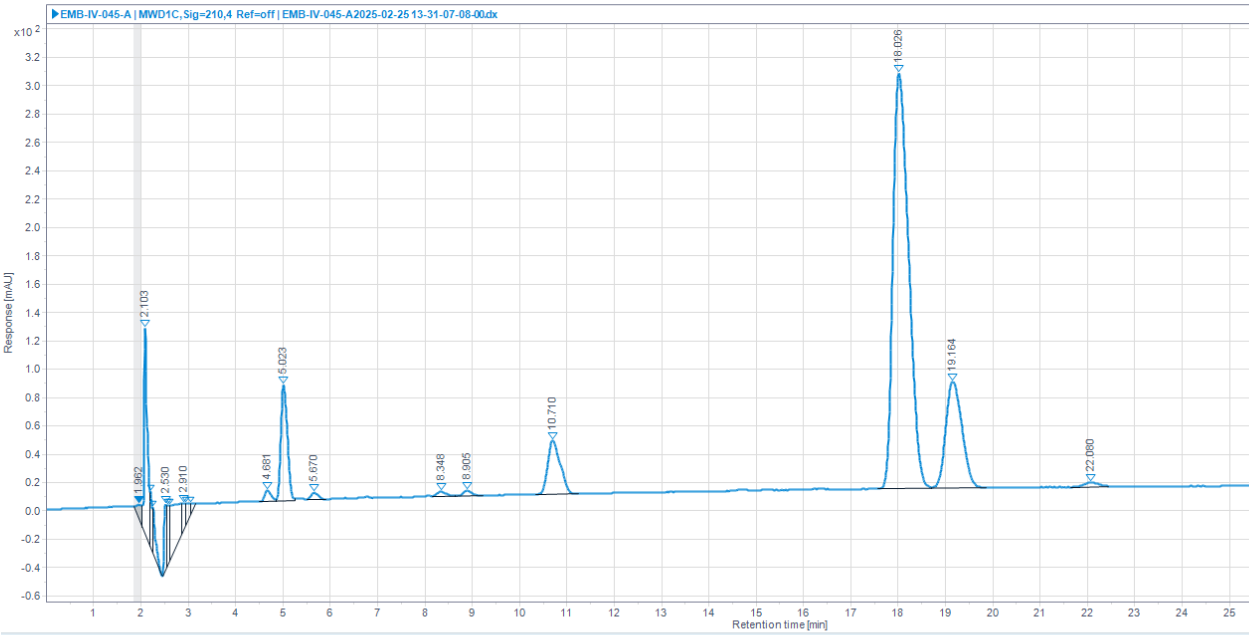

Injection Results

Peaks Summary

#	Name	Signal description	RT (min)	Area (mAU·s)	Area%	Height (mAU)	Height%	Amount	Concentration	Start time (min)	End time (min)
17		MWD1C,Sig=210,4 Ref=off	18.026	6753.290	33.307	293.012	19.90			17.590	18.716
18		MWD1C,Sig=210,4 Ref=off	19.164	1801.812	8.886	75.164	5.10			18.716	19.870

Note: Due to mechanical issues with our SFC system, we were unable to analyze reactions using **L65, L66, L144** with the Daicel ChrialPak IB column. Instead, enantiomeric excess for these reactions was determined using the following SFC method: Daicel ChiralPak IG column, 10% *i*-PrOH, 21 min run, 1.5 mL/min. To confirm the retention times for enantiomers using this method, the SFC sample for **L29** run 1 was analyzed using the new method (shown below). The *ee* for this reaction was calculated to be 64% regardless of the column used for analysis.

L29: (64% *ee* – IG column)

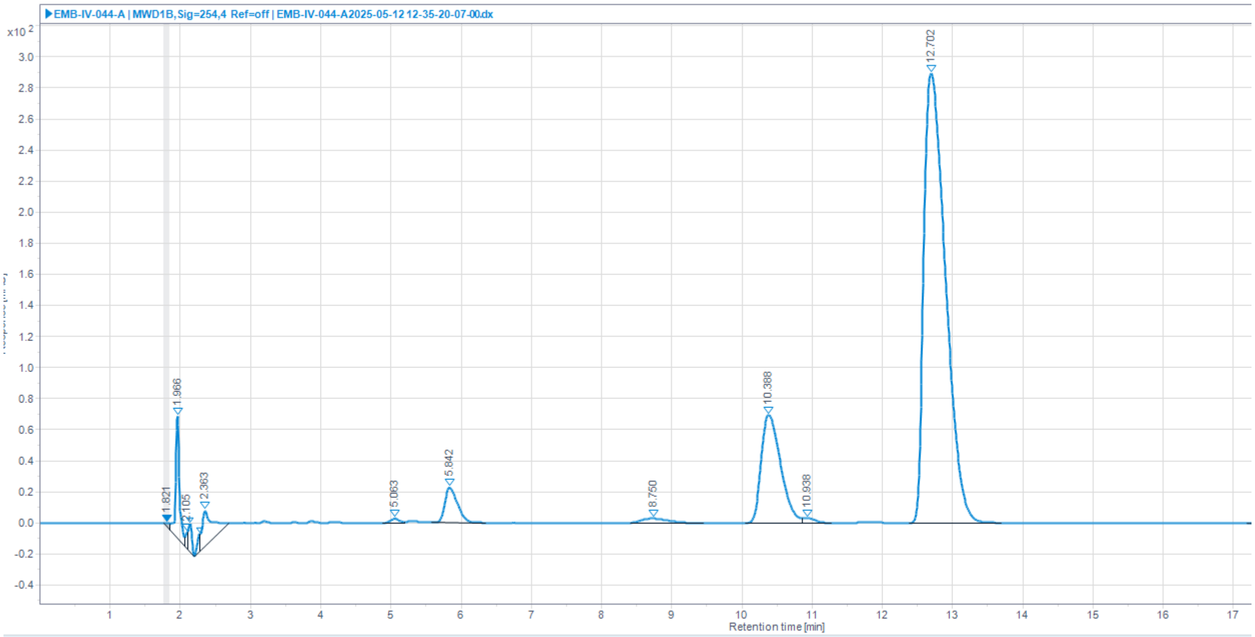

Injection Results

Peaks Summary

#	Name	Signal description	RT (min)	Area (mAU·s)	Area%	Height (mAU)	Height%	Amount	Concentration	Start time (min)	End time (min)
11		MWD1B,Sig=254,4 Ref=off	10.938	40.452	0.388	3.314	0.46			10.861	11.274
12		MWD1B,Sig=254,4 Ref=off	12.702	6209.766	59.556	289.397	40.17			12.394	13.699

L65: (71% *ee*, 72% *ee*)

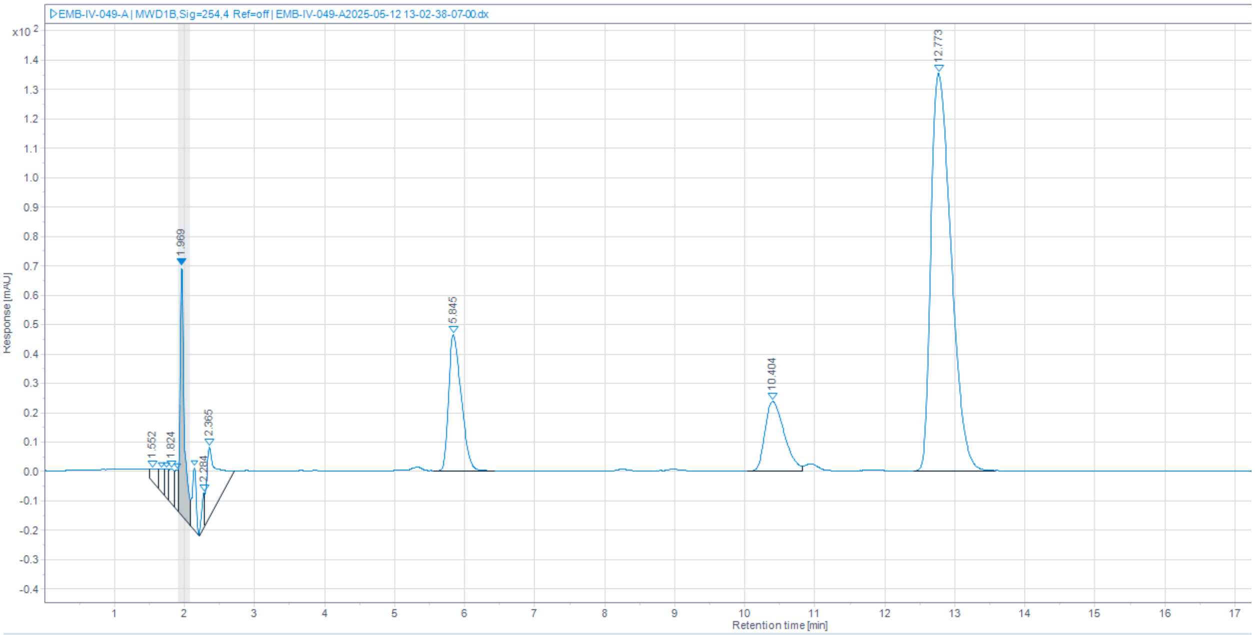

Injection Results

Peaks | Summary

#	Name	Signal description	RT (min)	Area (mAU·s)	Area%	Height (mAU)	Height%	Amount	Concentration	Start time (min)	End time (min)
11		MWD1B,Sig=254,4 Ref=off	10.404	452.457	7.027	23.620	4.07			10.046	10.829
12		MWD1B,Sig=254,4 Ref=off	12.773	2704.745	42.009	135.277	23.32			12.419	13.572

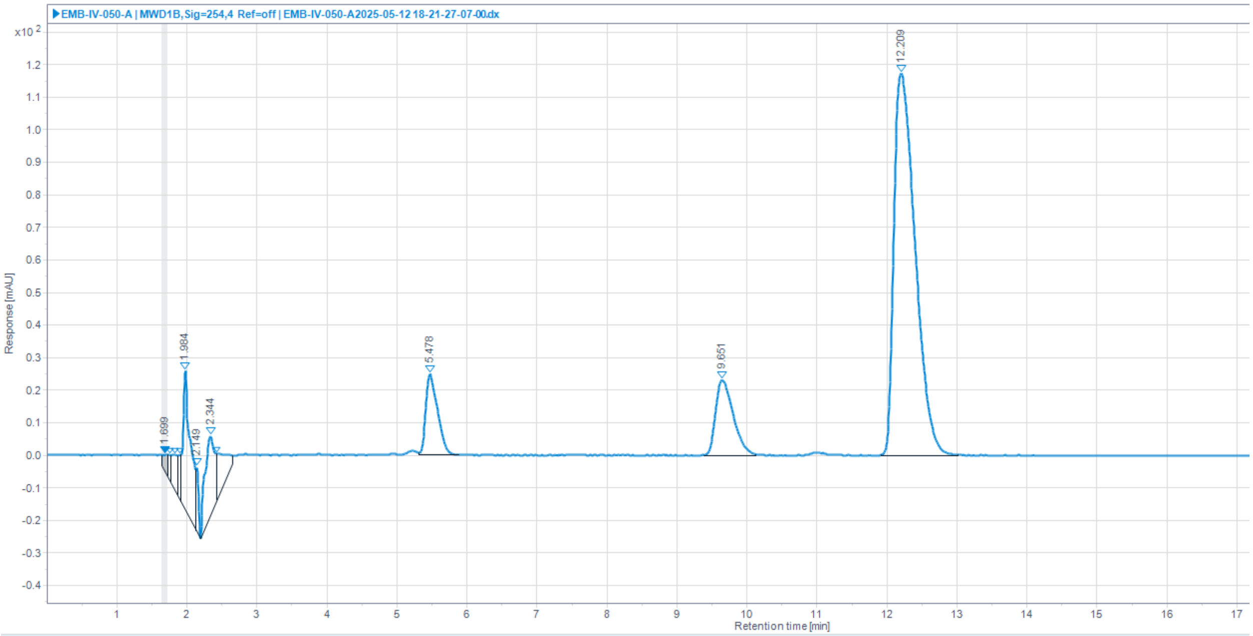

Injection Results

Peaks | Summary

#	Name	Signal description	RT (min)	Area (mAU·s)	Area%	Height (mAU)	Height%	Amount	Concentration	Start time (min)	End time (min)
10		MWD1B,Sig=254,4 Ref=off	9.651	407.886	6.801	23.110	5.46			9.396	10.139
11		MWD1B,Sig=254,4 Ref=off	12.209	2477.382	41.310	117.448	27.75			11.931	13.026

L66: (84% *ee*, 85% *ee*)

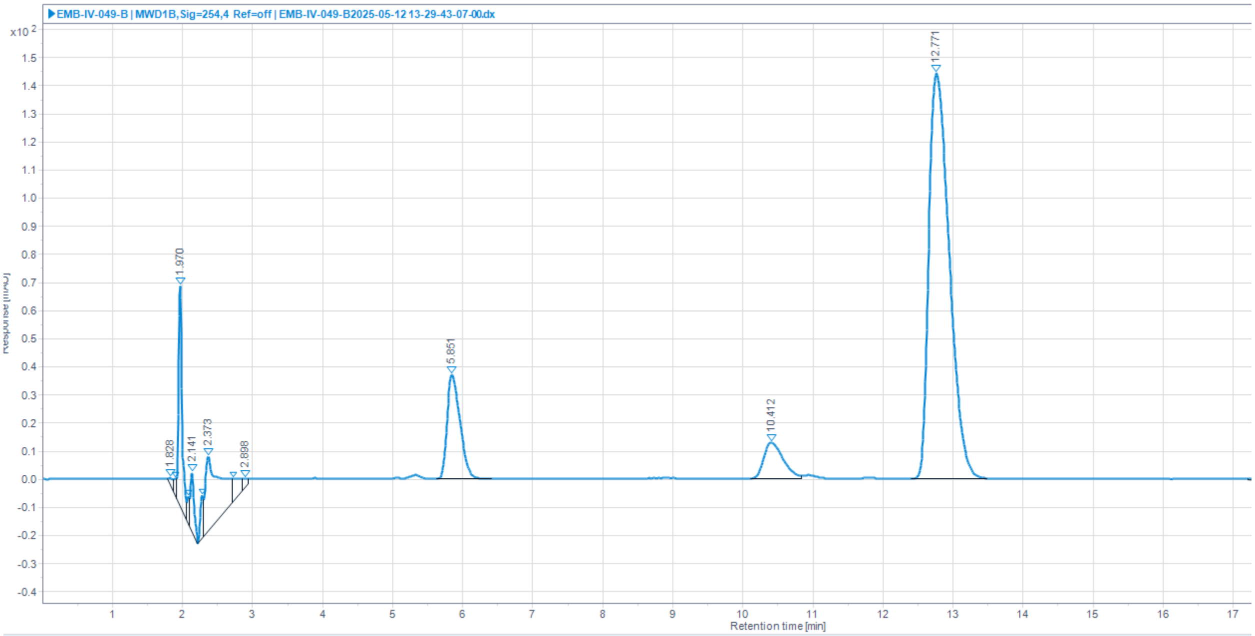

Injection Results

Peaks Summary

#	Name	Signal description	RT (min)	Area (mAU·s)	Area%	Height (mAU)	Height%	Amount	Concentration	Start time (min)	End time (min)
11		MWD1B,Sig=254,4 Ref=off	10.412	249.225	3.311	12.817	2.10			10.114	10.843
12		MWD1B,Sig=254,4 Ref=off	12.771	2935.579	39.003	144.271	23.66			12.405	13.485

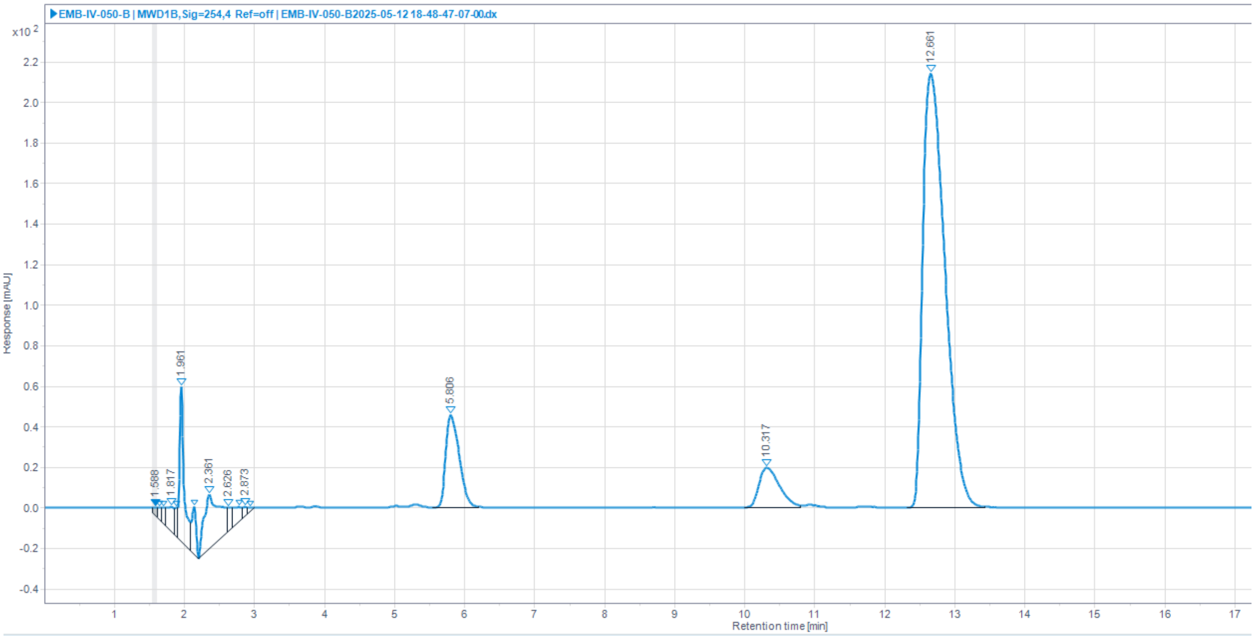

Injection Results

Peaks Summary

#	Name	Signal description	RT (min)	Area (mAU·s)	Area%	Height (mAU)	Height%	Amount	Concentration	Start time (min)	End time (min)
14		MWD1B,Sig=254,4 Ref=off	10.317	401.925	4.652	19.564	3.26			10.008	10.803
15		MWD1B,Sig=254,4 Ref=off	12.661	4724.012	54.672	214.177	35.73			12.327	13.434

L144: (69% *ee*, 70% *ee*)

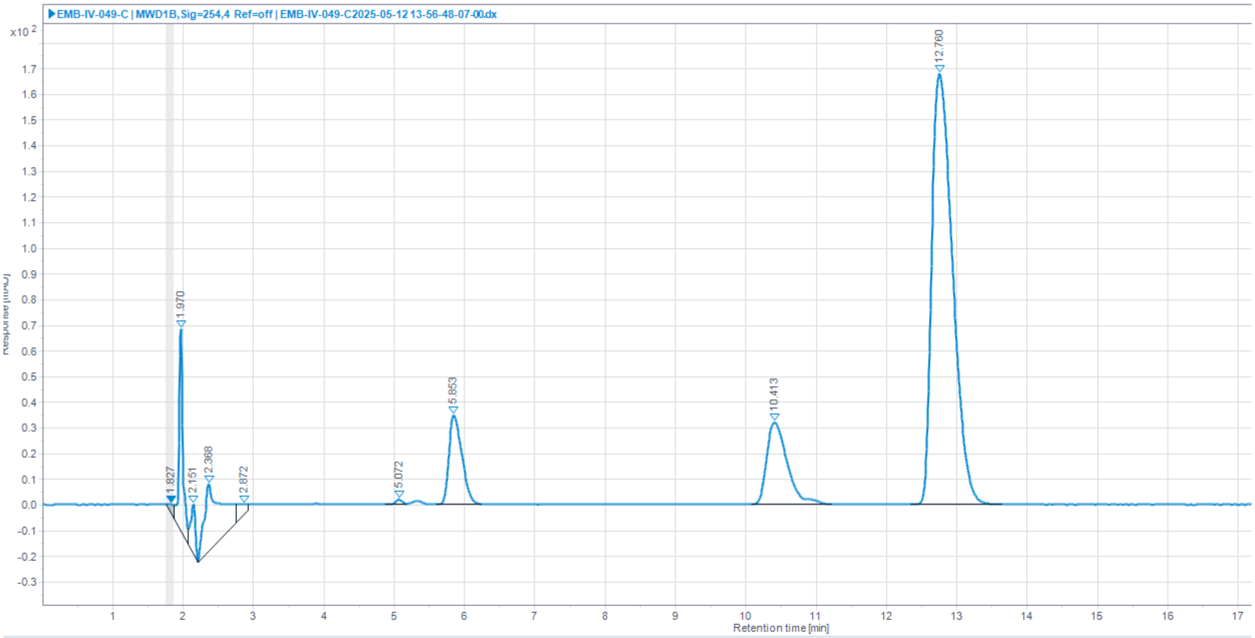

Injection Results

Peaks Summary

#	Name	Signal description	RT (min)	Area (mAU·s)	Area%	Height (mAU)	Height%	Amount	Concentration	Start time (min)	End time (min)
8		MWD1B,Sig=254,4 Ref=off	10.413	652.129	9.100	31.963	6.47			10.089	11.231
9		MWD1B,Sig=254,4 Ref=off	12.760	3454.667	48.207	167.901	33.98			12.351	13.645

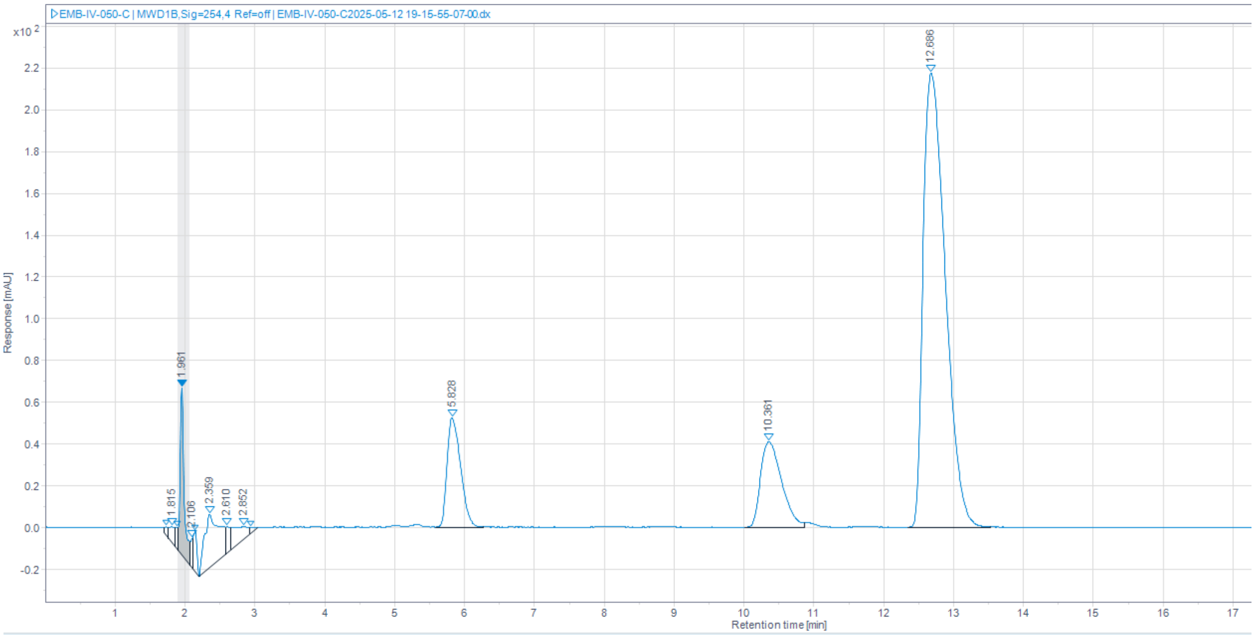

Injection Results

Peaks Summary

#	Name	Signal description	RT (min)	Area (mAU·s)	Area%	Height (mAU)	Height%	Amount	Concentration	Start time (min)	End time (min)
12		MWD1B,Sig=254,4 Ref=off	10.361	862.115	9.331	40.903	6.51			10.013	10.876
13		MWD1B,Sig=254,4 Ref=off	12.686	4792.802	51.875	217.262	34.60			12.358	13.536

3.4 MLR with TSRE-only features

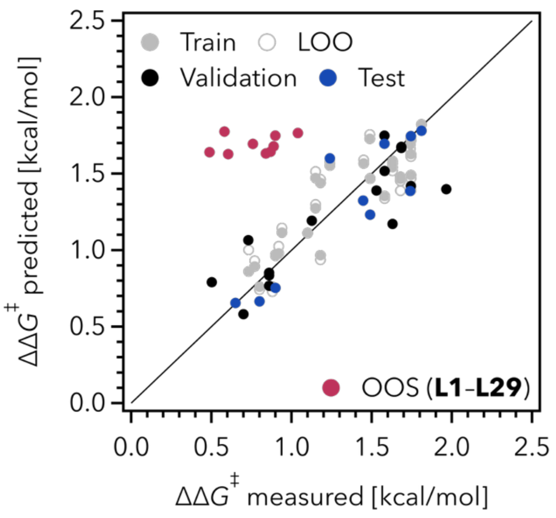

Figure S9. MLR analysis of ligand screening + substrate scope data for the enantioselective arylation of styrene oxides using only features extracted from **TSRE**.

Based on the benchmarking results in case study 1, we initially sought to train an interpretable MLR model using only features extracted from the reductive elimination transition state. To this end, MLR analysis was performed using an in-house Jupyter Notebook (https://github.com/SigmanGroup/python-modeling/Mattlab_modeling_v6.0.0.ipynb), which employs scikit-learn and forward stepwise linear regression to identify predictive combinations of features. Descriptors were first normalized to allow for direct interpretation of the coefficients' signs and magnitudes. Collinear ($R^2 \geq 0.7$) descriptors were not permitted in the same model. Models were filtered based on statistical measures and the number of parameters. Data (53 reactions) was split into train (grey points in Figure S9, 28 reactions), validation (black, 0.3 ratio, 15), and (internal) test (blue, 0.2 ratio, 10) sets using the Kennard-Stone algorithm. Data acquired for **P16** and **P23** with **L11**, **L15–16** and **L7–8**, **L14**, **L26–27** and **L29**, respectively, was used as an additional out-of-sample test set (9 reactions). The model S1 was identified with the following statistics: train $R^2 = 0.77$, $Q^2 = 0.69$, MAE = 0.14, RMSE = 0.17, 5-fold $R^2 = 0.67$; validation $R^2 = 0.72$, MAE = 0.18, RMSE = 0.25; test $R^2 = 0.76$, MAE = 0.15 kcal/mol.

(S1) $$\Delta\Delta G^{\ddagger} = 0.14 \times \%V_{\text{bur 3.0 Å (C1s)}}^{\text{max}} - 0.21 \times \%V_{\text{bur 4.0 Å (C2s)}}^{\text{max}} + 0.13 \times \text{Sterimol B1}_{(\text{Ni}\rightarrow\text{C1s})}^{\text{min}} - 0.18 \times d_{(\text{Ni–C2s})}^{\text{min}} + 1.35$$

Despite the good train/validation/internal test statistics, this model proved ineffective at out-of-sample predictions on **P16** and **P23** with ligands other than **L6** (OOS $R^2 = -1.39$, MAE = 0.92 kcal/mol), prompting the use of a reaction representation constructed with **TSRC–Int–TSRE** features.

3.5 MLR with TSRC–Int–TSRE features

To limit the size of the input representation and reduce the computational cost of the exhaustive feature selection, descriptors were pre-filtered *via* the Boruta algorithm, implemented in an in-house Jupyter Notebook (https://github.com/SigmanGroup/python-modeling/feature_curation.ipynb, percentage of maximum importance of shadow features = 60, maximum number of iterations = 70). Only Boltzmann-weighted average values were included for electronic descriptors, whereas min, max, and average values were used for steric parameters. Combinations of 4 features were selected *via* the repeated, stratified, nested *k*-fold CV scheme with a collinearity cutoff ≥ 0.5. Each cross-validation fold (inner and outer) was stratified with respect to the ligand class (BiIm, BnBiOx, and non-BnBiOx, 29 data points, **L1**–**L29**), or the nature of the substrate being varied (*i.e.*, aryl iodide, 16 data points, **P3**–**P18** *vs*. epoxide, 8 data points, **P19**–**P26**). Based on the 5×2 CV test, 5 models were selected (Table S6). Using a python script (available on GitHub), the predicted $\Delta\Delta G^{\ddagger}$ values of the 5 MLR models (averaged across the 10 folds of the 5×2 test) were averaged to obtain the ensemble $\Delta\Delta G^{\ddagger}$ values and the corresponding standard deviations (Table S7). Each $\Delta\Delta G^{\ddagger}$ value from the 5 MLR model was weighted according to the model's mean test RMSE and outliers across the 5 models were excluded based on the *z*-score (± 2).

Table S6. Linear models selected for ensemble predictions. Combinations of features were selected *via* the repeated, stratified, nested *k*-fold CV scheme[a] and evaluated *via* the 5×2 CV test.[b]

Features	Avg. Train RMSE[a]	Avg. Test RMSE[a]	Avg. Test Adj. R^{2}[a]	Test RMSE (mean, SD)[b]
$\%V_{bur\ 3.5\ Å\ (N2)}^{Int,\ min}$<br>Buried Sterimol $B1_{(N2\to Ni,\ 4.5\ Å)}^{Int,\ min}$<br>Sterimol $B1_{(Ni\to C1s)}^{TSRE,\ min}$<br>Hirsh atom $dipole_{(Br)}^{TSRE,\ Boltz}$	0.25	0.28	0.57	(0.29, 0.04)
$d_{(Ni–C2s)}^{Int,\ Boltz}$<br>$\%V_{bur\ 3.5\ Å\ (N2)}^{Int,\ min}$<br>Hirsh atom $dipole_{(H1s)}^{Int,\ Boltz}$<br>Hirsh atom $dipole_{(Br)}^{TSRE,\ Boltz}$	0.24	0.27	0.60	(0.30, 0.03)
$\theta_{(N1–Ni–C2s)}^{TSRC,\ min}$<br>$d_{(Ni–C2s)}^{Int,\ Boltz}$<br>$\%V_{bur\ 3.5\ Å\ (N2)}^{Int,\ min}$<br>Hirsh atom $dipole_{(Br)}^{TSRE,\ Boltz}$	0.24	0.27	0.59	(0.30, 0.04)
Sterimol $L_{(N2\to Ni)}^{Int,\ min}$<br>$d_{(Br–Ni)}^{TSRE,\ max}$<br>Sterimol $B1_{(Ni\to C1s)}^{TSRE,\ min}$<br>Hirsh atom $dipole_{(Br)}^{TSRE,\ Boltz}$	0.25	0.30	0.56	(0.32, 0.06)
$HOMO^{TSRC,\ Boltz}$<br>$d_{(Ni–C2s)}^{TSRE,\ min}$<br>Buried Sterimol $B1_{(N1\to Ni,\ 4.5\ Å)}^{TSRE,\ max}$<br>Buried Sterimol $L_{(C2\to R1,\ 4.5\ Å)}^{TSRE,\ max}$	0.25	0.29	0.56	(0.29, 0.04)

Table S7. Measured and predicted $\Delta\Delta G^{\ddagger}$ values using ensemble of MLR models with linear parameters[a] (see Table S6) and SISSO-augmented features (see Table S10).[b] SD = standard deviation.

Structure	Class	Expt.		Linear[a]		SISSO[b]	
		ee	**$\Delta\Delta G^{\ddagger}$**	**$\Delta\Delta G^{\ddagger}$**	**SD**	**$\Delta\Delta G^{\ddagger}$**	**SD**
L1_P0	BnBiOx	59	0.80	0.86	0.15	0.97	0.04
L2_P0	non-BnBiOx	88	1.63	1.21	0.05	1.48	0.09
L3_P0	non-BnBiOx	90	1.74	1.48	0.11	1.61	0.11
L4_P0	BiIm	87	1.58	1.44	0.16	1.46	0.05
L5_P0	BiIm	90	1.74	1.44	0.15	1.82	0.13
L6_P0	BiIm	89	1.68	1.57	0.15	1.60	0.14
L7_P0	BiIm	85	1.49	1.53	0.16	1.32	0.03
L8_P0	BiIm	75	1.15	1.34	0.16	1.39	0.05
L9_P0	non-BnBiOx	65	0.92	0.94	0.24	1.20	0.10
L10_P0	non-BnBiOx	64	0.90	0.84	0.08	1.22	0.06
L11_P0	non-BnBiOx	66	0.94	1.04	0.23	1.13	0.06
L12_P0	non-BnBiOx	89	1.68	1.31	0.15	1.54	0.11
L13_P0	non-BnBiOx	76	1.18	1.10	0.19	1.21	0.08
L14_P0	non-BnBiOx	87	1.58	1.25	0.19	1.35	0.13
L15_P0	non-BnBiOx	73	1.10	1.21	0.23	1.17	0.08
L16_P0	BnBiOx	62	0.86	0.85	0.10	0.75	0.03
L17_P0	BnBiOx	62	0.86	1.05	0.12	0.80	0.05
L18_P0	BnBiOx	62	0.86	0.70	0.14	0.56	0.04
L19_P0	BnBiOx	57	0.77	0.91	0.19	1.01	0.08
L20_P0	BnBiOx	63	0.88	0.88	0.21	0.81	0.04
L21_P0	BnBiOx	53	0.65	0.92	0.16	0.60	0.10
L22_P0	BnBiOx	59	0.80	0.98	0.12	0.75	0.07
L23_P0	BnBiOx	64	0.90	1.03	0.31	0.84	0.03
L24_P0	BnBiOx	55	0.73	0.89	0.08	0.61	0.03
L25_P0	BnBiOx	53	0.70	1.07	0.15	0.96	0.08
L26_P0	BiIm	85	1.49	1.53	0.12	1.79	0.08
L27_P0	BiIm	84	1.45	1.41	0.17	1.34	0.09
L28_P0	BiIm	75	1.15	1.27	0.12	1.24	0.12
L29_P0	BiIm	86	1.53	1.46	0.09	1.30	0.03
L6_P3	Aryl Iodide	90	1.74	1.61	0.06	1.58	0.04
L6_P4	Aryl Iodide	88	1.63	1.55	0.23	1.63	0.07
L6_P5	Aryl Iodide	87	1.58	1.62	0.13	1.62	0.04
L6_P6	Aryl Iodide	90	1.74	1.61	0.12	1.73	0.04
L6_P7	Aryl Iodide	90	1.74	1.60	0.13	1.71	0.06
L6_P8	Aryl Iodide	90	1.74	1.64	0.09	1.77	0.06
L6_P9	Aryl Iodide	93	1.96	1.59	0.14	1.80	0.10
L6_P10	Aryl Iodide	90	1.74	1.62	0.12	1.74	0.05
L6_P11	Aryl Iodide	87	1.58	1.54	0.10	1.62	0.11
L6_P12	Aryl Iodide	90	1.74	1.54	0.17	1.56	0.06
L6_P13	Aryl Iodide	91	1.81	1.60	0.05	1.56	0.08
L6_P14	Aryl Iodide	91	1.81	1.69	0.05	1.67	0.03
L6_P15	Aryl Iodide	76	1.18	1.44	0.11	1.42	0.05
L6_P16	Aryl Iodide	78	1.24	1.54	0.12	1.45	0.10
L6_P17	Aryl Iodide	55	0.73	1.11	0.22	0.84	0.09
L6_P18	Aryl Iodide	90	1.74	1.65	0.03	1.65	0.07
L6_P19	Epoxide	88	1.63	1.46	0.30	1.52	0.12
L6_P20	Epoxide	89	1.68	1.60	0.18	1.22	0.03
L6_P21	Epoxide	89	1.68	1.53	0.19	1.45	0.15
L6_P22	Epoxide	78	1.24	1.51	0.12	1.39	0.07
L6_P23	Epoxide	74	1.13	1.40	0.19	1.32	0.06
L6_P24	Epoxide	84	1.45	1.59	0.09	1.29	0.02
L6_P25	Epoxide	85	1.49	1.56	0.10	1.51	0.07
L6_P26	Epoxide	40	0.50	1.14	0.14	0.70	0.29

L11_P16	OOS (**L1**–**L29**)	46	0.58	0.49	0.38	0.61	0.23
L15_P16	OOS (**L1**–**L29**)	39	0.49	0.57	0.39	0.51	0.10
L16_P16	OOS (**L1**–**L29**)	47	0.60	0.40	0.28	0.23	0.08
L7_P23	OOS (**L1**–**L29**)	64	0.89	1.12	0.29	1.00	0.16
L8_P23	OOS (**L1**–**L29**)	57	0.76	0.98	0.26	0.85	0.04
L14_P23	OOS (**L1**–**L29**)	71	1.04	1.03	0.32	0.85	0.07
L26_P23	OOS (**L1**–**L29**)	64	0.90	1.14	0.37	0.99	0.18
L27_P23	OOS (**L1**–**L29**)	63	0.87	1.10	0.23	0.84	0.08
L29_P23	OOS (**L1**–**L29**)	61	0.84	0.95	0.20	0.81	0.06
L65_P23	OOS (Virtual)	72	1.06	1.45	0.33	1.36	0.07
L66_P23	OOS (Virtual)	85	1.47	1.50	0.18	1.43	0.09
L144_P23	OOS (Virtual)	70	1.02	1.43	0.21	1.40	0.04

3.6 Nonlinear parameters generation and modeling

The SISSO algorithm applies a variety of algebraic functions (Table S8) that transform the input parameters and potentially account for nonlinear relationships between substrates and catalyst; the SISSO-augmented features can then be linearly regressed against the experimental output ($\Delta\Delta G^{\ddagger}$). The nonlinear descriptor set used in modeling was generated from a subset of 50 **TSRC–Int–TSRE** features that was identified as being predictive in multiple MLR modeling attempts (Table S9). A Python code (available on GitHub) was written based on our previously implemented algorithm[26] where univariate and bivariate operations are performed on the input descriptor set over two iterations. In iteration 1, SISSO-augmented descriptors are filtered according to collinearity ($R^2 \geq 0.7$) and relative *p*-value permutation testing to ensure that the complexity introduced by nonlinearity was not less statistically significant than the original set (1,000 permutations, p-value = 0.001). In iteration 2, Boruta feature selection was applied (percentile threshold of shadow features = 75, maximum number of iterations = 100), resulting in 4,254 SISSO-augmented descriptors. This set (together with the initial 50 linear parameters) was then used as input of the repeated, stratified, nested *k*-fold CV scheme. Based on the 5×2 CV test, 9 4-feature MLR models were identified as being predictive (Table S10) and used for ensemble modeling (Table S7).

Table S8. Algebraic functions used in SISSO descriptor generation.

**Univariate Operators**	**Bivariate Operators**
$\sqrt{a}$, $\sqrt[3]{a}$, a^{-1}, a^2, a^3, ln(a), e^a	a + b, a – b, a × b, a / b

Table S9. Linear **TSRC–Int–TSRE** parameters used to generate SISSO-augmented descriptors.

**Name**	**Feature**
HOMO$^{\text{TSRC, Boltz}}$	x1
$d_{\text{(N2–Ni)}}{}^{\text{TSRC, Boltz}}$	x53
$\theta_{\text{(N1–Ni–C2s)}}{}^{\text{TSRC, min}}$	x86
%$V_{\text{bur 4.0 Å (N1)}}{}^{\text{TSRC, min}}$	x238
Sterimol $L_{\text{(Ni}\rightarrow\text{C2s)}}{}^{\text{TSRC, max}}$	x315
Buried Sterimol $L_{\text{(N1}\rightarrow\text{Ni, 4.5 Å)}}{}^{\text{TSRC, max}}$	x375
Buried Sterimol $L_{\text{(Ni}\rightarrow\text{C1s, 4.5 Å)}}{}^{\text{TSRC, min}}$	x382
%$V_{\text{bur, no H 3.5 Å (Ni)}}{}^{\text{TSRC, Boltz}}$	x681
%$V_{\text{bur, no H 3.5 Å (Br)}}{}^{\text{TSRC, Boltz}}$	x685
%$V_{\text{bur, no H 3.5 Å (C1s)}}{}^{\text{TSRC, max}}$	x691
$d_{\text{(Ni–C2s)}}{}^{\text{Int, Boltz}}$	x817
%$V_{\text{bur 2.0 Å (C1s)}}{}^{\text{Int, Boltz}}$	x889
%$V_{\text{bur 3.5 Å (N2)}}{}^{\text{Int, min}}$	x974
%$V_{\text{bur 4.0 Å (Br)}}{}^{\text{Int, min}}$	x982
Sterimol $B1_{\text{(C5}\rightarrow\text{R2)}}{}^{\text{Int, min}}$	x1006
Sterimol $B1_{\text{(Ni}\rightarrow\text{C1s)}}{}^{\text{Int, Boltz}}$	x1017
Sterimol $B5_{\text{(C5}\rightarrow\text{R2)}}{}^{\text{Int, Boltz}}$	x1029
Sterimol $L_{\text{(N2}\rightarrow\text{Ni)}}{}^{\text{Int, min}}$	x1062
Buried Sterimol $B1_{\text{(C5}\rightarrow\text{R2, 4.5 Å)}}{}^{\text{Int, Boltz}}$	x1077
Buried Sterimol $B1_{\text{(N2}\rightarrow\text{Ni, 4.5 Å)}}{}^{\text{Int, min}}$	x1086
Buried Sterimol $B5_{\text{(C2}\rightarrow\text{R1, 4.5 Å)}}{}^{\text{Int, max}}$	x1099
pyramidalization$_{\text{(Gavrish, C2s)}}{}^{\text{Int, max}}$	x1179
Hirsh atom dipole$_{\text{(C2)}}{}^{\text{Int, Boltz}}$	x1265
Hirsh atom dipole$_{\text{(H1s)}}{}^{\text{Int, Boltz}}$	x1281
%$V_{\text{bur, no H 3.5 Å (Br)}}{}^{\text{Int, min}}$	x1442
$\eta^{\text{TSRE, Boltz}}$	x1521
$d_{\text{(Br–Ni)}}{}^{\text{TSRE, max}}$	x1555
$d_{\text{(Ni–C2s)}}{}^{\text{TSRE, min}}$	x1574
$\varphi_{\text{(N1–C1–C4–N2)}}{}^{\text{TSRE, Boltz}}$	x1625
%$V_{\text{bur 2.0 Å (C1s)}}{}^{\text{TSRE, max}}$	x1647
%$V_{\text{bur 2.5 Å (Br)}}{}^{\text{TSRE, max}}$	x1667
%$V_{\text{bur 2.5 Å (N2)}}{}^{\text{TSRE, min}}$	x1682
%$V_{\text{bur\_2.5 Å (N2)}}{}^{\text{TSRE, max}}$	x1683
%$V_{\text{bur 3.0 Å (Br)}}{}^{\text{TSRE, Boltz}}$	x1689
%$V_{\text{bur 4.0 Å (C2s)}}{}^{\text{TSRE, max}}$	x1747
Sterimol $B1_{\text{(C5}\rightarrow\text{R2)}}{}^{\text{TSRE, min}}$	x1762
Sterimol $B1_{\text{(Ni}\rightarrow\text{C1s)}}{}^{\text{TSRE, min}}$	x1774
Sterimol $B5_{\text{(Ni}\rightarrow\text{C1s)}}{}^{\text{TSRE, Boltz}}$	x1797
Sterimol $L_{\text{(C2}\rightarrow\text{R1)}}{}^{\text{TSRE, max}}$	x1807
Buried Sterimol $B1_{\text{(N1}\rightarrow\text{Ni, 4.5 Å)}}{}^{\text{TSRE, max}}$	x1839
Buried Sterimol $B1_{\text{(Ni}\rightarrow\text{C1s, 4.5 Å)}}{}^{\text{TSRE, Boltz}}$	x1845
Buried Sterimol $B5_{\text{(Ni}\rightarrow\text{C2s, 4.5 Å)}}{}^{\text{TSRE, Boltz}}$	x1873
Buried Sterimol $L_{\text{(C2}\rightarrow\text{R1, 4.5 Å)}}{}^{\text{TSRE, max}}$	x1879
Buried Sterimol $L_{\text{(Ni}\rightarrow\text{C1s, 4.5 Å)}}{}^{\text{TSRE, min}}$	x1894
pyramidalization$_{\text{(Agranat-Radhakrishnan, N1)}}{}^{\text{TSRE, max}}$	x1919
pyramidalization$_{\text{(Gavrish, N2)}}{}^{\text{TSRE, min}}$	x1946
Hirsh atom dipole$_{\text{(Br)}}{}^{\text{TSRE, Boltz}}$	x2009
Spin Density$_{\text{(R2)}}{}^{\text{TSRE, Boltz}}$	x2169
Spin Density$_{\text{(Ni)}}{}^{\text{TSRE_Boltz}}$	x2173
Spin Density$_{\text{(Br)}}{}^{\text{TSRE, Boltz}}$	x2189

Table S10. SISSO-augmented MLR models selected for ensemble predictions. Combinations of features were selected *via* the repeated, stratified, nested *k*-fold CV scheme[a] and evaluated *via* the 5×2 CV test.[b]

**Features**	**Avg. Train RMSE[a]**	**Avg. Test RMSE[a]**	**Avg. Test Adj. R^{2}[a]**	**Test RMSE (mean, SD)[b]**
x1797<br>x2173 / (x1017 – x1839)<br>(x974 – x2009) × (x382 × x1)<br>(x2009 × x2173) – (x1647 + x2173)	0.16	0.21	0.86	(0.20, 0.02)
x2173 / (x1017 – x1839)<br>(x974 – x2009) × (x382 × x1)<br>(x1873 / x1) + x1682<br>(x1946 / x1521) × (x1442 / x2009)	0.16	0.24	0.84	(0.19, 0.01)
x2173 / (x1017 – x1839)<br>(x974 – x2009) × (x382 × x1)<br>(x1946 × x2169) / (x681 – x2198)<br>(x2009 × x2173) – (x1647 + x2173)	0.18	0.16	0.80	(0.25, 0.03)
x1797<br>x2173 / (x1017 – x1839)<br>(x974 – x2009) × (x382 × x1)<br>(x1946 / x1521) × (x1442 / x2009)	0.17	0.19	0.82	(0.18, 0.01)
(x974 – x2009) × (x382 × x1)<br>(x1797 / x685) – x1919<br>(x1946 / x1521) × (x1442 / x2009)<br>(x2009 × x2173) – (x1647 + x2173)	0.15	0.21	0.85	(0.21, 0.03)
x2173 / (x1017 – x1839)<br>(x974 – x2009) × (x382 × x1)<br>(x1683 / x1894) + (x1062 / x1017)<br>(x2009 × x2173) – (x1647 + x2173)	0.15	0.23	0.86	(0.21, 0.03)
x1797<br>(x974 – x2009) × (x382 × x1)<br>(x1762 / x685) + (x1555 + x2169)<br>(x2009 × x2173) – (x1647 + x2173)	0.15	0.25	0.85	(0.23, 0.03)
(x974 – x2009) × (x382 × x1)<br>(x1762 / x685) + (x1555 + x2169)<br>(x1919 / x1006) + (x2173 – x1647)<br>(x2009 × x2173) – (x1894 + x2173)	0.16	0.26	0.85	(0.23, 0.03)
x1797<br>(x974 – x2009) × (x382 × x1)<br>(x1683 / x1894) – x1845<br>(x1946 / x1521) × (x1442 / x2009)	0.15	0.27	0.85	(0.21, 0.03)

3.7 Predictions on L1–L29 ligands set

Table S11. Predicted $\Delta\Delta G^{\ddagger}$ values using ensemble of MLR models with linear parameters[a] (see Table S6) and SISSO-augmented features (see Table S10).[b] SD = standard deviation.

Structure	Linear[a]		SISSO[b]	
	$\Delta\Delta G^{\ddagger}$	SD	$\Delta\Delta G^{\ddagger}$	SD
L1_P16	0.39	0.31	0.40	0.17
L2_P16	0.57	0.50	0.19	0.52
L3_P16	0.71	0.44	-0.55	0.95
L4_P16	0.73	0.56	0.72	0.18
L5_P16	0.73	0.55	0.67	0.12
L6_P16	0.79	0.42	0.64	0.22
L7_P16	0.76	0.43	0.40	0.25
L8_P16	0.74	0.44	0.73	0.08
L9_P16	0.56	0.55	-0.20	0.60
L10_P16	0.53	0.39	-0.05	0.45
L11_P16	0.49	0.38	0.59	0.16
L12_P16	0.56	0.45	1.51	1.58
L13_P16	0.44	0.43	0.11	0.34
L14_P16	0.55	0.41	-0.10	0.77
L15_P16	0.57	0.39	0.56	0.10
L16_P16	0.40	0.28	0.35	0.09
L17_P16	0.31	0.53	0.12	0.25
L18_P16	0.32	0.16	0.02	0.18
L19_P16	0.23	0.40	0.30	0.17
L20_P16	0.39	0.40	0.24	0.12
L21_P16	0.19	0.22	0.48	0.27
L22_P16	0.46	0.26	2.16	1.90
L23_P16	0.53	0.41	0.27	0.07
L24_P16	0.29	0.22	0.26	0.07
L25_P16	0.72	0.58	0.62	0.09
L26_P16	0.80	0.50	0.84	0.11
L27_P16	0.80	0.45	0.62	0.08
L28_P16	0.87	0.61	0.61	0.17
L29_P16	0.76	0.62	0.47	0.15
L1_P23	0.65	0.21	0.42	0.09
L2_P23	1.01	0.25	0.97	0.09
L3_P23	0.82	0.21	0.98	0.14
L4_P23	0.96	0.09	0.91	0.09
L5_P23	1.02	0.13	1.07	0.12
L6_P23	1.01	0.44	1.06	0.12
L7_P23	1.12	0.29	0.99	0.13
L8_P23	0.98	0.26	0.87	0.03
L9_P23	0.57	0.38	0.36	0.16
L10_P23	0.40	0.42	0.59	0.05
L11_P23	0.76	0.35	0.83	0.06
L12_P23	0.95	0.22	1.06	0.11
L13_P23	0.72	0.19	0.42	0.18

L14_P23	1.03	0.32	0.88	0.08
L15_P23	0.75	0.34	0.49	0.08
L16_P23	0.55	0.32	0.45	0.10
L17_P23	0.51	0.31	0.47	0.13
L18_P23	0.40	0.18	0.21	0.13
L19_P23	0.39	0.50	0.44	0.41
L20_P23	0.67	0.30	0.39	0.12
L21_P23	0.69	0.22	0.26	0.10
L22_P23	0.54	0.26	0.07	0.70
L23_P23	0.59	0.15	0.25	0.19
L24_P23	0.18	0.56	0.07	0.16
L25_P23	0.98	0.45	0.57	0.09
L26_P23	1.14	0.37	0.98	0.15
L27_P23	1.10	0.23	0.87	0.09
L28_P23	0.97	0.16	0.53	0.14
L29_P23	0.95	0.20	0.83	0.04

3.8 Predictions on Virtual Ligands set

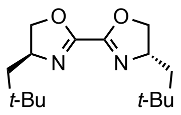

L45

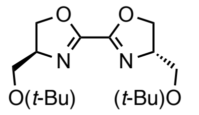

L46

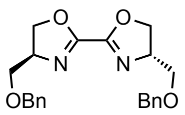

L50

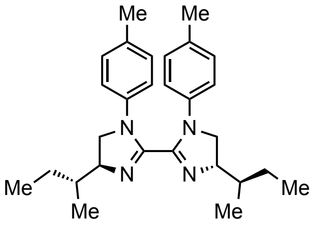

L65

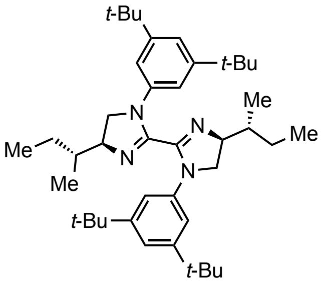

L66

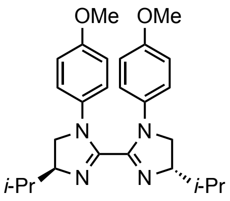

L71

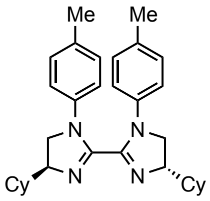

L72

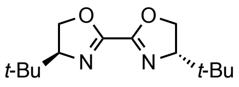

L73

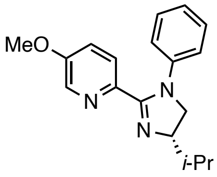

L118

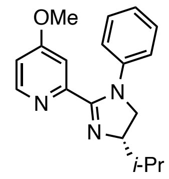

L119

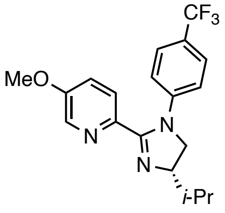

L120

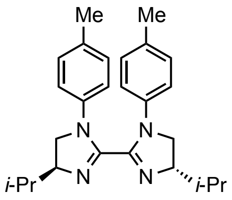

L124

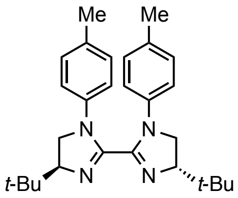

L127

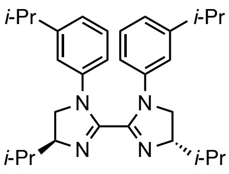

L143

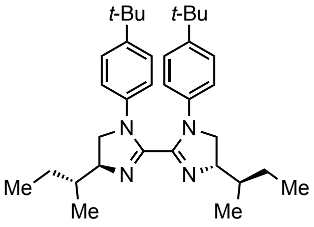

L144

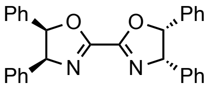

L145

Figure S10. Ligands in the virtual set.

Table S12. Predicted $\Delta\Delta G^{\ddagger}$ values using ensemble of MLR models with linear parameters[a] (see Table S6) and SISSO-augmented features (see Table S10).[b] SD = standard deviation.

Structure	Linear[a]		SISSO[b]	
	$\Delta\Delta G^{\ddagger}$	SD	$\Delta\Delta G^{\ddagger}$	SD
L45_P16	0.39	0.54	0.36	0.11
L46_P16	0.38	0.53	0.39	0.02
L50_P16	0.38	0.36	0.35	0.04
L65_P16	0.73	0.48	0.73	0.06
L66_P16	0.75	0.46	0.81	0.07
L71_P16	0.77	0.38	0.76	0.06
L72_P16	0.79	0.37	0.84	0.20
L73_P16	0.60	0.51	0.67	0.06
L118_P16	0.33	0.39	-0.10	0.19
L119_P16	0.36	0.30	1.03	0.95
L120_P16	0.32	0.32	-0.08	0.15
L124_P16	0.88	0.51	0.80	0.01
L127_P16	0.83	0.20	1.08	0.13
L143_P16	0.92	0.47	0.72	0.12
L144_P16	1.04	0.31	0.78	0.04
L145_P16	0.81	0.31	0.49	0.07
L45_P23	1.06	0.19	0.88	0.04
L46_P23	0.98	0.19	0.93	0.09
L50_P23	0.70	0.21	0.27	0.09
L65_P23	1.45	0.33	1.36	0.07
L66_P23	1.50	0.18	1.43	0.09
L71_P23	1.44	0.36	0.88	0.06
L72_P23	1.17	0.47	1.24	0.12
L73_P23	1.18	0.20	1.50	0.10
L118_P23	1.04	0.49	0.56	0.11
L119_P23	0.90	0.34	0.67	0.10
L120_P23	1.05	0.55	0.36	0.16
L124_P23	1.35	0.23	1.53	0.06
L127_P23	1.28	0.20	1.20	0.09
L143_P23	1.42	0.07	1.36	0.05
L144_P23	1.43	0.21	1.40	0.04
L145_P23	1.22	0.18	0.94	0.09

3.9 Non-linear regression

To verify whether non-linear regression methods could better capture complex and dynamic catalyst–substrate interactions, we explored a variety of algorithms (random forest, gradient boosting, and neural networks) with the ROBERT package (https://robert.readthedocs.io/en/latest/index.html).[27,28] Among the regression methods tested, GB (without low permutation feature importance filtering) was found to afford weak predictive accuracy (10×5-fold CV R^2 = 0.51, MAE = 0.22 kcal/mol, RMSE = 0.28 kcal/mol; Test R^2 = 0.74, MAE = 0.18 kcal/mol, RMSE = 0.22 kcal/mol, Figure S11). Additionally, the out-of-sample set (4 BiOx and 5 BiIm ligands tested to form products **16** or **23**) was incorrectly predicted (R^2 = 0.01, MAE = 0.63 kcal/mol, RMSE = 0.67, Figure S11), showing the inability of this non-linear method to predict untested ligand–substrate combinations with only sparse data available for training. The corresponding ROBERT report has been made available on GitHub.

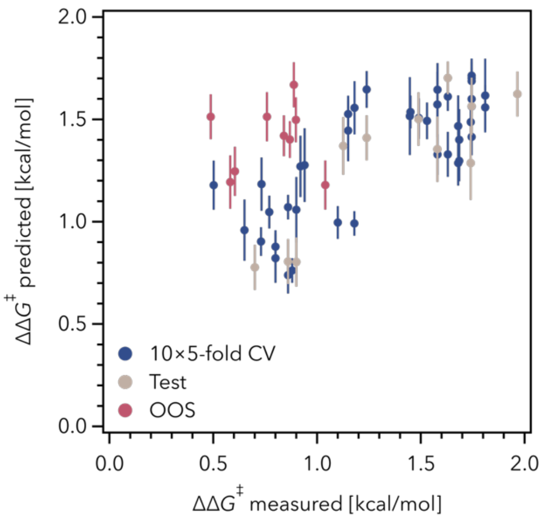

Figure S11. ROBERT Results: GB, standard descriptor filter (no PFI). CV (Train + Validation) : Test = 79 : 21. Points (Train + Validation) : descriptors = 42 : 18.

4. Case Study 2

4.1 Modeling of C(sp^3)–H activation dataset (Model 1)

To limit the size of the input representation and reduce the computational cost of the exhaustive feature selection, descriptors were pre-filtered *via* the Boruta algorithm (https://github.com/SigmanGroup/python-modeling/feature_curation.ipynb, percentage of maximum importance of shadow features = 60, maximum number of iterations = 70). Only Boltzmann-weighted average values were included for electronic descriptors, whereas min, max, and average values were used for steric parameters. Combinations of 4 features were selected *via* the repeated, stratified, nested *k*-fold CV scheme with a collinearity cutoff ≥ 0.7. Each cross-validation fold (inner and outer) was stratified with respect to the ligand class (BiOx, 13 data points; BiIm, 14 data points; or Other for BOX, PyOx, and PyIm ligands, 11 data points). Based on the 5×2 CV test, 10 models were selected for ensemble predictions (Table S13). "Leave-one-reaction-out" analysis results are reported in Table S14 and Table S15.

Table S13. MLR models selected for ensemble predictions. Combinations of features were selected *via* the repeated, stratified, nested *k*-fold CV scheme[a] and evaluated *via* the 5×2 CV test.[b]

Features	Avg. Train RMSE[a]	Avg. Test RMSE[a]	Avg. Test Adj. $R^{2[a]}$	Test RMSE (mean, SD)[b]
$\theta_{(N1–Ni–N2)}^{TSRC, max}$<br>Sterimol $B5_{(C2→R1)}^{TSRC, max}$<br>Buried Sterimol $B5_{(N1→Ni, 4.5 Å)}^{TSRC, min}$<br>$\%V_{bur\ 4.0\ Å\ (Br)}^{Int, Boltz}$	0.20	0.30	0.83	(0.27, 0.03)
$d_{(C1s–C2s)}^{TSRC, min}$<br>$\theta_{(N1–Ni–N2)}^{TSRC, max}$<br>Buried Sterimol $B1_{(Ni→C2s, 4.5 Å)}^{TSRE, max}$<br>$\%V_{bur, no\ H\ 3.5\ Å\ (N2)}^{TSRE, Boltz}$	0.19	0.30	0.81	(0.25, 0.04)
$d_{(C1s–C2s)}^{TSRC, min}$<br>Sterimol $B5_{(C5→R2)}^{Int, min}$<br>Buried Sterimol $B1_{(Ni→C2s, 4.5 Å)}^{TSRE, max}$<br>$\%V_{bur, no\ H\ 3.5\ Å\ (N2)}^{TSRE, Boltz}$	0.20	0.35	0.80	(0.29, 0.06)
$\theta_{(N1–Ni–N2)}^{TSRC, max}$<br>Sterimol $B5_{(C2→R1)}^{TSRC, max}$<br>Buried Sterimol $B5_{(N1→Ni, 4.5 Å)}^{TSRC, min}$<br>$d_{(Br–Ni)}^{Int, max}$	0.17	0.36	0.85	(0.27, 0.04)
$\theta_{(N1–Ni–N2)}^{TSRC, max}$<br>Sterimol $L_{(N1→Ni)}^{TSRC, max}$<br>Buried Sterimol $B5_{(N1→Ni, 4.5 Å)}^{TSRC, min}$<br>$\varphi_{(C4–N2–Ni–C1s)}^{TSRE, Boltz}$	0.16	0.38	0.87	(0.28, 0.06)
$\theta_{(N1–Ni–N2)}^{TSRC, max}$<br>$\theta_{(N1–Ni–C1s)}^{Int, max}$<br>$\varphi_{(N2–Ni–C2s–H1s)}^{Int, max}$<br>Buried Sterimol $L_{(C5→R2, 4.5 Å)}^{TSRE, Boltz}$	0.16	0.39	0.86	(0.29, 0.04)
$d_{(C1s–C2s)}^{TSRC, min}$<br>$d_{(Br–Ni)}^{Int, min}$<br>Buried Sterimol $B1_{(Ni→C2s, 4.5 Å)}^{TSRE, max}$<br>$\%V_{bur, no\ H\ 3.5\ Å\ (N2)}^{TSRE, Boltz}$	0.16	0.39	0.87	(0.26, 0.04)
$\theta_{(N1–Ni–N2)}^{TSRC, max}$<br>Buried Sterimol $B5_{(N1→Ni, 4.5 Å)}^{TSRC, min}$<br>$d_{(Br–Ni)}^{Int, max}$<br>$\theta_{(N1–Ni–C1s)}^{Int, max}$	0.16	0.42	0.85	(0.29, 0.03)
$\theta_{(N1–Ni–N2)}^{TSRC, max}$<br>$d_{(Br–Ni)}^{Int, max}$<br>Sterimol $B5_{(C5→R2)}^{Int, min}$<br>Buried Sterimol $B1_{(Ni→C2s, 4.5 Å)}^{TSRE, max}$	0.13	0.44	0.91	(0.29, 0.04)
$\theta_{(N1–Ni–N2)}^{TSRC, max}$<br>Buried Sterimol $L_{(C2→R1, 4.5 Å)}^{TSRC, min}$<br>$\varphi_{(N2–Ni–C2s–H1s)}^{Int, max}$<br>Sterimol $B5_{(Ni→C1s)}^{Int, Boltz}$	0.21	0.45	0.79	(0.32, 0.06)

Table S14. "Leave-one-reaction-out" (LORO) analysis on the ensemble model for reactions **A**–**C**.

**Reaction**	**Train R^2**	**Train MAE**	**LORO R^2**	**LORO MAE**
**A**	0.70	0.13	0.66	0.14
**B**	0.96	0.13	0.88	0.27
**C**	0.99	0.23	0.84	0.32
**Average**	0.88	0.16	0.79	0.25
**SD**	0.16	0.06	0.11	0.09

Table S15. Measured and predicted $\Delta\Delta G^{\ddagger}$ values using an ensemble of MLR models (see Table S13). [a]LORO values obtained by re-parametrizing the MLR models in the ensemble on two reactions and predicting on the third.

**Structure**	**Ligand**	**Reaction**	***ee***	**Measured $\Delta\Delta G^{\ddagger}$**	**Predicted $\Delta\Delta G^{\ddagger}$**	**SD**	**LORO $\Delta\Delta G^{\ddagger}$[a]**	**LORO SD[a]**
L1_Lu_2019	BiOx	A	57	0.83	0.90	0.11	0.86	0.21
L2_Lu_2019	BiOx	A	65	1.00	1.06	0.21	1.06	0.21
L9_Lu_2019	BiOx	A	65	1.00	0.94	0.10	0.83	0.15
L11_Lu_2019	BiOx	A	63	0.95	0.83	0.05	0.86	0.11
L12_Lu_2019	BiOx	A	59	0.87	0.61	0.11	0.65	0.18
L14_Lu_2019	BiOx	A	51	0.72	0.82	0.10	0.87	0.10
L27_Lu_2019	BiIm	A	73	1.19	1.04	0.16	1.06	0.29
L28_Lu_2019	BiIm	A	49	0.69	1.04	0.26	1.04	0.40
L65_Lu_2019	BiIm	A	76	1.28	1.02	0.08	1.13	0.19
L71_Lu_2019	BiIm	A	74	1.22	1.01	0.14	1.02	0.19
L72_Lu_2019	BiIm	A	77	1.31	1.31	0.07	1.36	0.14
L90_Lu_2019	PyOx	A	29	0.38	0.35	0.13	0.39	0.46
L98_Lu_2019	BiIm	A	59	0.87	1.07	0.18	1.07	0.15
L101_Lu_2019	BiIm	A	47	0.66	0.93	0.20	0.95	0.33
L113_Lu_2019	PyOx	A	29	0.38	0.42	0.06	0.41	0.21
L124_Lu_2019	BiIm	A	78	1.34	1.19	0.10	1.38	0.25
L125_Lu_2019	BiIm	A	-70	1.11	0.94	0.15	0.98	0.32
L126_Lu_2019	BiIm	A	66	1.02	1.03	0.05	1.26	0.15
L128_Lu_2019	BiOx	A	-67	1.04	0.91	0.10	0.97	0.23
L129_Lu_2019	BiOx	A	67	1.04	0.99	0.09	1.00	0.19
L130_Lu_2019	BiOx	A	57	0.83	1.08	0.20	1.08	0.22
L131_Lu_2019	PyIm	A	35	0.47	0.62	0.21	0.74	0.38
L133_Lu_2019	PyOx	A	34	0.45	0.55	0.14	0.57	0.47
L1_Huo_2021	BiOx	B	32	0.37	0.55	0.19	0.58	0.22
L6_Huo_2021	BiIm	B	42	0.50	0.53	0.10	0.68	0.15
L14_Huo_2021	BiOx	B	62	0.82	0.60	0.14	0.53	0.22
L35_Huo_2021	BiOx	B	42	0.50	0.54	0.12	0.60	0.13
L55_Huo_2021	BOX	B	-94	1.96	1.68	0.14	1.26	0.24
L106_Huo_2021	BOX	B	94	1.96	1.83	0.22	1.43	0.40
L113_Huo_2021	PyOx	B	-53	0.66	0.63	0.24	0.76	0.32
L139_Huo_2021	BOX	B	93	1.87	1.83	0.15	1.75	0.31
L140_Huo_2021	BOX	B	94	1.96	1.76	0.04	1.57	0.10
L141_Huo_2021	PyOx	B	-29	0.34	0.52	0.13	0.60	0.30
L142_Huo_2021	BOX	B	-89	1.60	1.41	0.10	1.46	0.16
L6_Lu_2021	BiIm	C	83	1.41	1.10	0.10	1.06	0.09
L12_Lu_2021	BiOx	C	46	0.59	0.74	0.08	0.72	0.09
L31_Lu_2021	BiIm	C	89	1.68	1.49	0.20	1.45	0.19
L66_Lu_2021	BiIm	C	91	1.81	1.35	0.14	1.24	0.16

4.2 Synthesis and characterization of *N*-tosyl-2-phenyl aziridine

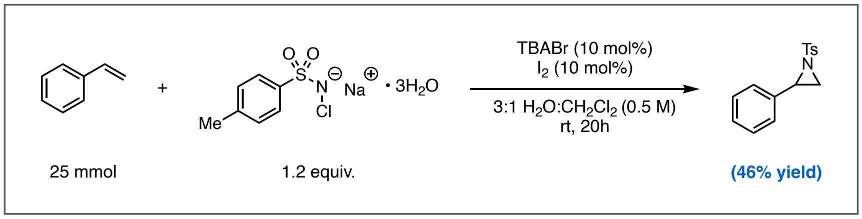

Reaction setup and workup: This procedure was adapted from Doyle *et. al*.[29] A 250 mL round-bottom flask equipped with a stir bar was charged with styrene (2.9 mL, 25 mmol, 1.0 equiv) dissolved in a 0.50 M solution of 3:1 deionized water/dichloromethane. Chloramine-T trihydrate (8.5 g, 30 mmol, 1.2 equiv) was added, followed by tetrabutylammonium bromide (0.81 g, 2.5 mmol, 0.10 equiv) and iodine (0.40 mL, 2.5 mmol, 0.10 equiv). The reaction mixture was stirred at 23 ºC under N_2. After 20 h, the reaction was quenched with saturated solution of sodium thiosulfate and was allowed to stir for 30 minutes. The reaction mixture was transferred to a separatory funnel, where the organic layer was collected, and the aqueous phase was extracted with dichloromethane. The combined organic solvents were collected and washed with sat. sodium thiosulfate (aq) and brine and then dried over $MgSO_4$. The organic solvent was concentrated under reduced pressure and the crude material was purified by flash column chromatography (100% hexanes → 30% EtOAc/hexanes). The product obtained was further crystallized with hot ethyl acetate and hexanes to yield the title compound as a white crystalline solid (3.2 g, 46% yield). Characterization data are in agreement with reported literature values.[30]

^{1}H NMR (500 MHz, $CDCl_3$): δ 7.90 – 7.84 (m, 2H), 7.36 – 7.32 (m, 2H), 7.31 – 7.27 (m, 3H), 7.24 – 7.19 (m, 2H), 3.78 (dd, J = 7.2, 4.4 Hz, 1H), 2.99 (d, J = 7.2 Hz, 1H), 2.43 (s, 3H), 2.39 (d, J = 4.5 Hz, 1H).

^{13}C NMR (126 MHz, $CDCl_3$): δ 144.8, 135.2, 135.1, 129.9, 128.7, 128.4, 128.1, 126.7, 41.2, 36.1, 21.8.

4.3 Preparation and characterization of racemic products

<u>Reaction **D**</u>: N-(2-(4-Methoxyphenyl)-2-phenylethyl)-4-methylbenzenesulfonamide

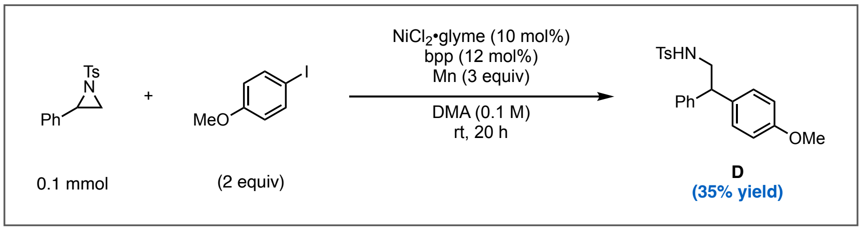

General procedure B: This procedure was adapted from Doyle *et. al*.[31] All reaction preparation was done inside a nitrogen-filled glovebox. Vials were oven-dried (140 °C) and were allowed to cool under vacuum prior to use in the glovebox. To a one-dram vial equipped with a Teflon stir bar were added $NiCl_2$•glyme (2.2 mg, 0.010 mmol, 0.10 equiv), bpp ligand (2.5 mg, 0.012 mmol, 0.12 equiv), and 500 µL DMA. The Ni stock solution was stirred for ~30 minutes. In a separate one-dram reaction vial equipped with a Teflon stir bar, 2-phenyl-1-tosylaziridine (27.3 mg, 0.10 mmol, 1.0 equiv), 1-iodo-4-methoxybenzene (46.8 mg, 0.20 mmol, 2.0 equiv), manganese (16.5 mg, 0.30 mmol, 3.0 equiv), and sodium iodide (30.0 mg, 0.20 mmol, 2.0 equiv) were added. 500 µL DMA and 500 µL of the Ni stock solution were added. The vial was capped with a Teflon septum cap, sealed with electrical tape, removed from the glovebox and stirred at rt for 20 h (700 rpm stir rate). The resulting mixture was diluted with EtOAc, quenched with sat. NH_4Cl solution, and the aqueous layer extracted with EtOAc (2x). The combined organic layers were dried over $MgSO_4$ and concentrated in vacuo. The product was isolated by preparative TLC (30% EtOAc/hexanes) to yield the title compound as a clear oil (13.4 mg, 35% yield). Characterization data are in agreement with reported literature values.[31]

^{1}H NMR (500 MHz, $CDCl_3$): δ 7.68 (dt, J = 8.2, 2.3 Hz, 2H), 7.33 – 7.29 (m, 2H), 7.29 – 7.24 (m, 2H), 7.23 – 7.19 (m, 1H), 7.09 – 7.06 (m, 2H), 7.00 (dt, J = 8.5, 3.0 Hz, 2H), 6.81 (dt, J = 8.7, 2.3 Hz, 2H), 4.27 (t, J = 6.2 Hz, 1H), 4.01 (t, J = 7.9 Hz, 1H), 3.52 (dh, J = 8.2, 6.4 Hz, 2H), 2.45 (s, 3H).

^{13}C NMR (126 MHz, $CDCl_3$): δ 158.8, 143.7, 141.2, 136.9, 132.8, 129.9, 129.1, 129.0, 128.0, 127.3, 127.2, 114.4, 55.4, 49.8, 47.5, 21.7.

SFC method: Daicel ChiralPak IB column, 20% *i*-PrOH, 21 min run, 1.5 mL/min.

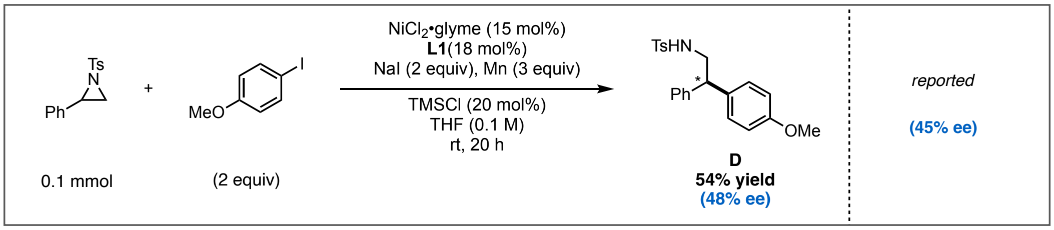

While literature *ee* data for Reaction **D** was sourced from the electrochemical reductive cross-coupling reported by Milo, Mei *et al.*,[7] experimental validation data was collected using reaction conditions that employ a stoichiometric Mn reductant to simplify reaction set-up.[31] To ensure reproducibility between published data used for modeling and our reaction set up, these reaction conditions (using **L1**) were evaluated prior to evaluating additional ligands. Measured *ee* using Mn as a reductant agreed with reported *ee* using electrochemical reduction, using **L1** as the chiral ligand.[7]

Racemic standard:

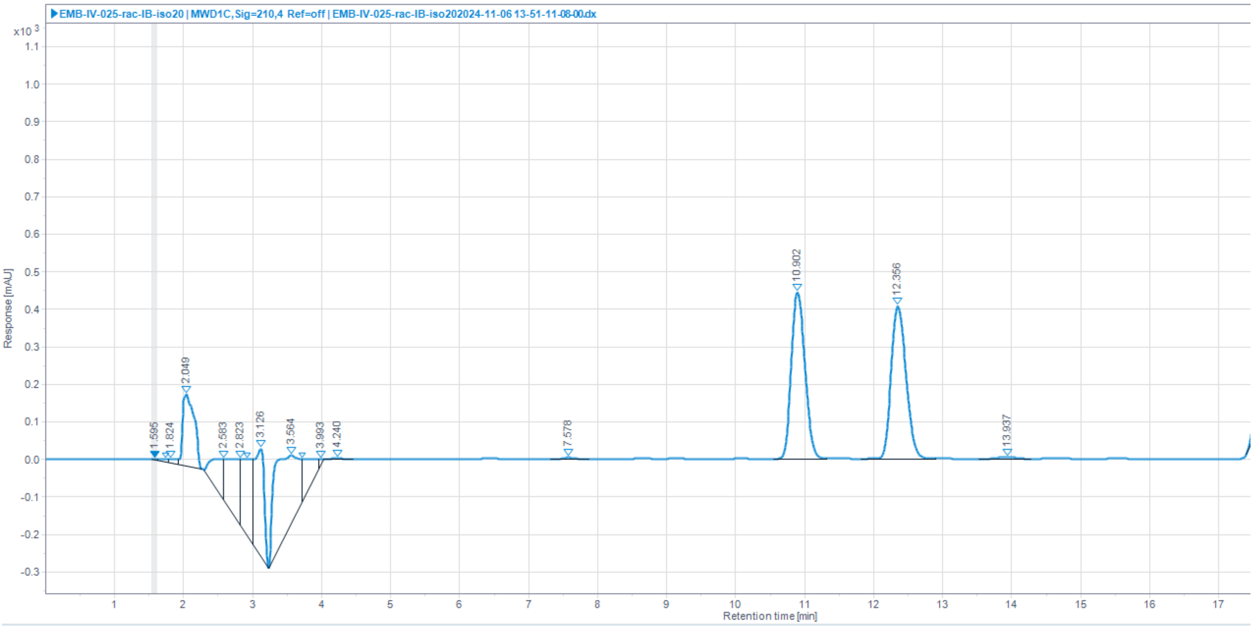

Injection Results

Peaks | Summary

#	Name	Signal description	RT (min)	Area (mAU·s)	Area%	Height (mAU)	Height%	Amount	Concentration	Start time (min)	End time (min)
14		MWD1C,Sig=210,4 Ref=off	10.902	6123.094	9.866	442.737	14.12			10.559	11.323
15		MWD1C,Sig=210,4 Ref=off	12.356	6115.719	9.854	406.584	12.97			11.816	12.900

L1: (48% *ee*)

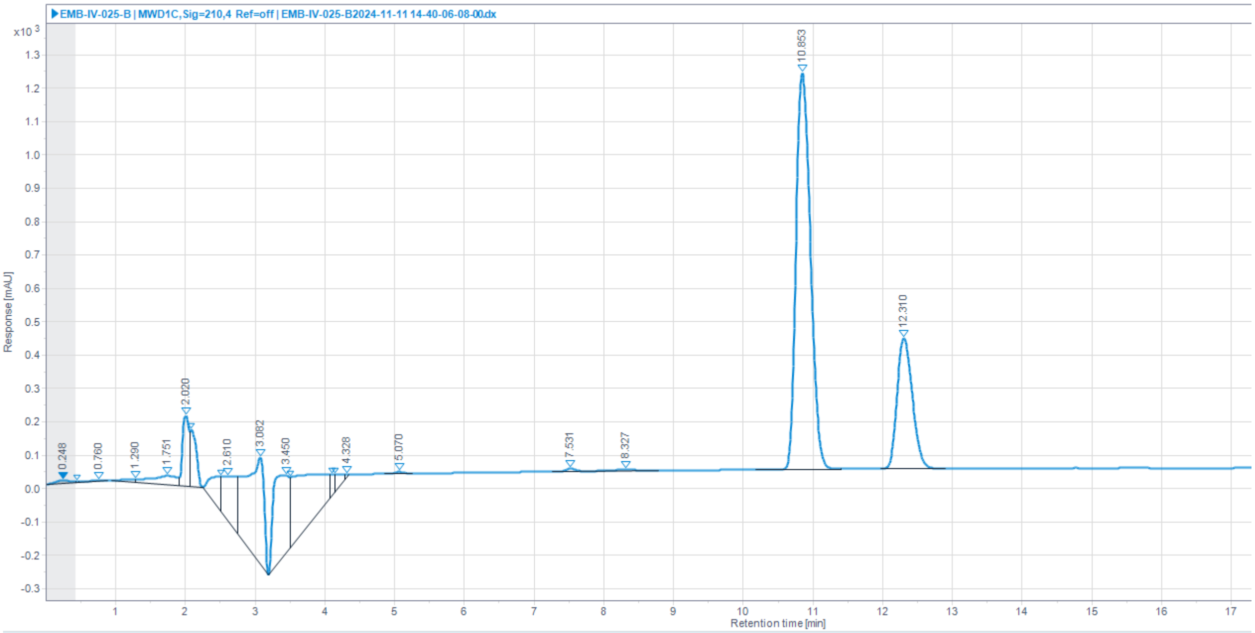

Injection Results

Peaks | Summary

#	Name	Signal description	RT (min)	Area (mAU·s)	Area%	Height (mAU)	Height%	Amount	Concentration	Start time (min)	End time (min)
19		MWD1C,Sig=210,4 Ref=off	10.853	17104.372	16.389	1188.560	22.24			10.190	11.409
20		MWD1C,Sig=210,4 Ref=off	12.310	5999.958	5.749	391.568	7.33			11.984	12.903

Reaction **G**:

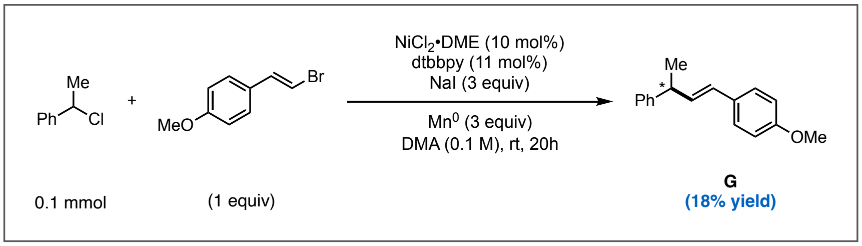

General Procedure C: This procedure was adapted from Reisman *et. al*.[10] All reaction preparation was done inside a nitrogen-filled glovebox. Vials were oven-dried (140 °C) and were allowed to cool under vacuum prior to use in the glovebox. To a one-dram vial equipped with a Teflon stir bar were added $NiCl_2$•glyme (2.2 mg, 0.010 mmol, 0.10 equiv), 4,4'-*t*-Bu-bipyridine (3.0 mg, 0.11 mmol, 0.011 equiv), and 500 µL DMA. The Ni stock solution was stirred for ~30 minutes. In a separate one-dram reaction vial equipped with a Teflon stir bar, (1-chloroethyl)benzene (14.1 mg, 0.10 mmol, 1.0 equiv), (E)-1-(2-bromovinyl)-4-methoxybenzene (21.3 mg, 0.10 mmol, 1.0 equiv), manganese (16.5 mg, 0.30 mmol, 3.0 equiv), sodium iodide (7.5 mg, 0.050 mmol, 0.50 equiv) were added. 500 µL DMA and 500 µL of the Ni stock solution were added. The vial was capped with a Teflon septum cap, sealed with electrical tape, removed from the glovebox and stirred at rt for 20 h (700 rpm stir rate). The resulting mixture was diluted with EtOAc, quenched with sat. NH_4Cl solution, and the aqueous layer extracted with EtOAc (2x). The combined organic layers were dried over $MgSO_4$ and concentrated in vacuo. The product was isolated by preparative TLC (25% PhMe/hexanes) to yield the title compound as a clear oil (4.3 mg, 18% yield). Characterization data are in agreement with reported literature values.[10]

^{1}H NMR (500 MHz, $CDCl_3$): δ 7.35 – 7.19 (m, 7H), 6.85 – 6.81 (m, 2H), 6.36 (d, *J* = 15.9 Hz, 1H), 6.25 (dd, *J* = 15.9, 6.7 Hz, 1H), 3.80 (s, 3H), 3.62 (pd, *J* = 7.2, 1.5 Hz, 1H), 1.46 (d, *J* = 7.0 Hz, 3H).

^{13}C NMR (126 MHz, $CDCl_3$): δ 159.0, 146.1, 133.3, 130.6, 128.6, 128.0, 127.5, 127.4, 126.3, 114.1, 55.5, 42.7, 21.5.

SFC method: Daicel ChiralPak IG column, 10% MeCN, 24 min run, 1.5 mL/min.

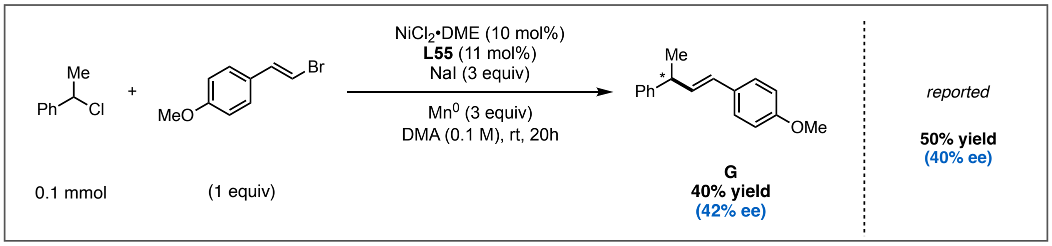

To ensure reproducibility between published data and our reaction set up, the standard reaction conditions using reported ligand **L55** were replicated prior to evaluating additional ligands. Measured *ee* agreed with reported *ee*.[10]

Racemic standard:

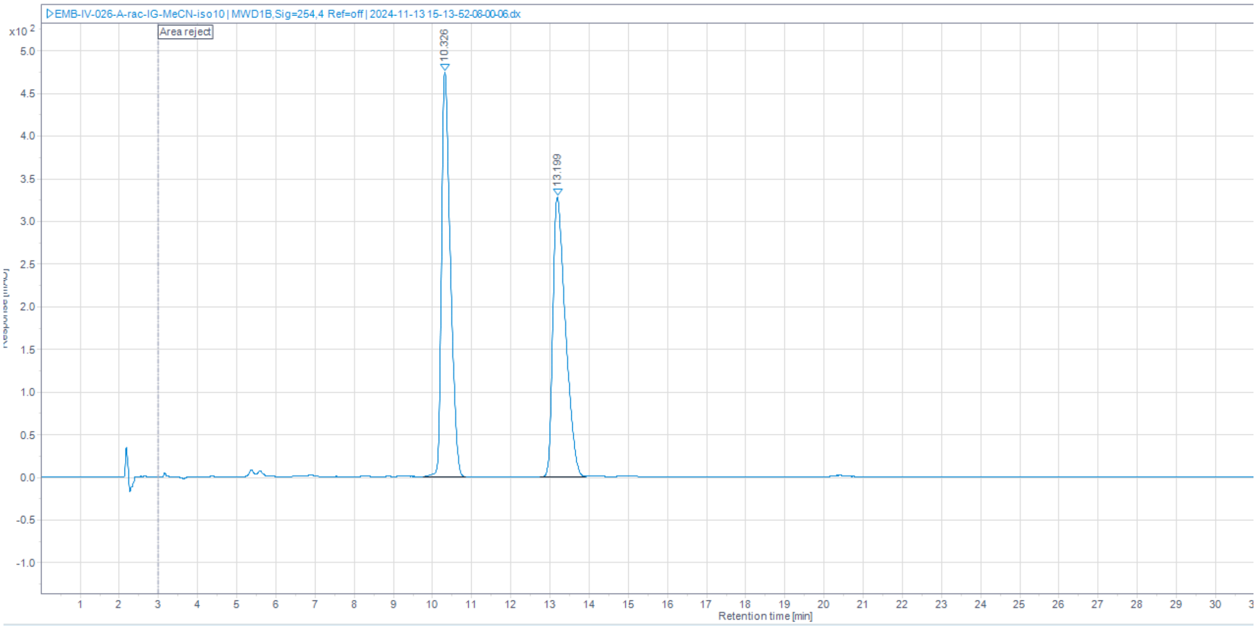

Injection Results

Peaks | Summary

#	Name	Signal description	RT (min)	Area (mAU·s)	Area%	Height (mAU)	Height%	Amount	Concentration	Start time (min)	End time (min)
1		MWD1B,Sig=254,4 Ref=off	10.326	7437.718	34.005	474.050	37.97			9.772	10.845
2		MWD1B,Sig=254,4 Ref=off	13.199	7318.280	33.458	327.347	26.22			12.759	13.923

L55: (42% *ee*)

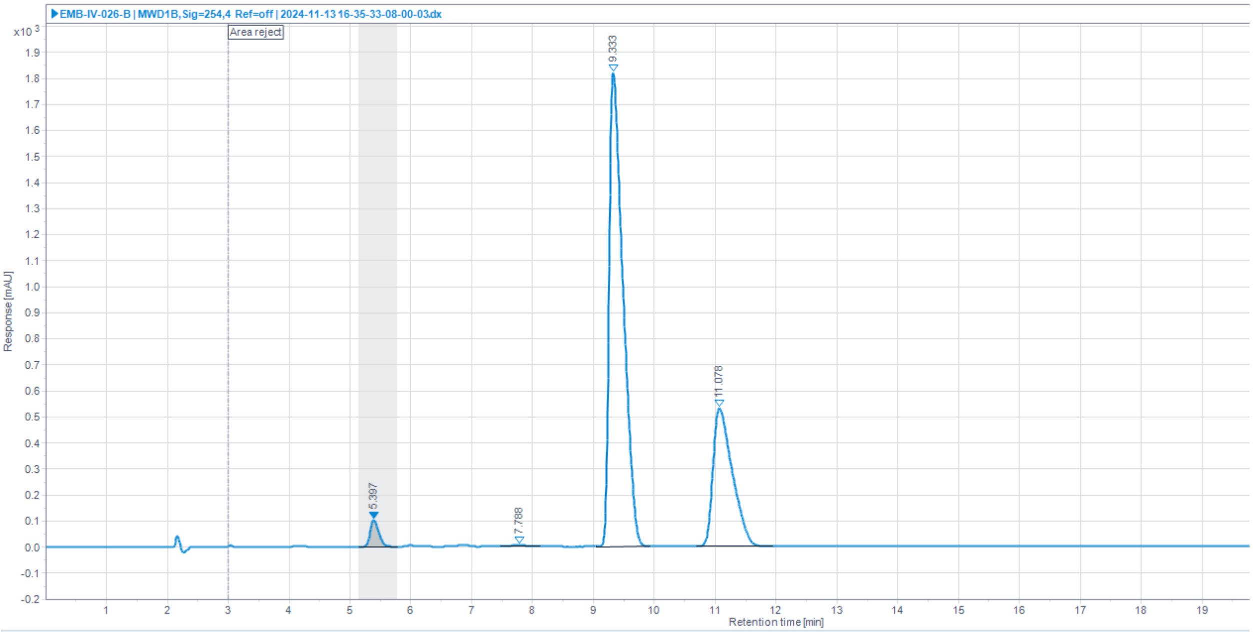

Injection Results

Peaks | Summary

#	Name	Signal description	RT (min)	Area (mAU·s)	Area%	Height (mAU)	Height%	Amount	Concentration	Start time (min)	End time (min)
3		MWD1B,Sig=254,4 Ref=off	9.333	28687.817	61.601	1818.250	65.22			9.055	9.940
4		MWD1B,Sig=254,4 Ref=off	11.078	11728.187	25.184	529.630	19.00			10.702	11.961

4.4 Evaluation of chiral ligands

Reactions (**D)** were set up according to General Procedure **B**, with the following modifications: THF was used as the reaction solvent, TMSCl (2.54 µL, 0.20 mmol, 0.20 equiv) was added, catalyst loading was increased to 15 mol% Ni, 18 mol% L, and the ligand was modified as noted in Table S16. Reactions (**G**) were set up according to General Procedure **C**, modifying the ligand as noted in Table S17. Products were isolated *via* preparative TLC for SFC analysis. Note: only enantioselectivity data was used for modeling. The reaction yields reported were calculated using the mass of product isolated for SFC analysis and were not checked rigorously for purity. We have included these approximate yields to help rationalize lower-than-expected and/or irreproducible experimental *ee* values. Additionally, impurities that did not elute at retention times that obscured integration of product peaks are present in some of the SFC traces reported.

Table S16: Ligand screening results for Reaction **D**.

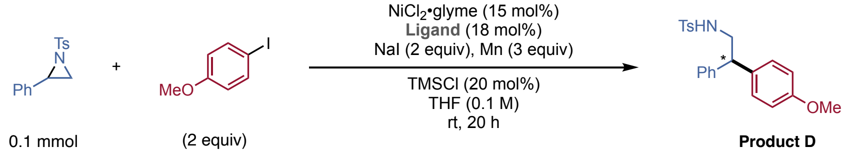

Ligand		Yield D (%)	ee (%)	Ligand		Yield D (%)	ee (%)
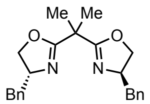 **L57**	run 1	4	6	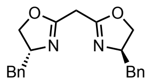 **L61**	run 1	6	6
	run 2	7	6		run 2	5	1
	**average**	**5**	**6***		**average**	**5**	**3***
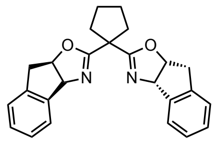 **L63**	run 1	7	22	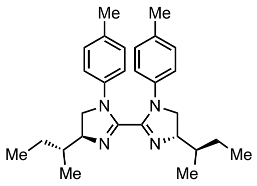 **L65**	run 1	50	68
	run 2	9	37		run 2	40	65
	run 3	< 5	16				
	**average**	**7**	**25***		**average**	**45**	**66**
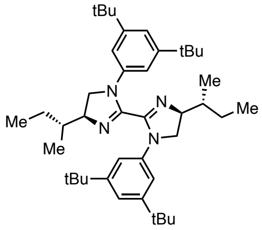 **L66**	run 1	41	91	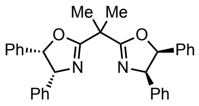 **L68**	run 1	< 5	29
	run 2	48	89		run 2	< 5	67
	**average**	**44**	**90**		**average**	**< 5**	**62***

*Due to low reaction yields (< 1 turnover Ni catalyst), measured *ee* values for reactions using ligands **L57**, **L61**, **L63**, and **L68** were considered unreliable and therefore excluded from the validation data set of models.

Table S17. Ligand screening results for Reaction **G**.

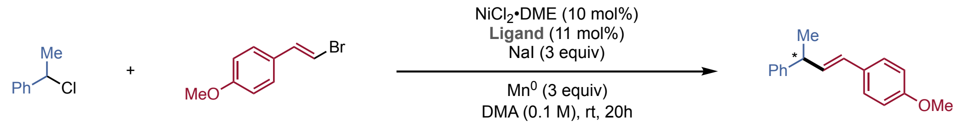

0.1 mmol (1 equiv) **Product G**

Ligand		Yield G (%)	ee (%)	Ligand		Yield G (%)	ee (%)
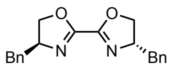	run 1	22	21	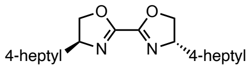	run 1	35	59
	run 2	23	22		run 2	43	59
**L1**	**average**	**22**	**21**	**L3**	**average**	**39**	**59**
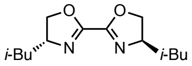	run 1	28	38	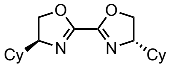	run 1	24	28
	run 2	28	32		run 2	41	34
**L11**	**average**	**28**	**35**	**L14**	**average**	**32**	**31**
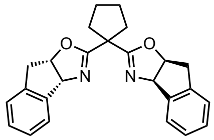	run 1	22	75	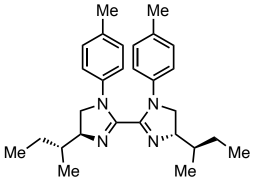	run 1	17	47
	run 2	31	80		run 2	32	55
	run 3	48	77				
**L63**	**average**	**34**	**77**	**L65**	**average**	**24**	**51**
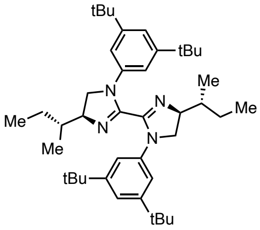	run 1	19	70	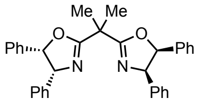	run 1	37	22
	run 2	42	66		run 2	52	23
**L66**	**average**	**30**	**68**	**L68**	**average**	**44**	**22**

4.5 SFC determination of enantiomeric excess

Reaction **D**:

L57: (6% *ee*, 6% *ee*) *data excluded from model validation

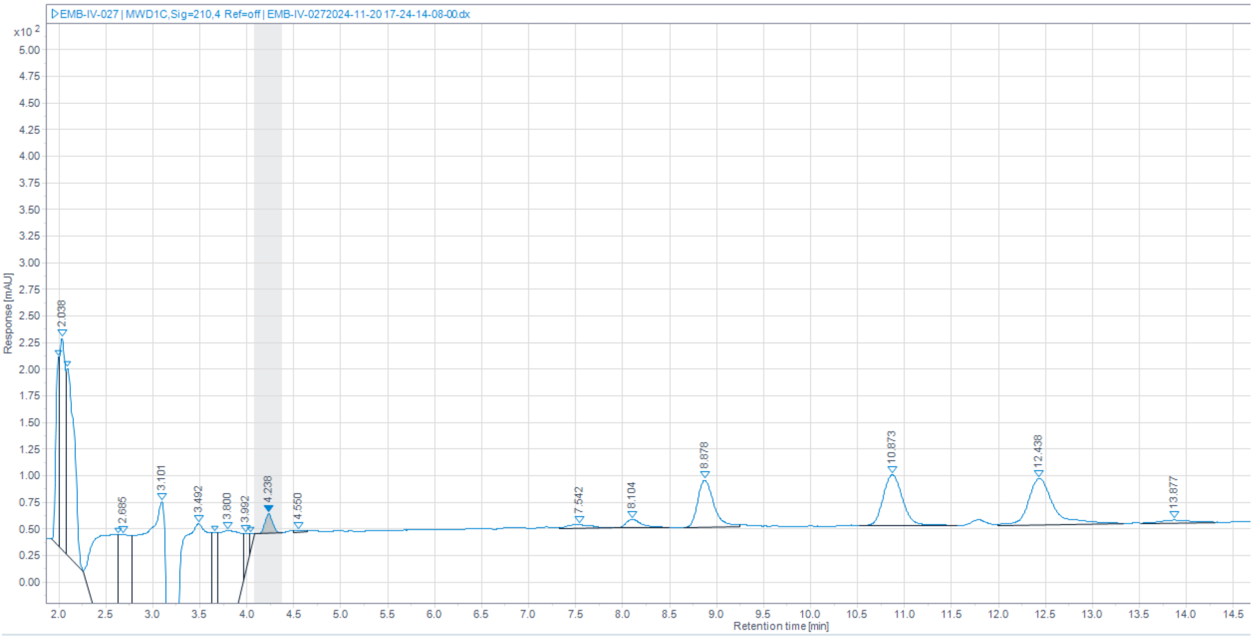

Injection Results

Peaks | Summary

#	Name	Signal description	RT (min)	Area (mAU·s)	Area%	Height (mAU)	Height%	Amount	Concentration	Start time (min)	End time (min)
20		MWD1C,Sig=210,4 Ref=off	10.873	697.179	1.002	47.688	1.28			10.530	11.569
21		MWD1C,Sig=210,4 Ref=off	12.438	784.043	1.126	43.399	1.16			11.998	13.333

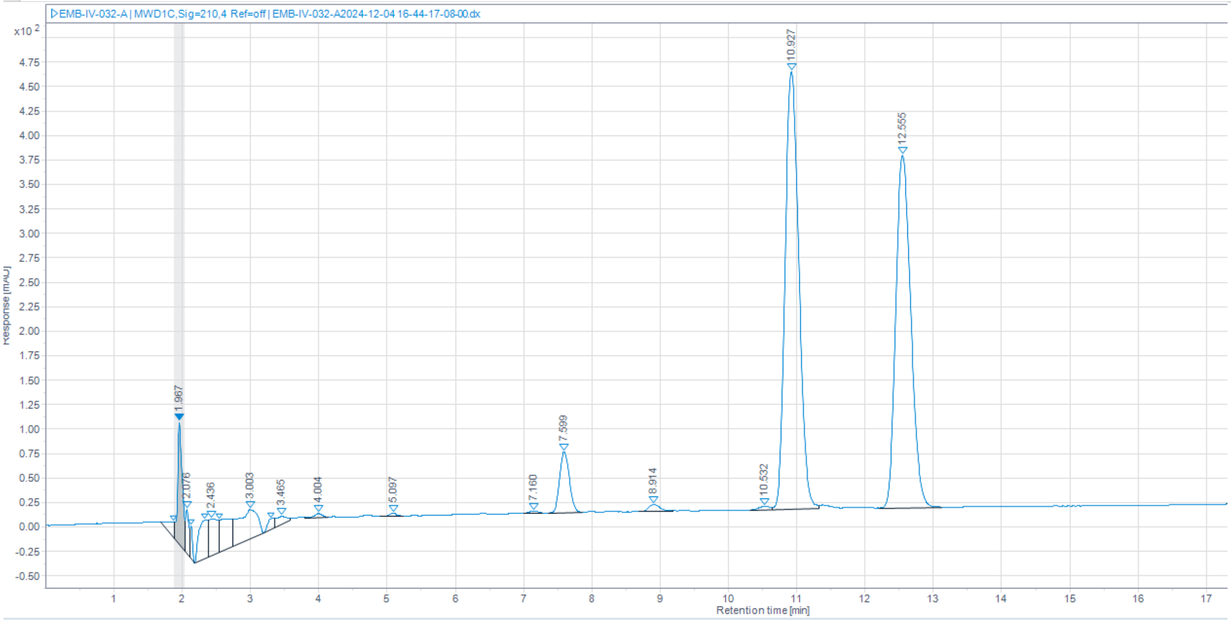

Injection Results

Peaks | Summary

#	Name	Signal description	RT (min)	Area (mAU·s)	Area%	Height (mAU)	Height%	Amount	Concentration	Start time (min)	End time (min)
17		MWD1C,Sig=210,4 Ref=off	10.927	6259.346	31.350	447.126	30.68			10.651	11.337
18		MWD1C,Sig=210,4 Ref=off	12.555	5510.086	27.597	360.383	24.73			12.222	13.132

L61: (6% *ee*, 1% *ee*) *data excluded from model validation

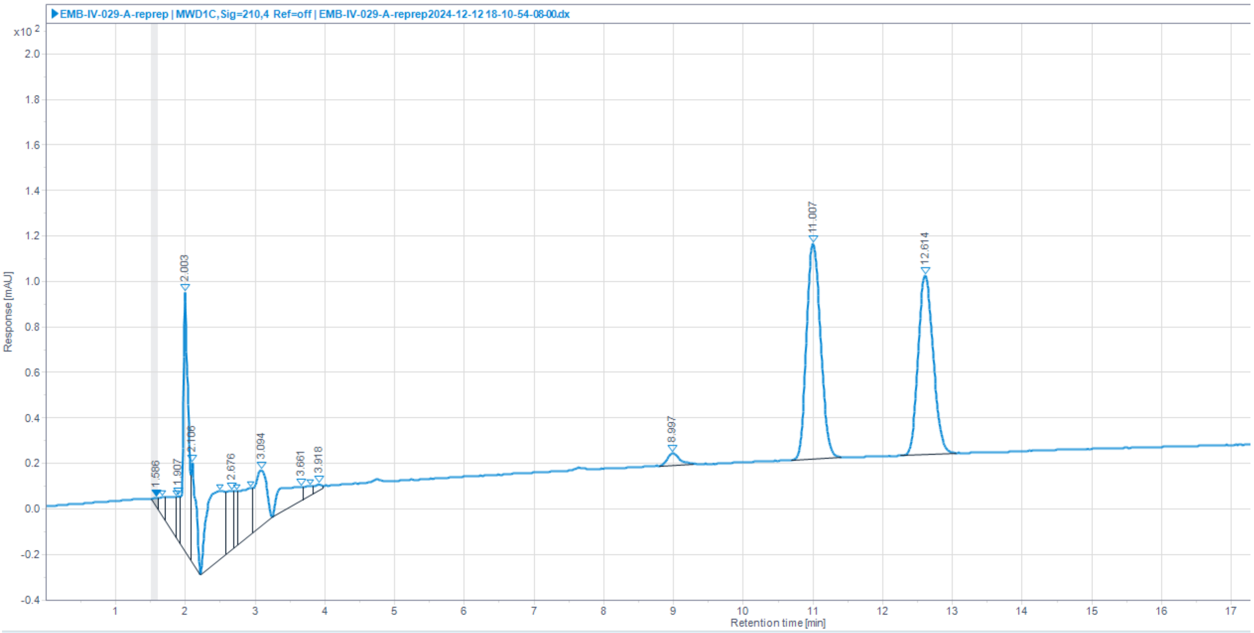

Injection Results

Peaks | Summary

#	Name	Signal description	RT (min)	Area (mAU·s)	Area%	Height (mAU)	Height%	Amount	Concentration	Start time (min)	End time (min)
16		MWD1C,Sig=210,4 Ref=off	11.007	1335.428	13.679	94.484	13.42			10.701	11.405
17		MWD1C,Sig=210,4 Ref=off	12.614	1192.754	12.218	78.859	11.20			12.268	13.063

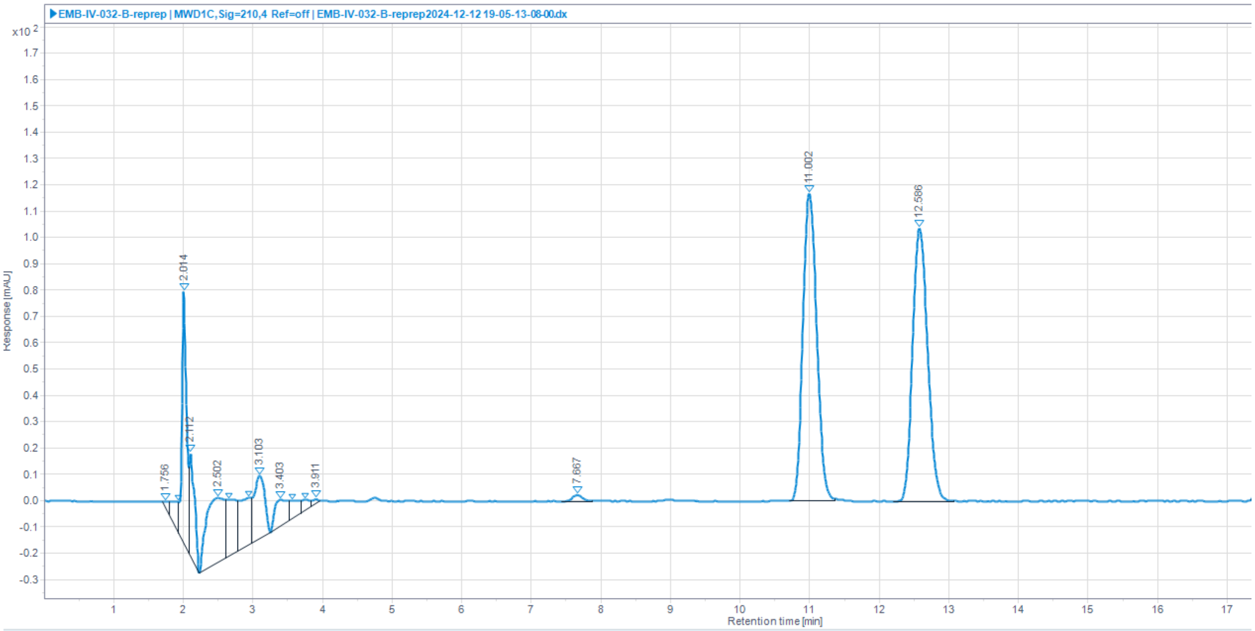

Injection Results

Peaks | Summary

#	Name	Signal description	RT (min)	Area (mAU·s)	Area%	Height (mAU)	Height%	Amount	Concentration	Start time (min)	End time (min)
14		MWD1C,Sig=210,4 Ref=off	11.002	1612.912	16.737	116.766	17.62			10.713	11.366
15		MWD1C,Sig=210,4 Ref=off	12.586	1565.245	16.243	103.787	15.66			12.207	13.083

L63: (22% *ee*, 37% *ee*, 16% *ee*) *data excluded from model validation

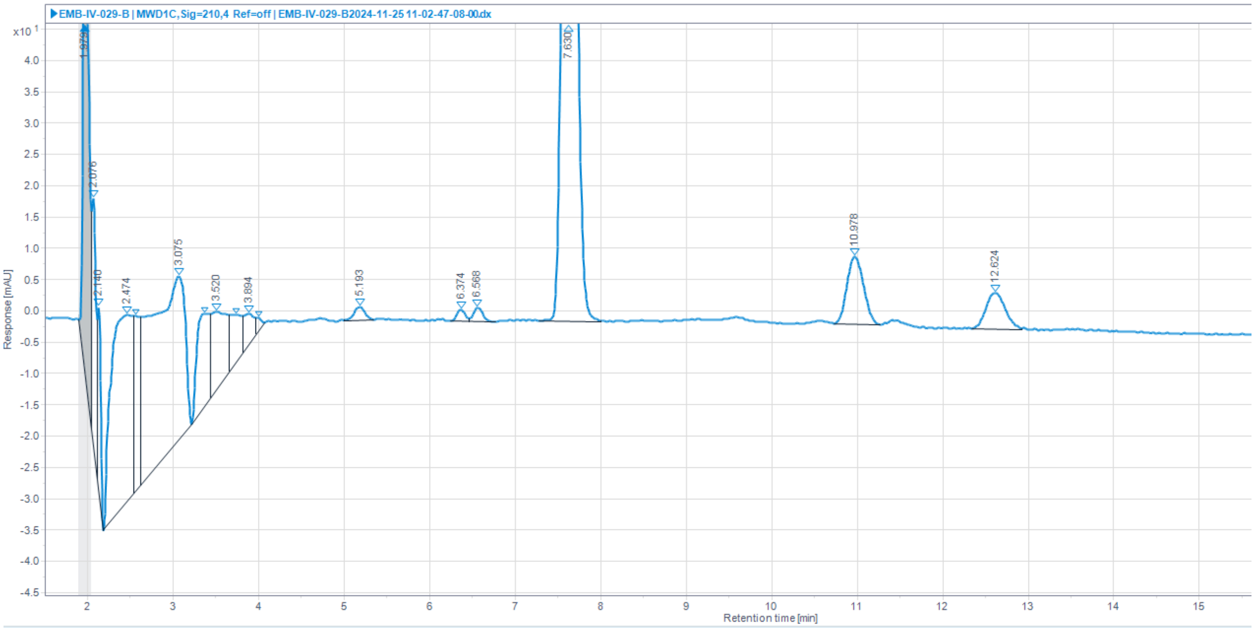

Injection Results

#	Name	Signal description	RT (min)	Area (mAU·s)	Area%	Height (mAU)	Height%	Amount	Concentration	Start time (min)	End time (min)
16		MWD1C,Sig=210,4 Ref=off	10.978	146.265	1.474	10.791	1.57			10.740	11.280
17		MWD1C,Sig=210,4 Ref=off	12.624	92.469	0.932	5.830	0.85			12.348	12.926

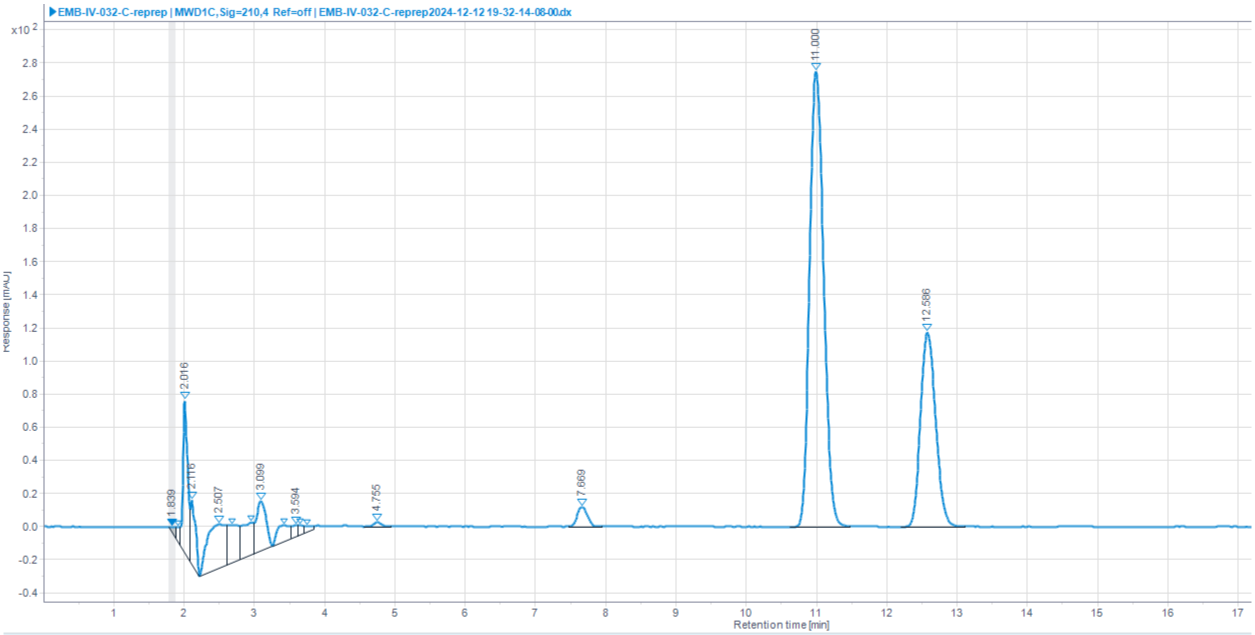

Injection Results

#	Name	Signal description	RT (min)	Area (mAU·s)	Area%	Height (mAU)	Height%	Amount	Concentration	Start time (min)	End time (min)
1		MWD1C,Sig=210,4 Ref=off	1.839	19.841	0.159	3.709	0.38			1.786	1.884
2		MWD1C,Sig=210,4 Ref=off	1.916	32.116	0.257	8.777	0.89			1.884	1.946

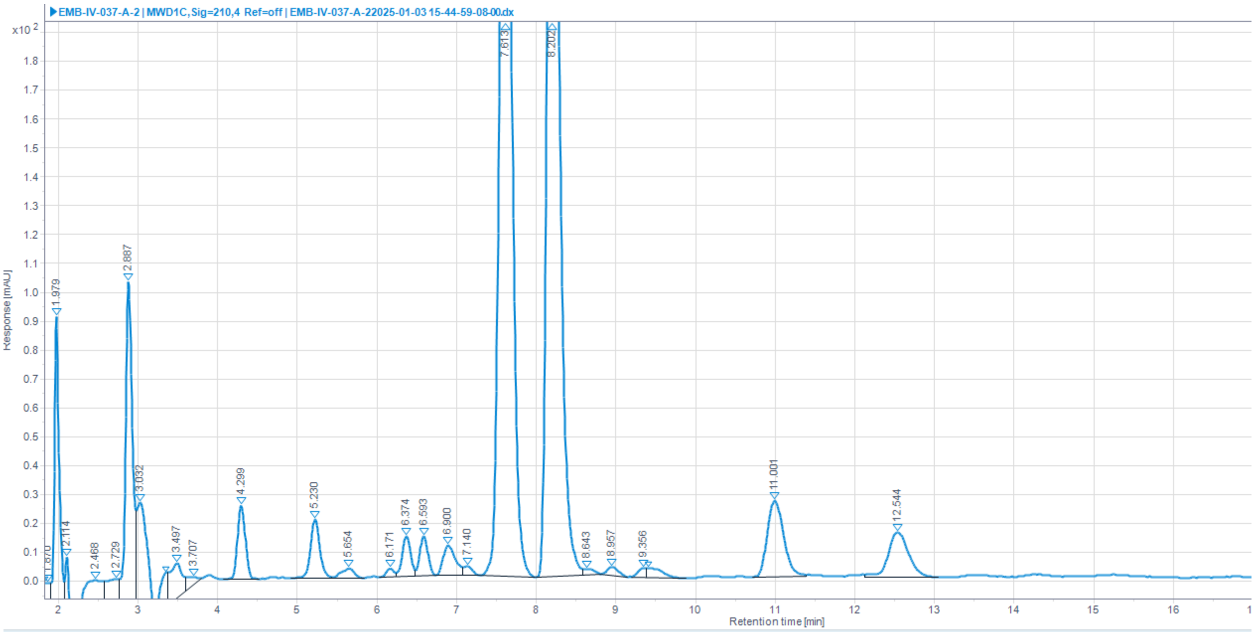

Injection Results

Peaks Summary

#	Name	Signal description	RT (min)	Area (mAU·s)	Area%	Height (mAU)	Height%	Amount	Concentration	Start time (min)	End time (min)
26		MWD1C,Sig=210,4 Ref=off	11.001	378.940	2.368	26.296	1.68			10.721	11.399
27		MWD1C,Sig=210,4 Ref=off	12.544	275.937	1.725	15.581	0.99			12.133	13.060

L65: (68% *ee*, 65% *ee*)

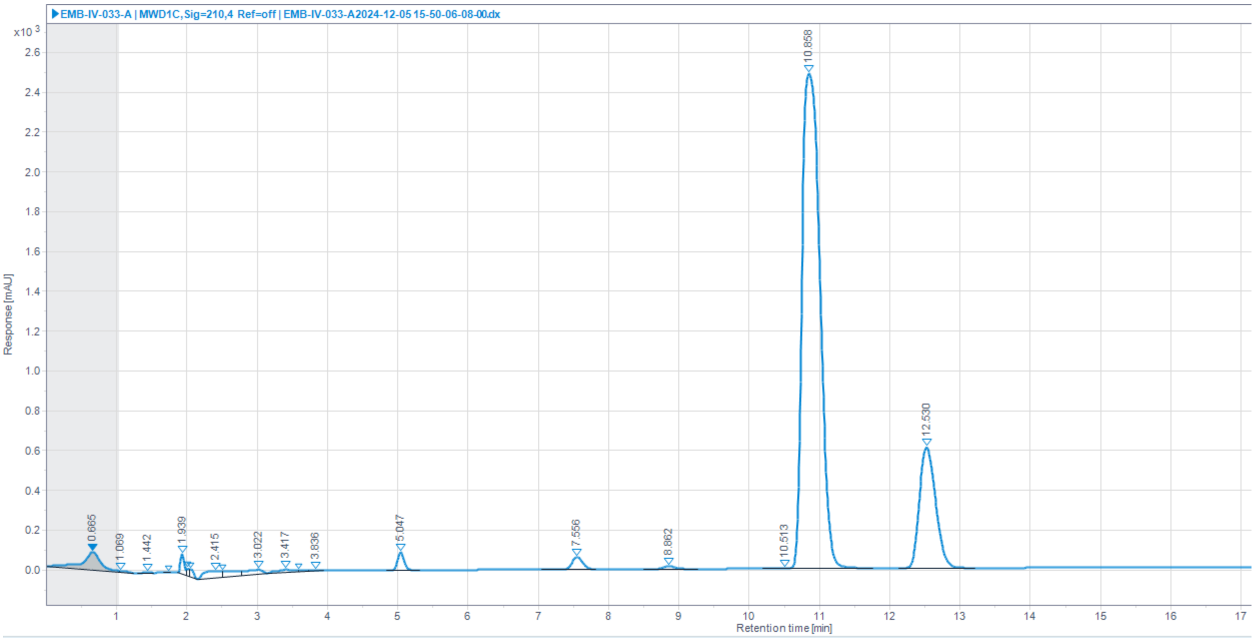

Injection Results

Peaks Summary

#	Name	Signal description	RT (min)	Area (mAU·s)	Area%	Height (mAU)	Height%	Amount	Concentration	Start time (min)	End time (min)
18		MWD1C,Sig=210,4 Ref=off	10.858	43705.151	68.533	2486.097	64.47			10.571	11.763
19		MWD1C,Sig=210,4 Ref=off	12.530	9745.494	15.282	605.380	15.70			12.129	13.214

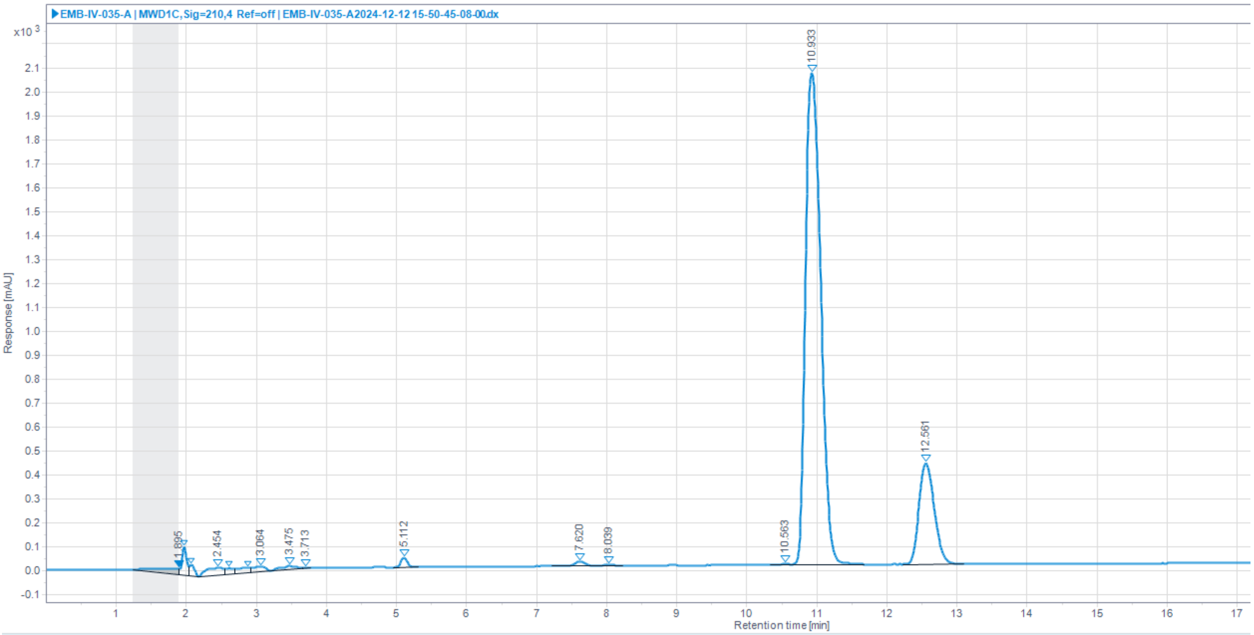

Injection Results

Peaks Summary

#	Name	Signal description	RT (min)	Area (mAU·s)	Area%	Height (mAU)	Height%	Amount	Concentration	Start time (min)	End time (min)
14		MWD1C,Sig=210,4 Ref=off	10.933	31024.626	69.211	2052.076	67.91			10.653	11.685
15		MWD1C,Sig=210,4 Ref=off	12.561	6437.782	14.362	421.390	13.95			12.229	13.106

L66: (91% *ee*, 89% *ee*)

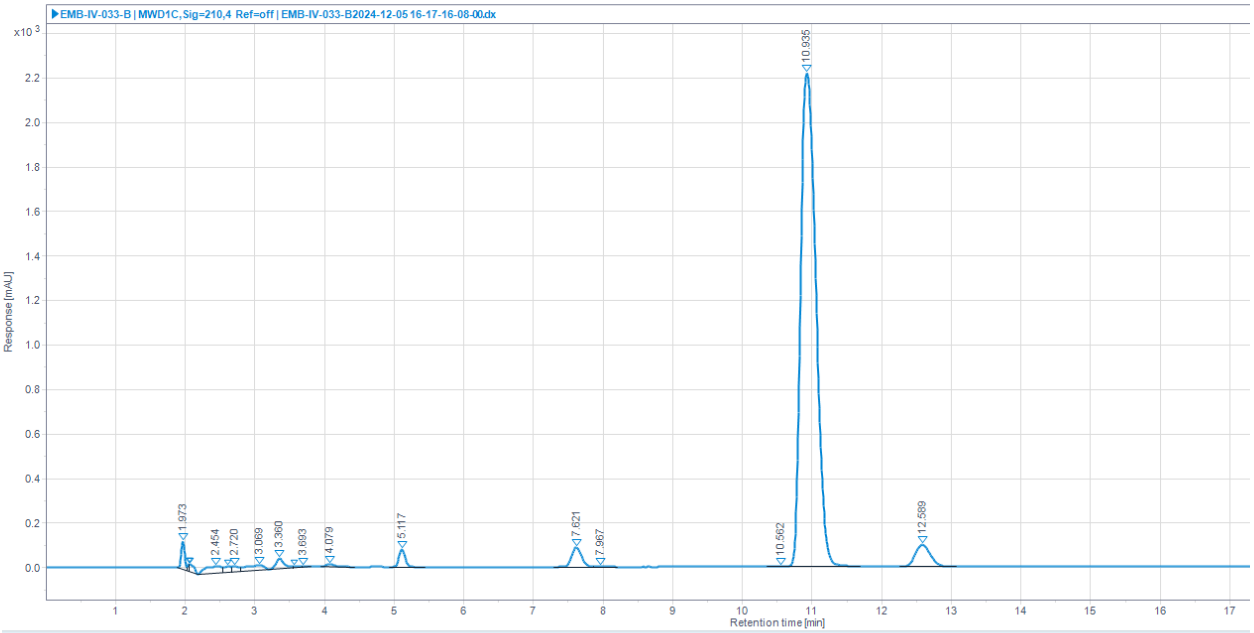

Injection Results

Peaks Summary

#	Name	Signal description	RT (min)	Area (mAU·s)	Area%	Height (mAU)	Height%	Amount	Concentration	Start time (min)	End time (min)
16		MWD1C,Sig=210,4 Ref=off	10.935	33495.709	77.811	2217.086	73.32			10.657	11.699
17		MWD1C,Sig=210,4 Ref=off	12.589	1501.426	3.488	98.388	3.25			12.274	13.077

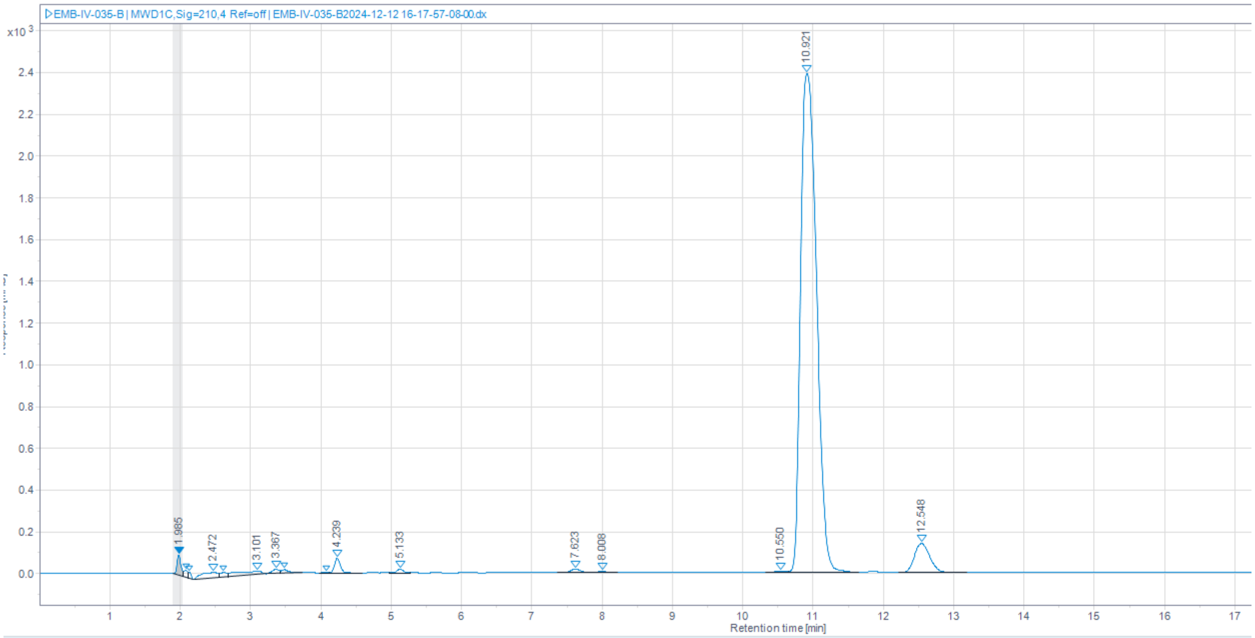

Injection Results

Peaks Summary

#	Name	Signal description	RT (min)	Area (mAU·s)	Area%	Height (mAU)	Height%	Amount	Concentration	Start time (min)	End time (min)
15		MWD1C,Sig=210,4 Ref=off	10.921	37639.947	81.139	2391.101	73.99			10.660	11.657
16		MWD1C,Sig=210,4 Ref=off	12.548	2126.918	4.585	139.678	4.32			12.222	13.192

L68: (29% *ee*, 67% *ee*) *data excluded from model validation

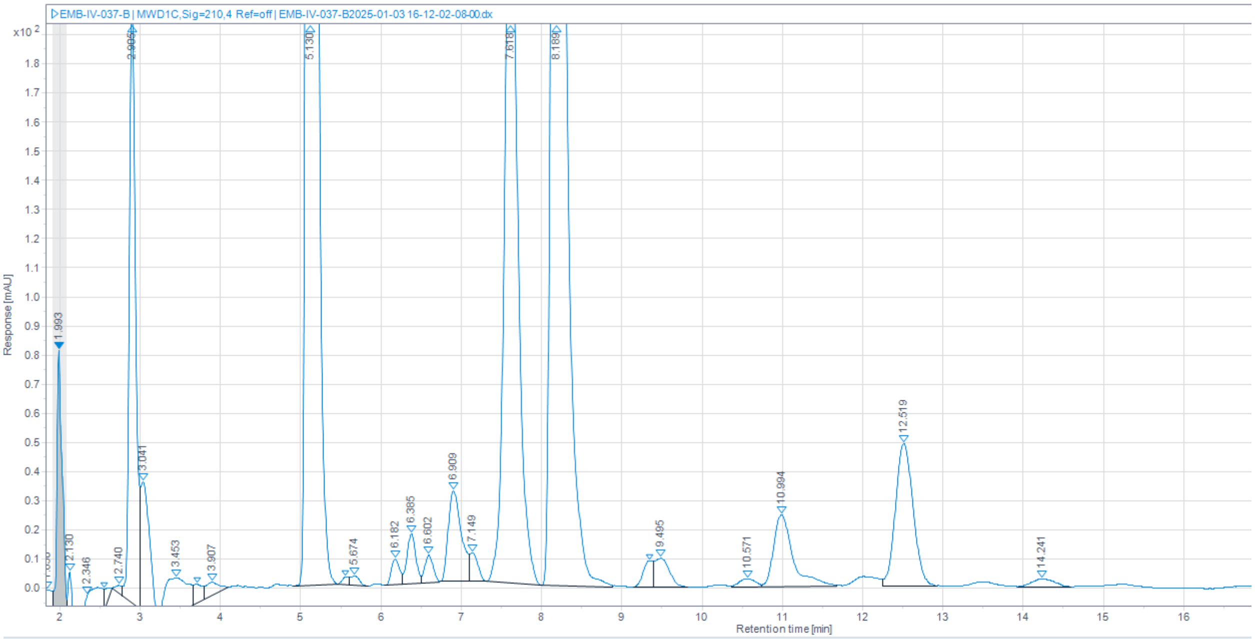

Injection Results

Peaks Summary

#	Name	Signal description	RT (min)	Area (mAU·s)	Area%	Height (mAU)	Height%	Amount	Concentration	Start time (min)	End time (min)
27		MWD1C,Sig=210,4 Ref=off	10.994	408.408	1.276	24.538	0.69			10.752	11.688
28		MWD1C,Sig=210,4 Ref=off	12.519	746.295	2.331	48.840	1.38			12.254	12.926

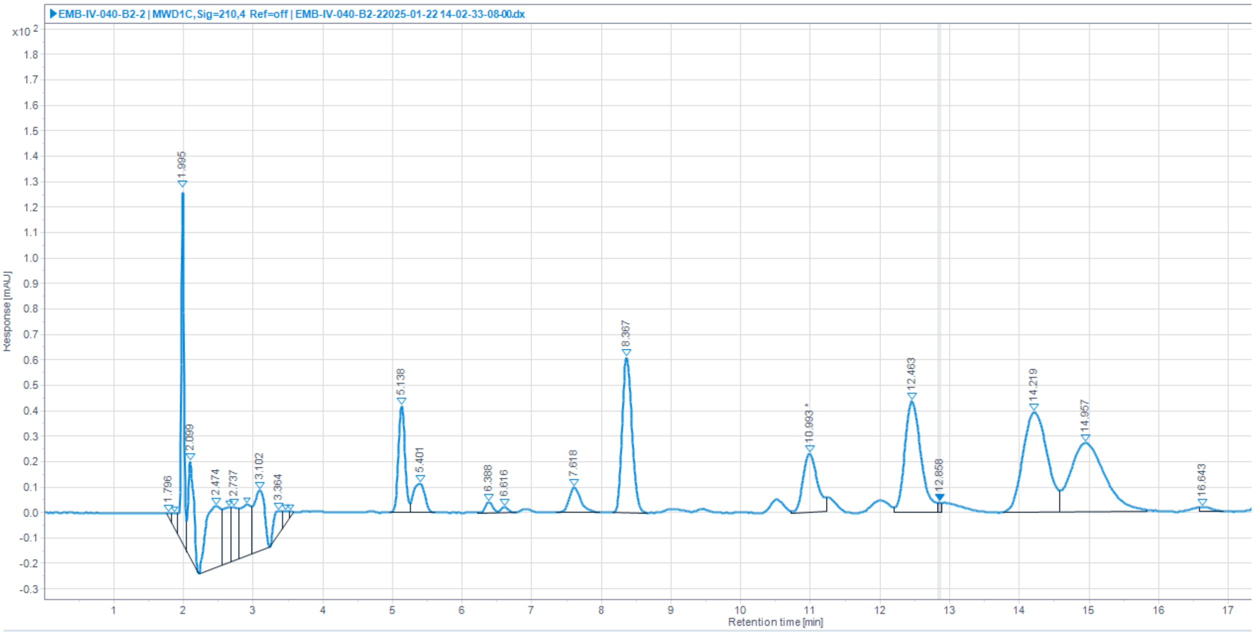

Injection Results

Peaks Summary

#	Name	Signal description	RT (min)	Area (mAU·s)	Area%	Height (mAU)	Height%	Amount	Concentration	Start time (min)	End time (min)
19		MWD1C,Sig=210,4 Ref=off	10.993	328.539	3.108	22.974	2.45			10.735	11.235
20		MWD1C,Sig=210,4 Ref=off	12.463	709.641	6.713	43.664	4.65			12.210	12.837

Reaction **G**:

L1: (21% *ee*, 21% *ee*)

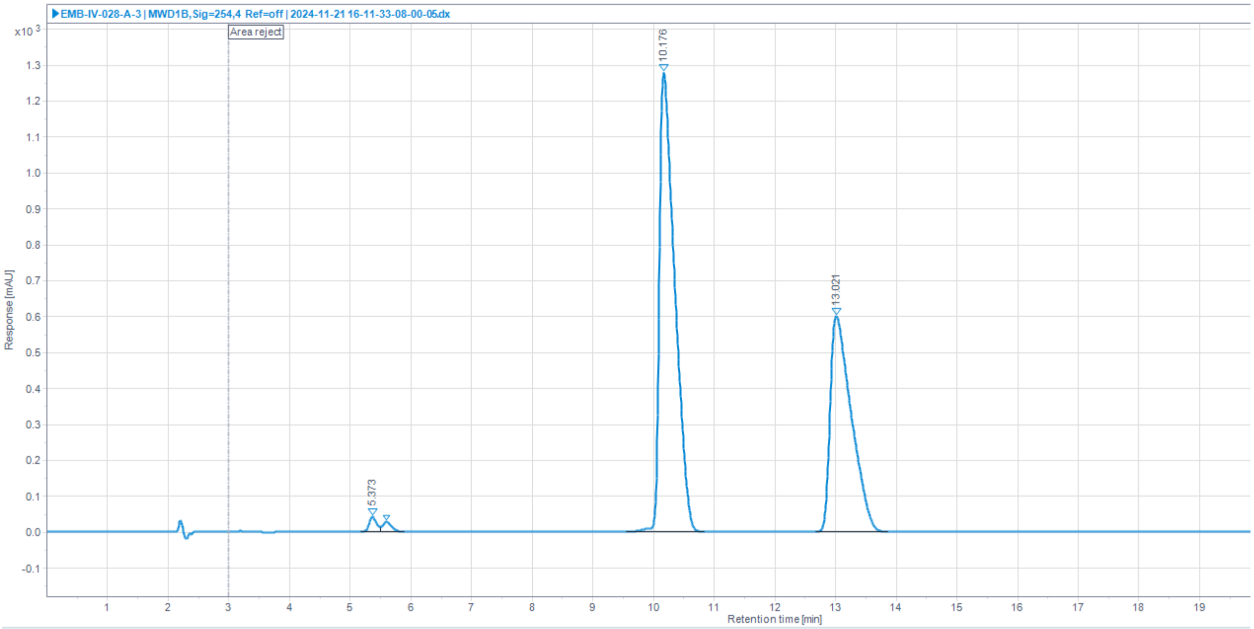

Injection Results

Peaks | Summary

#	Name	Signal description	RT (min)	Area (mAU·s)	Area%	Height (mAU)	Height%	Amount	Concentration	Start time (min)	End time (min)
3		MWD1B,Sig=254,4 Ref=off	10.176	22315.703	51.054	1278.032	55.07			9.561	10.850
4		MWD1B,Sig=254,4 Ref=off	13.021	14515.025	33.207	600.937	25.89			12.675	13.862

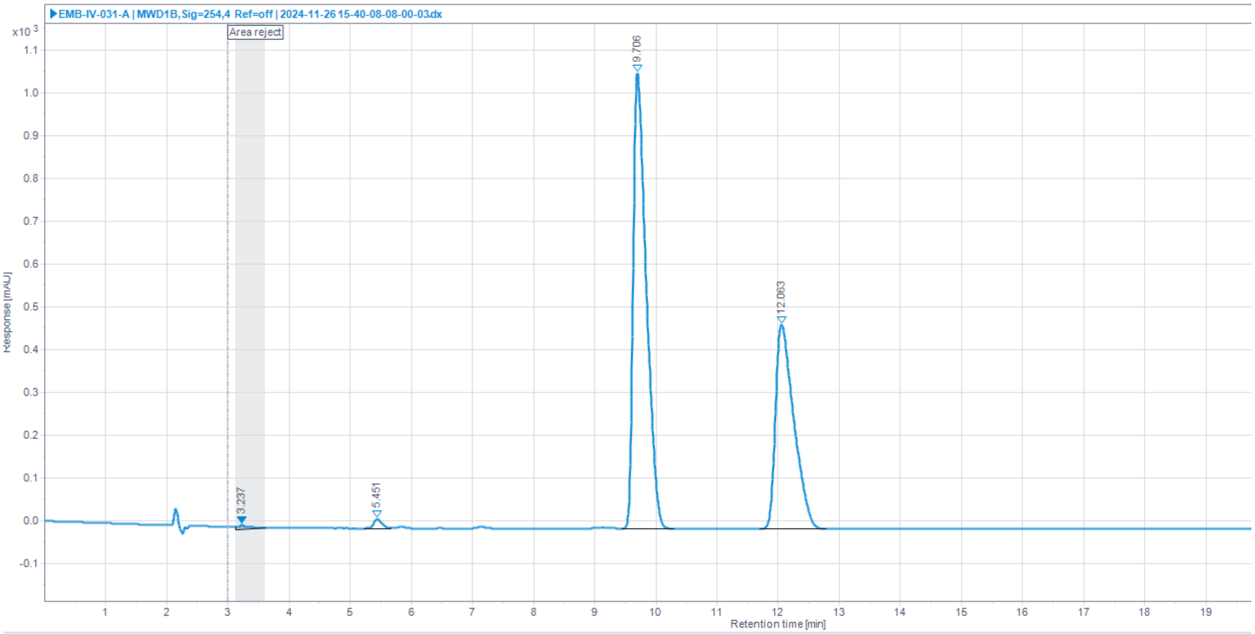

Injection Results

Peaks | Summary

#	Name	Signal description	RT (min)	Area (mAU·s)	Area%	Height (mAU)	Height%	Amount	Concentration	Start time (min)	End time (min)
3		MWD1B,Sig=254,4 Ref=off	9.706	15751.331	42.750	1066.513	44.93			9.453	10.312
4		MWD1B,Sig=254,4 Ref=off	12.063	9973.729	27.069	477.898	20.13			11.703	12.792

L3: (59% *ee*, 59% *ee*)

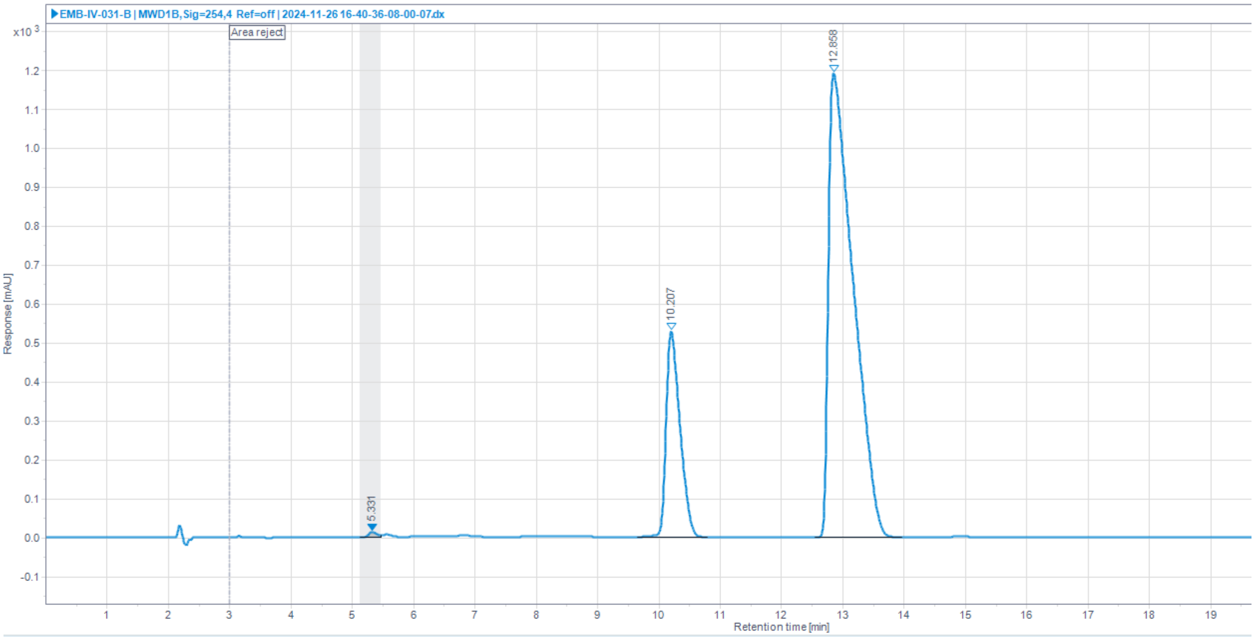

Injection Results

Peaks | Summary

#	Name	Signal description	RT (min)	Area (mAU·s)	Area%	Height (mAU)	Height%	Amount	Concentration	Start time (min)	End time (min)
2		MWD1B,Sig=254,4 Ref=off	10.207	8332.788	17.799	528.173	25.66			9.655	10.804
3		MWD1B,Sig=254,4 Ref=off	12.858	33137.797	70.782	1191.359	57.87			12.561	13.970

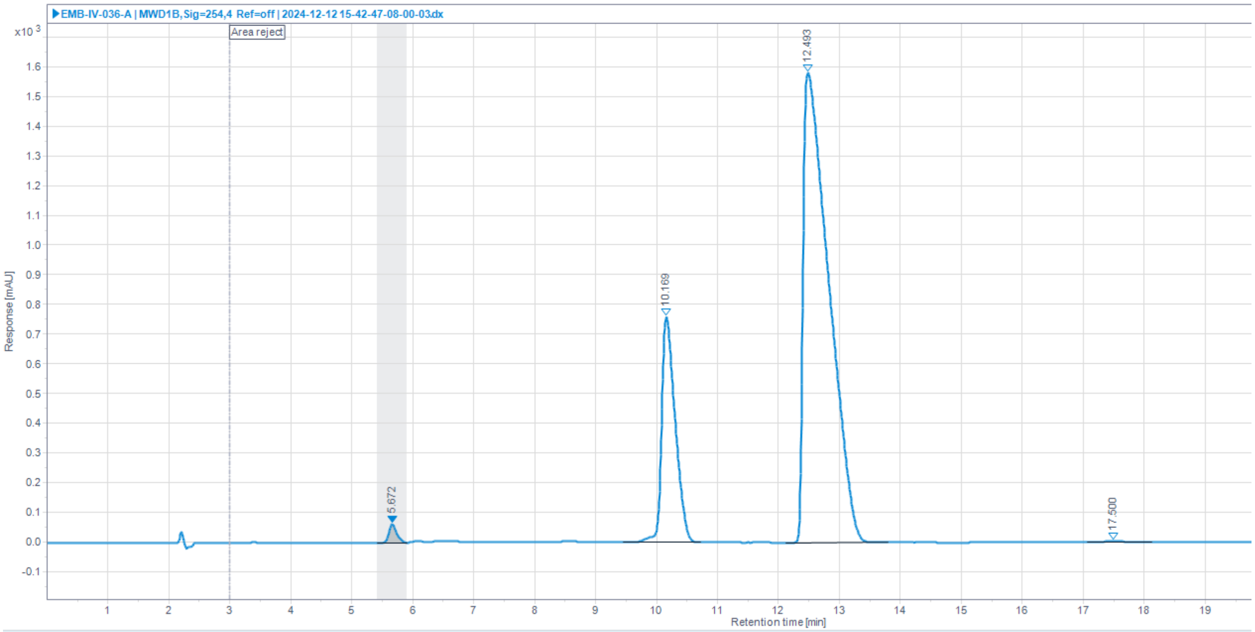

Injection Results

Peaks | Summary

#	Name	Signal description	RT (min)	Area (mAU·s)	Area%	Height (mAU)	Height%	Amount	Concentration	Start time (min)	End time (min)
2		MWD1B,Sig=254,4 Ref=off	10.169	11798.431	18.241	759.308	26.58			9.457	10.738
3		MWD1B,Sig=254,4 Ref=off	12.493	45871.128	70.919	1582.324	55.40			12.138	13.799

L11: (38% *ee*, 32% *ee*)

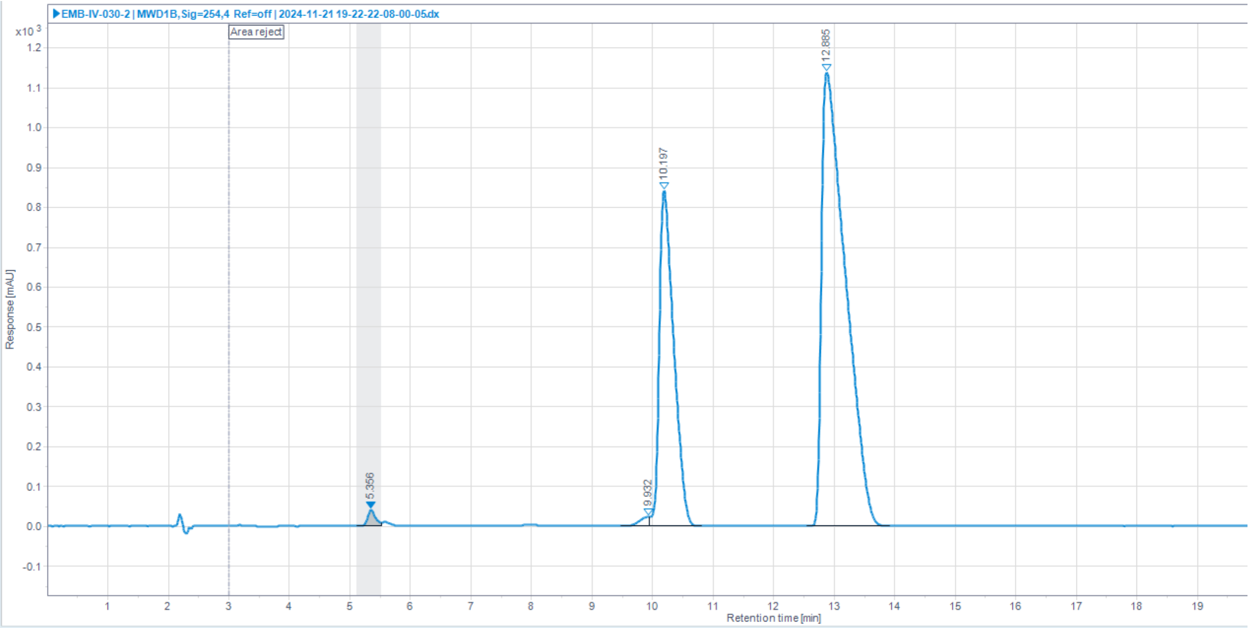

Injection Results

Peaks Summary

#	Name	Signal description	RT (min)	Area (mAU·s)	Area%	Height (mAU)	Height%	Amount	Concentration	Start time (min)	End time (min)
3		MWD1B,Sig=254,4 Ref=off	10.197	13904.746	27.191	841.041	35.05			9.961	10.812
4		MWD1B,Sig=254,4 Ref=off	12.885	31209.172	61.031	1137.176	47.40			12.562	13.929

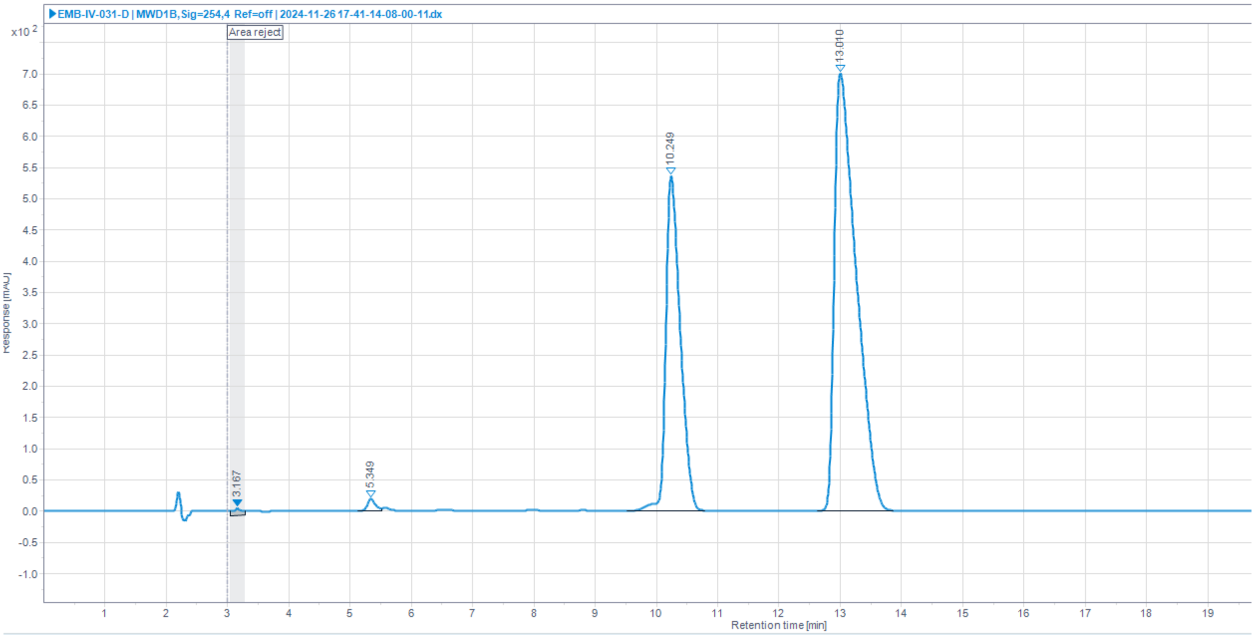

Injection Results

Peaks Summary

#	Name	Signal description	RT (min)	Area (mAU·s)	Area%	Height (mAU)	Height%	Amount	Concentration	Start time (min)	End time (min)
3		MWD1B,Sig=254,4 Ref=off	10.249	8794.200	27.233	535.450	30.55			9.533	10.803
4		MWD1B,Sig=254,4 Ref=off	13.010	17292.481	53.549	699.942	39.93			12.632	13.874

L14: (28% *ee*, 34% *ee*)

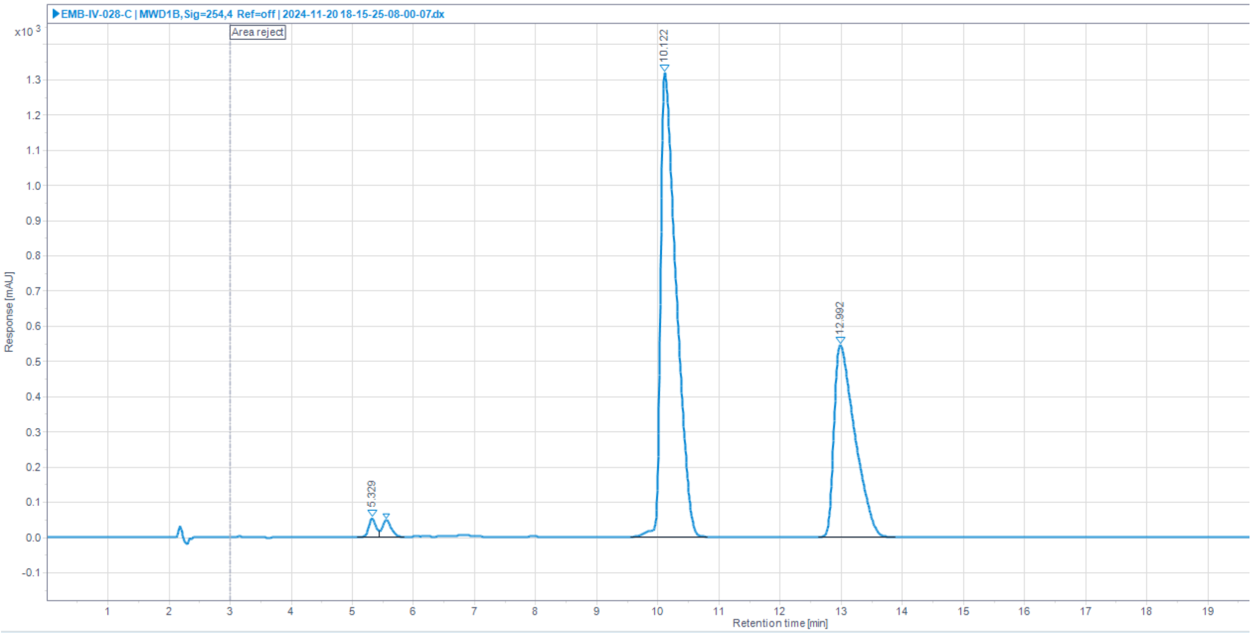

Injection Results

Peaks | Summary

#	Name	Signal description	RT (min)	Area (mAU·s)	Area%	Height (mAU)	Height%	Amount	Concentration	Start time (min)	End time (min)
3		MWD1B,Sig=254,4 Ref=off	10.122	23258.320	53.683	1318.223	54.44			9.559	10.811
4		MWD1B,Sig=254,4 Ref=off	12.992	12919.541	29.820	545.178	22.51			12.633	13.892

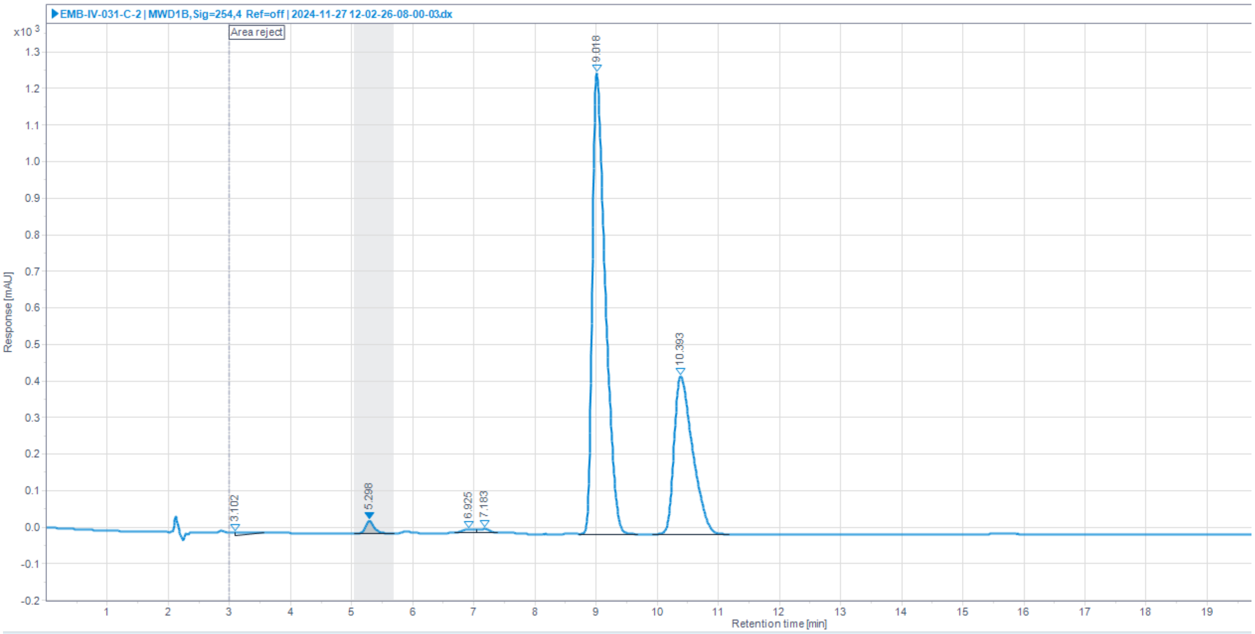

Injection Results

Peaks | Summary

#	Name	Signal description	RT (min)	Area (mAU·s)	Area%	Height (mAU)	Height%	Amount	Concentration	Start time (min)	End time (min)
5		MWD1B,Sig=254,4 Ref=off	9.018	18587.575	48.487	1262.278	55.76			8.730	9.695
6		MWD1B,Sig=254,4 Ref=off	10.393	9166.680	23.912	433.257	19.14			9.938	11.181

*slightly early retention times are due to data being collected directly after an SFC method using MeOH as the mobile phase.

L63: (75% *ee*, 80% *ee*, 77% *ee*)

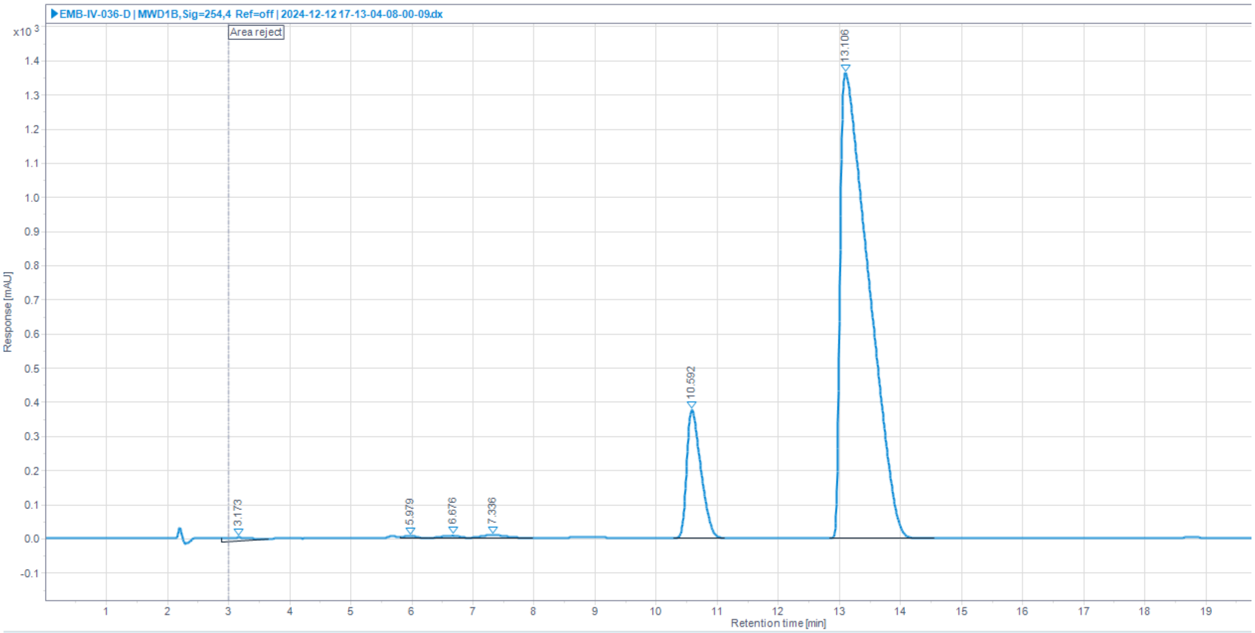

Injection Results

Peaks | Summary

#	Name	Signal description	RT (min)	Area (mAU·s)	Area%	Height (mAU)	Height%	Amount	Concentration	Start time (min)	End time (min)
5		MWD1B,Sig=254,4 Ref=off	10.592	6017.188	10.324	375.028	16.64			10.287	11.122
6		MWD1B,Sig=254,4 Ref=off	13.106	43835.543	75.210	1362.808	60.45			12.847	14.562

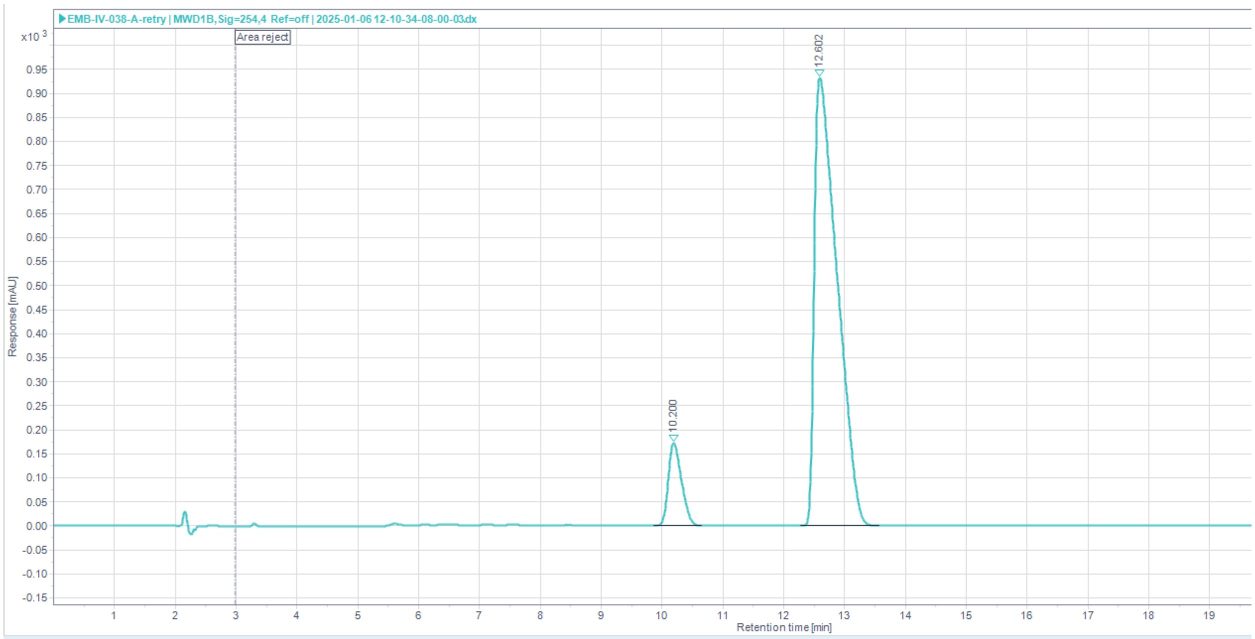

Injection Results

Peaks | Summary

#	Name	Signal description	RT (min)	Area (mAU·s)	Area%	Height (mAU)	Height%	Amount	Concentration	Start time (min)	End time (min)
1		MWD1B,Sig=254,4 Ref=off	10.200	2597.635	6.950	172.111	10.91			9.874	10.653
2		MWD1B,Sig=254,4 Ref=off	12.602	24149.776	64.608	932.247	59.12			12.286	13.576

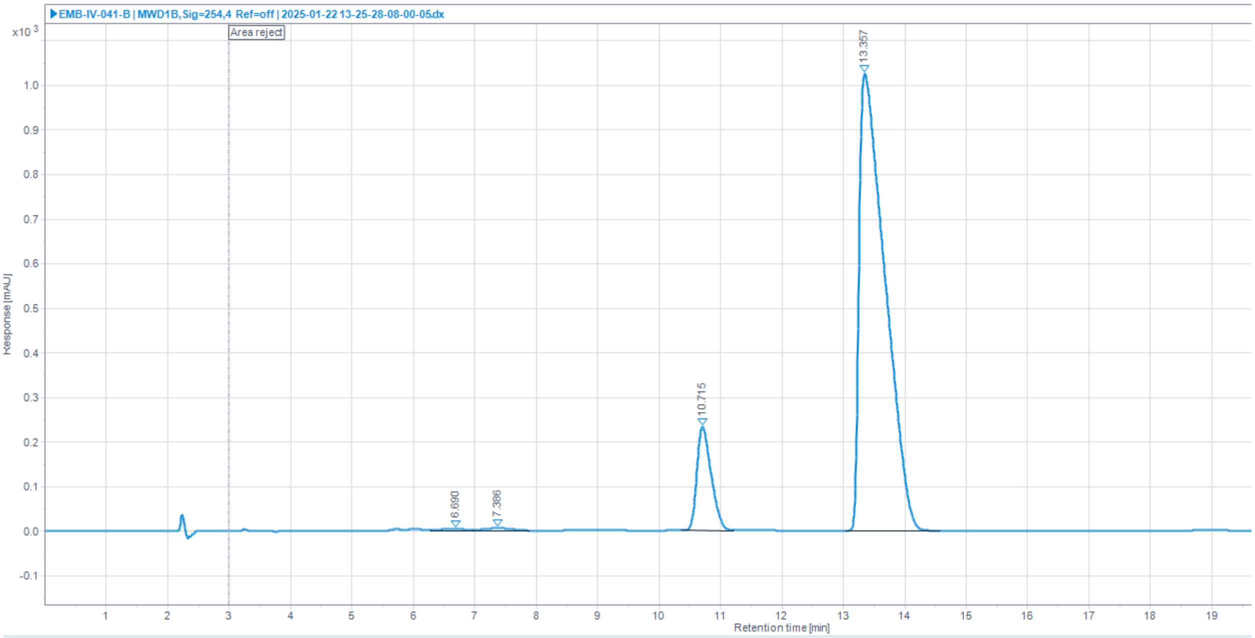

Injection Results

Peaks | Summary

#	Name	Signal description	RT (min)	Area (mAU·s)	Area%	Height (mAU)	Height%	Amount	Concentration	Start time (min)	End time (min)
3		MWD1B,Sig=254,4 Ref=off	10.715	3777.064	9.276	232.246	12.88			10.376	11.225
4		MWD1B,Sig=254,4 Ref=off	13.357	29689.415	72.912	1025.302	56.85			13.034	14.582

L65: (47% *ee*, 55% *ee*)

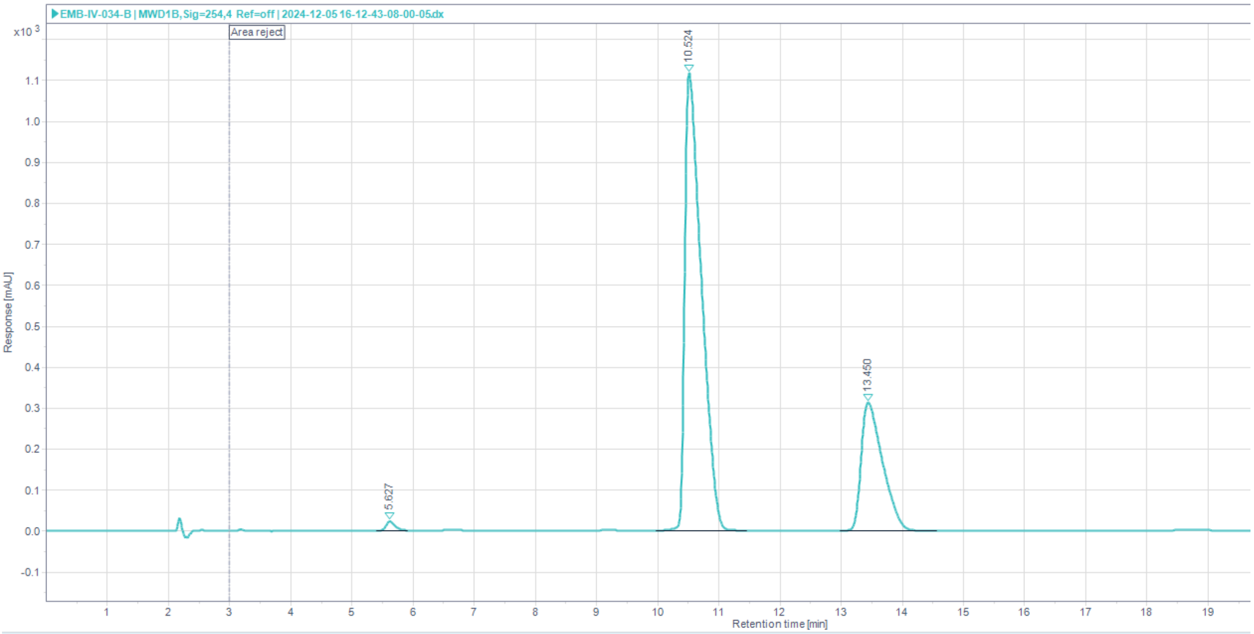

Injection Results

Peaks | Summary

#	Name	Signal description	RT (min)	Area (mAU·s)	Area%	Height (mAU)	Height%	Amount	Concentration	Start time (min)	End time (min)
2		MWD1B,Sig=254,4 Ref=off	10.524	21079.163	58.872	1116.874	58.85			9.982	11.468
3		MWD1B,Sig=254,4 Ref=off	13.450	7571.226	21.146	313.219	16.51			12.997	14.562

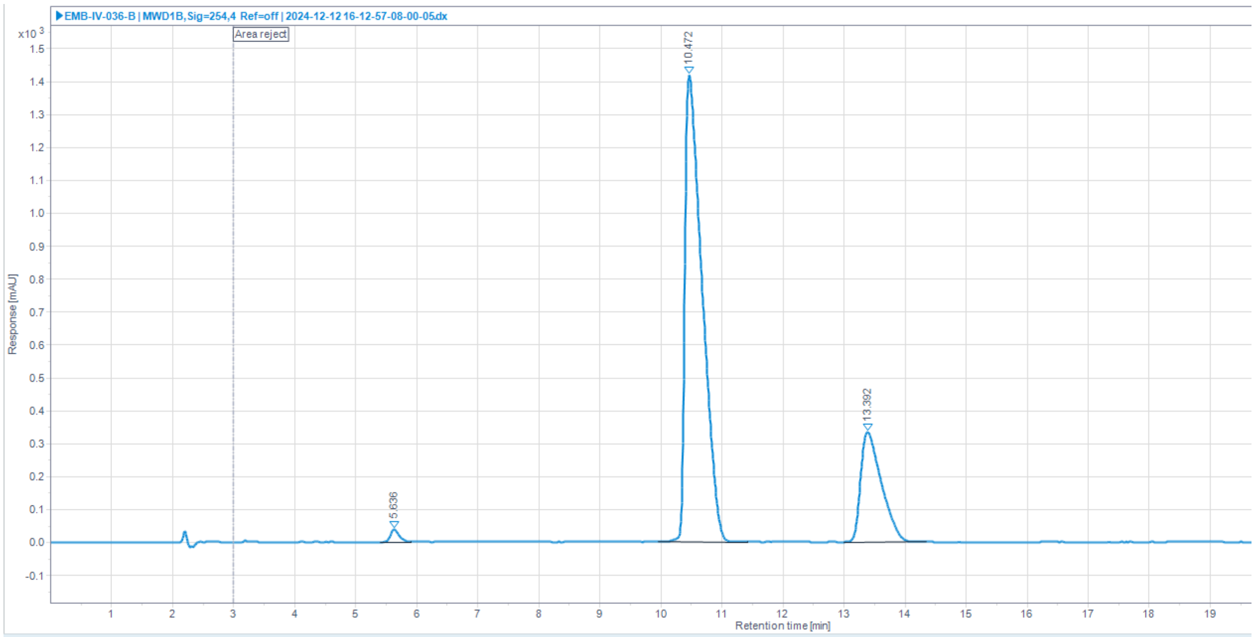

Injection Results

Peaks | Summary

#	Name	Signal description	RT (min)	Area (mAU·s)	Area%	Height (mAU)	Height%	Amount	Concentration	Start time (min)	End time (min)
2		MWD1B,Sig=254,4 Ref=off	10.472	27682.213	65.077	1418.838	62.33			9.968	11.425
3		MWD1B,Sig=254,4 Ref=off	13.392	7906.622	18.587	333.514	14.65			13.001	14.358

L66: (70% *ee*, 66% *ee*)

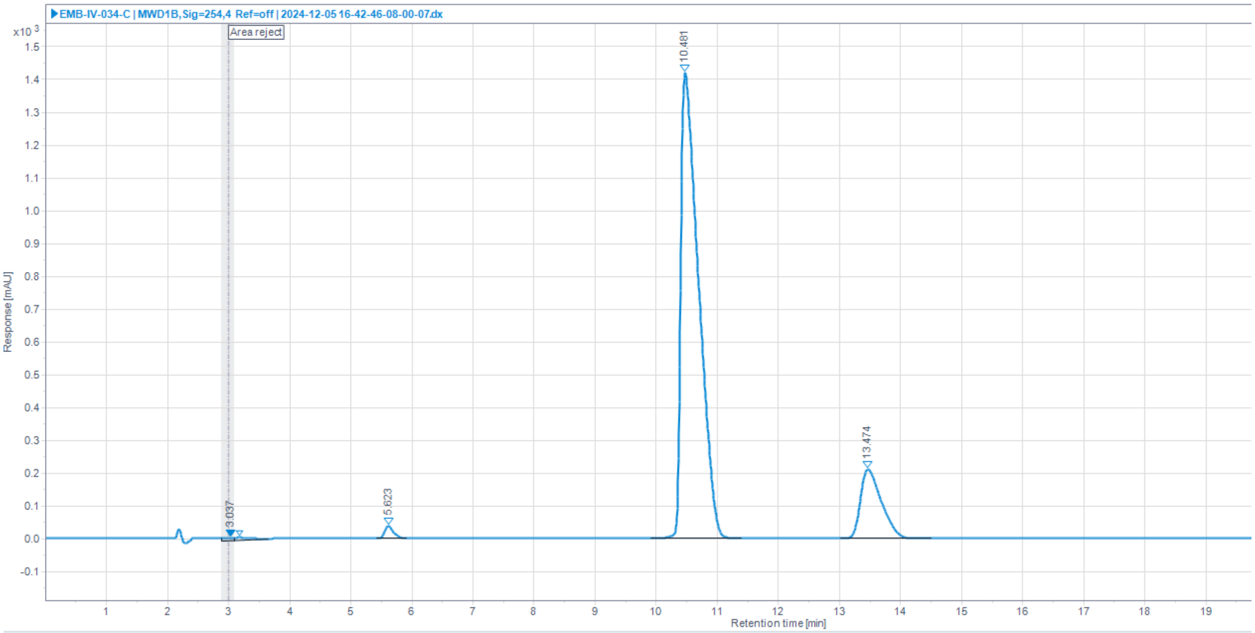

Injection Results

Peaks Summary

#	Name	Signal description	RT (min)	Area (mAU·s)	Area%	Height (mAU)	Height%	Amount	Concentration	Start time (min)	End time (min)
4		MWD1B,Sig=254,4 Ref=off	10.481	28544.428	69.121	1418.251	65.36			9.919	11.399
5		MWD1B,Sig=254,4 Ref=off	13.474	4996.968	12.100	210.430	9.70			13.019	14.500

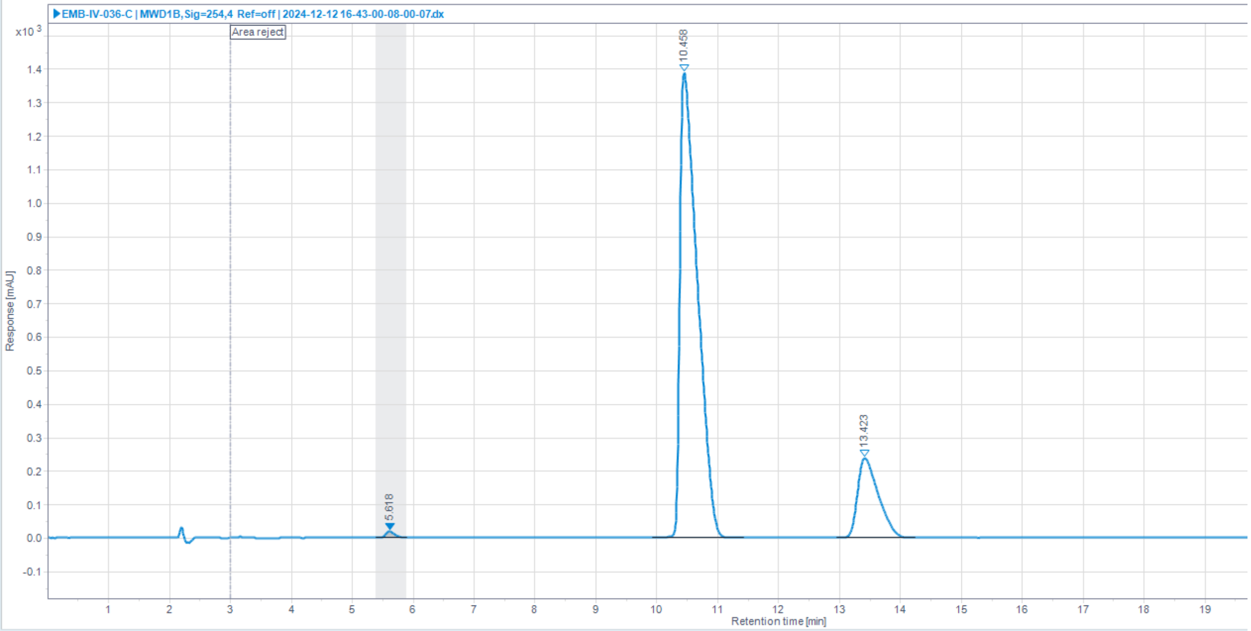

Injection Results

Peaks Summary

#	Name	Signal description	RT (min)	Area (mAU·s)	Area%	Height (mAU)	Height%	Amount	Concentration	Start time (min)	End time (min)
2		MWD1B,Sig=254,4 Ref=off	10.458	27572.355	68.959	1388.047	66.44			9.932	11.438
3		MWD1B,Sig=254,4 Ref=off	13.423	5653.097	14.138	237.687	11.38			12.965	14.244

L68: (22% *ee*, 23% *ee*)

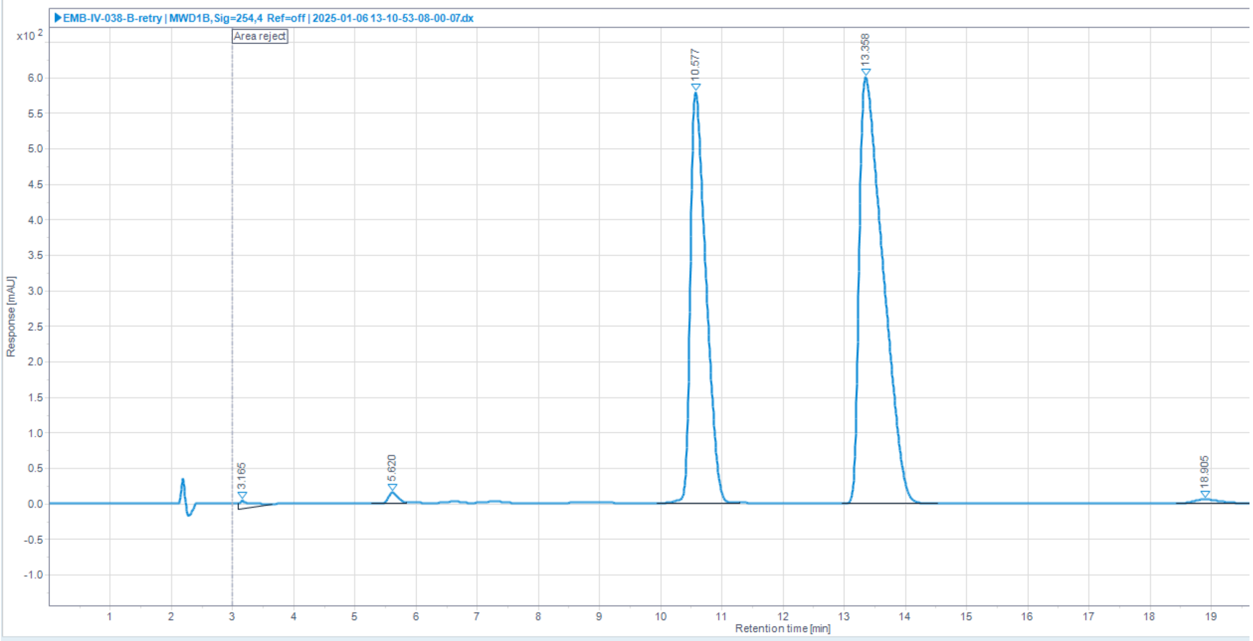

Injection Results

Peaks | Summary

#	Name	Signal description	RT (min)	Area (mAU·s)	Area%	Height (mAU)	Height%	Amount	Concentration	Start time (min)	End time (min)
3		MWD1B,Sig=254,4 Ref=off	10.577	10253.398	27.454	579.144	30.22			9.941	11.293
4		MWD1B,Sig=254,4 Ref=off	13.358	16131.681	43.193	600.102	31.31			12.977	14.534

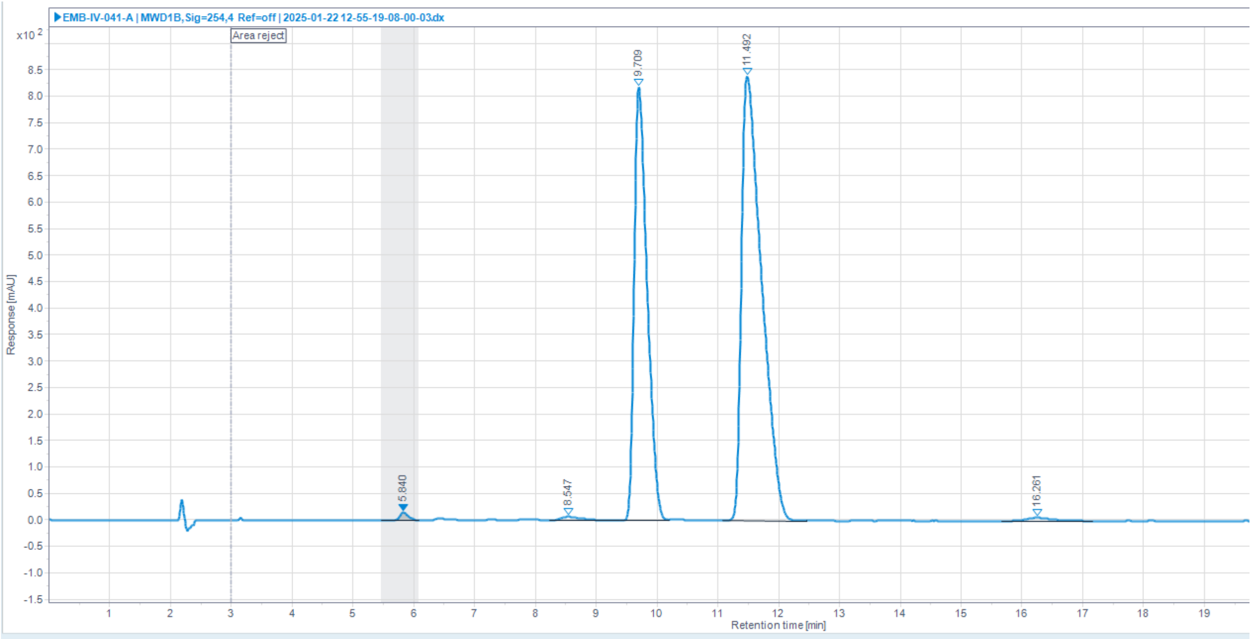

Injection Results

Peaks | Summary

#	Name	Signal description	RT (min)	Area (mAU·s)	Area%	Height (mAU)	Height%	Amount	Concentration	Start time (min)	End time (min)
3		MWD1B,Sig=254,4 Ref=off	9.709	12153.319	32.283	819.581	38.66			9.396	10.205
4		MWD1B,Sig=254,4 Ref=off	11.492	19337.524	51.367	839.586	39.60			11.092	12.471

*slightly early retention times are due to data being collected directly after an SFC method using MeOH as the mobile phase.

4.6 Modeling of Ni-catalyzed XECs (Model 2)

To limit the size of the input representation and reduce the computational cost of the exhaustive feature selection, descriptors were pre-filtered *via* the Boruta algorithm (https://github.com/SigmanGroup/python-modeling/feature_curation.ipynb, percentage of maximum importance of shadow features = 60, maximum number of iterations = 70). Only Boltzmann-weighted average values were included for electronic descriptors, whereas min, max, and average values were used for steric parameters. Combinations of 4 features were selected *via* the repeated, stratified, nested *k*-fold CV scheme with a collinearity cutoff ≥ 0.5. Each cross-validation fold (inner and outer) was stratified with respect to the nature of the C(sp^3)-electrophile (Az, 32 data points; BnCl, 14 datapoints). Based on the 5×2 CV test, 10 models were selected for ensemble predictions (Table S18 and Table S20). "Leave-one-out" analysis results are reported in Table S19.

Since yield data was not uniformly reported for all the ligand screens in reactions **D**–**H**, MLR analysis using yield as target was not attempted. Furthermore, training a MLR model of yield would be particularly challenging for case study 2, given the different conditions reactions **D**–**H** were performed in (*e.g.*, electrochemical conditions *vs*. Mn^0 reductant).

Table S18. MLR models selected for ensemble predictions. Combinations of features were selected via the repeated, stratified, nested *k*-fold CV scheme[a] and evaluated *via* the 5×2 CV test.[b]

Features	Avg. Train RMSE[a]	Avg. Test RMSE[a]	Avg. Test Adj. R^{2}[a]	Test RMSE (mean, SD)[b]
$\text{pyramidalization}_{(\text{Gavrish, C4})}^{\text{TSRC, max}}$<br>$P_{\text{int(N2)}}^{\text{TSRC, Boltz}}$<br>$\text{Buried Sterimol B1}_{(\text{N1}\rightarrow\text{Ni, 4.5 Å})}^{\text{TSRE, Boltz}}$<br>$\text{pyramidalization}_{(\text{Gavrish, N2})}^{\text{TSRE, max}}$	0.28	0.25	0.60	(0.79, 0.45)
$\text{pyramidalization}_{(\text{Gavrish, C4})}^{\text{TSRC, max}}$<br>$P_{\text{int(N2)}}^{\text{TSRC, Boltz}}$<br>$d_{(\text{Ni–C2s})}^{\text{TSRE, Boltz}}$<br>$\text{Buried Sterimol B1}_{(\text{N1}\rightarrow\text{Ni, 4.5 Å})}^{\text{TSRE, Boltz}}$	0.24	0.36	0.66	(0.51, 0.46)
$\varphi_{(\text{N1–Ni–C2s–H1s})}^{\text{TSRC, Boltz}}$<br>$\text{Buried Sterimol L}_{(\text{Ni}\rightarrow\text{C2s, 4.5 Å})}^{\text{TSRC, max}}$<br>$d_{(\text{Ni–C2s})}^{\text{TSRE, Boltz}}$<br>$\text{Buried Sterimol B1}_{(\text{N1}\rightarrow\text{Ni, 4.5 Å})}^{\text{TSRE, Boltz}}$	0.23	0.39	0.67	(0.34, 0.03)
$\text{pyramidalization}_{(\text{Gavrish, C4})}^{\text{TSRC, max}}$<br>$\text{Spin Density}_{(\text{R2})}^{\text{TSRC, Boltz}}$<br>$P_{\text{int(N2)}}^{\text{TSRC, Boltz}}$<br>$\text{Buried Sterimol B1}_{(\text{N1}\rightarrow\text{Ni, 4.5 Å})}^{\text{TSRE, Boltz}}$	0.25	0.39	0.60	(0.55, 0.39)
$P_{\text{int(N2)}}^{\text{TSRC, Boltz}}$<br>$\text{Buried Sterimol L}_{(\text{N1}\rightarrow\text{Ni, 4.5 Å})}^{\text{Int, max}}$<br>$d_{(\text{Ni–C2s})}^{\text{TSRE, Boltz}}$<br>$\text{Buried Sterimol B1}_{(\text{N1}\rightarrow\text{Ni, 4.5 Å})}^{\text{TSRE, Boltz}}$	0.24	0.40	0.58	(0.42, 0.18)
$\varphi_{(\text{N1–Ni–C2s–H1s})}^{\text{TSRC, Boltz}}$<br>$\varphi_{(\text{C4–N2–Ni–C1s})}^{\text{Int, Boltz}}$<br>$d_{(\text{Ni–C2s})}^{\text{TSRE, Boltz}}$<br>$\text{Buried Sterimol B1}_{(\text{N1}\rightarrow\text{Ni, 4.5 Å})}^{\text{TSRE, Boltz}}$	0.23	0.41	0.72	(0.32, 0.04)
$\varphi_{(\text{C4–N2–Ni–C1s})}^{\text{Int, Boltz}}$<br>$d_{(\text{Ni–C2s})}^{\text{TSRE, Boltz}}$<br>$\text{Buried Sterimol B1}_{(\text{N1}\rightarrow\text{Ni, 4.5 Å})}^{\text{TSRE, Boltz}}$<br>$\text{Buried Sterimol B5}_{(\text{C2}\rightarrow\text{R1, 4.5 Å})}^{\text{TSRE, Boltz}}$	0.24	0.41	0.62	(0.33, 0.05)
$P_{\text{int(N2)}}^{\text{TSRC, Boltz}}$<br>$\varphi_{(\text{C4–N2–Ni–C1s})}^{\text{Int, Boltz}}$<br>$\text{Buried Sterimol B5}_{(\text{C2}\rightarrow\text{R1, 4.5 Å})}^{\text{TSRE, Boltz}}$<br>$\text{Hirsh atom dipole}_{(\text{C5})}^{\text{TSRE, Boltz}}$	0.23	0.45	0.63	(0.49, 0.28)
$\varphi_{(\text{N1–Ni–C2s–H1s})}^{\text{TSRC, Boltz}}$<br>$\%V_{\text{bur, no H 3.5 Å (C2s)}}^{\text{Int, max}}$<br>$d_{(\text{Ni–C2s})}^{\text{TSRE, Boltz}}$<br>$\text{Buried Sterimol B1}_{(\text{N1}\rightarrow\text{Ni, 4.5 Å})}^{\text{TSRE, Boltz}}$	0.25	0.46	0.64	(0.36, 0.05)
$P_{\text{int(N2)}}^{\text{TSRC, Boltz}}$<br>$\varphi_{(\text{C4–N2–Ni–C1s})}^{\text{Int, Boltz}}$<br>$d_{(\text{N1–Ni})}^{\text{TSRE, min}}$<br>$\text{pyramidalization}_{(\text{Gavrish, N2})}^{\text{TSRE, max}}$	0.25	0.48	0.55	(0.91, 0.50)

Table S19. Leave-one-out analysis according to reaction type (**D**–**G**), electrophile type (C(sp^3)- and C(sp^2)-electrophiles), and ligand class. R^2 and MAE values are reported for the test set.

	Train	Data points	Test	Data points	R^2	MAE
**Reaction**	E, F, G	25	D	21	0.75	0.22
	D, F, G	35	E	11	0.78	0.18
	D, E, G	40	F	6	0.25	0.27
	D, E, F	38	G	8	0.34	0.30
**C(sp^3)**	BnCl	14	Az	32	0.51	0.24
	Az	32	BnCl	14	0.32	0.26
**C(sp^2)**	AlkenylBr	19	ArI	27	0.66	0.21
	ArI	27	AlkenylBr	19	0.30	0.32
**Ligand**	BiIm, BOX, PyOx	28	BiOx	18	0.74	0.17
	BiOx, BOX, PyOx	33	BiIm	13	0.75	0.19
	BiOx, BiIm, PyOx	32	BOX	14	0.17	0.34
	BiOx, BiIm, BOX	45	PyOx	1	n/a	0.15

Table S20. Measured and predicted $\Delta\Delta G^{\ddagger}$ values using an ensemble of MLR models (see Table S18).

Structure	Ligand	Reaction	*ee*	Measured $\Delta\Delta G^{\ddagger}$	Predicted $\Delta\Delta G^{\ddagger}$	SD
L1_Milo	BiOx	D	48	0.62	0.63	0.07
L2_Milo	BiOx	D	63	0.87	0.89	0.16
L3_Milo	BiOx	D	82	1.37	1.34	0.12
L6_Milo	BiIm	D	76	1.19	1.02	0.12
L8_Milo	BiIm	D	65	0.91	1.03	0.10
L11_Milo	BiOx	D	45	0.57	0.73	0.09
L12_Milo	BiOx	D	63	0.88	0.74	0.11
L14_Milo	BiOx	D	71	1.04	1.07	0.11
L15_Milo	BiOx	D	51	0.67	0.67	0.13
L30_Milo	BiIm	D	36	0.44	0.59	0.09
L31_Milo	BiIm	D	78	1.25	1.42	0.22
L32_Milo	BiIm	D	89	1.70	1.24	0.14
L33_Milo	BiIm	D	83	1.39	1.02	0.06
L34_Milo	BiIm	D	78	1.24	1.14	0.12
L35_Milo	BiOx	D	8	0.10	0.34	0.15
L36_Milo	BiOx	D	48	0.61	0.59	0.10
L37_Milo	BiOx	D	26	0.32	0.60	0.17
L38_Milo	BiOx	D	82	1.37	1.10	0.16
L39_Milo	BiIm	D	61	0.84	0.82	0.06
L40_Milo	BiIm	D	75	1.14	0.94	0.12
L41_Milo	BiIm	D	27	0.33	0.53	0.14
L12_Nevado	BiOx	E	68	0.98	0.70	0.11
L31_Nevado	BiIm	E	80	1.30	1.24	0.13
L57_Nevado	BOX	E	68	0.98	0.88	0.12
L58_Nevado	BOX	E	46	0.59	0.83	0.08
L60_Nevado	BOX	E	92	1.88	1.43	0.42
L63_Nevado	BOX	E	78	1.24	0.90	0.13
L64_Nevado	PyOx	E	12	0.14	0.48	0.50
L65_Nevado	BiIm	E	70	1.03	1.17	0.24
L66_Nevado	BiIm	E	86	1.53	1.30	0.27
L67_Nevado	BOX	E	70	1.03	1.18	0.15
L68_Nevado	BOX	E	54	0.72	1.07	0.28
L1_Reisman_2017	BiOx	F	60	0.82	0.94	0.19
L2_Reisman_2017	BiOx	F	68	0.98	0.83	0.13
L3_Reisman_2017	BiOx	F	90	1.74	1.36	0.14
L14_Reisman_2017	BiOx	F	75	1.15	1.08	0.14
L42_Reisman_2017	BiOx	F	80	1.30	1.34	0.10
L49_Reisman_2017	BiOx	F	86	1.53	1.20	0.10
L55_Reisman_2014	BOX	G	40	0.49	0.76	0.18
L56_Reisman_2014	BOX	G	57	0.75	0.93	0.23
L57_Reisman_2014	BOX	G	68	0.97	0.76	0.06
L58_Reisman_2014	BOX	G	70	1.01	0.69	0.19
L59_Reisman_2014	BOX	G	49	0.62	1.01	0.27
L60_Reisman_2014	BOX	G	87	1.55	1.25	0.13
L61_Reisman_2014	BOX	G	27	0.32	0.66	0.08
L62_Reisman_2014	BOX	G	78	1.22	1.24	0.11

L65_Milo	BiIm	OOS (D)	67	0.94	1.40	0.44
L66_Milo	BiIm	OOS (D)	90	1.73	1.25	0.30
L1_Reisman_2014	BiOx	OOS (D)	22	0.26	0.66	0.18
L3_Reisman_2014	BiOx	OOS (G)	59	0.79	0.85	0.17
L11_Reisman_2014	BiOx	OOS (G)	35	0.43	0.61	0.15
L14_Reisman_2014	BiOx	OOS (G)	31	0.38	0.99	0.16
L63_Reisman_2014	BOX	OOS (G)	77	1.21	1.15	0.11
L65_Reisman_2014	BiIm	OOS (G)	51	0.66	0.85	0.17
L66_Reisman_2014	BiIm	OOS (G)	68	0.97	1.03	0.12
L68_Reisman_2014	BOX	OOS (G)	23	0.27	0.72	0.14
L1_Liu	BiOx	OOS (H)	39	0.45	0.56	0.25
L79_Liu	PyOx	OOS (H)	60	0.82	0.50	0.40
L90_Liu	PyOx	OOS (H)	63	0.88	0.61	0.20
L91_Liu	PyOx	OOS (H)	65	0.92	0.98	0.48
L104_Liu	PyOx	OOS (H)	58	0.78	0.80	0.83
L107_Liu	BOX	OOS (H)	68	0.90	0.99	0.19
L113_Liu	PyOx	OOS (H)	54	0.72	1.05	0.42
L114_Liu	PyOx	OOS (H)	62	0.86	0.88	0.35
L115_Liu	PyOx	OOS (H)	61	0.84	0.79	0.27
L116_Liu	PyOx	OOS (H)	41	0.52	0.57	0.21
L117_Liu	PyOx	OOS (H)	66	0.94	0.39	0.44
L118_Liu	PyIm	OOS (H)	74	1.13	0.78	0.27
L119_Liu	PyIm	OOS (H)	71	1.05	0.89	0.22
L120_Liu	PyIm	OOS (H)	74	1.13	0.79	0.33
L121_Liu	PyIm	OOS (H)	74	1.13	1.13	0.27
L122_Liu	PyIm	OOS (H)	89	1.68	1.94	0.31
L123_Liu	PyIm	OOS (H)	80	1.30	1.45	0.34

4.7 Feature analysis

Table S21 reports the features that appear in the ensemble models (model 1 for reactions **A**–**C**, model 2 for reactions **D**–**G**, see Table S13 and Table S18) and how many times they occur in the distinct MLR models.

Table S21. Frequency of features in ensemble models 1 (C–H activation dataset, reactions **A**–**C**) and 2 (Ni-catalyzed XECs, reactions **D**–**G**).

**C(*sp*^{3})–H activation**		**Ni-catalyzed XECs**	
**Feature**	**Frequency**	**Feature**	**Frequency**
$\theta_{(N1–Ni–N2)}^{TSRC, max}$	8	Buried Sterimol $B1_{(N1\rightarrow Ni, 4.5 Å)}^{TSRE, Boltz}$	8
Buried Sterimol $B5_{(N1\rightarrow Ni, 4.5 Å)}^{TSRC, min}$	4	$d_{(Ni–C2s)}^{TSRE, Boltz}$	6
Buried Sterimol $B1_{(Ni\rightarrow C2s, 4.5 Å)}^{TSRE, max}$	4	$P_{int (N2)}^{TSRC, Boltz}$	6
$\%V_{bur, no H 3.5 Å (N2)}^{TSRE, Boltz}$	3	$\varphi_{(C4–N2–Ni–C1s)}^{Int, Boltz}$	4
$d_{(C1s–C2s)}^{TSRC, min}$	3	$\varphi_{(N1–Ni–C2s–H1s)}^{TSRC, Boltz}$	3
$d_{(Br–Ni)}^{Int, max}$	3	$pyramidalization_{(Gavrish, C4)}^{TSRC, max}$	3
Sterimol $B5_{(C2\rightarrow R1)}^{TSRC, max}$	2	$pyramidalization_{(Gavrish, N2)}^{TSRE, max}$	2
Sterimol $B5_{(C5\rightarrow R2)}^{Int, min}$	2	Buried Sterimol $B5_{(C2\rightarrow R1, 4.5 Å)}^{TSRE, Boltz}$	2
$\varphi_{(N2–Ni–C2s–H1s)}^{Int, max}$	2	Spin $Density_{(R2)}^{TSRC, Boltz}$	1
$\theta_{(N1–Ni–C1s)}^{Int, max}$	2	Buried Sterimol $L_{(Ni\rightarrow C2s, 4.5 Å)}^{TSRC, max}$	1
$\%V_{bur 4.0 Å (Br)}^{Int, Boltz}$	1	Buried Sterimol $L_{(N1\rightarrow Ni, 4.5 Å)}^{Int, max}$	1
$\varphi_{(C4–N2–Ni–C1s)}^{TSRE, Boltz}$	1	Hirsh atom $dipole_{(C5)}^{TSRE, Boltz}$	1
Sterimol $L_{(N1\rightarrow Ni)}^{TSRC, max}$	1	$\%V_{bur, no H 3.5 Å (C2s)}^{Int, max}$	1
Buried Sterimol $L_{(C5\rightarrow R2, 4.5 Å)}^{TSRE, Boltz}$	1	$d_{(N1–Ni)}^{TSRE, min}$	1
$d_{(Br–Ni)}^{Int, min}$	1		
Buried Sterimol $L_{(C2\rightarrow R1, 4.5 Å)}^{TSRC, min}$	1		
Sterimol $B5_{(Ni\rightarrow C1s)}^{Int, Boltz}$	1		
**TSRC**	6	**TSRC**	5
**Int**	7	**Int**	3
**TSRE**	4	**TSRE**	6

5. Case Study 3

5.1 Out-of-sample predictions using Model 2

In order to further investigate the domain of applicability of ensemble Model 2 from case study 2, we evaluated its ability to make accurate predictions on a reaction with another unseen C(sp^3)-electrophile. We selected the coupling of benzaldehyde dimethylacetal and 4-Ac-phenyl iodide (Figure S12A, reaction **I**)[32,33] to include one coupling partner from the original training set (aryl iodides, reactions **D**–**G**). However, while the C(sp^3)-electrophiles in the training set (styrenyl aziridines and benzylic chlorides) possess a CH or CH_2 group α to the benzylic position, the C(sp^3)-electrophile in reaction **I** possesses an ether substituent, which can coordinate η^2 to the Ni-center.[34] We tested the model on results from 35 BiOx and 6 BiIm ligands reported in a group member's thesis in 2020.[35] Model 2 afforded inaccurate predictions on this reaction (MAE = 0.38 kcal/mol, R^2 = 0.05, Figure S12B), with the $\Delta\Delta G^\ddagger$ of some ligands being severely overpredicted and associated with a large degree of uncertainty (± 1.79 kcal/mol). To help understand potential reasons for the inaccurate predictions, we analyzed how the distribution of the features in the ensemble model (see Table S21) differs for reactions **D**–**G** *vs*. reaction **H** (for which accurate out-of-sample predictions were obtained) and **I**. An example is given in Figure S12C, which shows the kernel density estimation curve of $d_{(\text{Ni–C2s})}{}^{\text{TSRE, Boltz}}$ *i.e.*, the distance between Ni and the C(sp^3)-atom of the electrophile in the reductive elimination transition state. The distribution of this feature for reaction **H** more closely resembles that of reactions **D**–**G**, whereas for reaction **I** the median is shifted to lower values. This is consistent with the electrophile being more tightly bound to Ni owing to the presence of the O atom, which can act as an additional donor (see the lowest energy **TSRE** conformer of **L1** in Figure S12C).

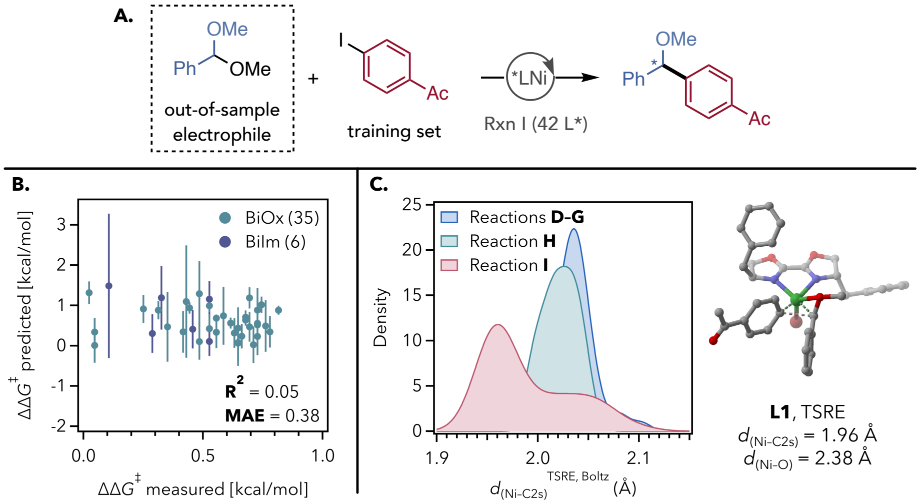

Figure S12. **A.** Ni-catalyzed reductive arylation of benzylic acetals. **B**. Out-of-sample predictions using ensemble Model 2 from case study 2. **C.** Kernel density estimate of feature $d_{(\text{Ni–C2s})}{}^{\text{TSRE, Boltz}}$ for reactions **D**–**G**, **H**, and **I**. The lowest energy **TSRE** conformer of **L1** for reaction **I** is shown.

5.2 GPR model training

EDBO+ was used in the active learning campaign.[11] A Gaussian Process Regression (GPR) model was pre-trained (round 0) on 250 Ni-catalyzed cross-coupling reactions from the literature,[1,4–10,31,36–38] featurized following the workflow described in Figure 2. Their **TSRC–Int–TSRE** descriptors and corresponding $\Delta\Delta G^{‡}$ values are available in the *CCs_Acetals.xlsx* spreadsheet contained in the "Step_9_Active_Learning" folder on GitHub at https://github.com/SigmanGroup/HT_TSs_Opt, together with the *Case_Study_3.ipynb* Jupyter notebook. In rounds 1–5, newly collected experimental data on reaction **I** (see Table S22) was added to the training set, and the GPR model retrained on the 250+n reactions. At each round, prior to training, descriptors were filtered *via* the Boruta algorithm (percentage of maximum importance of shadow features = 75, maximum number of iterations = 100) and based on collinearity (R^2 = 0.90). Only Boltzmann-weighted average values were included for electronic descriptors, whereas min, max, and average values were used for steric parameters. At the start of the active learning campaign, the virtual space consisted of 195 reactions (*i.e.*, 195 untested ligands for reaction **I**), which were featurized according to the Figure 2 workflow. Their corresponding **TSRC–Int–TSRE** descriptors are also included in the *CCs_Acetals.xlsx* spreadsheet. In each round, batches of 4–6 ligands were selected based on their expected improvement (EI) and tested based on the ligands' accessibility to our lab (either in our chemical inventory, commercially available, or easily accessed *via* known synthetic routes from inexpensive starting materials). Figure S14 shows how the MAE of the GPR model changes as more experimental data on reaction **I** is added to the training set.

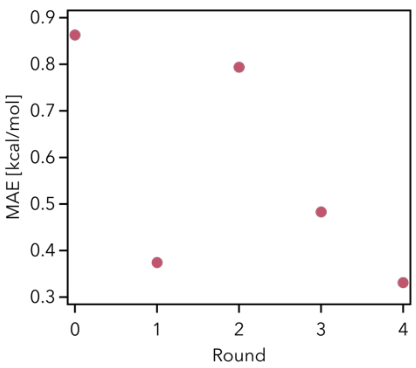

Figure S13. GPR MAE *vs*. EDBO+ round. The MAE was calculated based on the predicted $\Delta\Delta G^{‡}$ value of the ligands tested in round n. At round 0, reaction **I** was entirely unseen. After round 4, the average EI for round 5 (see Figure 6A) was ~5%, and no additional ligands were tested.

5.3 Preparation and characterization of racemic products

1-(4-(methoxy(phenyl)methyl)phenyl)ethan-1-one

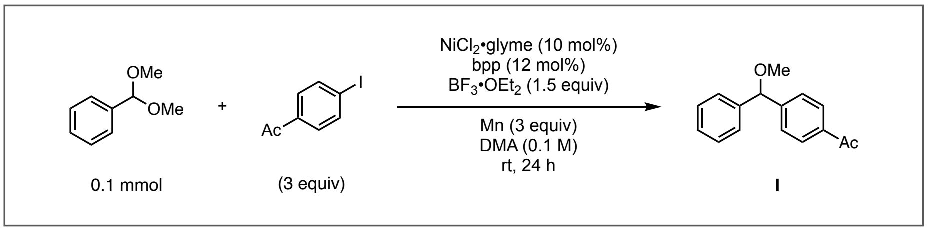

General procedure D: This procedure was adapted from Doyle *et. al.*[32] All reaction preparation was done inside a nitrogen-filled glovebox. Vials were oven-dried (140 °C) and were allowed to cool under vacuum prior to use in the glovebox. To a one-dram vial equipped with a Teflon stir bar were added $NiCl_2$•glyme (2.2 mg, 0.010 mmol, 0.10 equiv), bpp ligand (2.5 mg, 0.012 mmol, 0.12 equiv), and 500 µL DMA. The Ni stock solution was stirred for ~30 minutes. In a separate one-dram reaction vial equipped with a Teflon stir bar, 1-(4-iodophenyl)ethan-1-one (73.8 mg, 0.30 mmol, 3.0 equiv), (dimethoxymethyl)benzene (15.2 mg, 15.0 µL, 0.100 mmol, 1.0 equiv), manganese (16.5 mg, 0.30 mmol, 3.0 equiv), and boron trifluoride etherate (21.3 mg, 18.8 µL, 0.15 mmol, 1.5 equiv) were added. 500 µL DMA and 500 µL of the Ni stock solution were added. The vial was capped with a Teflon septum cap, sealed with electrical tape, removed from the glovebox and stirred at rt for 24 h (700 rpm stir rate). The resulting mixture was diluted with EtOAc, quenched with sat. $NaHCO_3$ solution, and the aqueous layer extracted with EtOAc (2x). The combined organic layers were dried over $NaSO_4$ and concentrated in vacuo. The product was isolated by preparative TLC (25% EtOAc/hexanes) to yield the title compound as a clear oil (13.4 mg, 35% yield). Characterization data are in agreement with reported literature values.[32]

^{1}H NMR (500 MHz, $CDCl_3$) δ 7.48 – 7.44 (m, 2H), 7.33 (d, J = 4.7 Hz, 4H), 7.29 – 7.26 (m, 1H), 5.29 (s, 1H), 3.39 (s, 3H), 2.58 (s, 3H).

^{13}C NMR (126 MHz, $CDCl_3$) δ 197.9, 147.7, 141.4, 136.4, 128.8 (2C), 128.7 (2C), 128.0, 127.12 (2C), 126.99 (2C), 85.1, 57.2, 26.8.

SFC method: Daicel ChiralPak IB column, 15% *i*-PrOH, 9 min run, 1.5 mL/min.

Racemic standard:

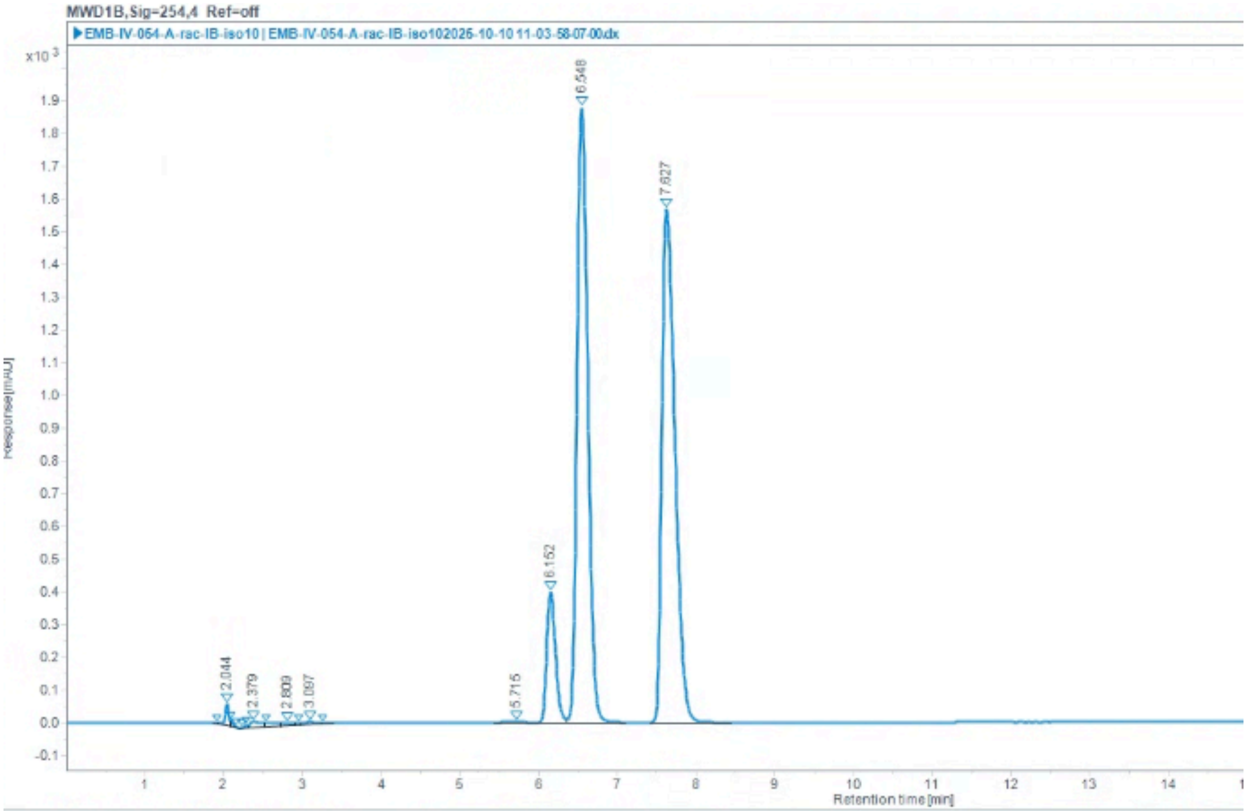

Injection Results

Peaks Summary

#	Signal description	RT (min)	Area (mAU·s)	Area%	Height (mAU)	Height%	Start time (min)	End time (min)
15	MWD1B,Sig=254,4 Ref=off	6.548	17976.753	41.083	1877.097	44.57	6.360	7.112
16	MWD1B,Sig=254,4 Ref=off	7.627	18005.265	41.148	1567.038	37.21	7.417	8.449

5.4 Evaluation of chiral ligands

Reactions **I** were set up according to General Procedure **D**, modifying the ligand as noted in Table S22. Products were isolated *via* preparative TLC for SFC analysis. Note: only enantioselectivity data was used for modeling. The reaction yields reported were determined by GC-FID analysis using 1,3,5-trimethoxybenzene as an external standard.

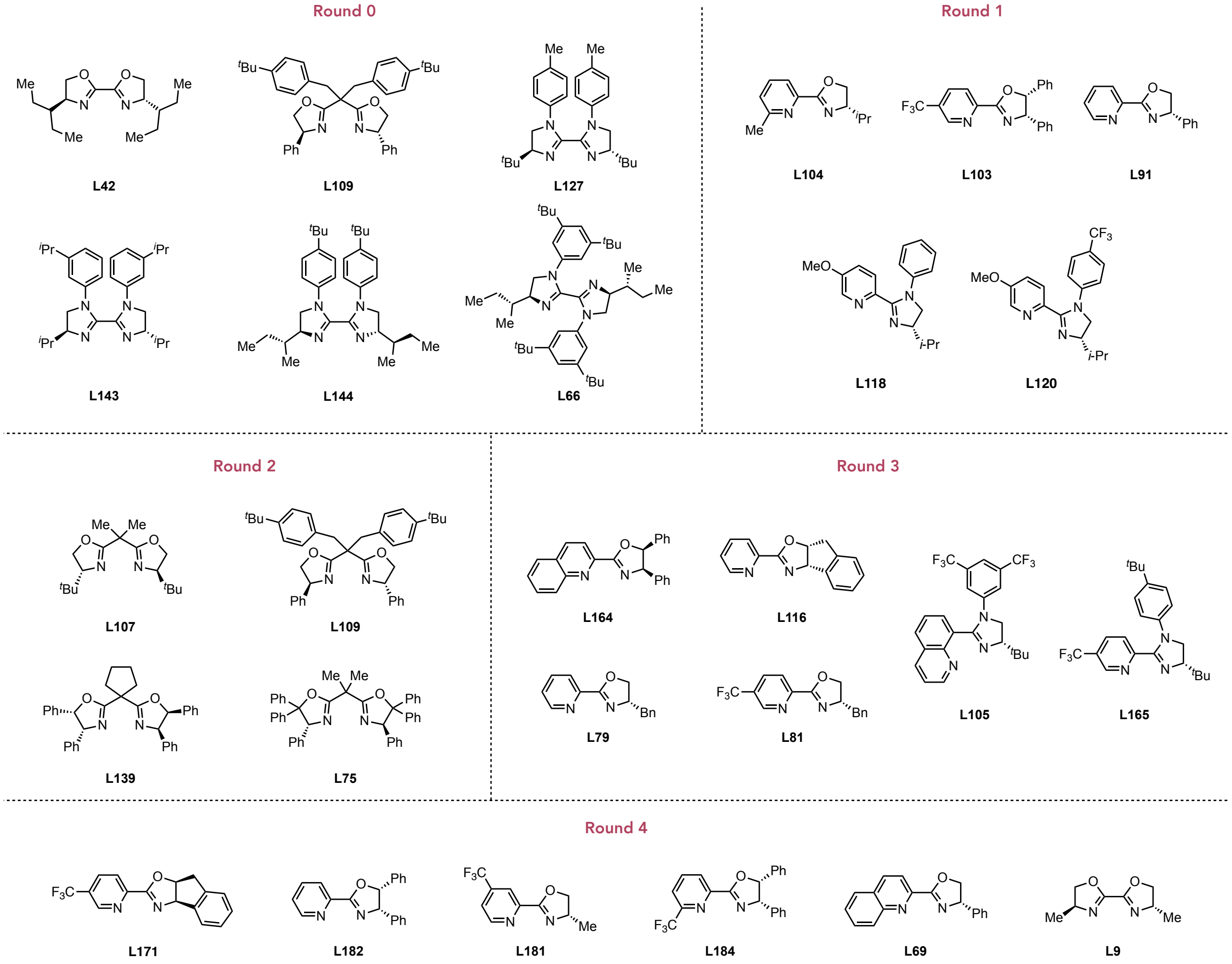

Figure S14. Ligands evaluated in each EDBO+ round.

Table S22. Ligand screening results for Reaction **I**.

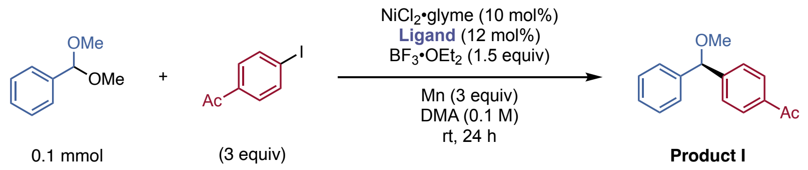

	Entry	Ligand	Yield 1 (%)	ee (%)
round 0	1[a]	L42	–	21
	2	L109	0	–
	3	L127	35	0
	4	L143	16	49
	5	L144	14	39
	6	L66	9	44
round 1	1	L104	35	7
	2	L103	9	37
	3	L91	9	65
	4	L118	6	20
	5	L120	10	39
round 2	1	L107	36	0
	2[b]	L109	2	14
	3	L75	2	1
	4	L139	3	29
round 3	1	L164	10	2
	2	L116	12	68
	3	L105	34	0
	4	L165	7	10
	5	L79	12	66
	6	L81	14	17
round 4	1	L171	7	20
	2	L182	16	38
	3	L181	19	1
	4	L184	11	0
	5	L69	18	4
	6[a]	L9	–	52

[a]BiOx ligands **L42** and **L9** had already been evaluated (see Ref. 35) and yield data was unavailable. Only enantioselectivity data was used in training the GPR model. [b]THF (0.1 M) instead of DMA was used as reaction solvent.

Representative SFC trace for enantioenriched product (L91):

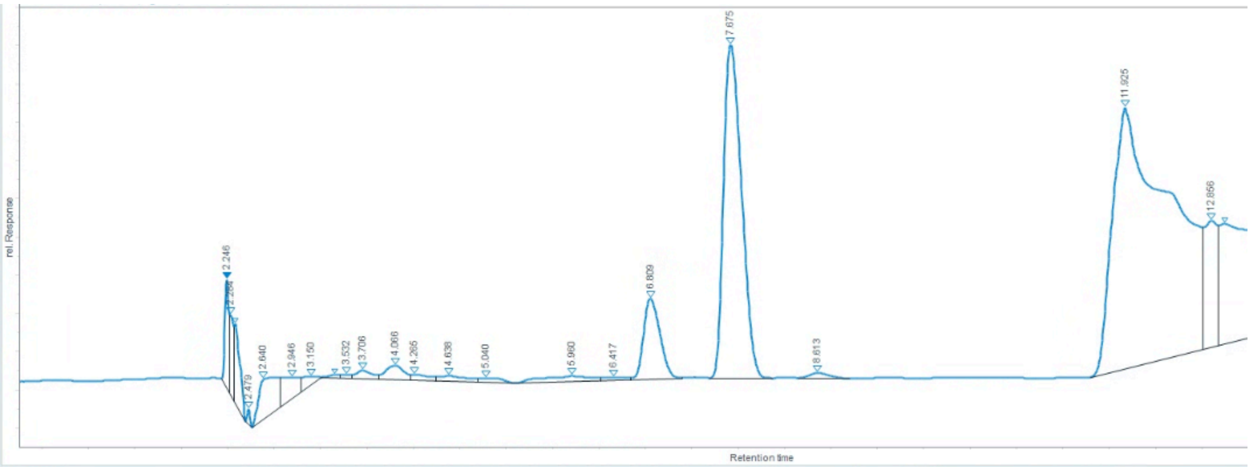

Injection Results

Peaks | Summary

#	Name	Signal description	RT (min)	Area (mAU·s)	Area%	Height (mAU)	Height%	Amount	Concentration	Start time (min)	End time (min)
17		DAD1C,Sig=210,4 Ref=off	6.809	721.793	3.478	58.047	5.29			6.595	7.157
18		DAD1C,Sig=210,4 Ref=off	7.675	3211.527	15.475	237.850	21.68			7.447	8.133

6. Additional Synthesis and Characterization of Ligands

L16, **L28**, **L29**, and **L144** were synthetized for a prior study reported by our lab[1] and used without further purification.

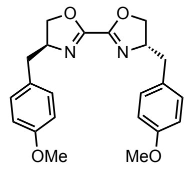

L16

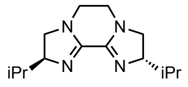

L28

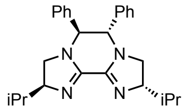

L29

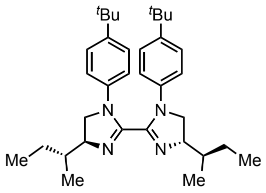

L144

<u>**L16:**</u> Characterization data are in agreement with reported literature values.[39]

^{1}H NMR (500 MHz, $CDCl_3$): δ 7.13 (dt, *J* = 8.7, 2.9 Hz, 4H), 6.85 (dt, *J* = 8.7, 2.8 Hz, 4H), 4.56 (tdd, *J* = 9.3, 8.0, 5.0 Hz, 2H), 4.36 (t, *J* = 8.7 Hz, 2H), 4.15 (t, *J* = 8.0 Hz, 2H), 3.79 (s, 6H), 3.20 (dd, *J* = 14.0, 5.0 Hz, 2H), 2.65 (dd, *J* = 13.9, 9.1 Hz, 2H).
^{13}C NMR (126 MHz, $CDCl_3$): δ 158.6, 155.2, 130.3, 129.3, 114.3, 72.8, 68.6, 55.4, 40.3.

<u>**L28:**</u> Characterization data are in agreement with reported literature values.[40]

^{1}H NMR (500 MHz, $CDCl_3$): δ 3.85 (dt, *J* = 10.1, 2.3 Hz, 2H), 3.62 (t, *J* = 9.4 Hz, 2H), 3.42 – 3.24 (m, 4H), 2.95 (dd, *J* = 12.2, 9.2 Hz, 2H), 1.86 (hept, *J* = 6.7 Hz, 2H), 1.07 (d, *J* = 6.6 Hz, 6H), 0.90 (d, *J* = 6.8 Hz, 6H).
^{13}C NMR (126 MHz, $CDCl_3$): δ 153.7, 71.7, 55.2, 45.2, 33.0, 20.0, 18.8.

<u>**L29:**</u>

^{1}H NMR (500 MHz, C_6D_6): δ 7.03 – 6.96 (m, 6H), 6.89 – 6.85 (m, 4H), 3.91 (dt, *J* = 11.5, 5.9 Hz, 2H), 3.86 (s, 2H), 2.78 (dd, *J* = 9.5, 6.0 Hz, 2H), 2.49 (t, *J* = 10.2 Hz, 2H), 1.80 (h, *J* = 6.6 Hz, 2H), 1.01 (d, *J* = 6.8 Hz, 6H), 0.92 (d, *J* = 6.7 Hz, 6H).
^{13}C NMR (126 MHz, C_6D_6): δ 153.4, 138.4, 128.6, 128.4, 128.2, 128.0, 71.4, 68.0, 52.6, 33.5, 18.9, 18.9.
HRMS: (APCI): Calculated for $C_{26}H_{33}N_4^+$ ($[M+H]^+$): 401.2700, found: 401.2700.
FTIR: (ATR, cm^{-1}): 2957, 2927, 2870, 1674, 1483, 1455, 1384, 1366, 1335, 1306, 1261, 1205, 1180, 909, 756, 699.
Optical Rotation: $[\alpha]_D^{26}$ (*c* 1.0, $CHCl_3$) = –154.2

<u>**L144:**</u>

^{1}H NMR (500 MHz, C_6D_6): δ 7.11 (d, *J* = 8.4 Hz, 3H), 6.85 (d, *J* = 8.7 Hz, 3H), 3.94 (td, *J* = 10.1, 6.8 Hz, 2H), 3.41 (t, *J* = 9.5 Hz, 2H), 3.31 (t, *J* = 9.6 Hz, 2H), 1.79 – 1.65 (m, 4H), 1.23* (s, 20H), 0.92 – 0.80 (m, 12H).
* *t*-Bu peak signal (s, 18H) overlaps with signal from methine H in *s*-Bu group (m, 2H).
^{13}C NMR (126 MHz, C_6D_6): δ 153.6, 145.4, 138.7, 125.5, 119.3, 70.1, 53.5, 39.8, 34.2, 31.6, 26.4, 15.4, 11.7.
HRMS: (APCI): Calculated for $C_{34}H_{51}N_4^+$ ($[M+H]^+$): 515.4108, found: 515.4104.
FTIR: (ATR, cm^{-1}): 2959, 2903, 2871, 1635, 1598, 1569, 1517, 1473, 1430, 1362, 1318, 1268, 1202, 831, 731.
Optical Rotation: $[\alpha]_D^{26}$ (*c* 1.0, $CHCl_3$) = –220.9

Ligands **L118** and **L120** were synthetized according to the reported procedure from Liu *et al.*[36]

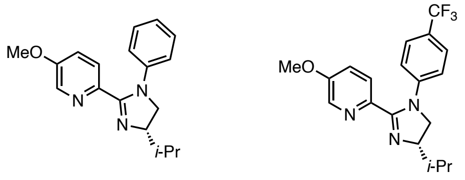

L118 **L120**

L118: Characterization data are in agreement with reported literature values.[36]

^{1}H NMR (500 MHz, $CDCl_3$): δ 8.18 (d, *J* = 2.9 Hz, 1H), 7.65 (d, *J* = 8.7 Hz, 1H), 7.18 – 7.07 (m, 3H), 6.94 (td, *J* = 7.4, 1.2 Hz, 1H), 6.75 – 6.68 (m, 2H), 4.15 – 4.05 (m, 2H), 3.83 (s, 3H), 3.75 – 3.68 (m, 1H), 1.94 (dq, *J* = 13.2, 6.8 Hz, 1H), 1.06 (d, *J* = 6.8 Hz, 3H), 0.95 (d, *J* = 6.7 Hz, 3H).
^{13}C NMR (126 MHz, $CDCl_3$): δ 160.1, 156.2, 143.3, 142.8, 137.2, 128.7 (2C), 125.0, 123.0, 122.0 (2C), 120.4, 70.5, 56.4, 55.7, 33.2, 19.2, 18.1.

L120: Characterization data are in agreement with reported literature values.[36]

^{1}H NMR (500 MHz, $CDCl_3$): δ 8.19 (d, *J* = 2.9 Hz, 1H), 7.76 (d, *J* = 8.6 Hz, 1H), 7.36 (d, *J* = 8.4 Hz, 2H), 7.23 (dd, *J* = 8.7, 3.0 Hz, 1H), 6.69 (d, *J* = 8.4 Hz, 2H), 4.17 – 4.07 (m, 2H), 3.88 (s, 3H), 3.80 – 3.73 (m, 1H), 1.94 (dt, *J* = 13.0, 6.5 Hz, 1H), 1.06 (d, *J* = 6.7 Hz, 3H), 0.96 (d, *J* = 6.8 Hz, 3H).
^{13}C NMR (126 MHz, $CDCl_3$): δ 159.1, 156.6, 145.7, 142.3, 137.4, 125.8 (2C, q, *J* = 3.8 Hz), 125.1, 124.4 (q, *J* = 271.2 Hz), 123.9 (q, *J* = 32.8 Hz) 120.7, 120.5 (2C), 70.5, 55.81, 55.78, 33.0, 19.1, 18.1.
^{19}F NMR (282 MHz, $CDCl_3$) δ -61.92.

7. NMR Spectra

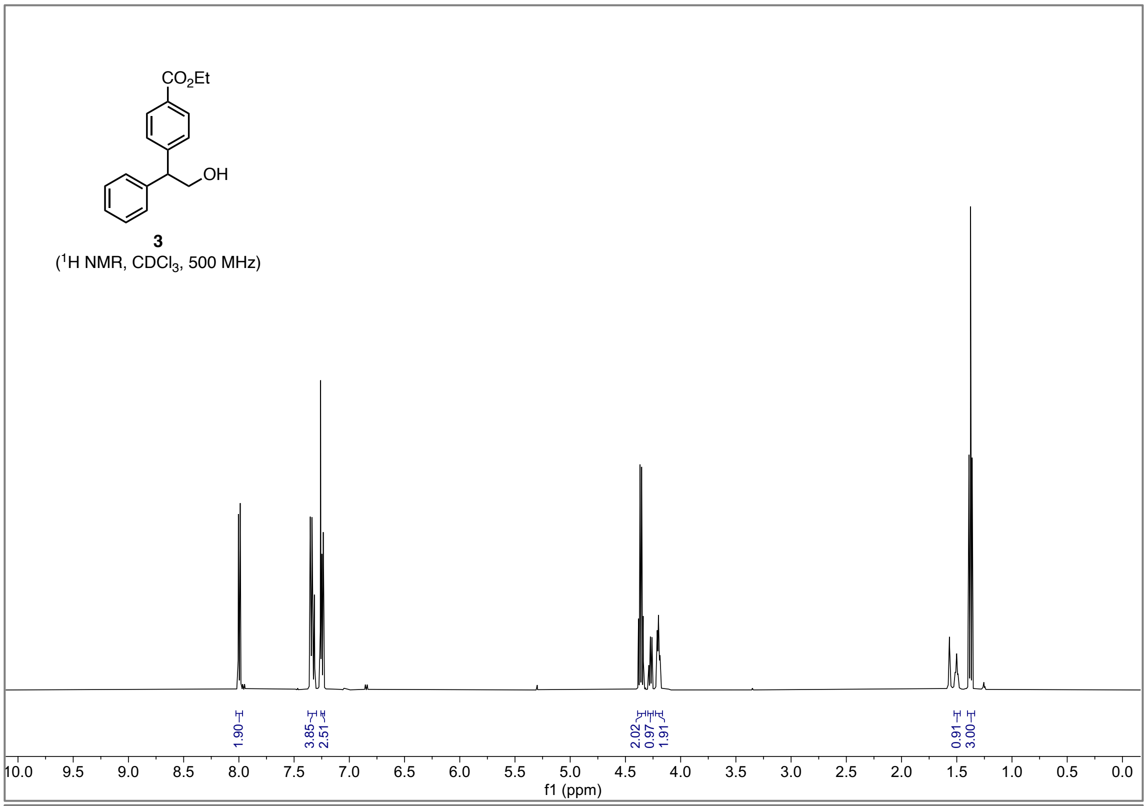

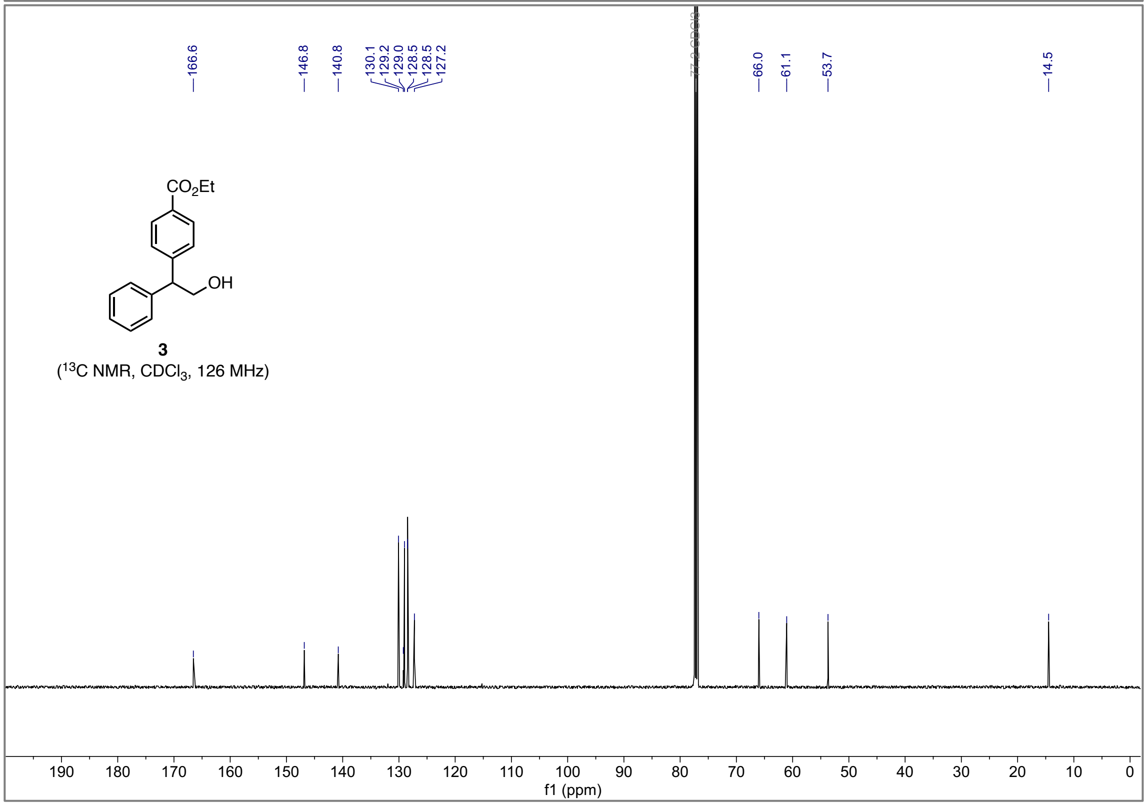

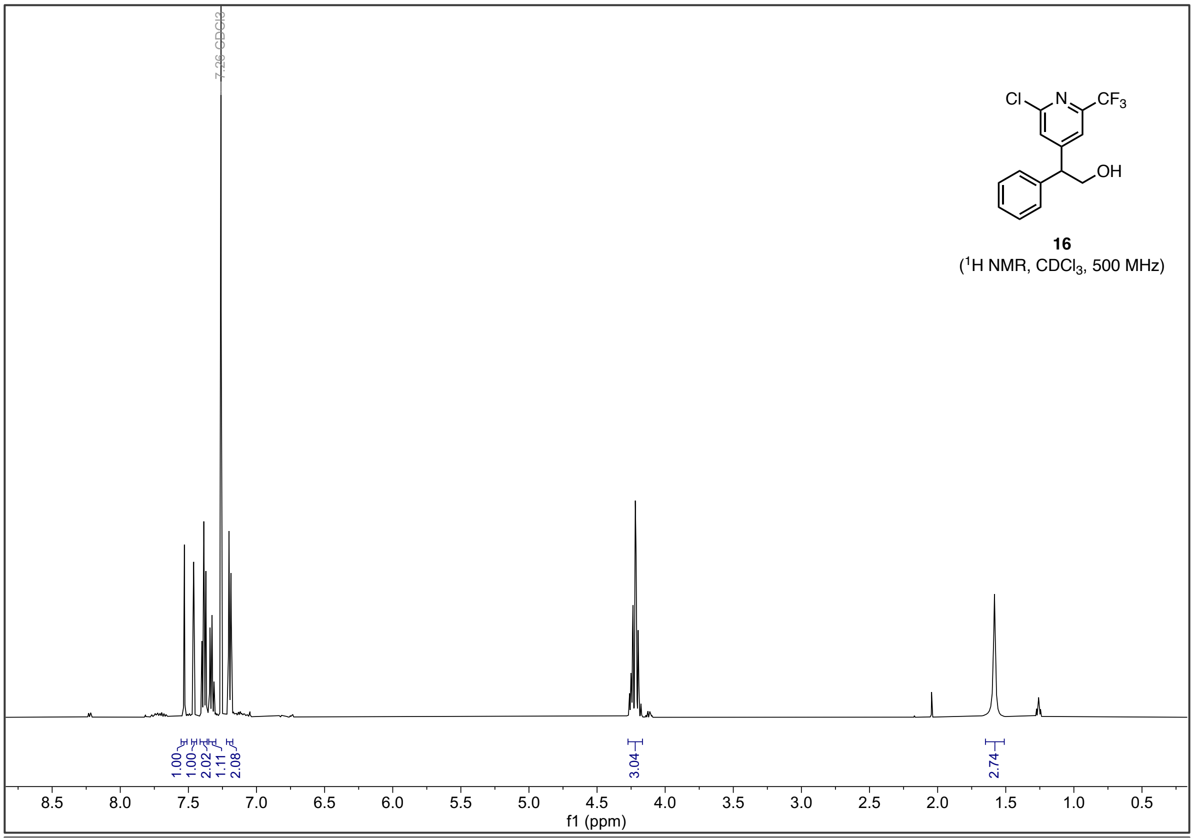
Cl
N
CF3
OH
16
(1H NMR, CDCl3, 500 MHz)
7.26 CDCl3
1.00
1.00
2.02
1.11
2.08
3.04
2.74
8.5 8.0 7.5 7.0 6.5 6.0 5.5 5.0 4.5 4.0 3.5 3.0 2.5 2.0 1.5 1.0 0.5
f1 (ppm)

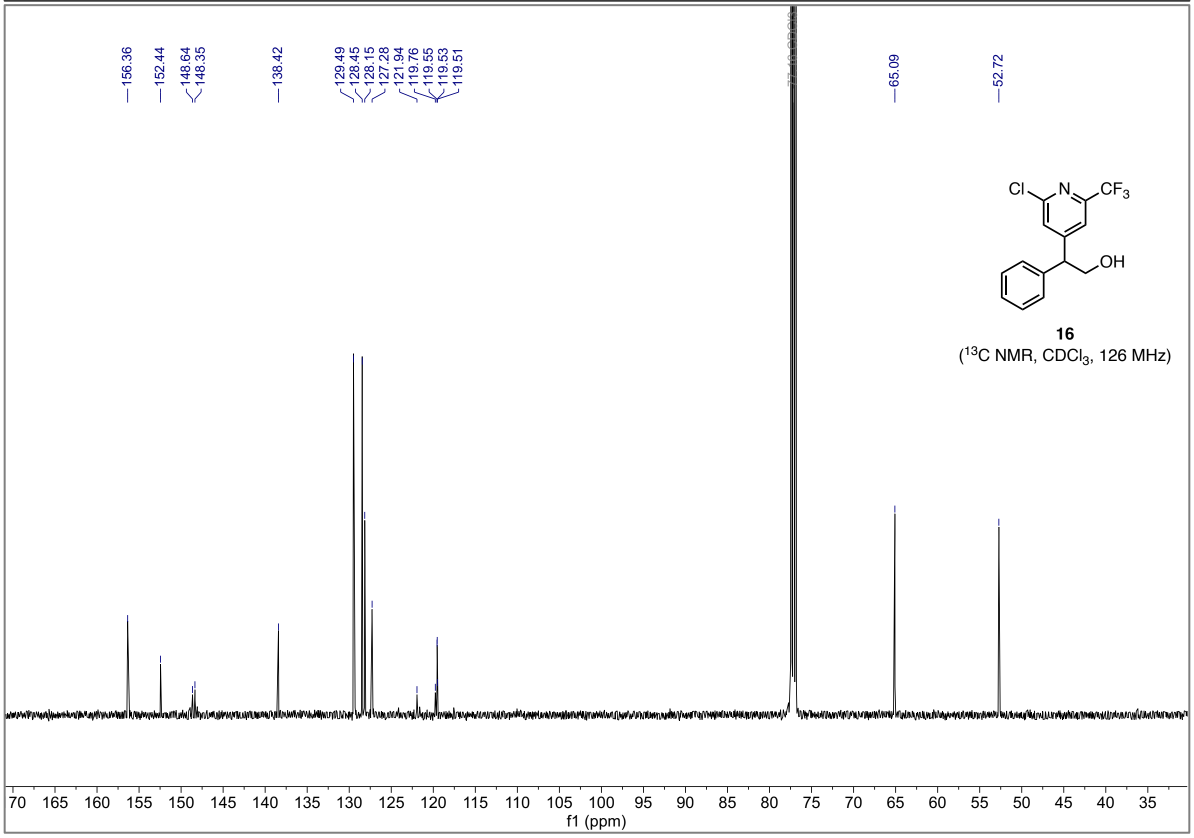
156.36
152.44
148.64
148.35
138.42
129.49
128.45
128.15
127.28
121.94
119.76
119.55
119.53
119.51
65.09
52.72
Cl
N
CF3
OH
16
(13C NMR, CDCl3, 126 MHz)
70 165 160 155 150 145 140 135 130 125 120 115 110 105 100 95 90 85 80 75 70 65 60 55 50 45 40 35
f1 (ppm)

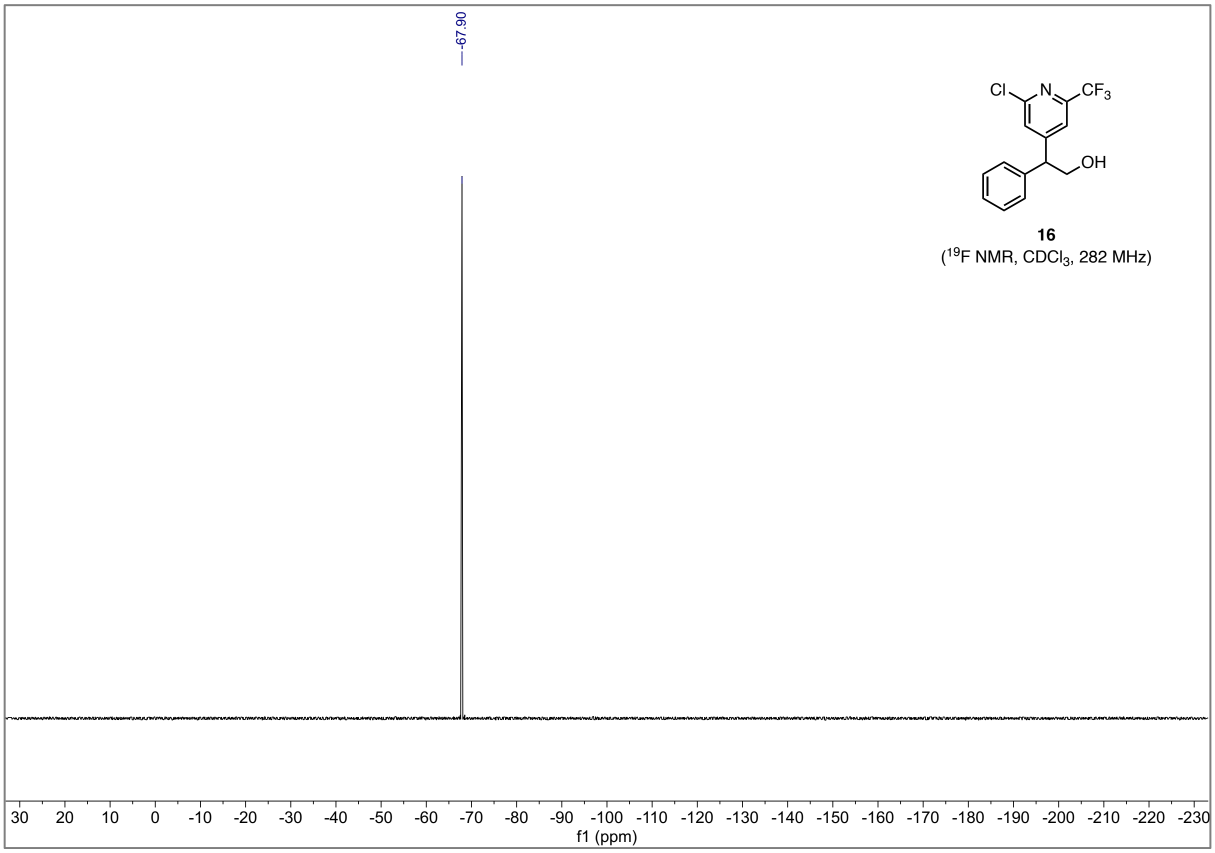
—-67.90
Cl
N
CF3
OH
16
(19F NMR, CDCl3, 282 MHz)
30 20 10 0 -10 -20 -30 -40 -50 -60 -70 -80 -90 -100 -110 -120 -130 -140 -150 -160 -170 -180 -190 -200 -210 -220 -230
f1 (ppm)

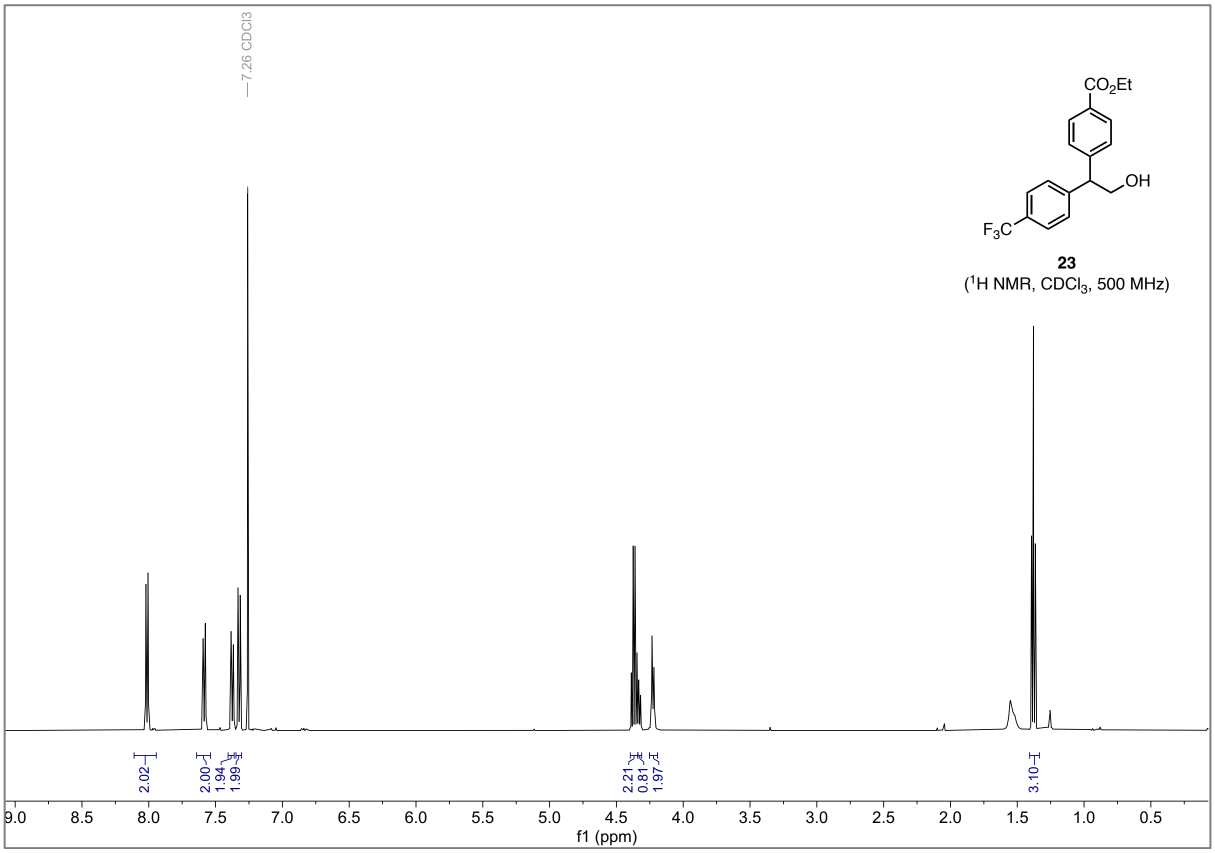
—7.26 CDCl3
CO2Et
OH
F3C
23
(1H NMR, CDCl3, 500 MHz)
2.02
2.00
1.94
1.99
2.21
0.81
1.97
3.10
9.0 8.5 8.0 7.5 7.0 6.5 6.0 5.5 5.0 4.5 4.0 3.5 3.0 2.5 2.0 1.5 1.0 0.5
f1 (ppm)

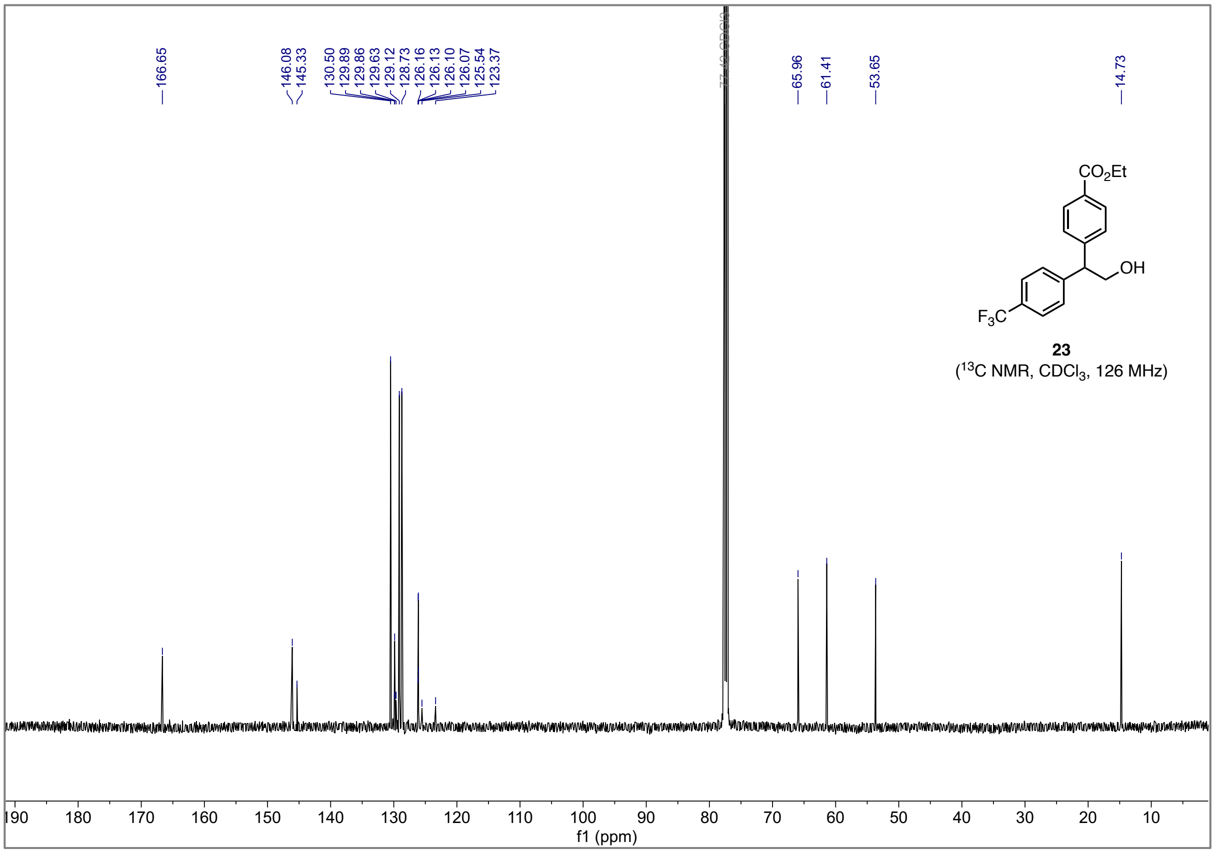
166.65
146.08
145.33
130.50
129.89
129.86
129.63
129.12
128.73
126.16
126.13
126.10
126.07
125.54
123.37
65.96
61.41
53.65
14.73
CO2Et
OH
F3C
23
(13C NMR, CDCl3, 126 MHz)
f1 (ppm)

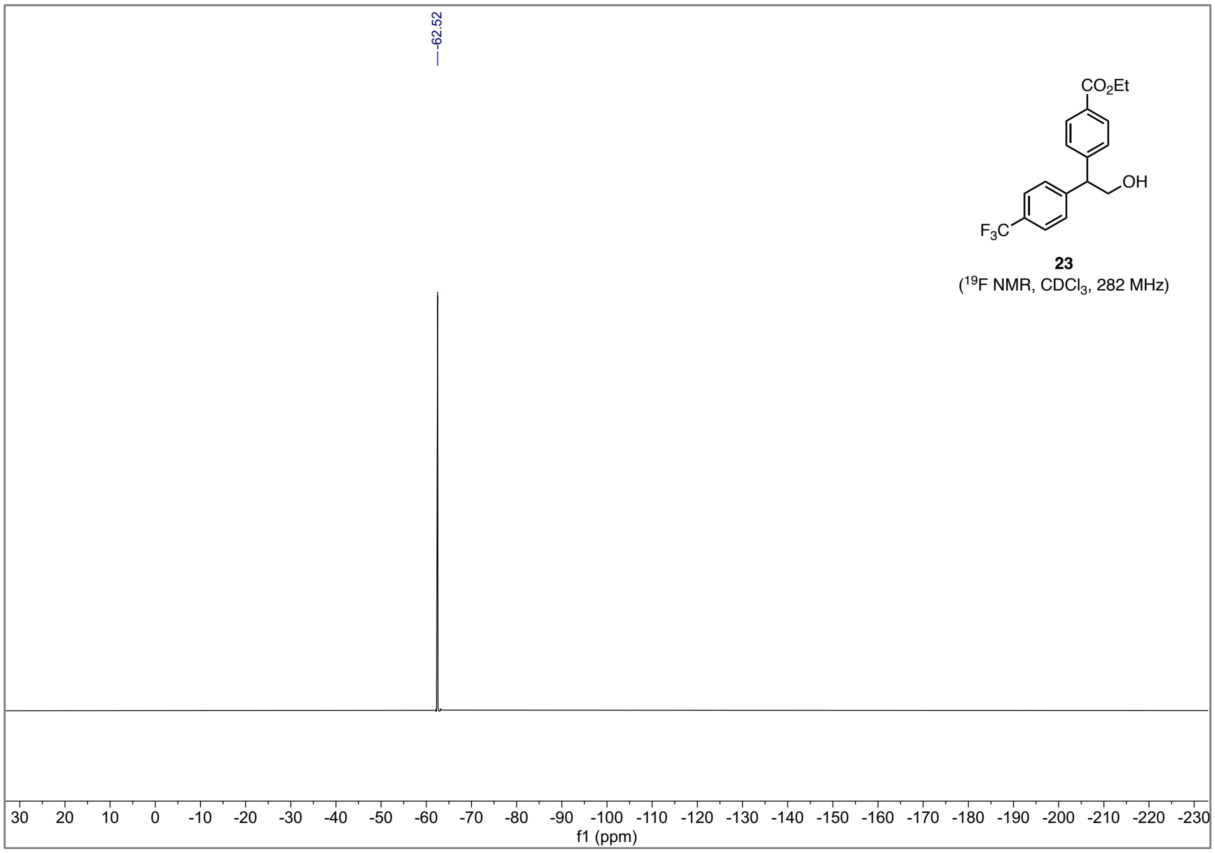
-62.52
CO2Et
OH
F3C
23
(19F NMR, CDCl3, 282 MHz)
f1 (ppm)

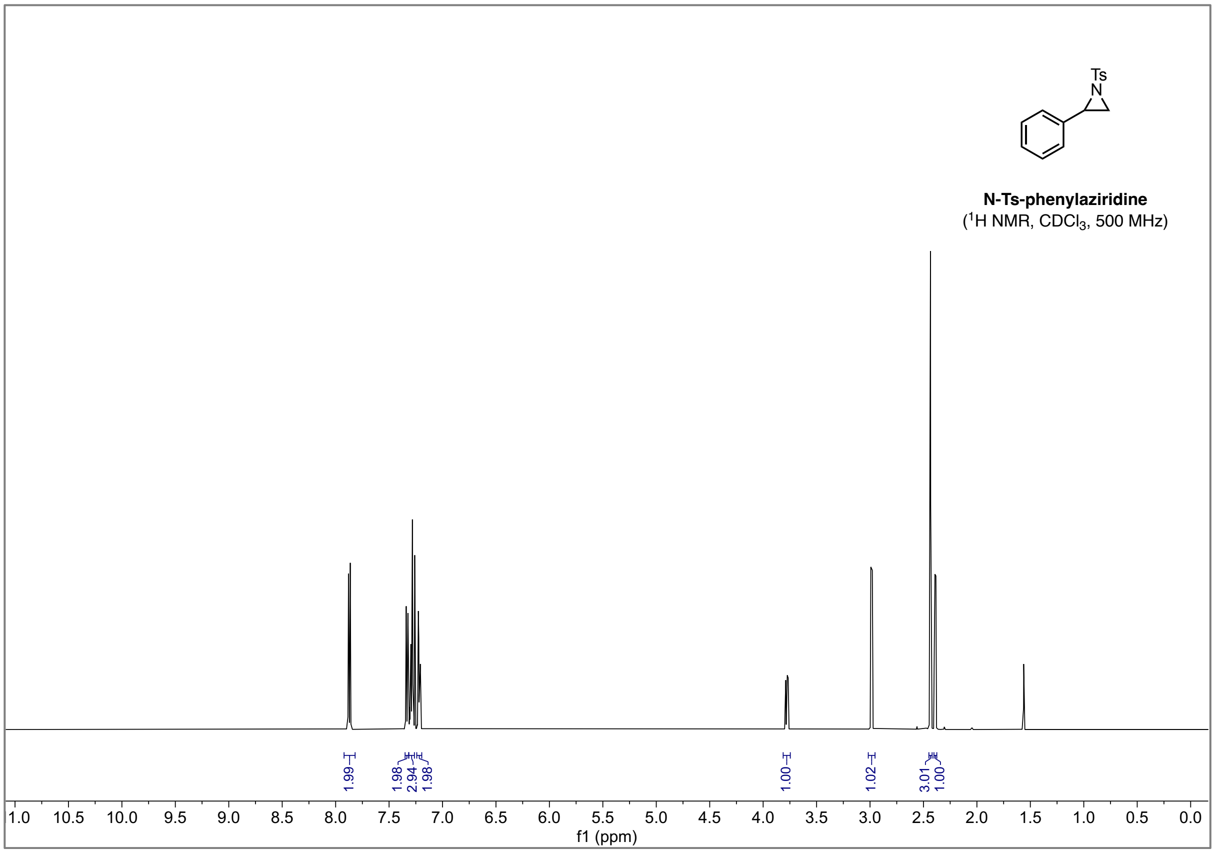
Ts
N
N-Ts-phenylaziridine
(1H NMR, CDCl3, 500 MHz)
1.99
1.98
2.94
1.98
1.00
1.02
3.01
1.00
f1 (ppm)

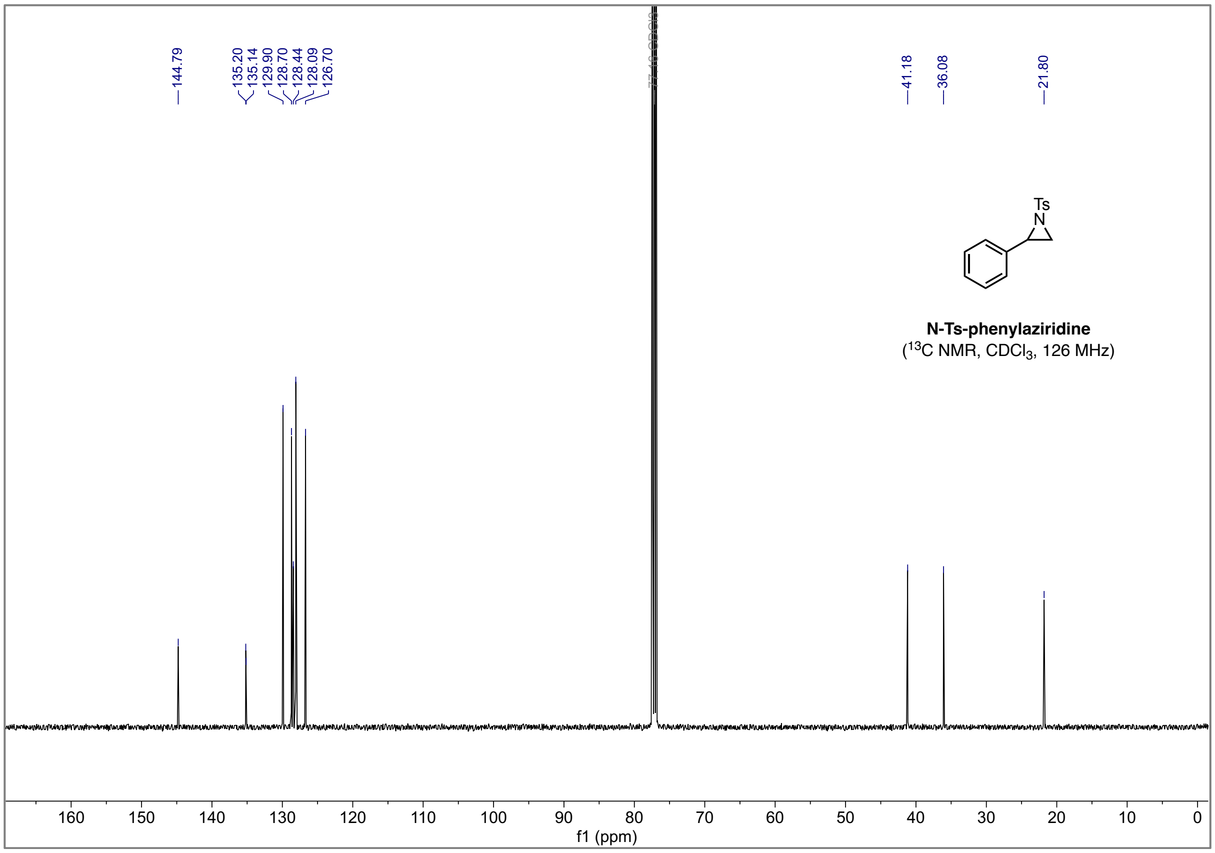
144.79
135.20
135.14
129.90
128.70
128.44
128.09
126.70
41.18
36.08
21.80
Ts
N
N-Ts-phenylaziridine
(13C NMR, CDCl3, 126 MHz)
f1 (ppm)

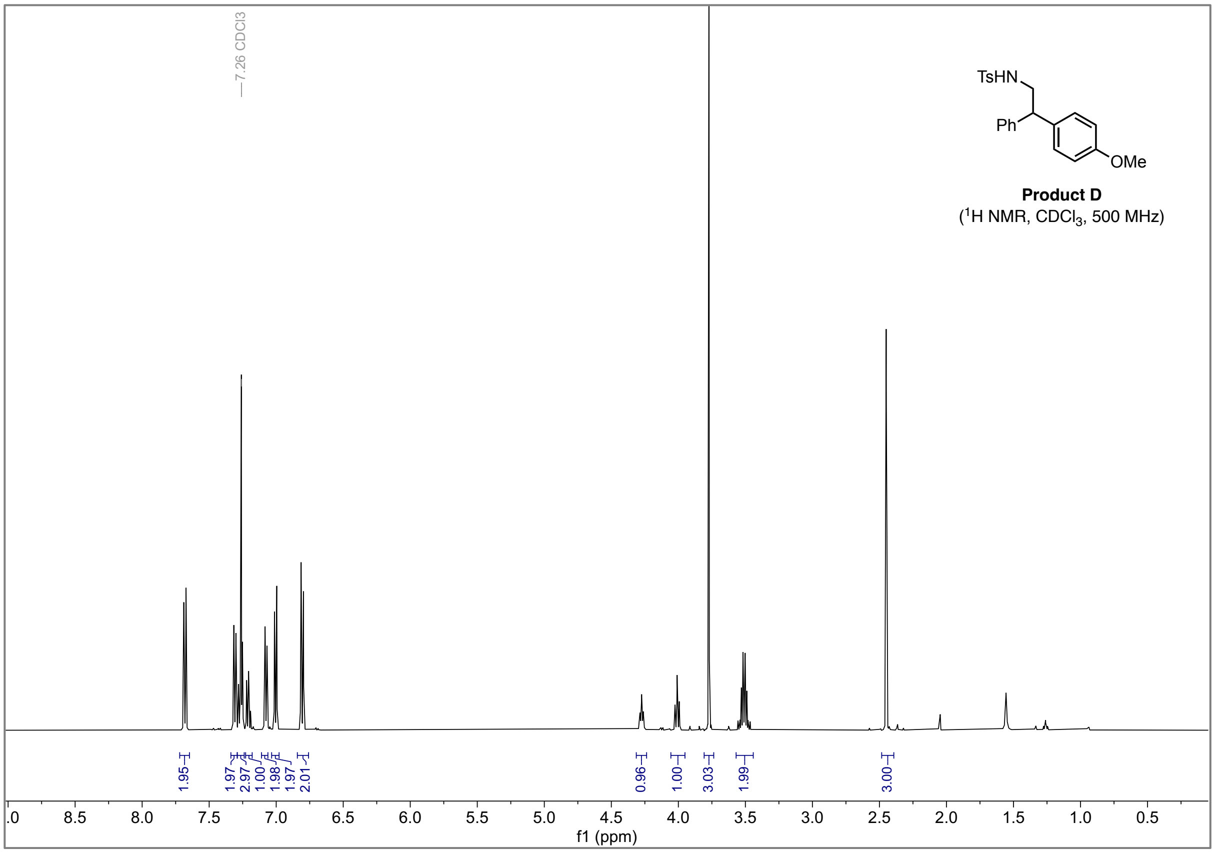
TsHN
Ph
OMe
Product D
(¹H NMR, CDCl₃, 500 MHz)
7.26 CDCl3
f1 (ppm)

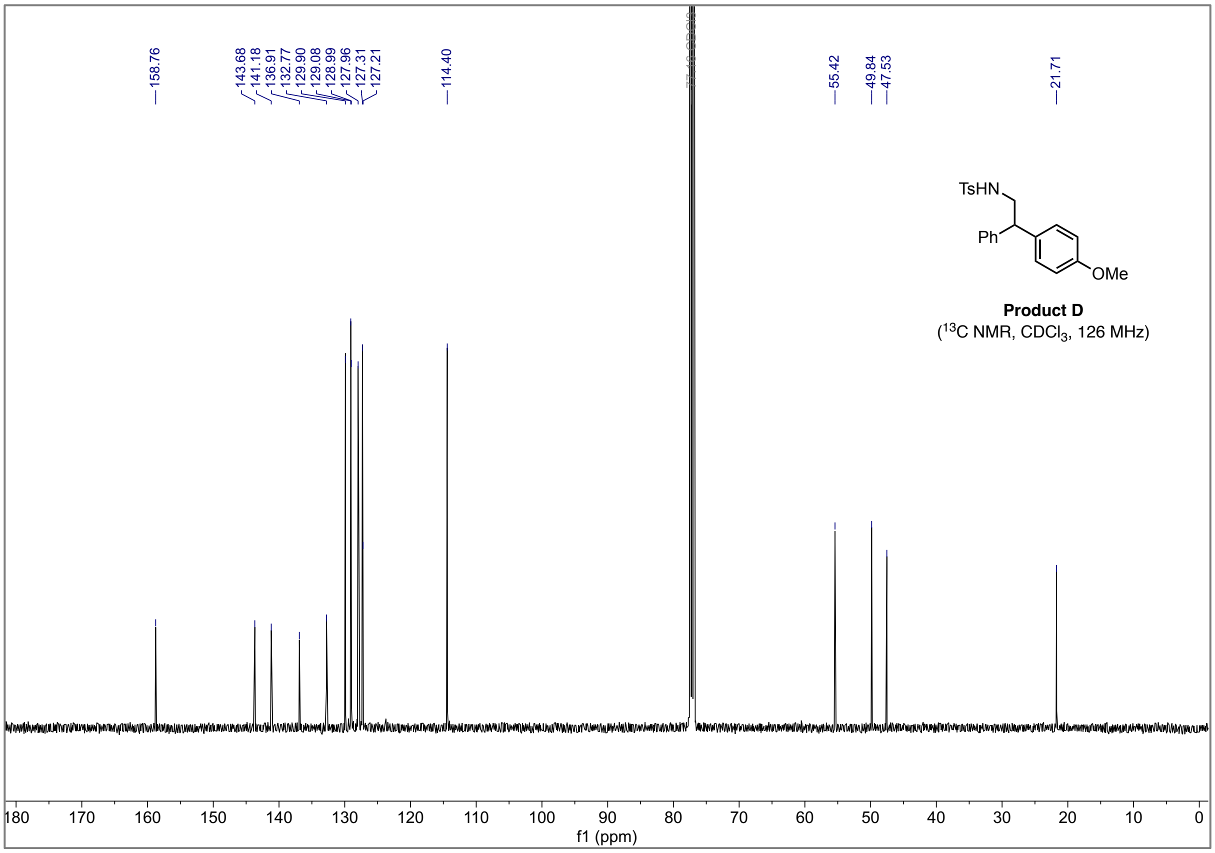
158.76
143.68
141.18
136.91
132.77
129.90
129.08
128.99
127.96
127.31
127.21
114.40
55.42
49.84
47.53
21.71
TsHN
Ph
OMe
Product D
(¹³C NMR, CDCl₃, 126 MHz)
f1 (ppm)

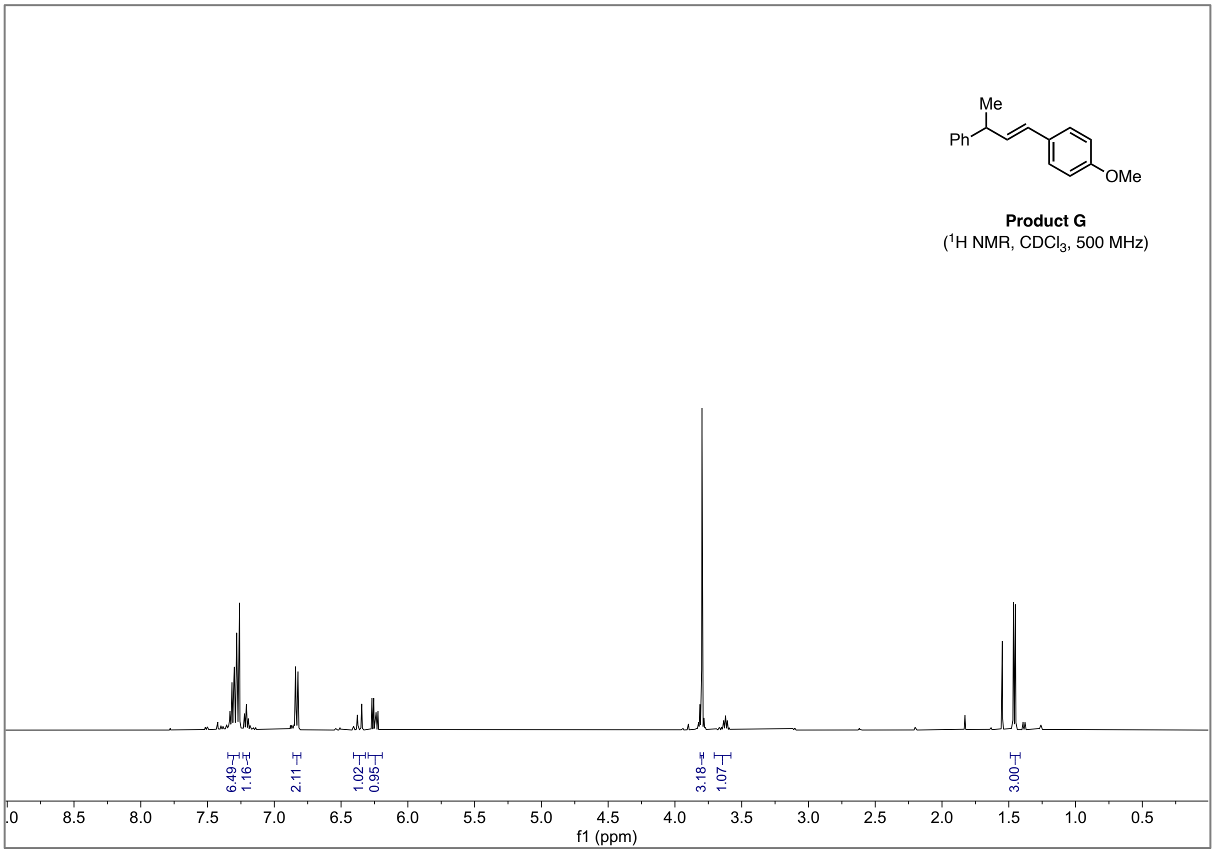
Me
Ph
OMe
Product G
(1H NMR, CDCl3, 500 MHz)
6.49
1.16
2.11
1.02
0.95
3.18
1.07
3.00
f1 (ppm)

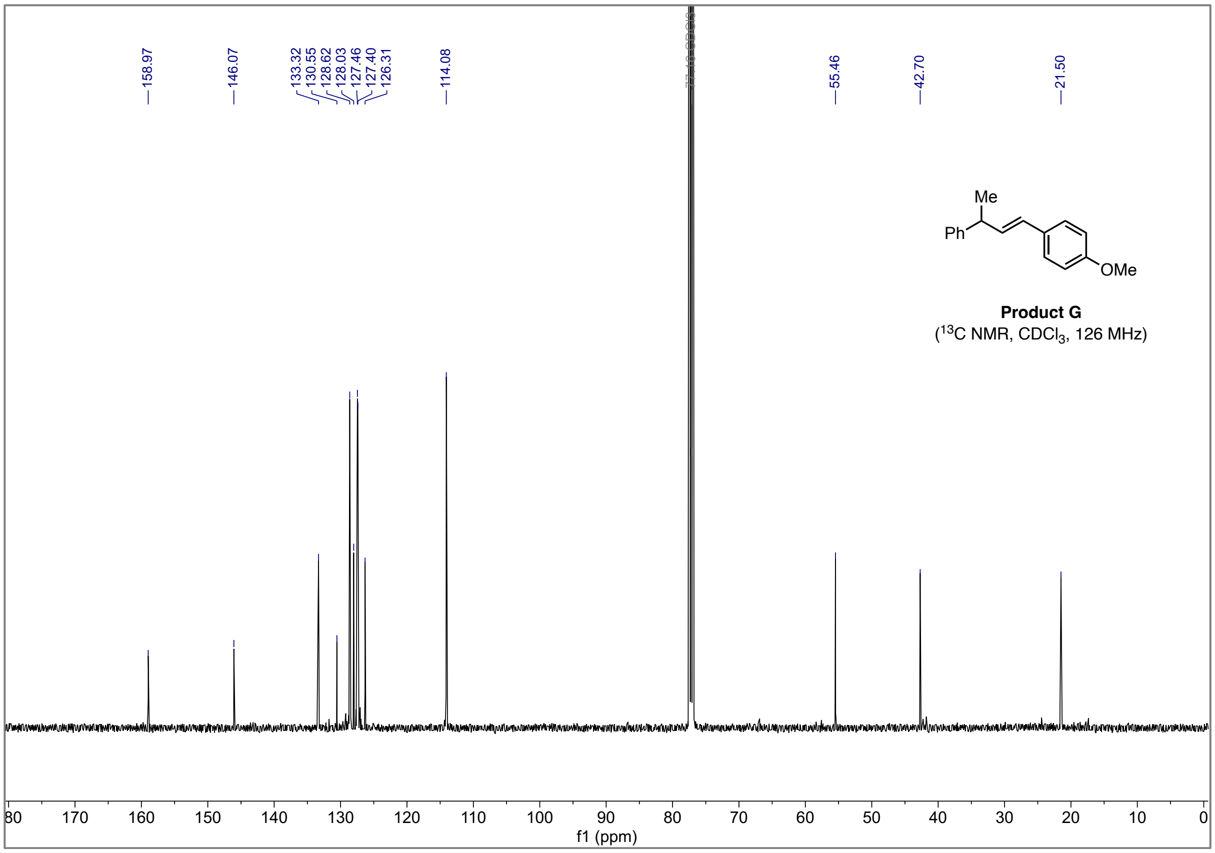
158.97
146.07
133.32
130.55
128.62
128.03
127.46
127.40
126.31
114.08
55.46
42.70
21.50
Me
Ph
OMe
Product G
(13C NMR, CDCl3, 126 MHz)
f1 (ppm)

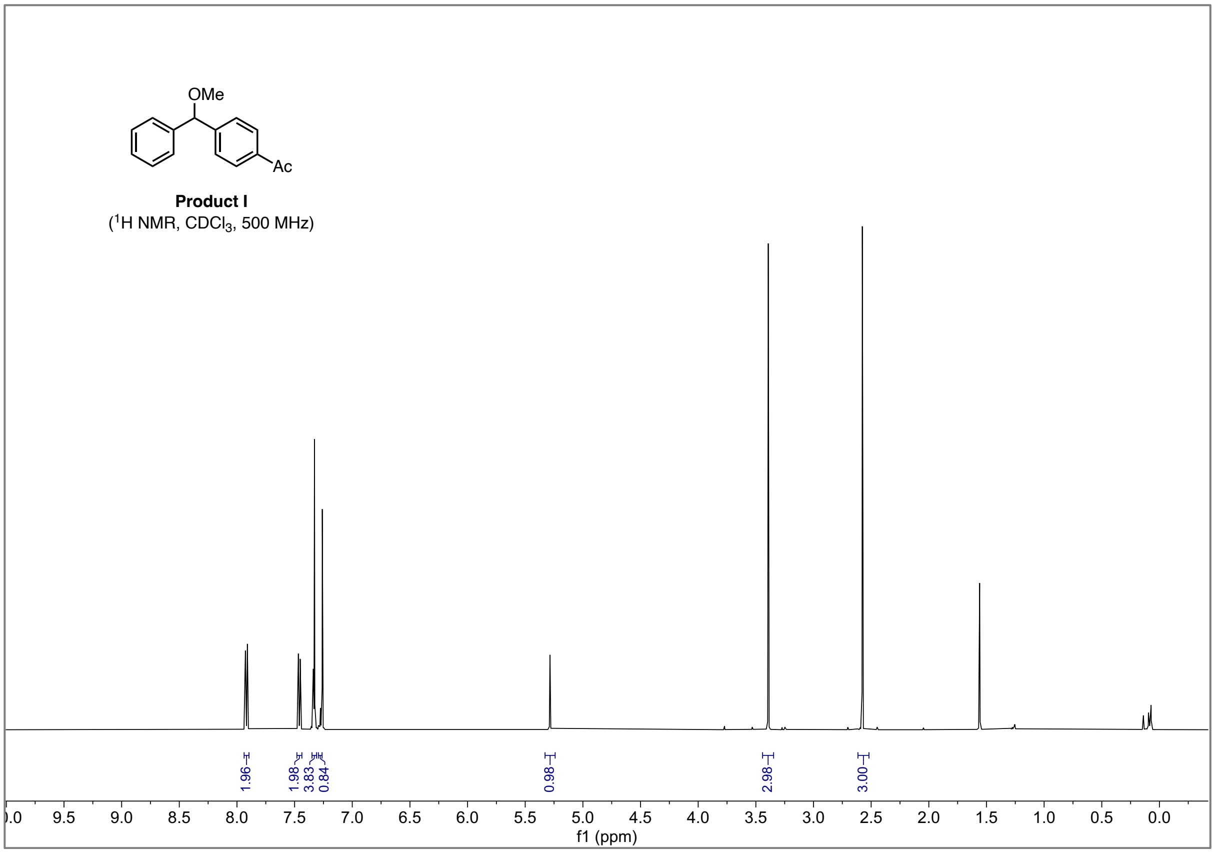
OMe
Ac
Product I
(1H NMR, CDCl3, 500 MHz)
1.96
1.98
3.83
0.84
0.98
2.98
3.00
f1 (ppm)

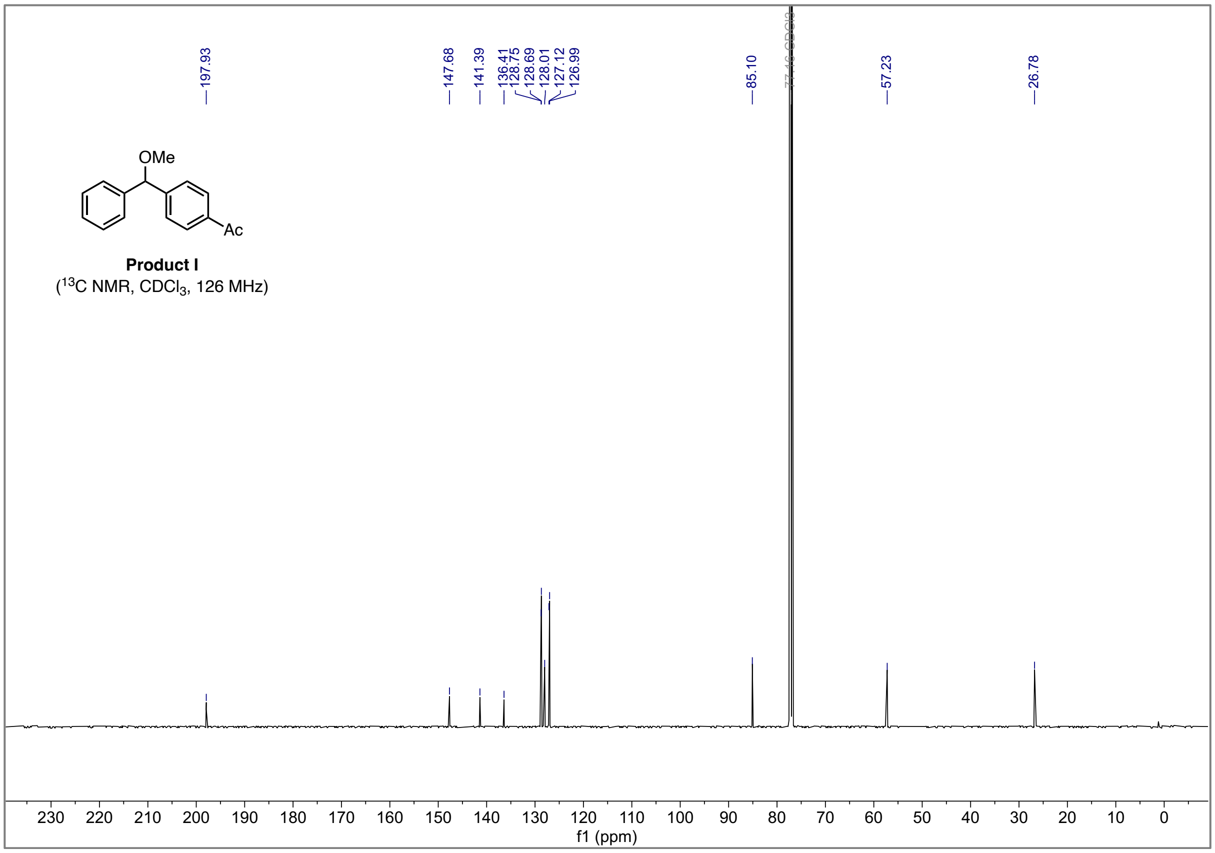
197.93
147.68
141.39
136.41
128.75
128.69
128.01
127.12
126.99
85.10
57.23
26.78
OMe
Ac
Product I
(13C NMR, CDCl3, 126 MHz)
f1 (ppm)

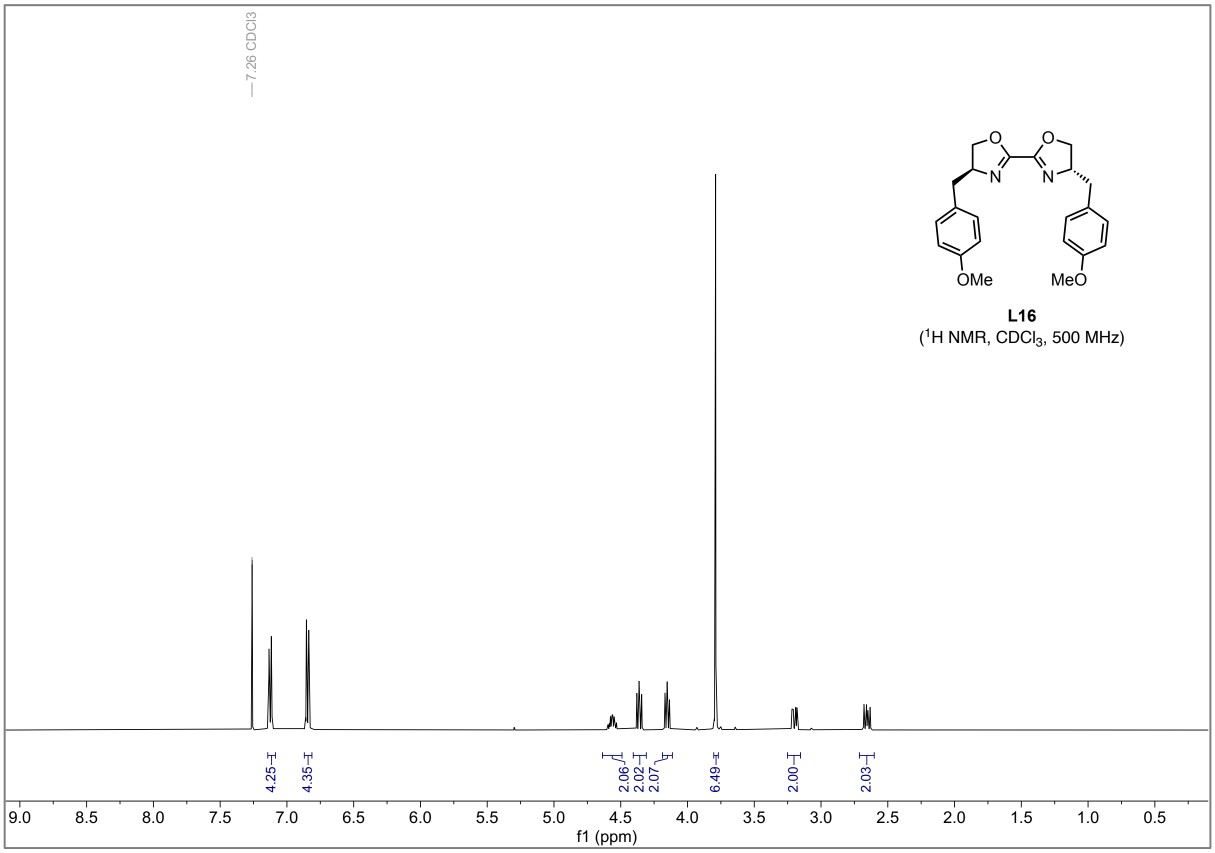

L16
(^{1}H NMR, $CDCl_3$, 500 MHz)

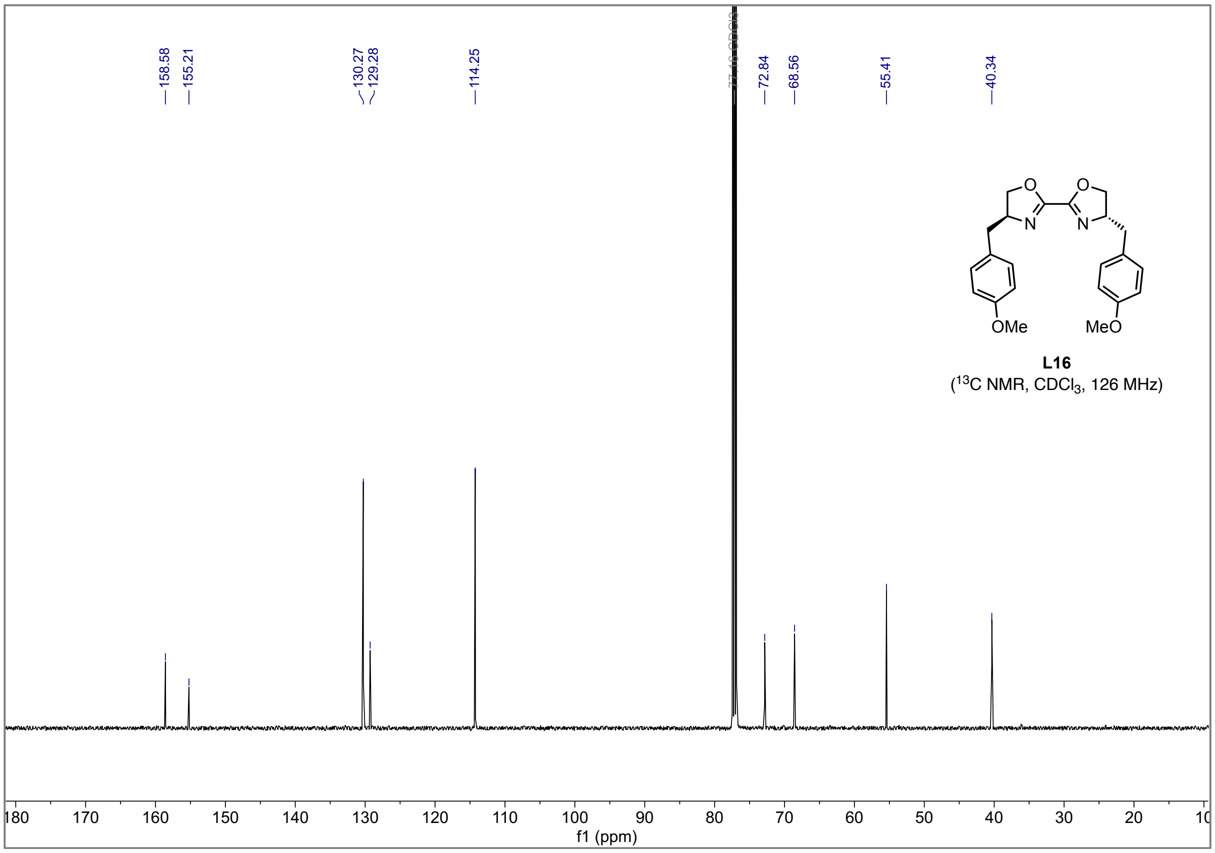

L16
(^{13}C NMR, $CDCl_3$, 126 MHz)

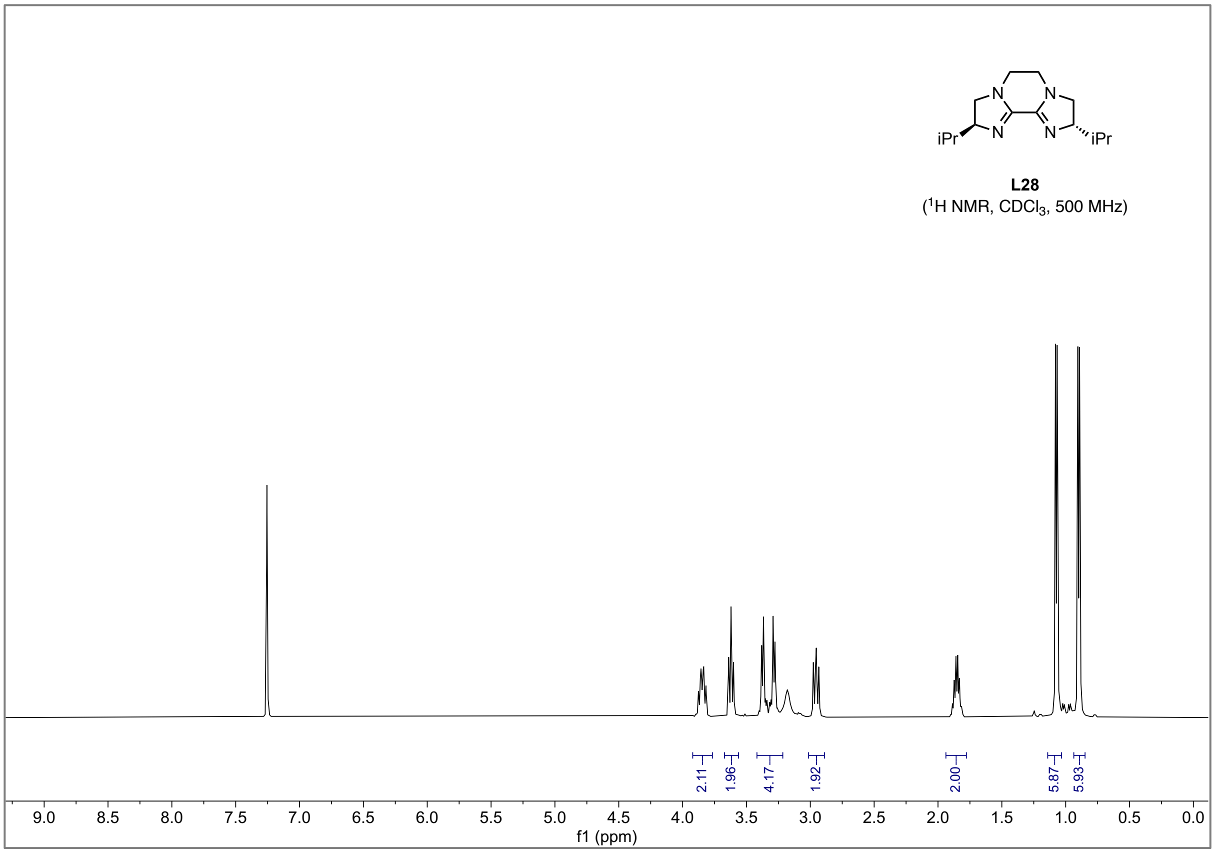

iPr
N
N
iPr
L28
(¹H NMR, CDCl₃, 500 MHz)
2.11
1.96
4.17
1.92
2.00
5.87
5.93
9.0 8.5 8.0 7.5 7.0 6.5 6.0 5.5 5.0 4.5 4.0 3.5 3.0 2.5 2.0 1.5 1.0 0.5 0.0
f1 (ppm)

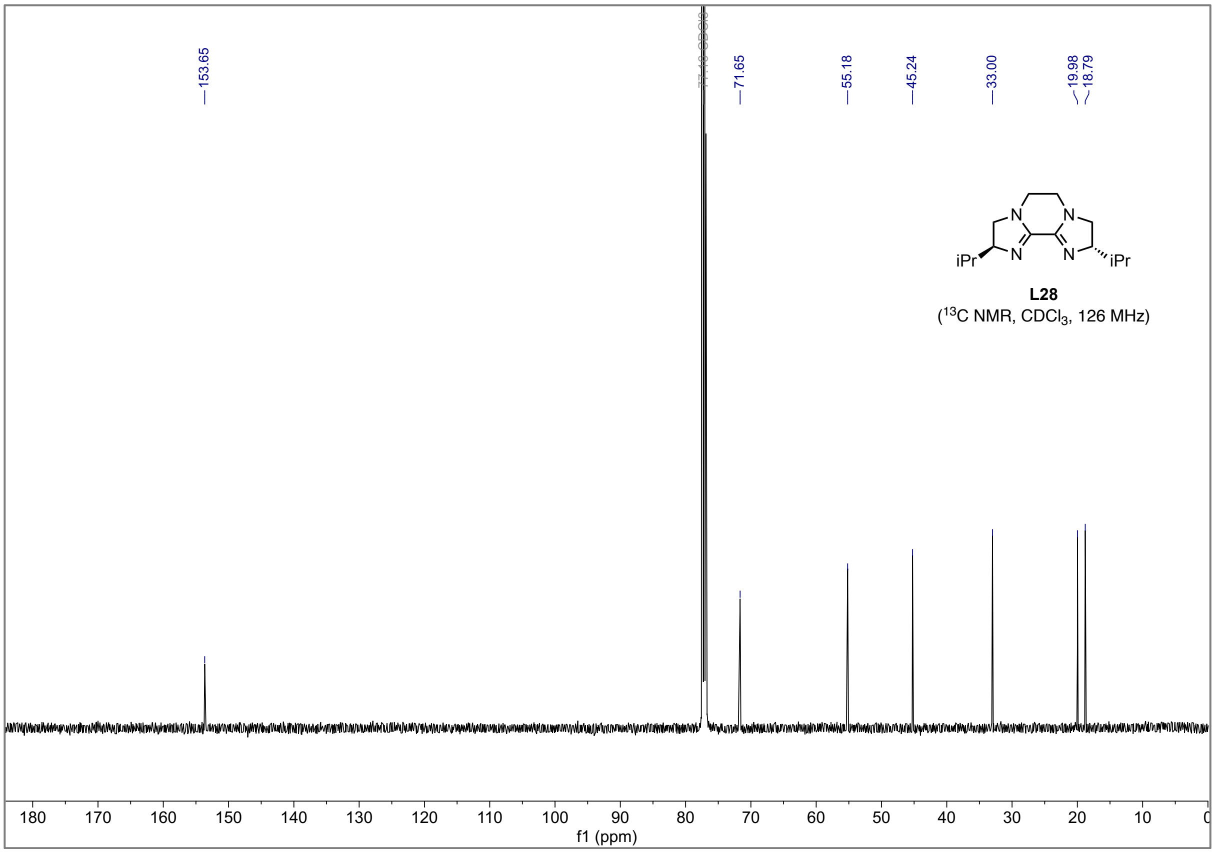

153.65
71.65
55.18
45.24
33.00
19.98
18.79
iPr
N
N
iPr
L28
(¹³C NMR, CDCl₃, 126 MHz)
180 170 160 150 140 130 120 110 100 90 80 70 60 50 40 30 20 10 0
f1 (ppm)

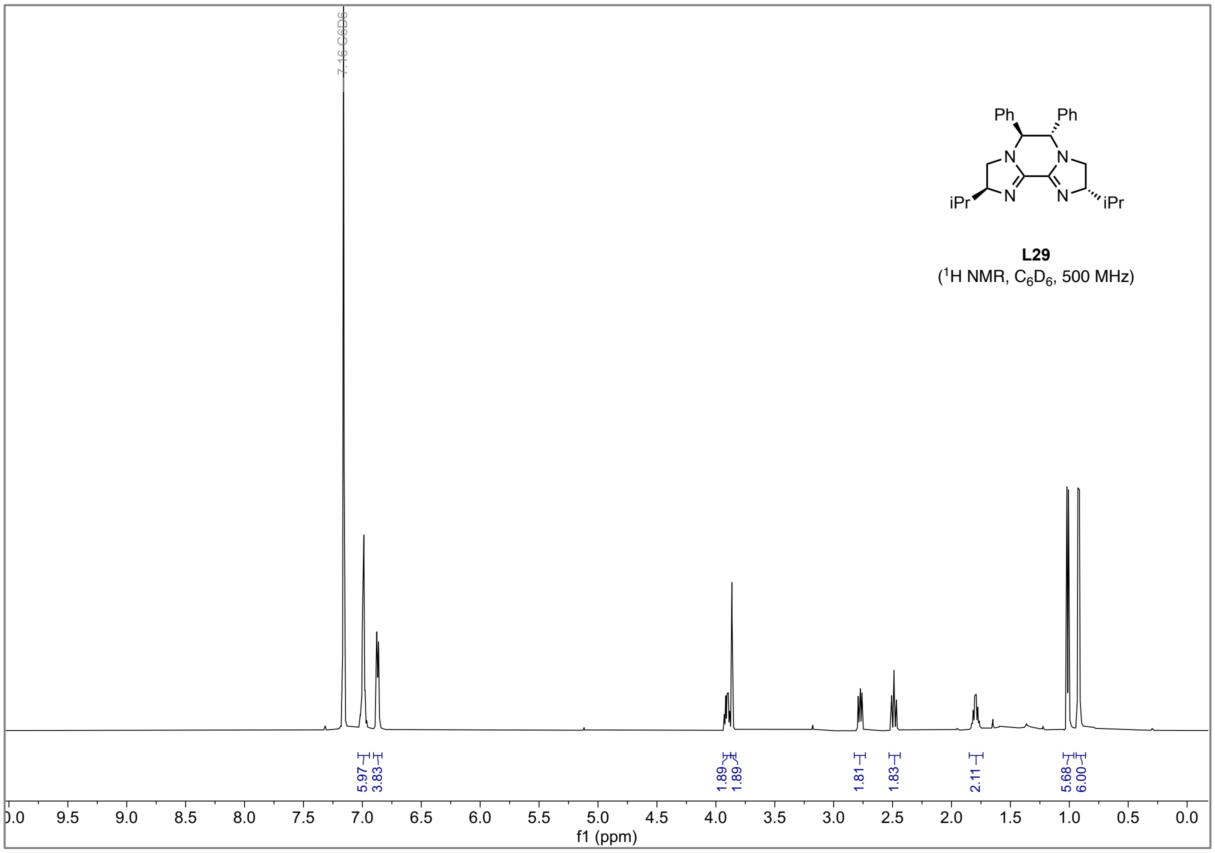
7.16 C6D6
Ph
Ph
N
N
N
N
iPr
iPr
L29
(1H NMR, C6D6, 500 MHz)
5.97
3.83
1.89
1.89
1.81
1.83
2.11
5.68
6.00
f1 (ppm)

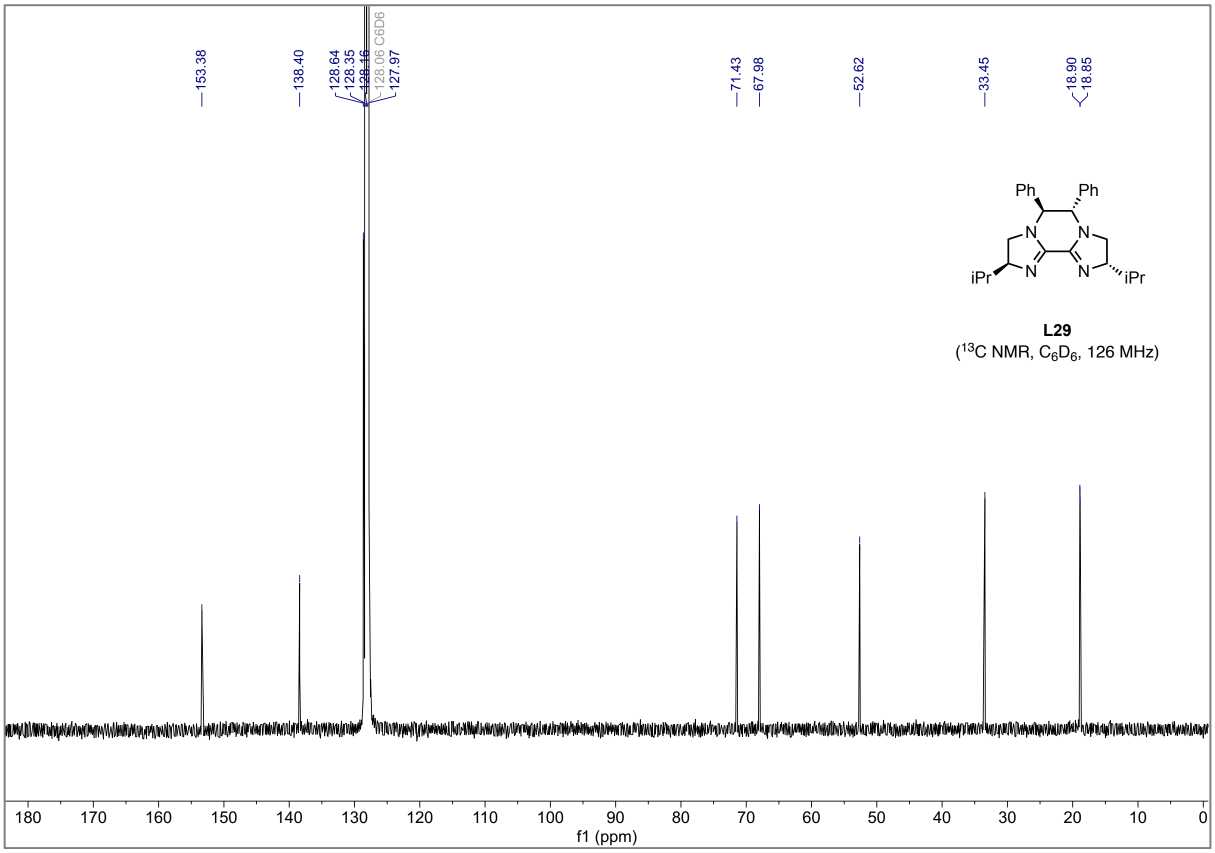
153.38
138.40
128.64
128.35
128.16
128.06 C6D6
127.97
71.43
67.98
52.62
33.45
18.90
18.85
Ph
Ph
N
N
N
N
iPr
iPr
L29
(13C NMR, C6D6, 126 MHz)
f1 (ppm)

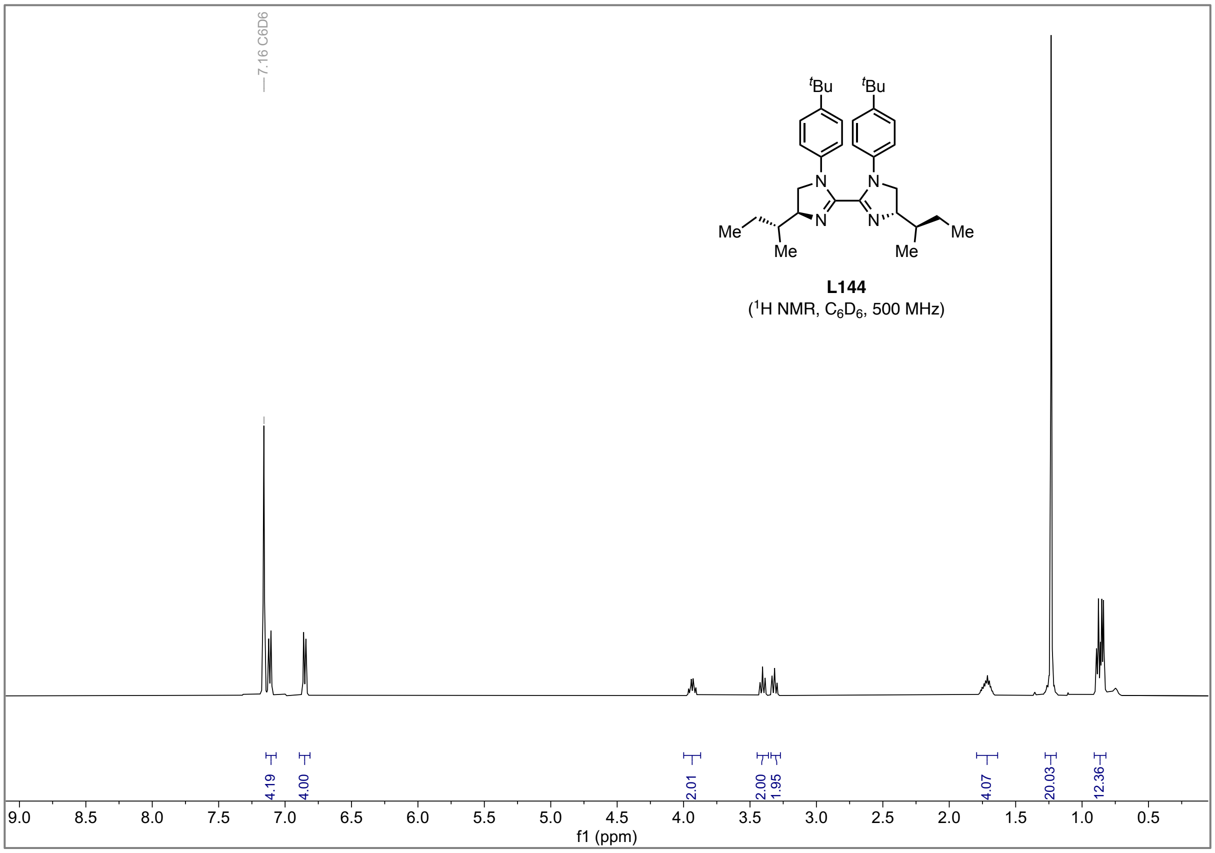

7.16 C6D6
tBu
tBu
N
N
N
N
Me
Me
Me
Me
L144
(1H NMR, C6D6, 500 MHz)
4.19
4.00
2.01
2.00
1.95
4.07
20.03
12.36
9.0
8.5
8.0
7.5
7.0
6.5
6.0
5.5
5.0
4.5
4.0
3.5
3.0
2.5
2.0
1.5
1.0
0.5
f1 (ppm)

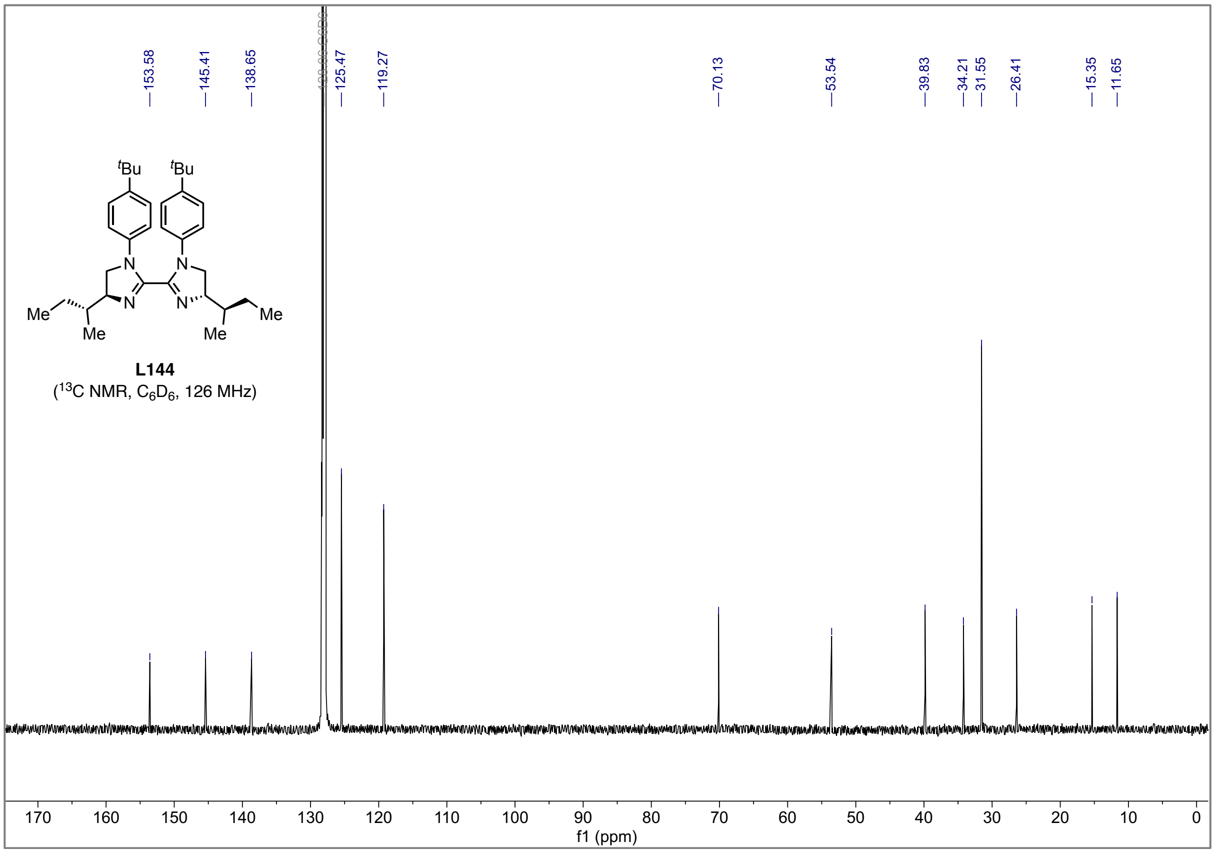

153.58
145.41
138.65
125.47
119.27
70.13
53.54
39.83
34.21
31.55
26.41
15.35
11.65
tBu
tBu
N
N
N
N
Me
Me
Me
Me
L144
(13C NMR, C6D6, 126 MHz)
170
160
150
140
130
120
110
100
90
80
70
60
50
40
30
20
10
0
f1 (ppm)

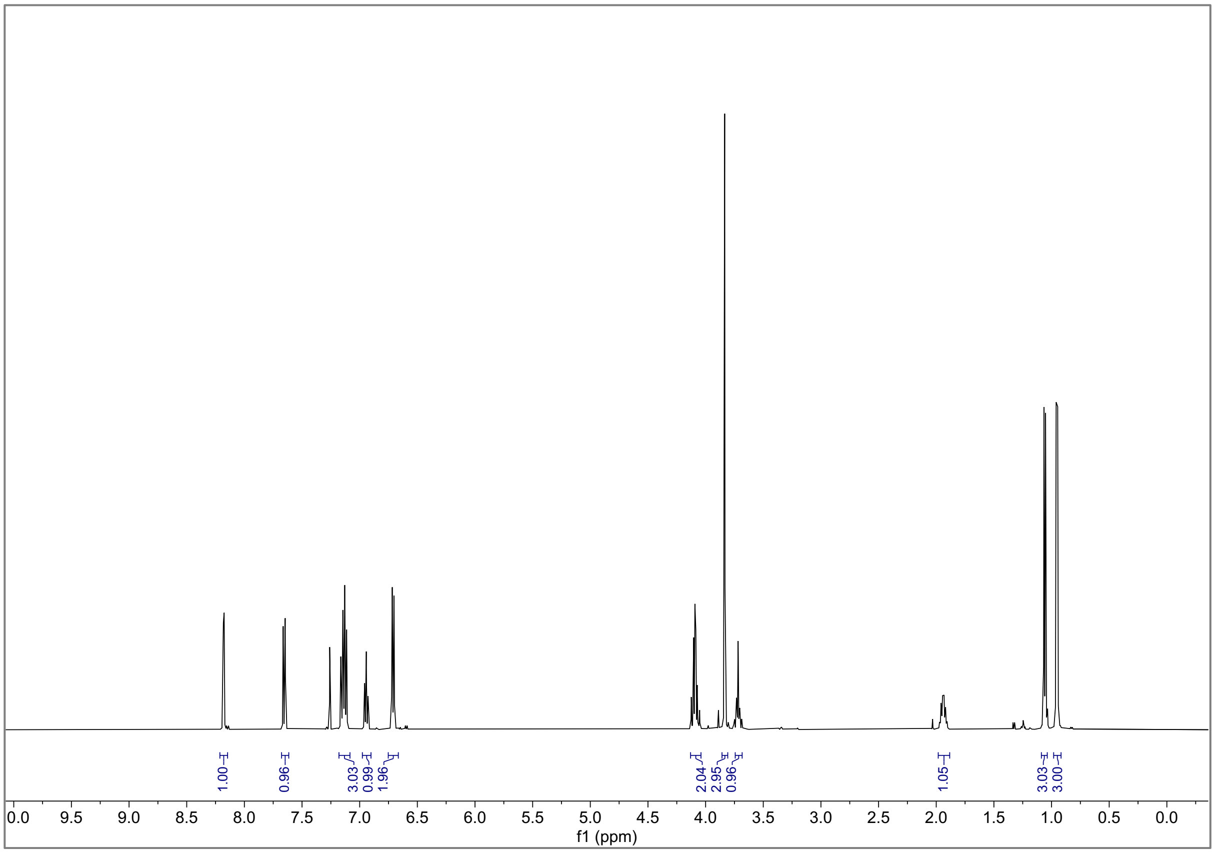

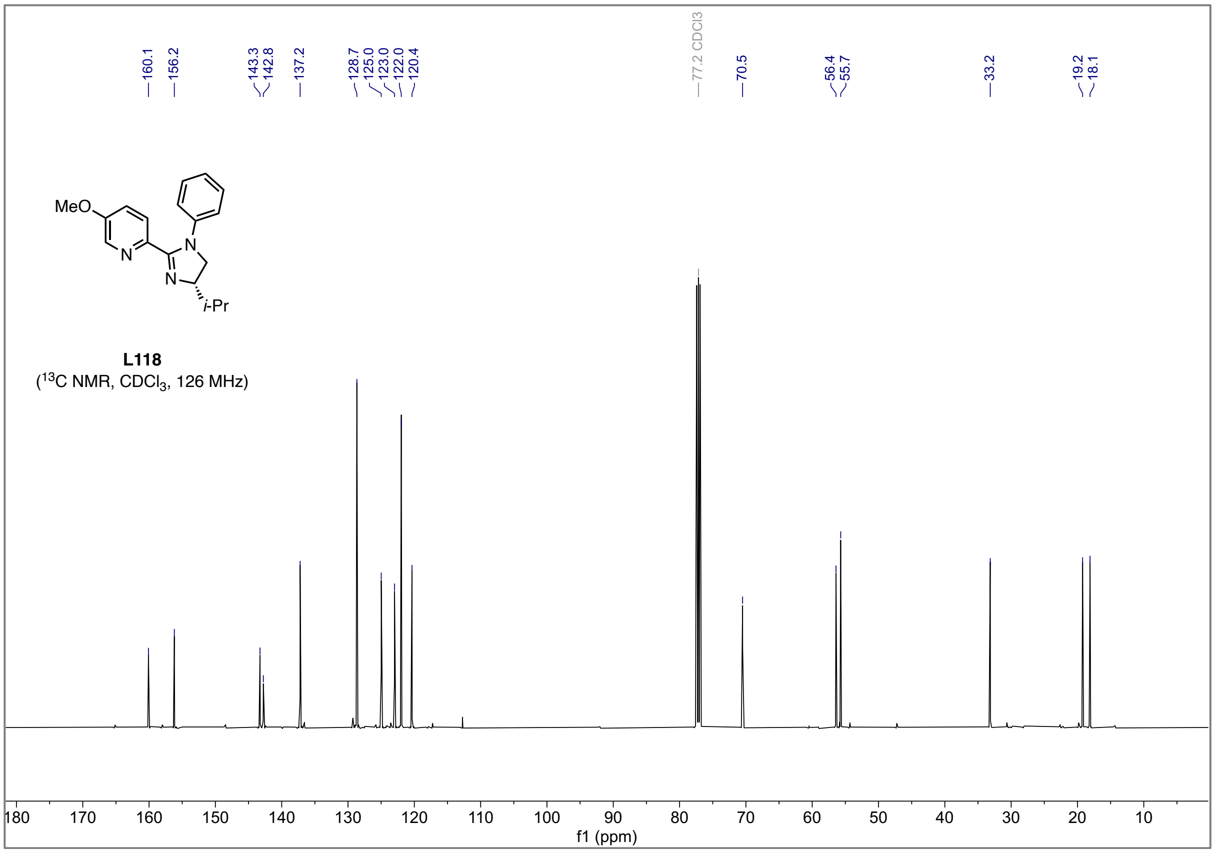

L118

(^{13}C NMR, $CDCl_3$, 126 MHz)

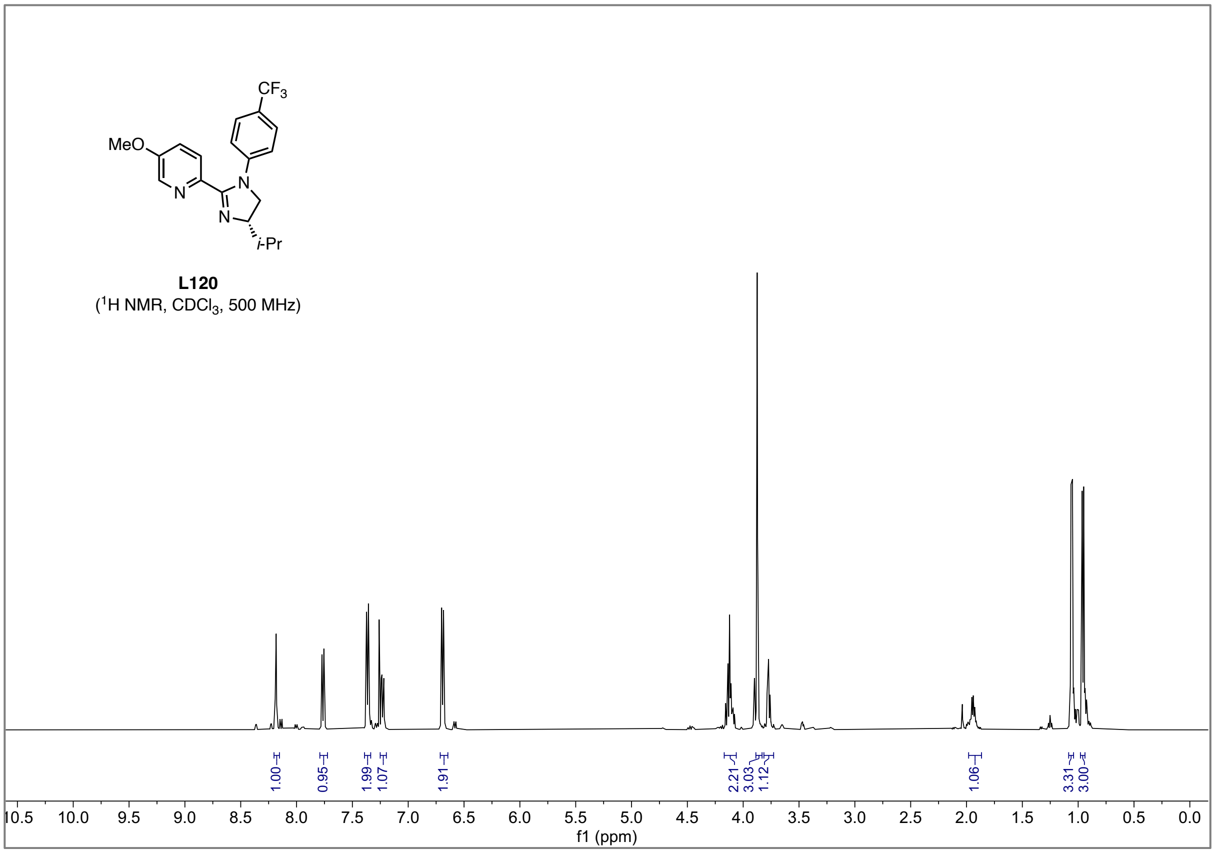
CF3
MeO
N
N
N
i-Pr
L120
(1H NMR, CDCl3, 500 MHz)
1.00
0.95
1.99
1.07
1.91
2.21
3.03
1.12
1.06
3.31
3.00
10.5 10.0 9.5 9.0 8.5 8.0 7.5 7.0 6.5 6.0 5.5 5.0 4.5 4.0 3.5 3.0 2.5 2.0 1.5 1.0 0.5 0.0
f1 (ppm)

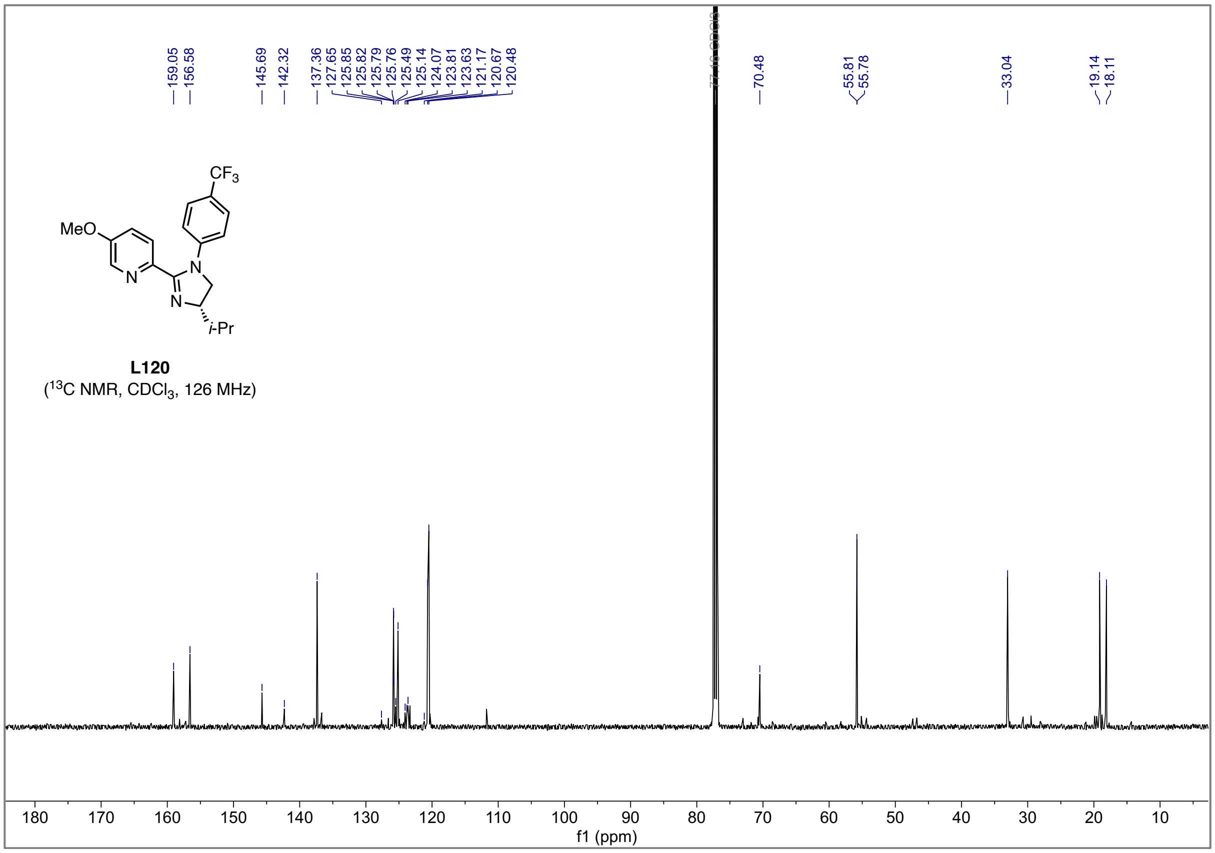
159.05
156.58
145.69
142.32
137.36
127.65
125.85
125.82
125.79
125.76
125.49
125.14
124.07
123.81
123.63
121.17
120.67
120.48
70.48
55.81
55.78
33.04
19.14
18.11
CF3
MeO
N
N
N
i-Pr
L120
(13C NMR, CDCl3, 126 MHz)
180 170 160 150 140 130 120 110 100 90 80 70 60 50 40 30 20 10
f1 (ppm)

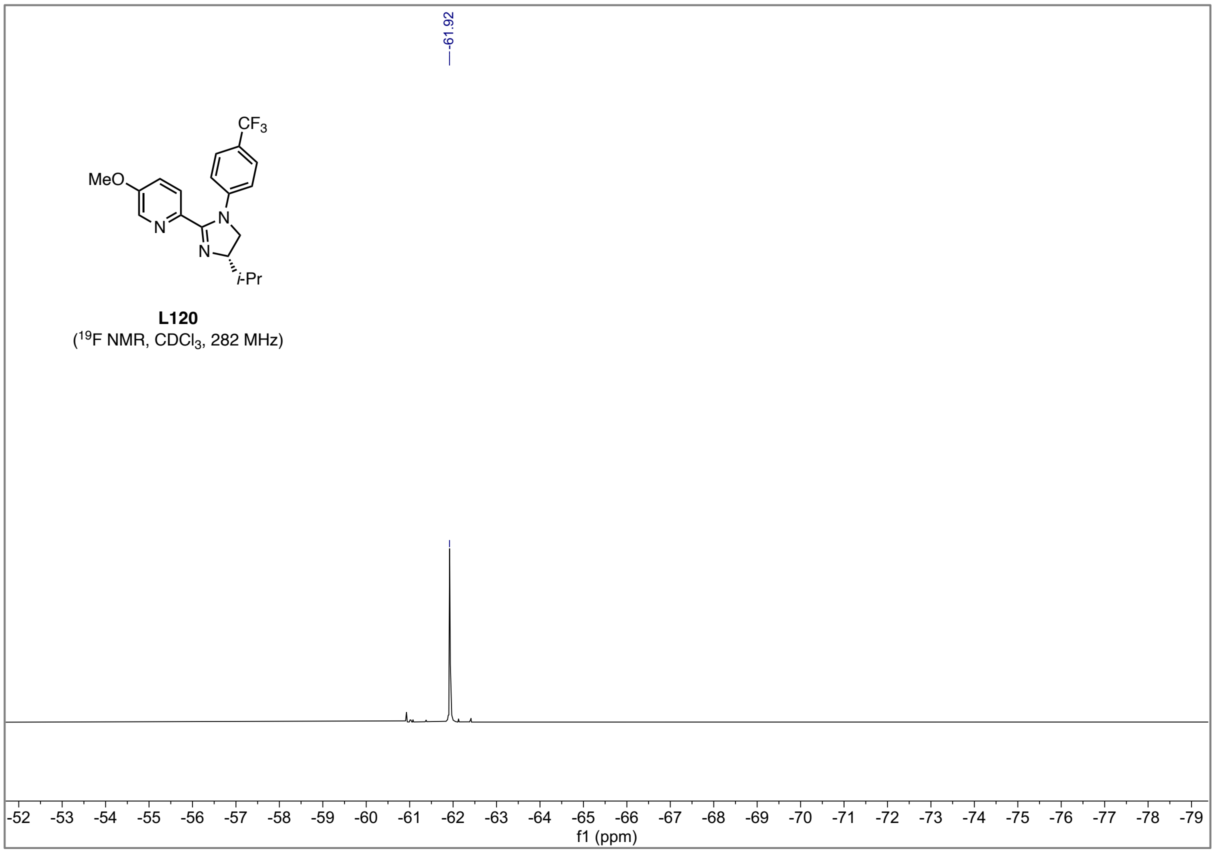
-61.92
CF3
MeO
N
N
N
i-Pr
L120
(19F NMR, CDCl3, 282 MHz)
-52 -53 -54 -55 -56 -57 -58 -59 -60 -61 -62 -63 -64 -65 -66 -67 -68 -69 -70 -71 -72 -73 -74 -75 -76 -77 -78 -79
f1 (ppm)

8. References

1. Lau, S. H. *et al.* Ni/Photoredox-Catalyzed Enantioselective Cross-Electrophile Coupling of Styrene Oxides with Aryl Iodides. *J. Am. Chem. Soc.* **143**, 15873–15881 (2021).
2. Haas, B. C. *et al.* Rapid prediction of conformationally-dependent DFT-level descriptors using graph neural networks for carboxylic acids and alkyl amines. *Digit. Discov.* **4**, 222–233 (2025).
3. Ingman, V. M., Schaefer, A. J., Andreola, L. R. & Wheeler, S. E. QChASM: Quantum chemistry automation and structure manipulation. *WIREs Comput. Mol. Sci.* **11**, e1510 (2021).
4. Cheng, X., Lu, H. & Lu, Z. Enantioselective benzylic C–H arylation via photoredox and nickel dual catalysis. *Nat. Commun.* **10**, 3549 (2019).
5. Huan, L., Shu, X., Zu, W., Zhong, D. & Huo, H. Asymmetric benzylic C(sp3)−H acylation via dual nickel and photoredox catalysis. *Nat. Commun.* **12**, 3536 (2021).
6. Cheng, X., Li, T., Liu, Y. & Lu, Z. Stereo- and Enantioselective Benzylic C–H Alkenylation via Photoredox/Nickel Dual Catalysis. *ACS Catal.* **11**, 11059–11065 (2021).
7. Wang, Y.-Z. *et al.* Nickel/biimidazole-catalyzed electrochemical enantioselective reductive cross-coupling of aryl aziridines with aryl iodides. *Nat. Commun.* **14**, 2322 (2023).
8. Hu, X., Cheng-Sánchez, I., Cuesta-Galisteo, S. & Nevado, C. Nickel-Catalyzed Enantioselective Electrochemical Reductive Cross-Coupling of Aryl Aziridines with Alkenyl Bromides. *J. Am. Chem. Soc.* **145**, 6270–6279 (2023).
9. Poremba, K. E., Kadunce, N. T., Suzuki, N., Cherney, A. H. & Reisman, S. E. Nickel-Catalyzed Asymmetric Reductive Cross-Coupling To Access 1,1-Diarylalkanes. *J. Am. Chem. Soc.* **139**, 5684–5687 (2017).
10. Cherney, A. H. & Reisman, S. E. Nickel-Catalyzed Asymmetric Reductive Cross-Coupling Between Vinyl and Benzyl Electrophiles. *J. Am. Chem. Soc.* **136**, 14365–14368 (2014).
11. Torres, J. A. G. *et al.* A Multi-Objective Active Learning Platform and Web App for Reaction Optimization. *J. Am. Chem. Soc.* **144**, 19999–20007 (2022).
12. Neugebauer, H., Badorf, B., Ehlert, S., Hansen, A. & Grimme, S. High-throughput screening of spin states for transition metal complexes with spin-polarized extended tight-binding methods. *J. Comput. Chem.* **44**, 2120–2129 (2023).
13. Zhao, Y. & Truhlar, D. G. The M06 suite of density functionals for main group thermochemistry, thermochemical kinetics, noncovalent interactions, excited states, and transition elements: two new functionals and systematic testing of four M06-class functionals and 12 other functionals. *Theor. Chem. Acc.* **120**, 215–241 (2008).
14. Zhao, Y. & Truhlar, D. G. Density Functionals with Broad Applicability in Chemistry. *Acc. Chem. Res.* **41**, 157–167 (2008).
15. Weigend, F. & Ahlrichs, R. Balanced basis sets of split valence, triple zeta valence and quadruple zeta valence quality for H to Rn: Design and assessment of accuracy. *Phys. Chem. Chem. Phys.* **7**, 3297–3305 (2005).
16. *xtb-gaussian*, https://github.com/aspuru-guzik-group/xtb-gaussian (accessed March 2024).
17. Laplaza, R., Wodrich, M. D. & Corminboeuf, C. Overcoming the Pitfalls of Computing Reaction Selectivity from Ensembles of Transition States. *J. Phys. Chem. Lett.* **15**, 7363–7370 (2024).
18. Sobez, J.-G. & Reiher, M. Molassembler: Molecular Graph Construction, Modification, and Conformer Generation for Inorganic and Organic Molecules. *J. Chem. Inf. Model.* **60**, 3884–3900 (2020).
19. Cao, Z. *et al.* COBRA web application to benchmark linear regression models for catalyst optimization with few-entry datasets. *Cell Rep. Phys. Sci.* **6**, 102348 (2025).
20. Ehehalt, L. E. *et al.* Cross-Electrophile Coupling: Principles, Methods, and Applications in Synthesis. *Chem. Rev.* **124**, 13397–13569 (2024).
21. Zhu, S., Zhao, X., Li, H. & Chu, L. Catalytic three-component dicarbofunctionalization reactions involving radical capture by nickel. *Chem. Soc. Rev.* **50**, 10836–10856 (2021).

22. Spielvogel, E. H., Yuan, J., Hoffmann, N. M. & Diao, T. Nickel-Mediated Radical Capture: Evidence for a Concerted Inner-Sphere Mechanism. *J. Am. Chem. Soc* (2025).
23. Becke, A. D. Density-functional thermochemistry. III. The role of exact exchange. *J. Chem. Phys.* **98**, 5648–5652 (1993).
24. Hehre, W. J., Stewart, R. F. & Pople, J. A. Self-Consistent Molecular-Orbital Methods. I. Use of Gaussian Expansions of Slater-Type Atomic Orbitals. *J. Chem. Phys.* **51**, 2657–2664 (1969).
25. Turro, R. F. *et al.* Mechanistic Investigation of Ni-Catalyzed Reductive Cross-Coupling of Alkenyl and Benzyl Electrophiles. *J. Am. Chem. Soc.* **145**, 14705–14715 (2023).
26. Souza, L. W. *et al.* Deconvoluting Nonlinear Catalyst–Substrate Effects in the Intramolecular Dirhodium-Catalyzed C–H Insertion of Donor/Donor Carbenes Using Data Science Tools. *ACS Catal.* **14**, 104–115 (2024).
27. Dalmau, D. & Alegre-Requena, J. V. ROBERT: Bridging the Gap Between Machine Learning and Chemistry. *WIREs Comput. Mol. Sci.* **14**, e1733 (2024).
28. Dalmau, D., Sigman, M. S. & Alegre-Requena, J. V. Machine learning workflows beyond linear models in low-data regimes. *Chem. Sci.* **16**, 8555–8560 (2025).
29. Dongbang, S. & Doyle, A. G. Ni/Photoredox-Catalyzed C(sp3)–C(sp3) Coupling between Aziridines and Acetals as Alcohol-Derived Alkyl Radical Precursors. *J. Am. Chem. Soc.* **144**, 20067–20077 (2022).
30. Arenas, I. *et al.* Syntheses of a Novel Fluorinated Trisphosphinoborate Ligand and Its Copper and Silver Complexes. Catalytic Activity toward Nitrene Transfer Reactions. *Inorg. Chem.* **53**, 3991–3999 (2014).
31. Woods, B. P., Orlandi, M., Huang, C.-Y., Sigman, M. S. & Doyle, A. G. Nickel-Catalyzed Enantioselective Reductive Cross-Coupling of Styrenyl Aziridines. *J. Am. Chem. Soc.* **139**, 5688–5691 (2017).
32. Arendt, K. M. & Doyle, A. G. Dialkyl Ether Formation by Nickel-Catalyzed Cross-Coupling of Acetals and Aryl Iodides. *Angew. Chem. Int. Ed.* **54**, 9876–9880 (2015).
33. Kim, E., Borden, M. A., Hwang, J., Doyle, A. G. & Dongbang, S. Ni-Catalyzed Reductive Coupling of Acetals with Anhydrides and Vinyl Triflates via Single-Electron C–O Activation. *Org. Lett.* **27**, 9454–9459 (2025).
34. Newman-Stonebraker, S. H. *et al.* Univariate classification of phosphine ligation state and reactivity in cross-coupling catalysis. *Science* **374**, 301–308 (2021).
35. Borden, M. A. Asymmetric Nickel-Catalyzed Cross-Coupling of Acetals and Epoxides. (Princeton University, 2020, http://arks.princeton.edu/ark:/88435/dsp010z709050b).
36. Lan, Y. *et al.* Nickel-Catalyzed Enantioselective C(sp3)–C(sp3) Cross-Electrophile Coupling of N-Sulfonyl Styrenyl Aziridines with Alkyl Bromides. *J. Am. Chem. Soc.* **146**, 25426–25432 (2024).
37. Zhou, J., Wang, D., Xu, W., Hu, Z. & XU, T. Enantioselective C(sp3)–C(sp3) Reductive Cross-Electrophile Coupling of Unactivated Alkyl Halides with α-Chloroboronates via Dual Nickel/Photoredox Catalysis. *J. Am. Chem. Soc.* **145**, 2081–2087 (2023).
38. DeLano, T. J. *et al.* Nickel-catalyzed asymmetric reductive cross-coupling of α-chloroesters with (hetero)aryl iodides. *Chem. Sci.* **12**, 7758–7762 (2021).
39. Apolinar, O. *et al.* Three-Component Asymmetric Ni-Catalyzed 1,2-Dicarbofunctionalization of Unactivated Alkenes via Stereoselective Migratory Insertion. *J. Am. Chem. Soc.* **144**, 19337–19343 (2022).
40. Boland, N. A. *et al.* Preparation of enantiopure biimidazoline ligands and their use in asymmetric catalysis. *Org. Biomol. Chem.* **2**, 1995–2002 (2004).
